# Probing substrate binding and release events in iridium-catalysed hydrogen isotope exchange reactions†

**DOI:** 10.1039/d5sc00759c

**Published:** 2025-07-01

**Authors:** Daria S. Timofeeva, William J. Kerr, David M. Lindsay, David J. Nelson

**Affiliations:** a Department of Pure & Applied Chemistry, University of Strathclyde 295 Cathedral Street Glasgow G1 1XL UK w.kerr@strath.ac.uk david.lindsay@strath.ac.uk david.nelson@strath.ac.uk

## Abstract

Directed, metal-catalysed C–H activation reactions rely on the binding of a Lewis basic functional group to the metal centre to ensure precise control of regioselectivity. However, groups that bind the metal centre too strongly have the potential to decrease turnover frequency and inhibit productive catalysis. Herein, we have used kinetic studies of iridium-catalysed hydrogen isotope exchange reactions, with NMR spectroscopy and mass spectrometry as the analytical techniques, to investigate the binding and release behaviour of a representative series of monosubsituted aromatic systems bearing a Lewis basic directing group. It was found that pyridine and pyrimidine exhibit anomalous behaviour, with a single-binding/dual labelling process dominating, or at least being competitive with, a binding/labelling/dissociation pathway. In contrast, with other directing groups (*e.g.* ketone, nitro, ester) initial formation of an appreciable population of *d*_1_-isotopologue is observed, and this is subsequently converted to the corresponding *d*_2_-isotopologue, suggesting a mainly binding/labelling/dissociation pathway. These data reveal three classes of substrate with rather different behaviour and for which reaction design and optimisation needs to be approached rather differently.

## Abstract 2

Mass spectrometry and computational chemistry show that, in iridium-catalysed hydrogen isotope exchange, some directing groups lead to doubly-deuterated products after one binding event, while others undergo dissociation and subsequent re-binding.

## Graphical abstract

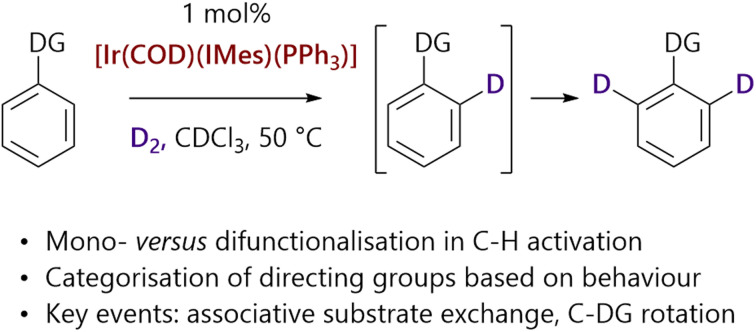

## Introduction

Within synthetic organic chemistry, the field of C–H activation remains an area with significant challenges and many opportunities.^1,2^ Achieving the desired regioselectivity within an organic molecule possessing multiple accessible C–H bonds, which are often present in rather similar environments, is a system-specific challenge that can be approached in several different ways, depending on the desired reaction outcome. One option is to deploy directing groups, typically Lewis basic in nature, which coordinate the metal centre and direct it to the desired site of activation and transformation.^3^ These can be functional units that are desired in the final product; in contrast, there are also an increasing number of reactions where the directing group is transient^4,5^ or cleaved after the C–H activation reaction, albeit at the cost of decreased overall atom economy.

**Scheme 1:**
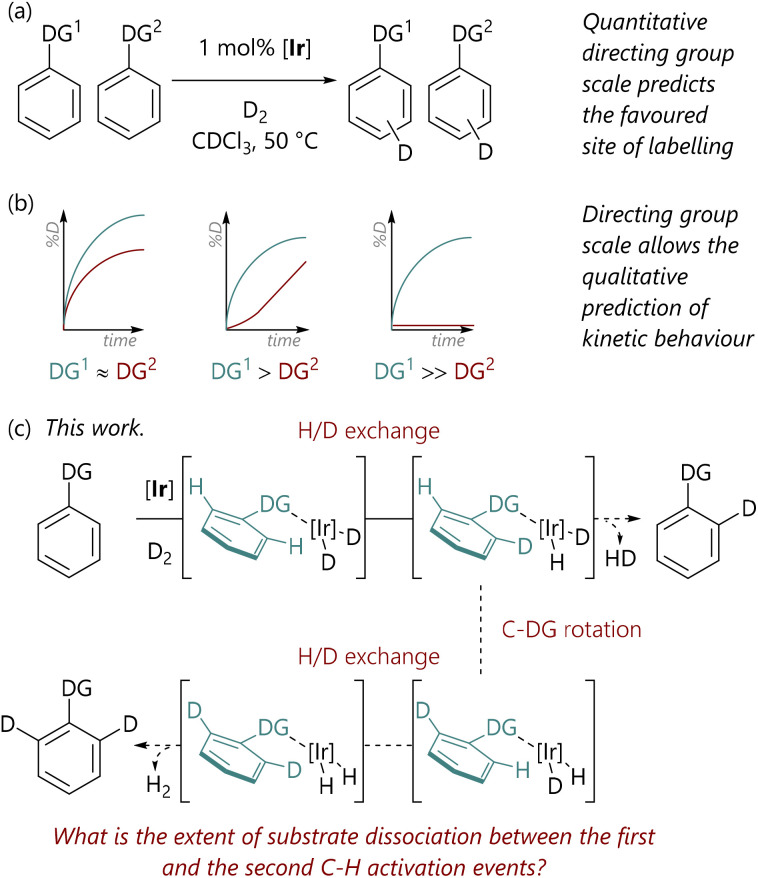
(a) Studies of directing group selectivity in iridium-catalysed hydrogen isotope exchange.^10^ (b) Kinetic studies of iridium-catalysed hydrogen isotope exchange.^11^ (c) This work.

In this domain, we have examined the competition between different directing groups in detail, initially using ruthenium-catalysed C–H arylation reactions as a model system,^6^ albeit with limited directing group scope. However, iridium-catalysed hydrogen isotope exchange (HIE) processes^7–9^ provide convenient and operationally simple model C–H activation systems for such studies, especially due to the wide scope and, generally, mild conditions of this reaction class. Related to this, we have recently used HIE systems to study the directing ability of various Lewis basic groups, noting differences in behaviour with Ir(i) catalysts bearing different ligand combinations (Scheme 1(a)).^10^ Furthermore, the data from directing group reactivity scales were used to qualitatively predict the behaviour of reactions in which there are multiple possible directing groups (Scheme 1(b)).^11^ Notably, Valero *et al.* have also qualitatively explored directing group strength in Ir(i)-catalysed HIE, and used DFT calculations to rationalise the experimental data.^12^

As part of our earlier studies, we previously reported preliminary data from mass spectrometry analysis of samples withdrawn at different times during iridium-catalysed HIE reactions;^11^ these analyses allowed the isotopologue composition (*d*_0_, *d*_1_, *d*_2_) to be quantified at each timepoint. These data suggested that for 2-phenylpyridine, there were typically two labelling events per binding event, since the concentration of phenylpyridine-*d*_1_ isotopologue remained low throughout the reaction. Given that the relationship between substrate binding and C–H activation is critical to the control of these transformations, we elected to study these aspects of the HIE process in significantly more depth.

Our initial hypothesis was that the observed isotopologue profiles ought to be correlated with the strength of the interaction between the directing group and the iridium centre; it was proposed that substrates with stronger directing groups would exchange less rapidly and, therefore, would undergo two isotopic labelling events before exchange with unlabelled substrate. Herein, we delineate the quantification of the isotopologue distribution as a function of reaction progress for eight substrates, representing a range of Lewis basicities, which, in turn, delivers a deeper understanding of the influence of directing group on the balance between substrate binding and C–H activation (Scheme 1(c)). Additionally, we have deployed DFT calculations to examine the underlying processes in a level of detail that complements our experimental studies.

## Results and discussion

### Profiling isotopologue populations for different directing groups

Experimental work began with a systematic study of eight monosubstituted aryl systems (1–8) bearing Lewis basic directing groups of varying strengths, and how the molar fraction of the *d*_0_-, *d*_1_-, and *d*_2_-isotopologues varies with time in the corresponding HIE reactions. Literature data are available to quantify the Lewis basicity of some of the functional groups employed.^13–17^ These data are available for a subset of the specific substrates considered here and for their parent directing groups (Table S27 in the ESI†); these illustrate the Lewis basicity range of the substrates, however, none of these metrics correlate directly to our recently established relative directing ability of the corresponding substrates.^10^ This was the first hint that the behaviour of these systems was more complicated than we had initially anticipated.

**Scheme 2:**
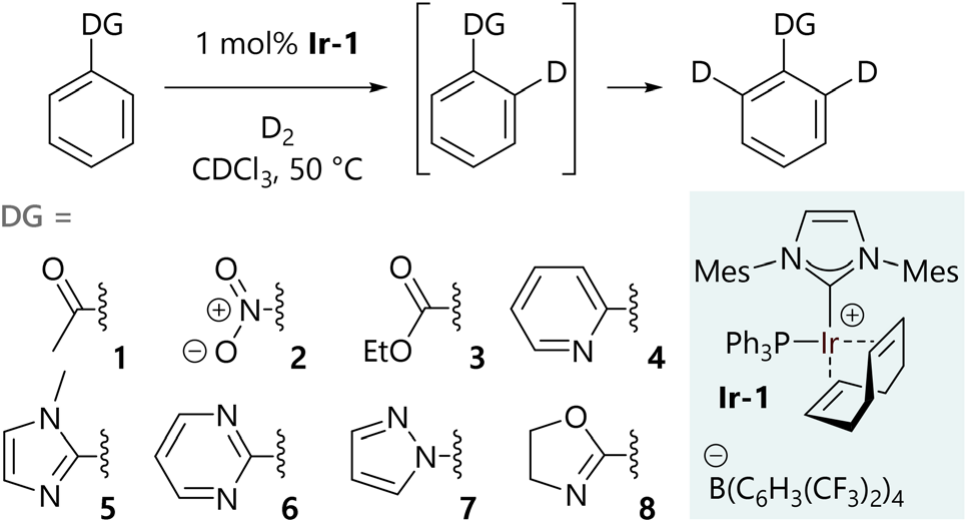
Substrates profiled to determine isotopologue content as a function of time.

The hydrogen isotope exchange reaction conditions that were used to collect isotopologue profiles are shown in Scheme 2. A 1 mol% loading of iridium catalyst Ir-1 was selected to ensure good levels of deuterium incorporation during the reaction, while limiting the rate to allow sufficient data density to be achieved by manual sampling of each reaction (full experimental details are described in the ESI†). All reactions were carried out under identical conditions in glassware of the same size and shape. Our catalyst Ir-1 was selected due to its widespread use in a range of labelling reactions^9^ as a result of its high turnover frequency. Additionally, we have previously assembled a comprehensive set of data related to directing group selectivity using complex Ir-1.^10,11^ Isotopic labelling was only observed *ortho* to the directing group in each case and not, for example, on the directing groups themselves.

The selected substrates represent a diverse range of directing group ability. The directing group strength, quantified relative to 2-phenylpyridine (4), decreases in the order 1-methyl-2-phenylimidazole (5) (10.13) >> 2-phenyloxazoline (8) (2.80) ≈ 2-phenylpyrimidine (6) (2.66) > 1-phenylpyrazole (7) (1.77) > 2-phenylpyridine (4) (1.00) >> acetophenone (1) (0.06) > nitrobenzene (2) (0.03) > ethyl benzoate (3) (0.01).^10^

**Fig. 1:**
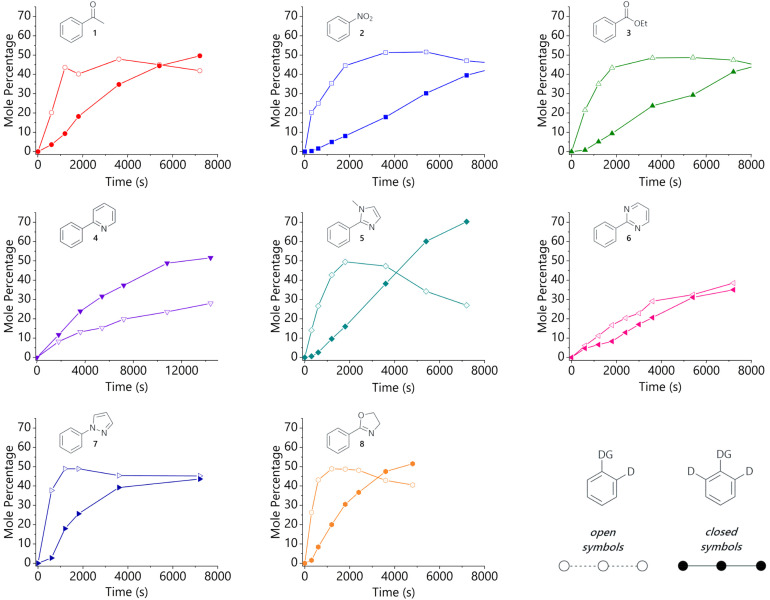
Isotopologue profiles for the hydrogen isotope exchange reactions of: acetophenone (red circles), nitrobenzene (blue squares), ethyl benzoate (green triangles), 2-phenylpyridine (purple triangles), 1-methyl-2-phenylimidazole (teal diamonds), 2-phenylpyrimidine (pink triangles), 1-phenylpyrazole (dark blue triangles), and 2-phenyloxazoline (orange hexagons). For each substrate, the *d*_1_-isotopologue is shown using open shapes and the *d*_2_-isotopologue is shown using filled shapes.

The isotopologue time course data for the labelling of each substrate are presented in Fig. 1. These are plotted as mole percent of (singly or doubly) deuterated substrate, *i.e.* the percentage of [*d*_0_-substrate]_*t*=0_ that is present as *d*_1_-substrate or *d*_2_-substrate at each timepoint. The same timescale was used throughout this figure to allow for the ready comparison of directing groups, but full reaction profiles can be found in the ESI.† Samples from reactions with heterocyclic substrates were analysed by high-performance liquid chromatography coupled to electrospray ionisation mass spectrometry (LC-ESI-MS), while those from the reactions of acetophenone, nitrobenzene, and ethyl benzoate were analysed by gas chromatography coupled to electron impact ionisation mass spectrometry (GC-EI-MS). The relative concentration of each isotopologue was determined by integration of the mass spectra, after corrections to account for the ^13^C content of the starting material.† The levels of overall deuterium incorporation determined from mass spectrometry data agree well with data obtained for the same systems by integration of the ^1^H NMR spectra of aliquots withdrawn at the same timepoints.†

Analysis of the HIE reactions of non-heterocyclic substrates reveals an initial spike in *d*_1_-isotopologue, peaking at *ca*. 50% of the total substrate, followed by the gradual conversion of the *d*_1_- into the *d*_2_-isotopologue. Acetophenone (1) is the most strongly directing of this substrate sub-class and shows the most rapid formation of *d*_2_-isotopologue. Substrates with a five-membered heterocycle directing group (5, 7, 8) also show a pronounced peak in *d*_1_-isotopologue, again at *ca*. 50% of the total substrate, but notably earlier in the reaction than the non-heterocyclic directing groups (1, 2, 3). These trends are broadly consistent with our initial hypothesis, as the heterocyclic directing groups are stronger than the non-heterocyclic directing units.

However, pyridine and pyrimidine directing groups behave somewhat differently to the other substrates that were studied and show that the situation is rather more complex. The labelling reaction of 2-phenylpyrimidine (6) proceeds with almost identical levels of *d*_1_- and *d*_2_-isotopologues as the reaction evolves; 2-phenylpyridine (4) shows both the *d*_1_- and *d*_2_-isotopologue levels increasing steadily as the reaction proceeds, with *d*_2_-4 growing slightly faster than *d*_1_-4.

**Fig. 2:**
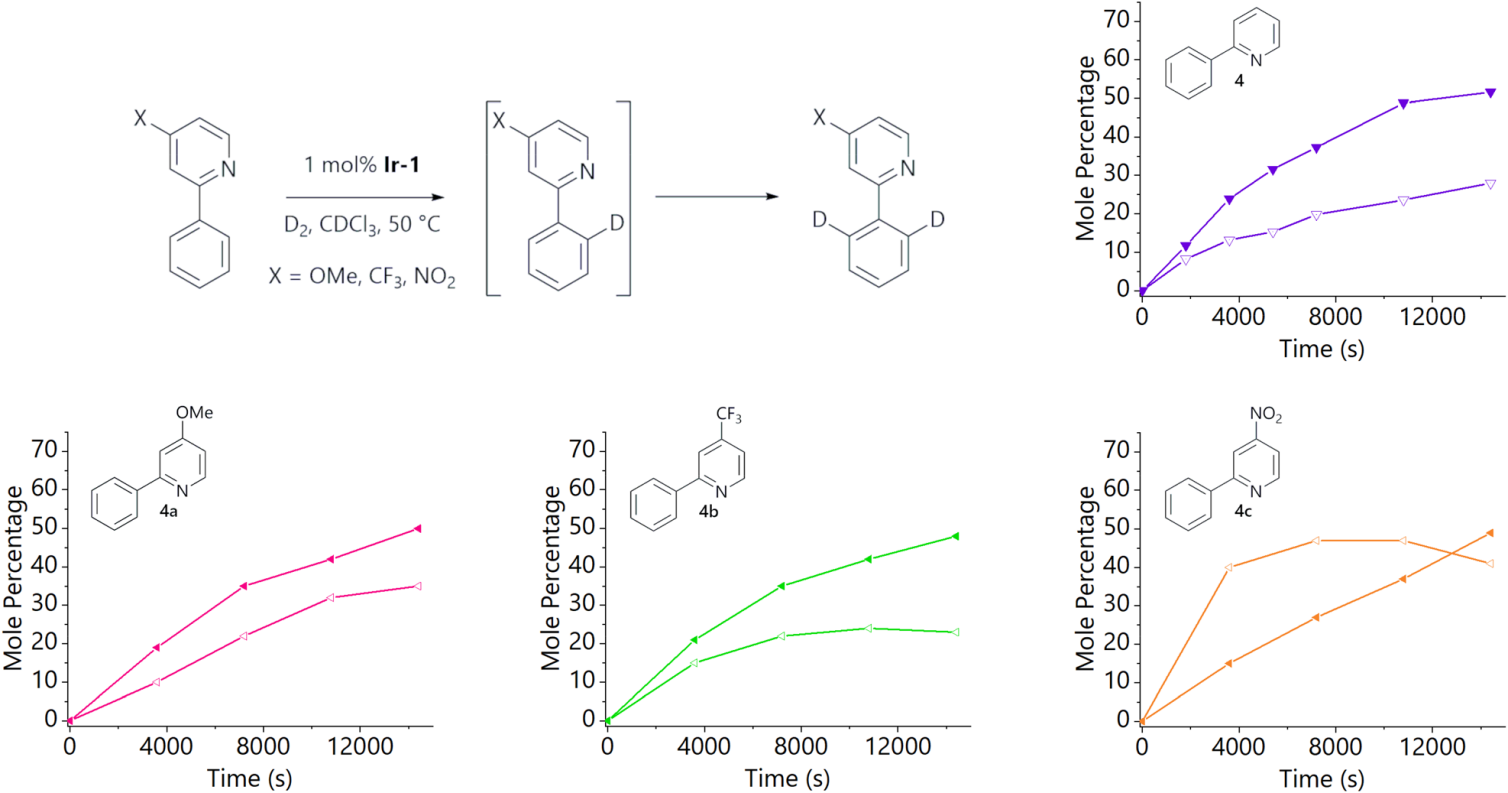
Isotopologue profiles for the hydrogen isotope exchange reactions of: 2-phenylpyridine (purple triangles; top right; reproduced from Fig. 1), 2-phenyl-4-methoxypyridine (pink triangles; bottom left), 2-phenyl-4-(trifluoromethyl)pyridine (green triangles; bottom middle), and 2-phenyl-4-nitropyridine (orange triangles; bottom right). For each substrate, the *d*_1_-isotopologue is shown using open shapes and the *d*_2_-isotopologue is shown using filled shapes.

To further probe this distinct behaviour of 2-phenylpyridine (4), several substituted derivatives were prepared in order to fine-tune the Lewis basicity of the pyridine nitrogen: 2-phenyl-4-methoxypyridine (4a), 2-phenyl-4-(trifluoromethyl)pyridine (4b), and 2-phenyl-4-nitropyridine (4c). These were prepared *via* palladium-catalysed cross-coupling reactions from the corresponding 2-chloropyridine analogue and phenylboronic acid.† The labelling reactions of 4a–c were carried out in the same manner as those described above, with the data shown in Fig. 2. LC-ESI-MS analysis again allowed the isotopologue distribution in each reaction to be profiled over time. Our working hypothesis was that the more electron-rich congener (4a, X = OMe, *σ*_p_ = −0.27)^18^ should lead to stronger binding to the metal centre, and therefore increase the proportion of *d*_2_-isotopologue relative to *d*_1_; the opposite effect should be observed as electron density is withdrawn from the pyridine moiety (4b, X = CF_3_, *σ*_p_ = 0.54; 4c, X = NO_2_, *σ*_p_ = 0.78).^18^ Indeed, the profiles from these reactions are qualitatively in agreement with this hypothesis. Most notably, there is a significant change in behaviour for the least electron-rich substrate (4c), which is consistent with weaker binding; an initial and rapid increase in concentration of *d*_1_-4c is observed, similar to the non-heterocyclic directing groups, with *d*_2_-4c forming more slowly. However, 4a (X = OMe) and 4b (X = CF_3_) behave similarly to the parent 2-phenylpyridine (4), with relatively small differences in rate and selectivity.

No *d*_3_- or *d*_4_-isotopologue was detected for the nitro-substituted analogue, suggesting negligible nitro-directed HIE; this is consistent with our previous observations regarding the kinetic behaviour of HIE reactions of substrates with multiple potential directing groups.^11^ No deuterium incorporation at the 6-position of 2-phenylpyridine (4) or substituted analogues (4a–c) was detected by ^1^H NMR analysis, and no *d*_3_-isotopomer was detected by mass spectrometry.

Computational chemistry studies were carried out to gain further insight into the processes occurring during the reaction.‡‡A full conformational search for each intermediate was carried out using CREST and xTB.^39,40^ Geometry optimisations were carried out (without a solvent model) using the M06-L functional,^28^ the SDD basis set/ECP on iridium,^29^ and the 6-311G(d,p) basis set^41,42^ on all other atoms. Energies were refined using single point calculations at M06-L/Def2-QZVP with the SMD solvation model (with chloroform or DCM solvent, as appropriate).^24^ Quasi-harmonic corrections to entropy and enthalpy were applied using GoodVibes.^25^ Further details of the level of theory can be found in the ESI. Methyl benzoate was used as a model for ethyl benzoate to decrease conformational complexity. The BAr^F^_24_ counterion was not modelled, and only the cationic iridium fragment is considered throughout (BAr^F^_24_ = tetrakis(3,5-trifluoromethylphenyl)borate. DFT data (coordinates, energies, *etc.*) can be accessed either *via* the ESI or *via* ioChem-BD^43^ (DOI: https://doi.org/10.19061/iochem-bd-6-541). We have previously used DFT calculations to rationalise site-selectivity in several studies.^19–22^ As part of this investigation, only iridium protide complexes are considered in the calculations; in practice, the reactions involve iridium protide and deuteride complexes, but our focus here is on the events around substrate binding. Pathways proceeding *via trans*-dihydride complexes are not considered because these are likely to be considerably higher in energy than the corresponding *cis*-dihydride isomers due to the strong *trans*-effect of the hydride ligands.^23^ The model systems are considered in chloroform solution using the (implicit) SMD model^24^ and with explicit chloroform microsolvation where solvent binding to iridium is considered; additionally, data for DCM complexes and in DCM solvent can be found in the ESI,† with the same trends apparent in the DCM data as in the chloroform study. Quasi-harmonic corrections, applied using GoodVibes,^25^ are used to better describe the contributions of low frequency vibrations to the free energy, specifically the Grimme correction to entropy^26^ and the Head-Gordon correction to enthalpy.^27^ Free energies at 323.15 K and 1 mol L^−1^ using the M06-L/Def2-QZVP/SMD(chloroform)//M06-L/6-311G(d,p),SDD level of theory are reported unless otherwise stated, and we refer to this as M06-L/BS2(chloroform) for simplicity; free energies (at 323.15 K and 1 mol L^−1^)§These calculations were carried out using the freqchk utility provided as part of the Gaussian16 software package; they are corrected for temperature (323.15 K) but quasi-harmonic corrections to entropy and enthalpy are not applied. at the M06-L/6-311G(d,p),SDD/SMD(chloroform)//M06-L/6-311G(d,p),SDD level of theory are provided in parentheses, and we term this M06-L/BS1(chloroform).^28–32^ EDA calculations were carried out using the M06-L/Def2-QZVP//M06-L/6-311G(d,p),SDD level of theory (or M06-L/BS2) and do not include a solvent model.

**Fig. 3:**
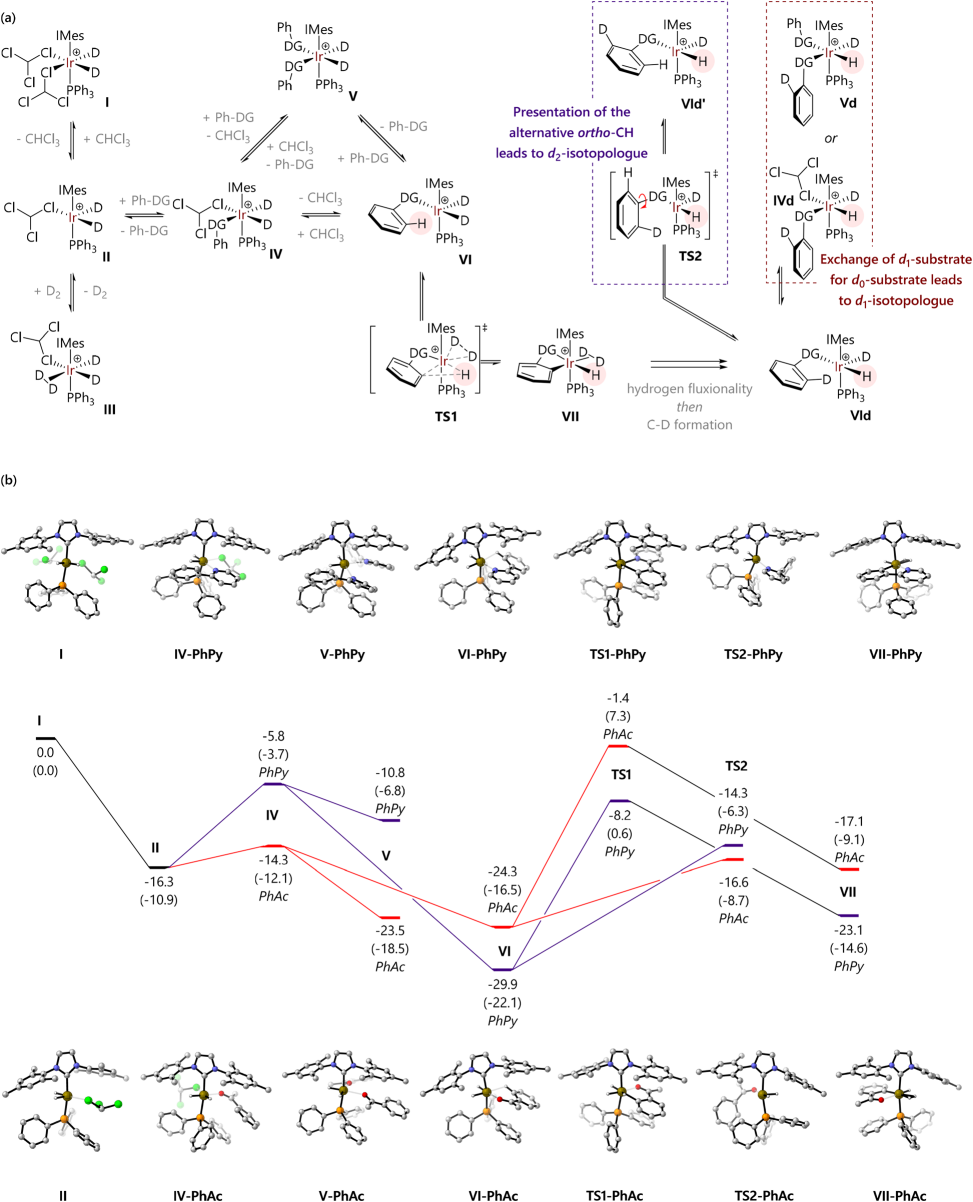
(a) Reaction mechanisms for the hydrogen isotope reactions studied in this work. (b) Free energy profiles for the reactions of 2-phenylpyridine and acetophenone with [Ir(H)_2_(CHCl_3_)(IMes)(PPh_3_)]^+^ (I); free energies are quoted in kcal mol^−1^ relative to species I at the M06-L/BS2(chloroform) level of theory, with free energies at the M06-L/BS1(chloroform) level of theory quoted in parentheses.

The possible mechanistic pathways are outlined in Fig. 3(a), based on substantial previous experimental and computational work to understand the catalytic cycle and structure/reactivity relationships of HIE mediated by pre-catalysts such as Ir-1.^33^ [IrD_2_(solvent)_2_(IMes)(PPh_3_)]^+^ (I) forms when Ir-1 is exposed to deuterium,^33^ and will then catalyse HIE. Dissociation of one solvent ligand would generate coordinatively unsaturated 16e species II, which might bind chloroform in *κ*^1^-Cl, *κ*^2^-Cl,Cl, or *κ*^2^-Cl,H coordination modes. Species II can, in turn, bind dideuterium to form III, or bind substrate to form IV. Complex IV must then progress – either dissociatively *via* loss of solvent or associatively *via*V – to form agostic complex VI. Complex VI can then undergo C–H activation *via*TS1 to form the key iridacycle VII. The bound deuterium in complex VII would then exchange into the product *via* a ‘hydrogen fluxionality’ transition state and C–D bond formation, in the microscopic reverse of the C–H activation process. The resulting species VId might progress in two ways:

(1) An exchange of *d*_1_-substrate for *d*_0_-substrate *via*IVd and/or Vd (red pathway). This would result in the release of the *d*_1_-substrate and coordination of a new molecule of *d*_0_-substrate, until the population of *d*_1_-substrate becomes sufficiently high to compete.

(2) Rotation of the *ipso*-C–DG bond (TS2) would present the second *ortho*-C–H bond to the iridium centre (VId′) (purple pathway), without release of the substrate. This pathway would ultimately result in formation and release of the *d*_2_-substrate.

Our hypothesis was therefore that the competition between these possible processes occurring from VId (substrate exchange *vs.* a second labelling event) might explain our experimental observations regarding labelling selectivity.

Fig. 3(b) shows free energy profiles for model substrates 2-phenylpyridine and acetophenone. Complex I is octahedral at iridium, while II is square-based pyramidal; attempts to locate isomers of II with bidentate chloroform ligands (*κ*^2^-Cl,Cl or *κ*^2^-Cl,H) gave one example (of the former) but this was *ca*. 5 kcal mol^−1^ higher in energy. For 2-phenylpyridine, (productive) complex VI is lower in energy than [IrH_2_(PhPy)_2_(IMes)(PPh_3_)] (V); for acetophenone, V and VI are of similar energy. Thereafter, the barriers to C–H activation (TS1) are similar for both substrates. These data support a scenario where substrate exchange is more readily achieved for acetophenone than for 2-phenylpyridine: IV and V might each present routes for the replacement of *d*_1_- or *d*_2_-substrate by *d*_0_-substrate, but these are much higher in energy for 2-phenylpyridine than for acetophenone. The 2-phenylpyridine system is perhaps more likely to undergo phenyl rotation – *via*TS2 – presenting the iridium centre with a second C–^1^H bond and allowing a second labelling event to occur before substrate dissociation.

As these calculations do not consider deuterated isotopomers, key transition states TS1 and TS2 were checked to consider possible isotope effects (Table 1).§ Kinetic isotope effects (KIEs) were calculated using eqn (1) and assume that transmission coefficient *κ* is independent of isotopologue.1
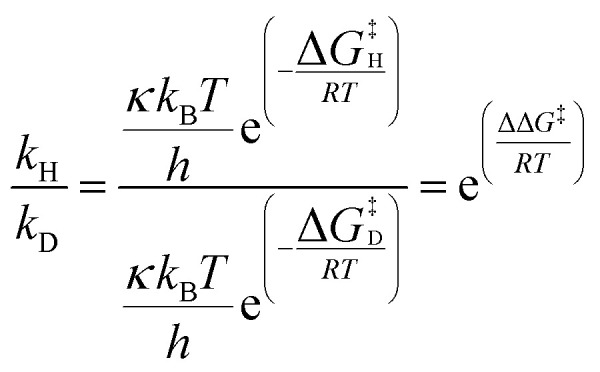


**Table 1:** Isotope effects on TS1 and TS2 for acetophenone (1) and 2-phenylpyridine (4), at the M06-L/BS2(chloroform) level of theory, at 323.15 K, without quasi-harmonic corrections to entropy and enthalpy

Substrate	TS	Iridium fragment	ΔΔ*G*^‡^*vs. d*_0_-TS (kcal mol^−1^)	Calculated KIE
Acetophenone-*d*_0_	TS1	[Ir(D)_2_(IMes)(PPh_3_)]	0.08	1.14
Acetophenone-*d*_1_^a^	TS1	[Ir(H)(D)(IMes)(PPh_3_)]^b^	0.78	3.76
Acetophenone-*d*_1_^a^	TS2	[Ir(H)(D)(IMes)(PPh_3_)]^b^	−0.01	0.98
2-Phenylpyridine-*d*_0_	TS1	[Ir(D)_2_(IMes)(PPh_3_)]	0.04	1.07
2-Phenylpyridine-*d*_1_^a^	TS1	[Ir(H)(D)(IMes)(PPh_3_)]^b^	0.84	4.15
2-Phenylpyridine-*d*_1_^a^	TS2	[Ir(H)(D)(IMes)(PPh_3_)]^b^	0.01	1.02

a Deuterium located on the phenyl ring, *ortho* to the directing group.

b Deuterium located *trans* to the developing Ir–C bond and *cis* to the directing group.

TS1 for the reaction of the iridium dideuteride with acetophenone or 2-phenylpyridine would give rise to a moderate secondary KIE, while the reaction of the iridium hydridodeuteride with the *d*_1_-substrates – produced after one labelling event – would give a primary KIE. This is consistent with what would be expected for reactions involving metal hydride intermediates.^34^ Much smaller effects are seen for TS2, with energy differences on the order of *ca.* 0.01 kcal mol^−1^ and very small secondary KIEs; therefore, the competition between TS2 and exchange of *d*_1_-substrate for *d*_0_-substrate is not sensitive to the presence of a ^2^H isotope.

**Fig. 4:**
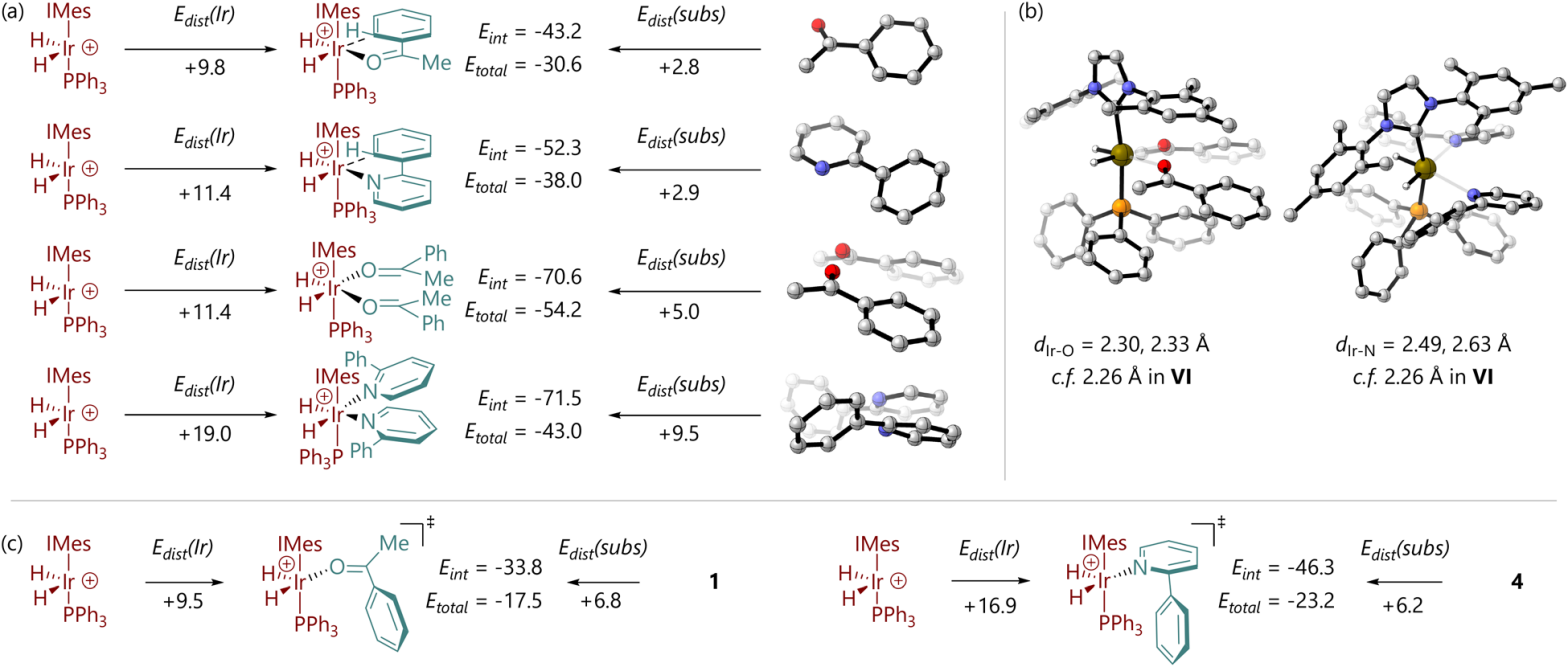
(a) Energy decomposition analysis of substrate binding in [Ir(H)_2_(substrate)_2_(IMes)(PPh_3_)] (V) and [Ir(H)_2_(substrate)(IMes)(PPh_3_)] (VI) complexes for acetophenone and 2-phenylpyridine. (b) Molecular structures of complexes V and VI for acetophenone and 2-phenylpyridine, indicating Ir–O and Ir–N distances, respectively. (c) Energy decomposition analysis of TS2 structures for acetophenone and 2-phenylpyridine. All energies are electronic energies in kcal mol^−1^, without a solvent model, at M06-L/BS2.

Energy decomposition analysis (EDA) was conducted for species V and VI, for both of the same two substrates, to interrogate the rather large difference in their relative energies for species 1 and 4. Note that these calculations make use of electronic energy (*E*) rather than free energy (*G*), and were carried out at the M06-L/BS2 level of theory without a solvent model. An optimised structure for [Ir(H)_2_(IMes)(PPh_3_)]^+^ (VIII) was obtained, and single point calculations were carried out on the [Ir(H)_2_(IMes)(PPh_3_)]^+^ and substrate fragments from V and VI. This quantified the distortion energy of the iridium fragment (*E*_dist_(Ir)) and substrate(s) (*E*_dist_(subs)), as well as the energy released by the interaction of these fragments (*E*_int_) (Fig. 4(a)). For VI, both acetophenone (1) and 2-phenylpyridine (4) are distorted to a similar extent to bind iridium; [IrH_2_(IMes)(PPh_3_)]^+^ is distorted similarly for each substrate. However, the pyridine complex has a greater *E*_int_. The two molecules of 2-phenylpyridine in V suffer more distortion compared to the two molecules of acetophenone (Δ*E*_dist_(subs) = 4.5 kcal mol^−1^), and the [Ir(H)_2_(IMes)(PPh_3_)]^+^ fragment in the 2-phenylpyridine complex is also considerably more distorted (Δ*E*_dist_(Ir) = 7.6 kcal mol^−1^). Inspection of the molecular structures shows the rather different way in which these two substrates bind (Fig. 4(b)). The acetophenone complex places the phenyl groups outside the iridium coordination sphere, and the Ir–O bonds are only slightly longer in V than in VI (2.30, 2.33 Å, *vs.* 2.26 Å). The required arrangement for 2-phenylpyridine binding places the phenyl substituents close to iridium, and the Ir–N bond lengths are long (2.49, 2.63 Å *vs.* 2.26 Å in VI). The steric clash between phenyl groups, evident from increased *E*_dist_ for the fragment containing the two 2-phenylpyridine molecules, clearly prevents tighter Ir–N binding and decreases the magnitude of *E*_int_ as a result. *E*_int_ for V is similar for both substrates. EDA therefore reveals that, for 2-phenylpyridine, V is higher in energy than VI because binding of two substrates introduces substantial strain, limiting the energy released upon binding of the directing group. However, for acetophenone, effective binding of two substrate molecules is possible.

A similar analysis was carried out for TS2 (Fig. 4(c)). For acetophenone (1), while the iridium fragment is not considerably more distorted than in VI, the substrate suffers an additional 4.0 kcal mol^−1^ energetic penalty; the interaction energy between the two fragments is also 9.4 kcal mol^−1^ less favourable. For 2-phenylpyridine (4) the iridium fragment in TS2 is further distorted compared to VI, costing 5.4 kcal mol^−1^, the substrate suffers an additional 3.3 kcal mol^−1^ of distortion, and the interaction energy suffers a 6.0 kcal mol^−1^ penalty. In both cases, the relatively high energy of TS2 is proposed to be due to the need to loosen the binding of the substrate in order to afford it the freedom to rotate between the relatively bulky umbrella-shaped IMes ligand and cone-shaped phosphine ligand.

**Fig. 5:**
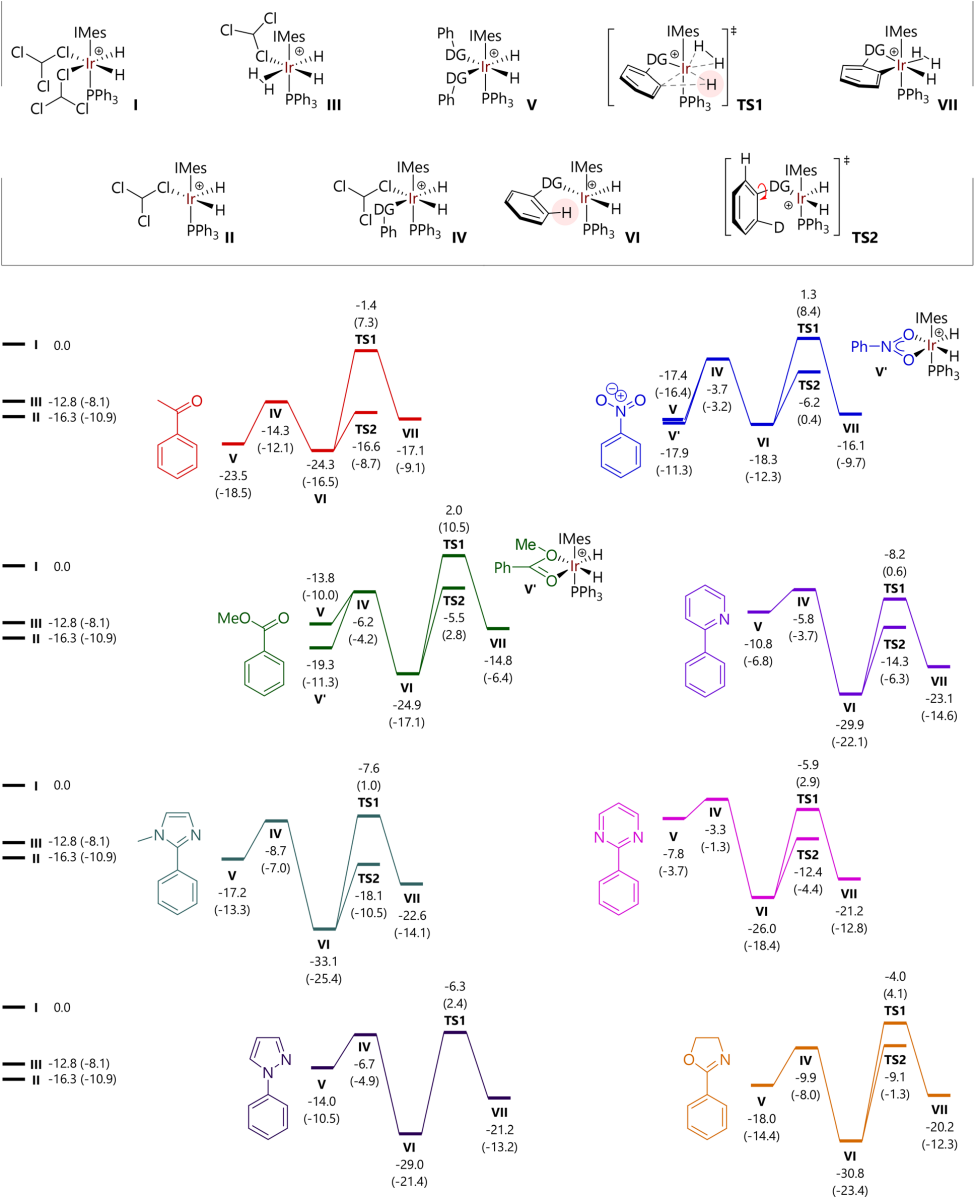
Free energy profiles for C–H activation reactions at the M06-L/BS2(chloroform) level of theory. Free energies are in kcal mol^−1^ relative to structure I and include quasi-harmonic corrections to entropy and enthalpy. Free energies at the M06-L/BS1 (chloroform) level of theory are provided in parentheses.

Free energy profiles for a further six of the substrates studied experimentally are shown in Fig. 5; data for acetophenone (1) and 2-phenylpyridine (4) are reproduced within the same figure for comparison. The free energies of [IrH_2_(CHCl_3_)_2_(IMes)(PPh_3_)]^+^ (I), [IrH_2_(CHCl_3_)(IMes)(PPh_3_)]^+^ (II), and [IrH_2_(CHCl_3_)(H_2_)(IMes)(PPh_3_)]^+^ (III) are also indicated.

For each substrate, the displacement of one CHCl_3_ molecule from I to form IV is exergonic (Δ*G* = −3.3 to −14.3 kcal mol^−1^). Complex IV is typically higher in energy than II, so a dissociative mechanism for the displacement of CHCl_3_ may be preferred.

The free energies of *cis*-[IrH_2_(substrate)_2_(IMes)(PPh_3_)]^+^ complexes (V) depend acutely on substrate structure. Acetophenone (1) and nitrobenzene (2) lead to complexes V that are energetically competitive with the agostic complexes [IrH_2_(substrate)(IMes)(PPh_3_)]^+^ (VI) because the phenyl group can be placed outside of the coordination sphere of iridium. Complex V for methyl benzoate is somewhat higher in energy because of the different orientation of the substrate molecules, so that the H_3_CO fragment is placed away from iridium. Complexes V containing N-heterocyclic directing substrates are considerably higher in energy than the corresponding complexes VI.

Structures were also located where nitrobenzene and methyl benzoate could bind the [IrH_2_(IMes)(PPh_3_)]^+^ fragment *via* both oxygen atoms. However, the free energies of [IrH_2_(*κ*^2^-O,O-PhCO_2_Me)(IMes)(PPh_3_)]^+^ (V′-PhCO_2_Me) and [IrH_2_(*κ*^2^-O,O-PhNO_2_)(IMes)(PPh_3_)]^+^ (V′-PhNO_2_) suggest that they do not represent the catalyst resting state in the corresponding reactions, although they are energetically accessible.

The free energy of TS1 – with respect to I – is somewhat similar for each of the substrates bearing an N-heterocyclic directing group (−4.0 to −8.2 kcal mol^−1^) and is slightly higher for the other substrates (−1.4 to 2.0 kcal mol^−1^). In all cases, the C–H activation step is endergonic overall, by 2.2 to 10.6 kcal mol^−1^. While not modelled here, the next steps would be exchange of the hydride ligand with a hydrogen from the dihydrogen (or D_2_) ligand (typically termed ‘hydrogen fluxionality’) and C–D bond formation. Barriers for C–H activation are broadly comparable for oxygen-based (19.6 to 26.9 kcal mol^−1^) and heterocyclic directing groups (20.1 to 26.8 kcal mol^−1^). As noted above, the replacement of ^1^H with ^2^H in key positions will change some of the barriers, but will do so by less than 1 kcal mol^−1^.

As an alternative to direct C–H activation, rotation of the bond between the phenyl group and the directing group can take place, *via*TS2, to present the alternative *ortho* C–H bond to the iridium catalyst. These transition states were located *via* relaxed potential energy scans of the X–C–C–C dihedral angle, for all substrates except *N*-phenylpyrazole, for which TS2 could not be located.¶¶The optimised structure of the corresponding [Ir(H)_2_(substrate)(IMes)(PPh_3_)]^+^ (VI) structure was used as a starting point in each case, with the X–C–C–C (X = O or N) dihedral angle stepped in increments of typically 10°. The results of this calculation were visualised using GaussView 6, and the lowest energy structure corresponding to an energy peak, with a corresponding minimum in the RMS gradient plot, was selected. This structure was then used to undertake a TS search; in some cases, it was necessary to limit the maximum step size in the optimisation process using the *maxstep* keyword. IRC calculations were performed using the *lqa* and *nogradstop* keywords. This workflow did not yield a TS2 structure for phenylpyrazole; despite repeated relaxed potential energy scans (rotating the phenyl group in both directions), frequency calculations of any promising structures led to zero imaginary frequencies and were therefore not suitable guess structures for transition state optimisation. Intrinsic reaction coordinate (IRC) calculations confirm that this stationary point corresponds to the rotation of the phenyl group. The energy of TS2 is invariably lower than that for TS1 for each substrate, but ΔΔ*G* varies between 5.1 kcal mol^−1^ (oxazoline) and 15.2 kcal mol^−1^ (acetophenone).

At this stage, an analysis of these energies reveals two trends that might explain the observed experimental behaviour with respect to isotopologue distribution as a function of time; key data are summarised in Table 2. The energy difference between the complexes with one (VI) and two (V) molecules of coordinated substrate vary considerably, but the two substrates for which the most rapid *d*_2_-isotopologue formation is observed, 2-phenylpyridine and 2-phenylpyrimidine, show the largest VI*vs.*V energy difference, in favour of the single substrate complex, VI. An associative pathway for substrate exchange in these systems is therefore less feasible here than for other substrates. In addition, and perhaps more importantly, TS2 is considerably lower in energy than intermediate V for 4 and 6, meaning that rotation around the phenyl-directing group bond will take place before associative substrate exchange *via*V.

**Table 2:** Selected key free energy differences as a function of substrate structure, at the M06-L/BS2(chloroform) level of theory

Substrate	Δ*G* (V *vs.* VI) (kcal mol^−1^)	Δ*G* (TS2 *vs.* V) (kcal mol^−1^)
Acetophenone (1)	+0.8	+6.9
Nitrobenzene (2)	+0.9	+11.2
Methyl benzoate (3)	+11.1	+8.3
**2-Phenylpyridine (4)**	**+19.1**	**−3.5**
1-Methyl-2-phenylimidazole (5)	+15.9	−0.9
**2-Phenylpyrimidine (6)**	**+18.2**	**−4.6**
1-Phenylpyrazole (7)	+15.0	
2-Phenyloxazoline (8)	+12.8	+8.9

## Conclusions

In summary, we have developed new insights into the binding behaviour of substrates in prototypical iridium-catalysed HIE reactions which are relevant to understanding selectivity for mono- *versus* difunctionalisation of aromatic substrates.

In our model HIE reactions, isotopologue distribution as a function of time can be assayed by sampling reactions for analysis by mass spectrometry. A broad range of substrates were assayed in this way in the HIE of aryl substrates where both C–H bonds *ortho* to the directing group can be exchanged for C–D bonds. While in most substrates there is an initial build-up of *d*_1_-isotopologue population before significant quantities of the *d*_2_-isotopologue are detected, 2-phenylpyridine and 2-phenylpyrimidine behave anomalously. Electronically modified pyridyl directing groups show different behaviour, implicating the binding of the directing group in the control of isotopologue distribution. Importantly, there is not a simple correlation between binding strength of the directing group and the isotopologue distribution during the reaction.

DFT studies provided further information on the relationship between structure and reactivity. [IrH_2_(IMes)(PPh_3_)(substrate)_2_]^+^ complexes (V) provide a route for the exchange of *d*_0_/*d*_1_/*d*_2_-substrate, while transition states (TS2) were obtained for rotation of the phenyl group on bound substrates, which can facilitate double *ortho*-C-H deuteration after a single binding event. For those substrates that lead to higher initial *d*_2_-isotopologue populations, the rotation around the C–C bond linking the phenyl group and the directing group is more favourable than the coordination of a second molecule of substrate.

**Scheme 3:**
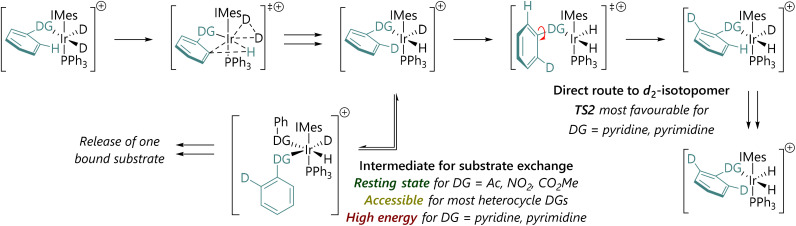
Summary of mechanistic hypothesis and rationale for why the *d*_2_-isotopologue is produced more quickly for 2-phenylpyridine and 2-phenylpyrimidine.

We therefore propose a mechanistic rationale for the quite different isotopologue distributions as a function of substrate (Scheme 3), and position our substates within three key types with different behaviours.

Type 1: For non-heterocyclic directing groups, TS2 for C–DG bond rotation is not competitive with intermediates such as V. Exchange of *d*_1_-substrate for *d*_0_-substrate can occur readily, and the *d*_2_-substrate is, ultimately, most likely formed *via* two separate, sequential labelling events.

Type 2: For some heterocyclic directing groups, such as those based on a five-membered heterocycle, TS2 and V are both energetically feasible. Experimentally, these systems build up significant populations of the *d*_1_-isotopologue, presumably through substrate exchange *via*V. These five-membered directing groups are typically quite small.

Type 3: For other heterocycle-directed substrates, such as 2-phenylpyridine and 2-phenylpyrimidine, HIE is directed by a six-membered directing group. TS2 is lower in energy than V and so most substrate molecules are doubly deuterated directly after one binding event. Slow substrate exchange, presumably resulting from steric effects, and a lower barrier to phenyl-directing group rotation, promote selectivity for the *d*_2_-isotopologue. Importantly, these results may also provide some explanation for the lack of correlation between rate and selectivity in iridium-catalysed HIE reactions.^11^ If substrate exchange is slow, as is the case for type 3 substrates, then this may account for slow turnover.^11^

These results also suggest ways in which control might be exerted over these processes. The rotation of an aryl group while the substrate is bound has a steric demand that is a factor of both the substitution pattern of the substrate, and the steric impact of the NHC and phosphine ligand. For example, recent studies have shown the promise of iridium complexes bearing bidentate *cis*-chelating ligands for hydrogen isotope exchange,^35–38^ and these have a very different steric profile from systems that bear monodentate NHC and phosphine ligands, such as Ir-1.

Finally, we note that type 3 substrates such as 2-phenylpyridine are ubiquitous across C–H activation chemistry under iridium, rhodium, ruthenium, palladium, and platinum catalysis (*inter alia*), yet behave anomalously in HIE reactions. This suggests that results obtained with such substrates cannot always be readily extrapolated to other systems.

Site-selectivity is an enduring challenge in C–H activation chemistry; over-reaction results in waste of often precious substrate and complicates the isolation and purification of products. This work provides insight into substrate binding and reactivity that we anticipate will be of interest and importance to researchers applying C–H activation across the broad field of chemical synthesis.

## Author contributions

DST: methodology, formal analysis, investigation, data curation. WJK: conceptualisation, resources, supervision, funding acquisition, writing – editing. DML: conceptualisation, writing – original draft, supervision. DJN: conceptualisation, resources, methodology, formal analysis, investigation, data curation, writing – original draft, visualisation, supervision, project administration, funding acquisition.

## Conflicts of interest

There are no conflicts to declare.

## Supplementary Material

SC-OLF-D5SC00759C-s001

SC-OLF-D5SC00759C-s002

## Data Availability

Data for this article, including synthetic methods, kinetic data, characterisation data, and DFT coordinates, are available as part of the ESI.† DFT data (coordinates, energies, *etc.*) can also be accessed *via* ioChem-BD (DOI: https://doi.org/10.19061/iochem-bd-6-541).
