# Supplementary material for: Probing substrate binding and release events in iridium-catalysed hydrogen isotope exchange reactions
Source: Chem Sci. 2025 Jul 1;16(29):13532–42. doi: 10.1039/d5sc00759c (PMC12209880; doi:10.1039/d5sc00759c)
Supplement: SC-016-D5SC00759C-s001 [file SC-016-D5SC00759C-s001.pdf]

This journal is © The Royal Society of Chemistry 2025

Active Catalyst Species

H_2

1	H	0.1625	-2.6462	0.0000
2	H	-0.5820	-2.6462	0.0000

$CHCl_3$

1	C	-0.0552	0.7257	-0.0059
2	H	-1.1409	0.7257	-0.0059
3	Cl	0.4778	-0.9634	-0.0059
4	Cl	0.4778	1.5702	1.4569
5	Cl	0.4778	1.5702	-1.4688

$Ir(H)_2(CHCl_3)_2(IMes)(PPh_3)$

1	C	-4.0513	-1.4811	0.2583
2	C	-3.1745	-1.5877	-0.8317
3	C	-3.3836	-0.8963	-2.0295
4	C	-4.4796	-0.0330	-2.0908
5	C	-5.3511	0.1380	-1.0188
6	C	-5.1243	-0.6031	0.1435
7	H	-5.8010	-0.4926	0.9873
8	C	-6.5011	1.0937	-1.1008
9	H	-6.6344	1.4809	-2.1121
10	H	-7.4380	0.6209	-0.7964
11	H	-6.3518	1.9493	-0.4343
12	C	-3.8420	-2.2790	1.5093
13	H	-4.3159	-1.7954	2.3659
14	H	-4.2778	-3.2800	1.4214
15	H	-2.7820	-2.4140	1.7412
16	C	-2.2196	-3.8588	-0.9213
17	C	-0.9948	-4.4101	-0.7892
18	H	-3.1815	-4.2927	-1.1375
19	H	-0.6545	-5.4296	-0.8630
20	C	-2.5045	-1.0624	-3.2325
21	H	-2.1273	-0.0944	-3.5765
22	H	-1.6442	-1.7055	-3.0456
23	H	-3.0712	-1.4925	-4.0637
24	H	-4.6556	0.5150	-3.0137
25	N	-2.0664	-2.4989	-0.7255
26	N	-0.1162	-3.3749	-0.5159
27	C	3.3945	-4.1304	-1.3853
28	C	3.1257	-4.3428	0.9877
29	H	4.0164	-4.2072	-2.2740
30	C	2.0729	-3.7134	-1.5263
31	C	1.5038	-3.3586	-2.8647
32	H	0.6387	-3.9789	-3.1209
33	H	1.1576	-2.3193	-2.8841
34	H	2.2459	-3.4821	-3.6537
35	C	1.2920	-3.6345	-0.3680
36	C	1.7891	-3.9572	0.8975
37	C	0.9047	-3.9395	2.1057
38	H	1.4846	-3.7844	3.0174
39	H	0.1452	-3.1560	2.0450
40	H	0.3711	-4.8899	2.2174
41	H	3.5348	-4.5863	1.9651
42	C	3.9400	-4.4456	-0.1392
43	C	5.3803	-4.8300	-0.0097
44	H	5.9997	-3.9444	0.1715
45	H	5.5453	-5.5106	0.8273
46	H	5.7569	-5.3056	-0.9168
47	Ir	-0.0394	-0.2366	-0.2460
48	H	1.0240	-0.6030	-1.3248
49	H	-0.9814	0.0655	-1.4395
50	C	-0.7611	-2.1729	-0.4591
51	P	0.5062	2.0694	-0.3575
52	C	0.4094	2.9137	1.2716

53	C	2.1597	2.5219	-0.9978
54	C	-0.6647	3.0226	-1.3943
55	C	1.4385	2.7488	2.2089
56	C	-0.7419	3.6084	1.6580
57	C	2.8715	3.6326	-0.5264
58	C	2.7254	1.7298	-2.0046
59	C	-0.2593	4.1846	-2.0568
60	C	-2.0048	2.6261	-1.4746
61	C	1.3192	3.2577	3.4968
62	H	2.3579	2.2429	1.9338
63	C	-0.8608	4.1150	2.9486
64	H	-1.5517	3.7536	0.9496
65	C	4.1323	3.9241	-1.0339
66	H	2.4421	4.2692	0.2409
67	C	3.9820	2.0311	-2.5147
68	H	2.1826	0.8662	-2.3775
69	C	-1.1757	4.9313	-2.7886
70	H	0.7768	4.5052	-2.0120
71	C	-2.9195	3.3815	-2.1987
72	H	-2.3406	1.7222	-0.9739
73	C	0.1633	3.9371	3.8725
74	H	2.1326	3.1258	4.2028
75	H	-1.7610	4.6499	3.2320
76	C	4.6913	3.1219	-2.0236
77	H	4.6777	4.7819	-0.6556
78	H	4.4117	1.4050	-3.2892
79	C	-2.5064	4.5341	-2.8585
80	H	-0.8469	5.8261	-3.3059
81	H	-3.9564	3.0639	-2.2468
82	H	0.0652	4.3311	4.8781
83	H	5.6778	3.3488	-2.4131
84	H	-3.2188	5.1203	-3.4288
85	Cl	1.7945	-0.7070	1.7459
86	C	3.3174	-0.0574	0.9715
87	H	3.0269	0.8772	0.4968
88	Cl	4.5148	0.2660	2.2115
89	Cl	-1.9832	0.4512	1.5473
90	C	-1.3224	0.5646	3.2402
91	H	-0.4513	1.2180	3.1870
92	Cl	-0.8096	-1.0294	3.7965
93	Cl	-2.5340	1.2693	4.2873
94	Cl	3.8928	-1.1389	-0.2769

Ir(H)$_2$(CHCl$_3$)(IMes)(PPh$_3$)

1	C	-4.0351	-2.0005	0.7135
2	C	-3.3565	-1.9242	-0.5085
3	C	-3.7835	-1.1025	-1.5592
4	C	-4.9211	-0.3253	-1.3471
5	C	-5.6229	-0.3580	-0.1416
6	C	-5.1685	-1.2044	0.8691
7	H	-5.7085	-1.2446	1.8118
8	C	-6.8039	0.5311	0.0888
9	H	-7.2263	0.8999	-0.8468
10	H	-7.5942	0.0197	0.6420
11	H	-6.5147	1.4047	0.6842
12	C	-3.5885	-2.9248	1.8051
13	H	-3.9017	-2.5555	2.7832
14	H	-4.0270	-3.9210	1.6826
15	H	-2.5038	-3.0552	1.8225
16	C	-2.2041	-4.0896	-1.0204
17	C	-0.9104	-4.4695	-1.1430
18	H	-3.1237	-4.6409	-1.1251
19	H	-0.4669	-5.4237	-1.3743
20	C	-3.0645	-1.0836	-2.8753
21	H	-2.0285	-0.7416	-2.7771
22	H	-3.0155	-2.0826	-3.3208

23	H	-3.5677	-0.4269	-3.5861
24	H	-5.2728	0.3172	-2.1509
25	N	-2.1915	-2.7408	-0.7065
26	N	-0.1384	-3.3468	-0.9017
27	C	3.2934	-2.9506	-2.2246
28	C	3.4109	-3.3261	0.1411
29	H	3.7865	-2.7597	-3.1748
30	C	1.9021	-3.0554	-2.2007
31	C	1.0851	-2.9018	-3.4459
32	H	0.6079	-3.8436	-3.7356
33	H	0.2800	-2.1721	-3.3119
34	H	1.7015	-2.5761	-4.2842
35	C	1.2972	-3.2988	-0.9662
36	C	2.0262	-3.4398	0.2196
37	C	1.3297	-3.6734	1.5244
38	H	2.0448	-3.8085	2.3359
39	H	0.6833	-2.8272	1.7845
40	H	0.6864	-4.5580	1.4923
41	H	3.9972	-3.4302	1.0506
42	C	4.0631	-3.0802	-1.0697
43	C	5.5552	-2.9626	-1.1160
44	H	5.9112	-2.7102	-2.1159
45	H	5.9156	-2.1955	-0.4245
46	H	6.0354	-3.9003	-0.8221
47	Ir	-0.2964	-0.3250	-0.3272
48	H	1.1056	-0.8797	0.0045
49	H	0.1905	-0.3489	-1.8125
50	C	-0.9188	-2.2642	-0.6264
51	P	0.6311	1.8322	-0.2706
52	C	0.3647	2.7706	1.2760
53	C	2.4443	1.8524	-0.4943
54	C	-0.0329	2.9155	-1.5815
55	C	0.9514	2.2828	2.4519
56	C	-0.4980	3.8680	1.3491
57	C	3.2415	2.8575	0.0644
58	C	3.0471	0.8576	-1.2725
59	C	0.5982	4.1102	-1.9405
60	C	-1.2187	2.5509	-2.2254
61	C	0.6757	2.8786	3.6760
62	H	1.6243	1.4297	2.4054
63	C	-0.7775	4.4589	2.5788
64	H	-0.9617	4.2557	0.4471
65	C	4.6142	2.8666	-0.1543
66	H	2.7916	3.6303	0.6812
67	C	4.4191	0.8737	-1.4907
68	H	2.4434	0.0640	-1.7028
69	C	0.0458	4.9254	-2.9203
70	H	1.5276	4.4001	-1.4582
71	C	-1.7718	3.3686	-3.2040
72	H	-1.7087	1.6149	-1.9642
73	C	-0.1963	3.9644	3.7423
74	H	1.1391	2.4963	4.5792
75	H	-1.4483	5.3101	2.6254
76	C	5.2047	1.8765	-0.9324
77	H	5.2233	3.6488	0.2861
78	H	4.8736	0.0962	-2.0963
79	C	-1.1398	4.5575	-3.5506
80	H	0.5433	5.8489	-3.1961
81	H	-2.6915	3.0744	-3.6991
82	H	-0.4149	4.4274	4.6985
83	H	6.2763	1.8860	-1.1006
84	H	-1.5665	5.1956	-4.3169
85	Cl	-2.9137	1.4870	0.7307
86	C	-2.3195	1.0654	2.3440
87	Cl	-3.6149	0.7291	3.4678
88	H	-1.7184	1.8925	2.7188

89	Cl	-1.1953	-0.3633	2.2257

Ir(H)$_2$(CHCl$_3$)(H$_2$)(IMes)(PPh$_3$)

1	C	2.0925	1.7528	2.8252
2	C	2.3274	2.2307	1.5312
3	C	3.5600	2.0880	0.8861
4	C	4.5636	1.3971	1.5634
5	C	4.3661	0.8707	2.8391
6	C	3.1295	1.0672	3.4549
7	H	2.9682	0.6839	4.4594
8	C	5.4457	0.0784	3.5070
9	H	5.2709	-0.0273	4.5786
10	H	5.5003	-0.9303	3.0821
11	H	6.4284	0.5335	3.3657
12	C	0.7873	1.9988	3.5180
13	H	0.7637	1.5124	4.4939
14	H	0.6166	3.0685	3.6795
15	H	-0.0611	1.6302	2.9337
16	C	1.1935	4.3405	0.8819
17	C	0.1101	4.6758	0.1451
18	H	1.9107	4.9433	1.4137
19	H	-0.3222	5.6320	-0.0984
20	C	3.8242	2.6943	-0.4590
21	H	4.5768	2.1232	-1.0060
22	H	2.9262	2.7508	-1.0785
23	H	4.2067	3.7160	-0.3584
24	H	5.5279	1.2692	1.0779
25	N	1.2803	2.9589	0.8641
26	N	-0.4384	3.4911	-0.3111
27	C	-2.6408	3.1678	-3.2628
28	C	-3.9887	3.1077	-1.2797
29	H	-2.5552	3.1227	-4.3456
30	C	-1.4871	3.3463	-2.5054
31	C	-0.1344	3.4301	-3.1419
32	H	0.4030	4.3357	-2.8454
33	H	0.4993	2.5852	-2.8445
34	H	-0.2077	3.4245	-4.2294
35	C	-1.6261	3.3946	-1.1133
36	C	-2.8636	3.2898	-0.4738
37	C	-2.9814	3.3797	1.0163
38	H	-2.8695	4.4131	1.3609
39	H	-3.9564	3.0287	1.3563
40	H	-2.2107	2.7947	1.5260
41	H	-4.9624	3.0191	-0.8039
42	C	-3.8995	3.0382	-2.6689
43	C	-5.1180	2.8262	-3.5128
44	H	-5.2476	3.6369	-4.2346
45	H	-5.0429	1.9005	-4.0912
46	H	-6.0245	2.7711	-2.9086
47	Ir	-0.2379	0.4092	-0.1340
48	H	0.3930	0.2023	1.2985
49	H	-1.5459	0.7600	0.6476
50	C	0.2773	2.4114	0.1211
51	P	-1.0500	-1.8059	-0.1191
52	C	-2.6159	-1.9676	-1.0556
53	C	0.0592	-3.0263	-0.9116
54	C	-1.4140	-2.5281	1.5181
55	C	-3.5564	-0.9329	-0.9832
56	C	-2.9130	-3.1006	-1.8203
57	C	0.3682	-2.8589	-2.2688
58	C	0.6732	-4.0570	-0.1957
59	C	-2.3309	-3.5794	1.6318
60	C	-0.7351	-2.0897	2.6587
61	C	-4.7630	-1.0284	-1.6644
62	H	-3.3443	-0.0453	-0.3932
63	C	-4.1216	-3.1912	-2.5024

64	H	-2.1983	-3.9154	-1.8872
65	C	1.2738	-3.7047	-2.8955
66	H	-0.1059	-2.0618	-2.8371
67	C	1.5893	-4.8973	-0.8240
68	H	0.4457	-4.1988	0.8563
69	C	-2.5607	-4.1795	2.8632
70	H	-2.8724	-3.9270	0.7572
71	C	-0.9652	-2.6968	3.8882
72	H	-0.0289	-1.2691	2.5845
73	C	-5.0471	-2.1565	-2.4275
74	H	-5.4816	-0.2176	-1.5990
75	H	-4.3387	-4.0742	-3.0938
76	C	1.8926	-4.7225	-2.1703
77	H	1.5000	-3.5702	-3.9479
78	H	2.0629	-5.6926	-0.2585
79	C	-1.8771	-3.7410	3.9927
80	H	-3.2776	-4.9897	2.9407
81	H	-0.4342	-2.3480	4.7677
82	H	-5.9880	-2.2296	-2.9622
83	H	2.6043	-5.3803	-2.6574
84	H	-2.0596	-4.2095	4.9538
85	Cl	2.1415	-0.1099	-1.3031
86	C	3.0436	-1.5013	-0.5110
87	H	2.6818	-2.3970	-1.0117
88	Cl	2.6749	-1.6091	1.1969
89	H	-0.7382	0.4796	-1.9210
90	H	-1.3355	0.8965	-1.5218
91	Cl	4.7565	-1.3131	-0.8274

CH_2Cl_2

1	C	-0.6859	0.0470	-0.0258
2	H	-0.3317	0.5479	0.8715
3	H	-0.3317	0.5479	-0.9231
4	Cl	-0.0399	-1.6030	-0.0258
5	Cl	-2.4568	0.1061	-0.0258

$Ir(H)_2(DCM)_2(IMes)(PPh_3)$

1	C	-4.1092	-1.5764	0.2127
2	C	-3.2422	-1.6709	-0.8852
3	C	-3.4504	-0.9556	-2.0678
4	C	-4.5425	-0.0854	-2.1084
5	C	-5.4096	0.0676	-1.0305
6	C	-5.1816	-0.6961	0.1174
7	H	-5.8546	-0.5984	0.9657
8	C	-6.5550	1.0306	-1.0897
9	H	-6.6946	1.4324	-2.0944
10	H	-7.4920	0.5589	-0.7836
11	H	-6.3949	1.8764	-0.4134
12	C	-3.8760	-2.3868	1.4501
13	H	-4.4544	-1.9983	2.2895
14	H	-4.1683	-3.4325	1.3071
15	H	-2.8211	-2.3915	1.7405
16	C	-2.2038	-3.9213	-1.0377
17	C	-0.9594	-4.4246	-0.8812
18	H	-3.1364	-4.3898	-1.3044
19	H	-0.5747	-5.4262	-0.9786
20	C	-2.5728	-1.1009	-3.2742
21	H	-2.2014	-0.1268	-3.6068
22	H	-1.7097	-1.7428	-3.0960
23	H	-3.1373	-1.5246	-4.1103
24	H	-4.7183	0.4832	-3.0189
25	N	-2.1190	-2.5638	-0.7861
26	N	-0.1404	-3.3625	-0.5369
27	C	3.5036	-3.5377	-1.1723
28	C	3.0956	-4.0068	1.1443
29	H	4.1951	-3.4249	-2.0037

30	C	2.1403	-3.3630	-1.4062
31	C	1.6185	-3.0561	-2.7762
32	H	1.0340	-3.8917	-3.1755
33	H	0.9567	-2.1851	-2.7756
34	H	2.4347	-2.8650	-3.4737
35	C	1.2720	-3.5089	-0.3159
36	C	1.7210	-3.8486	0.9632
37	C	0.7618	-4.0413	2.0968
38	H	1.2677	-3.9495	3.0590
39	H	-0.0528	-3.3126	2.0709
40	H	0.3026	-5.0350	2.0609
41	H	3.4675	-4.2475	2.1370
42	C	4.0015	-3.8587	0.0942
43	C	5.4704	-4.0473	0.3122
44	H	6.0561	-3.2915	-0.2162
45	H	5.7302	-3.9983	1.3705
46	H	5.7990	-5.0220	-0.0617
47	Ir	-0.1548	-0.2568	-0.2173
48	H	0.9948	-0.6615	-1.1849
49	H	-0.9815	0.0338	-1.4999
50	C	-0.8433	-2.1937	-0.4608
51	P	0.5025	2.0077	-0.3554
52	C	0.5377	2.8723	1.2603
53	C	2.1514	2.3189	-1.0880
54	C	-0.6422	3.0344	-1.3499
55	C	1.5861	2.6362	2.1608
56	C	-0.5483	3.6480	1.6807
57	C	2.9958	3.3427	-0.6446
58	C	2.5672	1.5119	-2.1550
59	C	-0.2164	4.2503	-1.8937
60	C	-1.9765	2.6465	-1.5107
61	C	1.5489	3.1611	3.4468
62	H	2.4499	2.0535	1.8538
63	C	-0.5851	4.1685	2.9710
64	H	-1.3686	3.8474	0.9977
65	C	4.2389	3.5354	-1.2386
66	H	2.6854	3.9923	0.1676
67	C	3.8047	1.7146	-2.7516
68	H	1.9150	0.7230	-2.5183
69	C	-1.1080	5.0589	-2.5879
70	H	0.8172	4.5645	-1.7821
71	C	-2.8673	3.4622	-2.1995
72	H	-2.3250	1.7022	-1.1024
73	C	0.4586	3.9239	3.8571
74	H	2.3740	2.9763	4.1264
75	H	-1.4296	4.7759	3.2796
76	C	4.6473	2.7205	-2.2880
77	H	4.8870	4.3280	-0.8805
78	H	4.1127	1.0848	-3.5795
79	C	-2.4347	4.6682	-2.7397
80	H	-0.7649	5.9963	-3.0121
81	H	-3.9008	3.1501	-2.3136
82	H	0.4299	4.3351	4.8604
83	H	5.6181	2.8718	-2.7475
84	H	-3.1283	5.3022	-3.2814
85	Cl	1.5045	-0.7467	1.9093
86	C	3.0241	-0.5917	0.9315
87	H	3.0025	-1.4081	0.2117
88	H	2.9878	0.3805	0.4420
89	Cl	4.4601	-0.7039	1.9291
90	Cl	-2.1303	0.5487	1.5246
91	C	-1.3683	0.6409	3.1556
92	H	-0.3958	1.1166	3.0358
93	H	-2.0405	1.2320	3.7728
94	Cl	-1.1604	-0.9508	3.8786

Ir(H)$_2$(DCM)(IMes)(PPh$_3$)

1	C	-3.9309	2.1633	0.3109
2	C	-3.4943	1.4300	-0.7946
3	C	-3.0660	2.0343	-1.9828
4	C	-3.0698	3.4249	-2.0337
5	C	-3.4888	4.2026	-0.9514
6	C	-3.9152	3.5545	0.2063
7	H	-4.2453	4.1462	1.0563
8	C	-3.5031	5.6966	-1.0483
9	H	-2.6147	6.0759	-1.5579
10	H	-4.3684	6.0422	-1.6228
11	H	-3.5589	6.1660	-0.0652
12	C	-4.4196	1.4868	1.5557
13	H	-5.4607	1.1640	1.4487
14	H	-3.8383	0.5932	1.7987
15	H	-4.3789	2.1616	2.4117
16	C	-4.5033	-0.8654	-0.8957
17	C	-4.0396	-2.1201	-0.6813
18	H	-5.4807	-0.5048	-1.1696
19	H	-4.5277	-3.0793	-0.7295
20	C	-2.6175	1.2095	-3.1495
21	H	-1.7804	0.5578	-2.8797
22	H	-3.4176	0.5572	-3.5143
23	H	-2.3015	1.8427	-3.9794
24	H	-2.7361	3.9155	-2.9446
25	N	-3.4384	-0.0026	-0.6935
26	N	-2.7018	-1.9918	-0.3513
27	C	-0.1774	-4.6771	-0.7247
28	C	-0.7142	-4.4262	1.5999
29	H	0.3849	-5.1687	-1.5148
30	C	-1.0775	-3.6693	-1.0727
31	C	-1.2932	-3.2822	-2.5024
32	H	-0.4677	-3.6208	-3.1295
33	H	-2.2099	-3.7315	-2.8993
34	H	-1.3936	-2.2007	-2.6235
35	C	-1.7857	-3.0528	-0.0353
36	C	-1.6285	-3.4180	1.3084
37	C	-2.4272	-2.7496	2.3847
38	H	-2.1134	-3.0876	3.3731
39	H	-2.3222	-1.6598	2.3484
40	H	-3.4963	-2.9626	2.2829
41	H	-0.5741	-4.7209	2.6372
42	C	0.0194	-5.0681	0.5991
43	C	0.9920	-6.1514	0.9509
44	H	1.4805	-6.5619	0.0658
45	H	1.7703	-5.7846	1.6272
46	H	0.4949	-6.9773	1.4670
47	Ir	-0.3755	-0.0608	-0.0761
48	H	-0.0933	-0.6113	-1.5122
49	H	-0.7382	1.2985	-0.7181
50	C	-2.3143	-0.6877	-0.3513
51	P	1.8877	0.5655	-0.0811
52	C	2.9714	-0.8672	-0.4048
53	C	2.3356	1.8055	-1.3464
54	C	2.5264	1.2890	1.4716
55	C	2.4398	-2.1553	-0.2915
56	C	4.3175	-0.7113	-0.7488
57	C	3.4050	2.6891	-1.1639
58	C	1.6146	1.8472	-2.5444
59	C	3.4146	0.6098	2.3098
60	C	2.0211	2.5328	1.8738
61	C	3.2428	-3.2693	-0.5098
62	H	1.3878	-2.2939	-0.0418
63	C	5.1177	-1.8255	-0.9665
64	H	4.7393	0.2845	-0.8544
65	C	3.7451	3.5938	-2.1629

66	H	3.9682	2.6791	-0.2352
67	C	1.9601	2.7511	-3.5412
68	H	0.7797	1.1695	-2.6918
69	C	3.7874	1.1644	3.5315
70	H	3.8106	-0.3566	2.0130
71	C	2.3997	3.0857	3.0901
72	H	1.3290	3.0687	1.2284
73	C	4.5821	-3.1047	-0.8453
74	H	2.8178	-4.2641	-0.4250
75	H	6.1603	-1.6961	-1.2363
76	C	3.0245	3.6261	-3.3518
77	H	4.5743	4.2762	-2.0101
78	H	1.3943	2.7741	-4.4667
79	C	3.2809	2.3993	3.9241
80	H	4.4803	0.6314	4.1739
81	H	2.0094	4.0530	3.3883
82	H	5.2088	-3.9728	-1.0198
83	H	3.2907	4.3348	-4.1287
84	H	3.5773	2.8313	4.8739
85	Cl	-0.9347	0.9136	2.4037
86	C	0.1778	0.0369	3.5213
87	H	1.0381	0.6811	3.6975
88	H	-0.3874	-0.1664	4.4277
89	Cl	0.7274	-1.4917	2.8306

$Ir(H)_2(DCM)(H_2)(IMes)(PPh_3)$

1	C	1.3222	3.8568	1.6805
2	C	1.0221	3.7169	0.3204
3	C	2.0011	3.7390	-0.6756
4	C	3.3278	3.8872	-0.2675
5	C	3.6780	4.0164	1.0755
6	C	2.6599	4.0064	2.0333
7	H	2.9161	4.1118	3.0845
8	C	5.1087	4.1625	1.4917
9	H	5.4091	3.3574	2.1687
10	H	5.7834	4.1503	0.6347
11	H	5.2717	5.1006	2.0293
12	C	0.2399	3.7912	2.7131
13	H	-0.2567	2.8129	2.7085
14	H	0.6373	3.9586	3.7141
15	H	-0.5435	4.5336	2.5336
16	C	-1.2485	4.4600	-0.4061
17	C	-2.4075	3.8308	-0.7050
18	H	-0.9794	5.5031	-0.4081
19	H	-3.3660	4.2092	-1.0190
20	C	1.6422	3.6184	-2.1243
21	H	0.9071	2.8282	-2.3013
22	H	1.2016	4.5471	-2.5022
23	H	2.5226	3.4069	-2.7320
24	H	4.1080	3.9026	-1.0246
25	N	-0.3434	3.4749	-0.0550
26	N	-2.1833	2.4749	-0.5347
27	C	-4.2563	-0.1517	-2.1236
28	C	-5.1102	0.2895	0.0730
29	H	-4.3171	-0.6862	-3.0683
30	C	-3.2625	0.8147	-1.9639
31	C	-2.3191	1.1494	-3.0781
32	H	-2.4827	0.4990	-3.9383
33	H	-2.4564	2.1822	-3.4149
34	H	-1.2732	1.0528	-2.7736
35	C	-3.2027	1.4805	-0.7341
36	C	-4.1257	1.2517	0.2935
37	C	-4.0846	2.0225	1.5779
38	H	-4.5211	1.4440	2.3940
39	H	-3.0677	2.2993	1.8652
40	H	-4.6591	2.9516	1.4974

41	H	-5.8304	0.0891	0.8623
42	C	-5.1873	-0.4298	-1.1210
43	C	-6.2489	-1.4678	-1.3132
44	H	-6.1443	-1.9837	-2.2682
45	H	-6.2182	-2.2177	-0.5177
46	H	-7.2468	-1.0213	-1.2843
47	Ir	0.0859	0.4143	0.1194
48	H	-0.9082	-0.1511	-0.9792
49	H	0.9741	0.8556	-1.0929
50	C	-0.9055	2.2325	-0.1233
51	P	1.3252	-1.5887	-0.0246
52	C	0.7608	-2.9598	1.0430
53	C	1.3681	-2.3134	-1.7007
54	C	3.0820	-1.3520	0.4222
55	C	0.3429	-4.1918	0.5301
56	C	0.6759	-2.7298	2.4225
57	C	0.2026	-2.3391	-2.4749
58	C	2.5308	-2.9064	-2.2012
59	C	3.8031	-2.3146	1.1345
60	C	3.7314	-0.1833	0.0062
61	C	-0.1622	-5.1690	1.3837
62	H	0.4105	-4.3900	-0.5350
63	C	0.1774	-3.7094	3.2699
64	H	1.0028	-1.7772	2.8328
65	C	0.2010	-2.9494	-3.7225
66	H	-0.7072	-1.8776	-2.1006
67	C	2.5270	-3.5075	-3.4554
68	H	3.4438	-2.8961	-1.6142
69	C	5.1457	-2.1053	1.4328
70	H	3.3169	-3.2300	1.4575
71	C	5.0729	0.0193	0.3027
72	H	3.1865	0.5723	-0.5541
73	C	-0.2496	-4.9288	2.7499
74	H	-0.4839	-6.1216	0.9769
75	H	0.1170	-3.5207	4.3363
76	C	1.3642	-3.5318	-4.2164
77	H	-0.7091	-2.9656	-4.3131
78	H	3.4369	-3.9582	-3.8366
79	C	5.7817	-0.9403	1.0195
80	H	5.6950	-2.8574	1.9889
81	H	5.5641	0.9293	-0.0269
82	H	-0.6470	-5.6909	3.4113
83	H	1.3642	-4.0011	-5.1943
84	H	6.8285	-0.7806	1.2545
85	Cl	-1.6845	-0.4121	1.9562
86	C	-2.5040	-1.7045	0.9773
87	H	-2.8570	-1.2146	0.0699
88	H	-1.7469	-2.4635	0.7862
89	Cl	-3.8469	-2.4137	1.8464
90	H	1.0743	0.8738	1.6030
91	H	1.4360	1.2912	0.9781

Acetophenone

PhAc

1	C	-1.8675	1.2346	0.0003
2	C	-0.4782	1.2060	-0.0002
3	C	0.2050	-0.0141	-0.0008
4	C	-0.5298	-1.2044	-0.0008
5	C	-1.9151	-1.1766	-0.0003
6	C	-2.5868	0.0443	0.0003
7	H	-2.3900	2.1854	0.0008
8	H	0.0738	2.1403	-0.0001
9	H	0.0206	-2.1393	-0.0013
10	H	-2.4772	-2.1047	-0.0004

11	H	-3.6719	0.0672	0.0006
12	C	1.6957	-0.1047	-0.0013
13	O	2.2529	-1.1851	-0.0018
14	C	2.4863	1.1798	-0.0013
15	H	2.2497	1.7875	-0.8793
16	H	2.2503	1.7870	0.8773
17	H	3.5495	0.9483	-0.0017

Ir(H)$_2$(CHCl$_3$)(PhAc)(IMes)(PPh$_3$)

1	C	-3.0392	-2.8467	1.4168
2	C	-2.1061	-3.2038	0.4407
3	C	-2.4529	-3.4010	-0.9010
4	C	-3.7821	-3.1951	-1.2584
5	C	-4.7486	-2.8188	-0.3224
6	C	-4.3601	-2.6606	1.0061
7	H	-5.1029	-2.3735	1.7466
8	C	-6.1590	-2.5627	-0.7531
9	H	-6.5976	-3.4405	-1.2355
10	H	-6.7968	-2.2903	0.0891
11	H	-6.2021	-1.7463	-1.4813
12	C	-2.6390	-2.6800	2.8506
13	H	-1.7347	-2.0730	2.9544
14	H	-3.4346	-2.2079	3.4288
15	H	-2.4236	-3.6450	3.3215
16	C	-0.2271	-4.6090	1.2823
17	C	1.0891	-4.4073	1.5190
18	H	-0.8470	-5.4834	1.3905
19	H	1.8593	-5.0694	1.8779
20	C	-1.4231	-3.8204	-1.9047
21	H	-0.9867	-4.7919	-1.6513
22	H	-1.8569	-3.9052	-2.9021
23	H	-0.5971	-3.1038	-1.9522
24	H	-4.0735	-3.3330	-2.2978
25	N	-0.7370	-3.4054	0.8262
26	N	1.3528	-3.0849	1.2068
27	C	4.7940	-2.1317	0.2545
28	C	4.2718	-1.0961	2.3502
29	H	5.4960	-2.3213	-0.5528
30	C	3.5417	-2.7362	0.2092
31	C	3.1578	-3.6368	-0.9252
32	H	3.1848	-4.6897	-0.6252
33	H	2.1431	-3.4375	-1.2814
34	H	3.8413	-3.5207	-1.7670
35	C	2.6588	-2.4889	1.2711
36	C	3.0142	-1.7069	2.3727
37	C	2.1239	-1.5325	3.5656
38	H	1.1189	-1.9223	3.4039
39	H	2.5477	-2.0482	4.4331
40	H	2.0319	-0.4781	3.8401
41	H	4.5578	-0.4651	3.1891
42	C	5.1686	-1.2856	1.3029
43	C	6.5048	-0.6087	1.2846
44	H	7.3194	-1.3349	1.2206
45	H	6.6055	0.0471	0.4126
46	H	6.6673	-0.0044	2.1783
47	Ir	-0.0129	-0.4228	0.4150
48	H	-1.1407	-0.4941	1.4722
49	H	0.8721	-0.2415	1.6830
50	C	0.2284	-2.4437	0.7668
51	P	-0.1712	1.9161	0.4509
52	C	-1.0245	2.7152	-0.9580
53	C	-1.0182	2.6125	1.9175
54	C	1.4744	2.7251	0.4490
55	C	-2.1928	3.4665	-0.8109
56	C	-0.4984	2.5162	-2.2405
57	C	-2.1335	1.9527	2.4440

58	C	-0.6256	3.8297	2.4819
59	C	1.6669	3.9999	-0.0944
60	C	2.5642	2.0665	1.0309
61	C	-2.8170	4.0160	-1.9267
62	H	-2.6182	3.6262	0.1753
63	C	-1.1184	3.0763	-3.3503
64	H	0.4073	1.9277	-2.3683
65	C	-2.8475	2.5027	3.5012
66	H	-2.4457	1.0022	2.0227
67	C	-1.3351	4.3726	3.5476
68	H	0.2408	4.3558	2.0943
69	C	2.9200	4.6011	-0.0540
70	H	0.8342	4.5240	-0.5550
71	C	3.8163	2.6709	1.0692
72	H	2.4339	1.0728	1.4512
73	C	-2.2811	3.8270	-3.1954
74	H	-3.7238	4.5984	-1.8003
75	H	-0.6879	2.9334	-4.3368
76	C	-2.4487	3.7141	4.0564
77	H	-3.7121	1.9800	3.8978
78	H	-1.0148	5.3142	3.9807
79	C	3.9968	3.9389	0.5270
80	H	3.0552	5.5902	-0.4787
81	H	4.6509	2.1467	1.5257
82	H	-2.7646	4.2667	-4.0617
83	H	-3.0008	4.1406	4.8870
84	H	4.9737	4.4097	0.5536
85	Cl	1.7959	-0.4935	-1.8016
86	C	3.4458	0.2561	-1.8170
87	H	3.7992	0.2398	-0.7856
88	Cl	3.3636	1.9196	-2.3799
89	C	-2.0016	-0.3300	-2.3797
90	C	-3.2502	0.2802	-1.8953
91	C	-4.1586	0.8859	-2.7741
92	C	-3.5411	0.2553	-0.5259
93	C	-5.3188	1.4727	-2.2877
94	H	-3.9519	0.9175	-3.8386
95	C	-4.7052	0.8303	-0.0435
96	H	-2.8498	-0.2525	0.1397
97	C	-5.5922	1.4480	-0.9230
98	H	-6.0109	1.9508	-2.9722
99	H	-4.9295	0.7921	1.0184
100	H	-6.5024	1.9029	-0.5458
101	C	-1.8183	-0.5621	-3.8493
102	H	-0.8756	-1.0763	-4.0296
103	H	-2.6437	-1.1485	-4.2630
104	H	-1.8072	0.3917	-4.3852
105	O	-1.0923	-0.6717	-1.6159
106	Cl	4.5257	-0.7066	-2.8204

Ir(H)$_2$(DCM)(PhAc)(IMes)(PPh$_3$)

1	C	1.2222	-3.0739	2.7531
2	C	0.0848	-3.2685	1.9661
3	C	-1.2116	-3.2511	2.4937
4	C	-1.3492	-2.9922	3.8541
5	C	-0.2436	-2.7690	4.6784
6	C	1.0281	-2.8243	4.1126
7	H	1.8983	-2.6598	4.7437
8	C	-0.4339	-2.4486	6.1281
9	H	-0.9749	-3.2442	6.6478
10	H	0.5189	-2.3031	6.6394
11	H	-1.0214	-1.5331	6.2513
12	C	2.5974	-3.1455	2.1638
13	H	3.3460	-2.7756	2.8659
14	H	2.8706	-4.1753	1.9099
15	H	2.6773	-2.5638	1.2409

16	C	0.4094	-4.7983	0.0285
17	C	0.4943	-4.6452	-1.3121
18	H	0.4484	-5.6762	0.6518
19	H	0.6259	-5.3617	-2.1056
20	C	-2.3986	-3.5069	1.6164
21	H	-2.3692	-4.5118	1.1829
22	H	-3.3297	-3.4193	2.1782
23	H	-2.4366	-2.8007	0.7809
24	H	-2.3483	-2.9636	4.2846
25	N	0.2437	-3.5318	0.5628
26	N	0.3814	-3.2890	-1.5668
27	C	-0.7918	-2.2221	-4.9072
28	C	1.4909	-1.5472	-4.6502
29	H	-1.6989	-2.2721	-5.5061
30	C	-0.7939	-2.7968	-3.6393
31	C	-2.0168	-3.4767	-3.1030
32	H	-2.9072	-3.1813	-3.6608
33	H	-1.9352	-4.5660	-3.1795
34	H	-2.1846	-3.2454	-2.0479
35	C	0.3872	-2.7231	-2.8871
36	C	1.5561	-2.1422	-3.3872
37	C	2.8582	-2.1786	-2.6463
38	H	3.5410	-2.8917	-3.1194
39	H	3.3550	-1.2053	-2.6684
40	H	2.7436	-2.4704	-1.6026
41	H	2.3850	-1.0711	-5.0469
42	C	0.3302	-1.5650	-5.4191
43	C	0.2648	-0.8658	-6.7416
44	H	-0.2725	-1.4562	-7.4868
45	H	-0.2686	0.0884	-6.6513
46	H	1.2582	-0.6456	-7.1352
47	Ir	0.2433	-0.5237	-0.1640
48	H	1.4253	-0.7733	0.8075
49	H	1.3875	-0.5421	-1.2198
50	C	0.2190	-2.5758	-0.4105
51	P	0.6813	1.7787	-0.0132
52	C	-0.5034	2.7742	0.9656
53	C	2.3077	2.2297	0.7021
54	C	0.6826	2.6218	-1.6437
55	C	-0.1312	3.4878	2.1075
56	C	-1.8464	2.7752	0.5684
57	C	2.9645	3.4017	0.3132
58	C	2.8732	1.4439	1.7109
59	C	0.2446	3.9416	-1.7971
60	C	1.1890	1.9449	-2.7596
61	C	-1.0846	4.1950	2.8335
62	H	0.9042	3.4930	2.4342
63	C	-2.7930	3.4913	1.2906
64	H	-2.1495	2.2215	-0.3176
65	C	4.1569	3.7769	0.9211
66	H	2.5469	4.0249	-0.4713
67	C	4.0608	1.8257	2.3238
68	H	2.3832	0.5256	2.0185
69	C	0.3069	4.5649	-3.0384
70	H	-0.1483	4.4857	-0.9434
71	C	1.2445	2.5692	-4.0005
72	H	1.5374	0.9207	-2.6559
73	C	-2.4139	4.2016	2.4268
74	H	-0.7835	4.7453	3.7190
75	H	-3.8271	3.5017	0.9599
76	C	4.7059	2.9928	1.9297
77	H	4.6580	4.6853	0.6041
78	H	4.4873	1.2050	3.1054
79	C	0.8029	3.8806	-4.1426
80	H	-0.0364	5.5888	-3.1424
81	H	1.6365	2.0282	-4.8570

82	H	-3.1527	4.7630	2.9894
83	H	5.6365	3.2873	2.4028
84	H	0.8446	4.3680	-5.1108
85	Cl	-2.0548	-0.2355	-1.7234
86	C	-1.8189	0.4359	-3.3779
87	H	-0.7563	0.3586	-3.6029
88	H	-2.4227	-0.1730	-4.0466
89	Cl	-2.3307	2.1127	-3.5000
90	C	-2.2318	-0.0044	2.1654
91	C	-1.5069	0.5216	3.3332
92	C	-2.1634	1.2425	4.3400
93	C	-0.1292	0.2980	3.4433
94	C	-1.4503	1.7461	5.4193
95	H	-3.2300	1.4286	4.2730
96	C	0.5788	0.7906	4.5270
97	H	0.3606	-0.2939	2.6761
98	C	-0.0794	1.5232	5.5130
99	H	-1.9626	2.3139	6.1884
100	H	1.6446	0.6000	4.6106
101	H	0.4750	1.9140	6.3601
102	C	-3.7311	-0.0105	2.1735
103	H	-4.1198	-0.5336	3.0520
104	H	-4.1145	1.0132	2.2183
105	H	-4.1053	-0.4857	1.2680
106	O	-1.6510	-0.4551	1.1726

Ir(H)$_2$(PhAc)$_2$(IMes)(PPh$_3$)

1	C	-3.9097	-2.2188	0.6508
2	C	-3.2740	-2.4643	-0.5702
3	C	-3.8205	-2.0786	-1.7953
4	C	-5.0469	-1.4145	-1.7722
5	C	-5.7151	-1.1392	-0.5797
6	C	-5.1304	-1.5493	0.6209
7	H	-5.6383	-1.3422	1.5603
8	C	-7.0363	-0.4328	-0.5796
9	H	-7.2890	-0.0469	-1.5685
10	H	-7.8439	-1.1054	-0.2756
11	H	-7.0454	0.4044	0.1240
12	C	-3.2731	-2.6418	1.9389
13	H	-2.2479	-2.2632	2.0191
14	H	-3.8423	-2.2856	2.7987
15	H	-3.2046	-3.7316	2.0191
16	C	-1.8581	-4.4948	-0.5321
17	C	-0.5359	-4.7539	-0.4343
18	H	-2.7125	-5.1483	-0.5907
19	H	0.0121	-5.6803	-0.3909
20	C	-3.0832	-2.3236	-3.0730
21	H	-2.1469	-1.7549	-3.0891
22	H	-2.8141	-3.3772	-3.1945
23	H	-3.6780	-2.0258	-3.9368
24	H	-5.4874	-1.0984	-2.7147
25	N	-1.9948	-3.1179	-0.5488
26	N	0.1061	-3.5271	-0.3894
27	C	3.6918	-3.3417	-1.2720
28	C	3.4661	-3.1567	1.1066
29	H	4.3192	-3.3719	-2.1603
30	C	2.3077	-3.4511	-1.4225
31	C	1.6739	-3.6013	-2.7714
32	H	1.2759	-4.6105	-2.9198
33	H	0.8325	-2.9121	-2.8996
34	H	2.3941	-3.4154	-3.5696
35	C	1.5298	-3.4023	-0.2668
36	C	2.0830	-3.2655	1.0116
37	C	1.2056	-3.2087	2.2214
38	H	0.5391	-4.0750	2.2830
39	H	1.7984	-3.1787	3.1371

40	H	0.5676	-2.3194	2.1951
41	H	3.9173	-3.0369	2.0891
42	C	4.2881	-3.1865	-0.0224
43	C	5.7711	-3.0370	0.1172
44	H	6.0232	-2.1410	0.6934
45	H	6.2094	-3.8878	0.6473
46	H	6.2646	-2.9620	-0.8533
47	Ir	-0.4743	-0.4446	-0.5097
48	H	-0.4255	-0.5443	-2.0803
49	H	-2.0155	-0.3923	-0.7011
50	C	-0.7798	-2.4891	-0.4587
51	P	-0.4278	1.8919	-0.7614
52	C	-0.0537	2.8318	0.7712
53	C	0.8526	2.4698	-1.9442
54	C	-1.9402	2.6881	-1.4189
55	C	1.0427	2.4155	1.5380
56	C	-0.8144	3.9173	1.2161
57	C	2.0446	3.0811	-1.5508
58	C	0.6328	2.2207	-3.3053
59	C	-1.8668	3.9709	-1.9781
60	C	-3.1845	2.0579	-1.3382
61	C	1.3901	3.0887	2.7020
62	H	1.6089	1.5414	1.2273
63	C	-0.4779	4.5739	2.3968
64	H	-1.6754	4.2508	0.6452
65	C	3.0003	3.4344	-2.5015
66	H	2.2317	3.2966	-0.5038
67	C	1.5802	2.5861	-4.2507
68	H	-0.2909	1.7434	-3.6229
69	C	-3.0117	4.6045	-2.4431
70	H	-0.9067	4.4743	-2.0522
71	C	-4.3299	2.6951	-1.8048
72	H	-3.2602	1.0577	-0.9251
73	C	0.6270	4.1677	3.1371
74	H	2.2471	2.7557	3.2797
75	H	-1.0791	5.4124	2.7328
76	C	2.7710	3.1908	-3.8503
77	H	3.9213	3.9115	-2.1818
78	H	1.3917	2.3994	-5.3027
79	C	-4.2464	3.9675	-2.3570
80	H	-2.9393	5.5963	-2.8765
81	H	-5.2872	2.1874	-1.7424
82	H	0.8880	4.6851	4.0545
83	H	3.5109	3.4774	-4.5901
84	H	-5.1390	4.4619	-2.7256
85	C	2.8189	-0.1830	-0.8150
86	C	-1.1238	0.3026	2.6069
87	O	-0.3927	-0.2660	1.7852
88	O	1.8164	-0.3761	-0.1168
89	C	-2.5072	0.7377	2.2418
90	H	-2.7930	0.2945	1.2881
91	H	-3.2336	0.4621	3.0102
92	H	-2.5402	1.8285	2.1439
93	C	-0.5983	0.5731	3.9563
94	C	-1.3162	1.3366	4.8848
95	C	0.6800	0.1073	4.2973
96	C	-0.7656	1.6324	6.1245
97	H	-2.3006	1.7170	4.6343
98	C	1.2213	0.3919	5.5389
99	H	1.2327	-0.4697	3.5632
100	C	0.5003	1.1591	6.4537
101	H	-1.3239	2.2308	6.8357
102	H	2.2071	0.0225	5.8009
103	H	0.9266	1.3861	7.4252
104	C	2.7743	-0.2795	-2.3059
105	H	1.7888	-0.6186	-2.6240

106	H	2.9736	0.6975	-2.7569
107	H	3.5414	-0.9630	-2.6766
108	C	4.0834	0.1828	-0.1419
109	C	5.2288	0.5341	-0.8656
110	C	4.1316	0.1955	1.2596
111	C	6.3945	0.8969	-0.2027
112	H	5.2079	0.5357	-1.9506
113	C	5.2963	0.5527	1.9189
114	H	3.2406	-0.0944	1.8091
115	C	6.4302	0.9069	1.1879
116	H	7.2764	1.1728	-0.7704
117	H	5.3283	0.5569	3.0036
118	H	7.3417	1.1894	1.7041

$Ir(H)_2(PhAc)(IMes)(PPh_3)$

1	C	-1.3649	3.8649	-1.3431
2	C	-0.9489	3.5660	-0.0405
3	C	-1.8424	3.4676	1.0298
4	C	-3.1981	3.6524	0.7604
5	C	-3.6639	3.9237	-0.5249
6	C	-2.7321	4.0286	-1.5601
7	H	-3.0780	4.2570	-2.5660
8	C	-5.1252	4.1112	-0.7914
9	H	-5.4190	3.6706	-1.7471
10	H	-5.7392	3.6635	-0.0081
11	H	-5.3840	5.1737	-0.8392
12	C	-0.3711	4.0048	-2.4547
13	H	-0.8705	4.1096	-3.4190
14	H	0.2594	4.8890	-2.3179
15	H	0.3017	3.1432	-2.5048
16	C	1.3325	4.4273	0.4290
17	C	2.5491	3.8729	0.6275
18	H	1.0048	5.4536	0.4239
19	H	3.5114	4.3148	0.8258
20	C	-1.3597	3.1148	2.4033
21	H	-0.5624	3.7831	2.7421
22	H	-2.1705	3.1608	3.1311
23	H	-0.9365	2.1016	2.4289
24	H	-3.9104	3.5632	1.5770
25	N	0.4519	3.3798	0.2097
26	N	2.3865	2.5031	0.5276
27	C	4.8671	0.2054	2.0301
28	C	5.1653	0.2259	-0.3504
29	H	5.1547	-0.1596	3.0130
30	C	3.8165	1.1170	1.9343
31	C	3.0765	1.5941	3.1451
32	H	1.9955	1.4668	3.0268
33	H	3.3874	1.0499	4.0371
34	H	3.2494	2.6591	3.3316
35	C	3.4701	1.5672	0.6586
36	C	4.1227	1.1372	-0.5008
37	C	3.6870	1.6199	-1.8498
38	H	4.3310	1.2259	-2.6362
39	H	2.6585	1.3084	-2.0669
40	H	3.7044	2.7123	-1.9170
41	H	5.6853	-0.1256	-1.2379
42	C	5.5573	-0.2444	0.9039
43	C	6.7139	-1.1867	1.0385
44	H	6.6260	-1.8143	1.9278
45	H	6.8108	-1.8362	0.1658
46	H	7.6559	-0.6364	1.1302
47	Ir	0.3555	0.2467	0.0723
48	H	1.8186	-0.1229	-0.2546
49	H	0.9042	-0.0282	1.5222
50	C	1.0874	2.1711	0.2677
51	P	-0.1389	-2.0472	0.0281

52	C	-1.3204	-2.5528	-1.2723
53	C	1.3347	-3.0939	-0.2586
54	C	-0.8442	-2.7356	1.5680
55	C	-0.9508	-2.3557	-2.6096
56	C	-2.6048	-3.0215	-0.9854
57	C	1.2937	-4.2494	-1.0442
58	C	2.5258	-2.7523	0.3924
59	C	-0.9955	-4.1181	1.7230
60	C	-1.2454	-1.8870	2.6011
61	C	-1.8415	-2.6432	-3.6360
62	H	0.0431	-1.9814	-2.8443
63	C	-3.4964	-3.3016	-2.0165
64	H	-2.9133	-3.1673	0.0453
65	C	2.4274	-5.0435	-1.1821
66	H	0.3774	-4.5311	-1.5541
67	C	3.6518	-3.5539	0.2582
68	H	2.5717	-1.8572	1.0071
69	C	-1.5510	-4.6367	2.8848
70	H	-0.6728	-4.7909	0.9327
71	C	-1.8032	-2.4080	3.7644
72	H	-1.0992	-0.8152	2.4957
73	C	-3.1186	-3.1159	-3.3412
74	H	-1.5357	-2.5064	-4.6687
75	H	-4.4896	-3.6697	-1.7803
76	C	3.6063	-4.6986	-0.5314
77	H	2.3862	-5.9354	-1.7983
78	H	4.5669	-3.2811	0.7730
79	C	-1.9587	-3.7818	3.9057
80	H	-1.6615	-5.7098	2.9974
81	H	-2.1063	-1.7411	4.5650
82	H	-3.8119	-3.3463	-4.1434
83	H	4.4880	-5.3215	-0.6390
84	H	-2.3883	-4.1893	4.8145
85	C	-1.6980	0.7722	-2.3055
86	C	-2.7856	0.4675	-1.3630
87	C	-4.0590	0.1127	-1.8289
88	C	-2.5653	0.4911	0.0223
89	C	-5.0641	-0.2424	-0.9417
90	H	-4.2547	0.0843	-2.8953
91	C	-3.5703	0.1432	0.9115
92	H	-1.6511	0.9322	0.4415
93	C	-4.8196	-0.2356	0.4285
94	H	-6.0389	-0.5321	-1.3184
95	H	-3.3888	0.1891	1.9802
96	H	-5.6065	-0.5109	1.1226
97	O	-0.5101	0.7585	-1.9542
98	C	-2.0200	1.0866	-3.7314
99	H	-2.7924	1.8564	-3.8059
100	H	-1.1200	1.4092	-4.2513
101	H	-2.4066	0.1925	-4.2311

$Ir(H)_2(PhAc)(IMes)(PPh_3)$ C-C Rotation TS2 ***Imaginary Frequency: -27.2 cm^{-1}***

1	C	-1.3789	4.3586	-0.5635
2	C	-0.7801	3.6262	0.4711
3	C	-1.4786	3.2493	1.6235
4	C	-2.8131	3.6459	1.7278
5	C	-3.4425	4.3973	0.7371
6	C	-2.7114	4.7336	-0.4055
7	H	-3.1894	5.3116	-1.1936
8	C	-4.8791	4.7995	0.8661
9	H	-5.5074	4.2555	0.1522
10	H	-5.2675	4.6001	1.8657
11	H	-5.0191	5.8626	0.6563
12	C	-0.6022	4.7393	-1.7874
13	H	-1.2662	5.0689	-2.5878
14	H	0.0919	5.5618	-1.5882

15	H	0.0023	3.9049	-2.1572
16	C	1.6120	4.2953	0.5151
17	C	2.7943	3.6584	0.3562
18	H	1.3776	5.3255	0.7248
19	H	3.8082	4.0211	0.3867
20	C	-0.8176	2.4761	2.7239
21	H	0.1638	2.8886	2.9757
22	H	-1.4305	2.4794	3.6259
23	H	-0.6451	1.4288	2.4472
24	H	-3.3689	3.3736	2.6217
25	N	0.6208	3.3339	0.3707
26	N	2.5031	2.3273	0.1221
27	C	4.8859	-0.4378	0.7281
28	C	4.6382	-0.1253	-1.6366
29	H	5.3105	-0.9835	1.5670
30	C	4.0224	0.6250	0.9885
31	C	3.6692	1.0174	2.3893
32	H	2.5845	1.0747	2.5276
33	H	4.0646	0.2994	3.1086
34	H	4.0727	2.0014	2.6498
35	C	3.4773	1.2944	-0.1093
36	C	3.7672	0.9415	-1.4318
37	C	3.1123	1.6518	-2.5765
38	H	3.5138	1.3117	-3.5315
39	H	2.0296	1.4756	-2.5823
40	H	3.2530	2.7356	-2.5206
41	H	4.8743	-0.4200	-2.6562
42	C	5.2042	-0.8291	-0.5715
43	C	6.1460	-1.9656	-0.8231
44	H	6.2380	-2.6133	0.0504
45	H	5.8217	-2.5773	-1.6683
46	H	7.1490	-1.5985	-1.0637
47	Ir	0.2921	0.2390	-0.0152
48	H	1.5937	-0.2431	-0.6866
49	H	1.0136	-0.0615	1.3431
50	C	1.1564	2.1038	0.1281
51	P	-0.1991	-2.0357	0.1245
52	C	-0.6569	-2.9195	-1.4059
53	C	1.3112	-2.9092	0.6932
54	C	-1.4520	-2.4560	1.3784
55	C	-0.0911	-2.4730	-2.6063
56	C	-1.5518	-3.9910	-1.4289
57	C	2.0886	-3.6850	-0.1673
58	C	1.7502	-2.6981	2.0056
59	C	-1.4845	-3.6961	2.0260
60	C	-2.3888	-1.4794	1.7264
61	C	-0.4118	-3.0937	-3.8075
62	H	0.5939	-1.6270	-2.5946
63	C	-1.8802	-4.6018	-2.6350
64	H	-2.0084	-4.3392	-0.5072
65	C	3.2867	-4.2383	0.2775
66	H	1.7620	-3.8653	-1.1867
67	C	2.9392	-3.2598	2.4481
68	H	1.1547	-2.0928	2.6848
69	C	-2.4474	-3.9535	2.9938
70	H	-0.7446	-4.4536	1.7812
71	C	-3.3498	-1.7377	2.6968
72	H	-2.3714	-0.5117	1.2238
73	C	-1.3120	-4.1557	-3.8238
74	H	0.0354	-2.7451	-4.7326
75	H	-2.5809	-5.4302	-2.6456
76	C	3.7138	-4.0280	1.5820
77	H	3.8829	-4.8409	-0.4001
78	H	3.2634	-3.0978	3.4712
79	C	-3.3794	-2.9751	3.3303
80	H	-2.4666	-4.9164	3.4929

81	H	-4.0730	-0.9714	2.9577
82	H	-1.5698	-4.6349	-4.7621
83	H	4.6447	-4.4656	1.9278
84	H	-4.1248	-3.1773	4.0921
85	C	-1.8958	1.1426	-2.2850
86	C	-3.0089	0.4768	-1.5521
87	C	-3.8442	1.2250	-0.7214
88	C	-3.2476	-0.8879	-1.7331
89	C	-4.8872	0.6034	-0.0434
90	H	-3.6655	2.2875	-0.5974
91	C	-4.3106	-1.4955	-1.0762
92	H	-2.6206	-1.4617	-2.4064
93	C	-5.1255	-0.7548	-0.2257
94	H	-5.5209	1.1871	0.6175
95	H	-4.4982	-2.5534	-1.2282
96	H	-5.9471	-1.2370	0.2930
97	O	-0.7146	0.9569	-1.9962
98	C	-2.2732	2.0560	-3.4016
99	H	-2.9058	2.8599	-3.0080
100	H	-1.3965	2.4775	-3.8904
101	H	-2.8916	1.5216	-4.1288

$Ir(H)_2(PhAc)(IMes)(PPh_3)$ C-H Activation TS1 ***Imaginary Frequency: -649.3 cm^{-1}***

1	Ir	-0.0369	0.3319	-0.0637
2	H	0.9706	1.3517	0.7077
3	P	1.7758	-1.2041	0.0156
4	C	-1.9527	4.1731	-0.2510
5	C	-3.0618	3.4179	-0.0922
6	H	-1.8205	5.2361	-0.3642
7	H	-4.1056	3.6781	-0.0360
8	N	-0.8738	3.3065	-0.2495
9	N	-2.6359	2.1032	0.0002
10	C	-1.2803	2.0156	-0.0904
11	H	0.7452	0.9500	-1.2973
12	C	-1.8475	-1.9363	0.6767
13	C	-1.2676	-1.4149	-0.5162
14	C	-1.4499	-2.1637	-1.6861
15	C	-2.1837	-3.3451	-1.6939
16	C	-2.7681	-3.8302	-0.5252
17	C	-2.5915	-3.1269	0.6512
18	H	-0.8525	-0.0340	-1.3888
19	H	-1.0150	-1.8202	-2.6197
20	H	-2.3050	-3.8885	-2.6261
21	H	-3.3431	-4.7494	-0.5363
22	H	-3.0133	-3.5095	1.5764
23	C	-1.5608	-1.2610	1.9255
24	C	-2.0453	-1.7665	3.2415
25	H	-1.8279	-1.0392	4.0218
26	H	-3.1154	-1.9833	3.2228
27	H	-1.5255	-2.7003	3.4831
28	O	-0.8335	-0.2430	1.9126
29	C	-3.5365	0.9992	0.1910
30	C	-3.9787	0.2841	-0.9261
31	C	-3.9711	0.7158	1.4897
32	C	-4.8450	-0.7844	-0.7042
33	C	-4.8521	-0.3526	1.6529
34	C	-5.2902	-1.1207	0.5740
35	H	-5.1782	-1.3720	-1.5562
36	H	-5.2044	-0.5900	2.6543
37	C	0.4848	3.7576	-0.3967
38	C	1.0173	3.8681	-1.6837
39	C	1.2075	4.0803	0.7540
40	C	2.3301	4.3204	-1.7987
41	C	2.5164	4.5291	0.5840
42	C	3.0916	4.6634	-0.6799
43	H	2.7655	4.4161	-2.7902

44	H	3.1003	4.7834	1.4650
45	C	0.5982	3.9244	2.1121
46	H	1.2960	4.2240	2.8942
47	H	-0.3080	4.5286	2.2234
48	H	0.3088	2.8835	2.2962
49	C	4.4831	5.1949	-0.8400
50	H	5.0261	4.6780	-1.6349
51	H	4.4665	6.2557	-1.1097
52	H	5.0596	5.1090	0.0831
53	C	0.2009	3.5160	-2.8888
54	H	0.7893	3.6028	-3.8024
55	H	-0.1831	2.4923	-2.8285
56	H	-0.6705	4.1705	-2.9928
57	C	-3.5071	1.5314	2.6572
58	H	-3.8996	2.5524	2.6157
59	H	-3.8411	1.0937	3.5989
60	H	-2.4161	1.6124	2.6853
61	C	-3.5657	0.6658	-2.3139
62	H	-4.1000	1.5613	-2.6497
63	H	-2.4997	0.8988	-2.3840
64	H	-3.7880	-0.1326	-3.0231
65	C	-6.2259	-2.2712	0.7805
66	H	-6.0557	-3.0636	0.0483
67	H	-6.1248	-2.7007	1.7798
68	H	-7.2687	-1.9559	0.6732
69	C	1.5682	-2.4036	1.3716
70	C	1.9962	-2.0718	2.6624
71	C	0.8456	-3.5857	1.1770
72	C	1.7393	-2.9249	3.7282
73	H	2.5372	-1.1450	2.8311
74	C	0.5910	-4.4375	2.2466
75	H	0.4818	-3.8438	0.1859
76	C	1.0433	-4.1130	3.5215
77	H	2.0886	-2.6640	4.7215
78	H	0.0415	-5.3584	2.0786
79	H	0.8539	-4.7836	4.3532
80	C	4.4818	-1.2624	0.7154
81	C	3.6588	0.8911	0.0014
82	C	5.7555	-0.7300	0.8580
83	H	4.3120	-2.3112	0.9422
84	C	4.9392	1.4173	0.1395
85	H	2.8480	1.5321	-0.3294
86	C	5.9863	0.6121	0.5702
87	H	6.5692	-1.3639	1.1932
88	H	5.1122	2.4637	-0.0880
89	H	6.9825	1.0267	0.6815
90	C	2.0527	-2.1973	-1.4941
91	C	2.6699	-3.4516	-1.4459
92	C	1.7416	-1.6414	-2.7381
93	C	2.9448	-4.1435	-2.6191
94	H	2.9346	-3.8946	-0.4908
95	C	2.0254	-2.3324	-3.9099
96	H	1.2767	-0.6608	-2.7861
97	C	2.6215	-3.5872	-3.8522
98	H	3.4182	-5.1182	-2.5688
99	H	1.7812	-1.8892	-4.8696
100	H	2.8400	-4.1284	-4.7664
101	C	3.4190	-0.4529	0.2952

Ir(H)(H)$_2$(PhAc)(IMes)(PPh$_3$)

1	C	-4.3560	-0.0787	0.8520
2	C	-3.7007	-0.6122	-0.2596
3	C	-3.8853	-0.1184	-1.5562
4	C	-4.7567	0.9541	-1.7177
5	C	-5.4335	1.5240	-0.6371
6	C	-5.2130	1.0013	0.6359

7	H	-5.7237	1.4404	1.4896
8	C	-6.3700	2.6733	-0.8491
9	H	-5.9358	3.4295	-1.5090
10	H	-7.2991	2.3420	-1.3228
11	H	-6.6397	3.1564	0.0915
12	C	-4.1446	-0.6406	2.2228
13	H	-4.5390	0.0303	2.9867
14	H	-4.6467	-1.6063	2.3445
15	H	-3.0833	-0.8060	2.4311
16	C	-3.4018	-3.0457	-0.0014
17	C	-2.3602	-3.9043	0.0434
18	H	-4.4666	-3.2085	0.0058
19	H	-2.3198	-4.9788	0.1078
20	C	-3.1499	-0.7038	-2.7204
21	H	-2.0856	-0.4446	-2.6755
22	H	-3.2151	-1.7961	-2.7400
23	H	-3.5433	-0.3258	-3.6643
24	H	-4.9125	1.3554	-2.7162
25	N	-2.8661	-1.7702	-0.0836
26	N	-1.2111	-3.1373	-0.0124
27	C	1.9845	-4.5146	-1.2667
28	C	2.0881	-4.3776	1.1248
29	H	2.4479	-4.7675	-2.2171
30	C	0.6806	-4.0257	-1.2660
31	C	-0.0933	-3.8705	-2.5397
32	H	-0.8757	-4.6321	-2.6240
33	H	-0.5997	-2.9027	-2.6030
34	H	0.5553	-3.9727	-3.4102
35	C	0.1117	-3.7005	-0.0289
36	C	0.7851	-3.8815	1.1806
37	C	0.1318	-3.5575	2.4878
38	H	0.8744	-3.4007	3.2717
39	H	-0.4933	-2.6630	2.4211
40	H	-0.5198	-4.3741	2.8173
41	H	2.6341	-4.5168	2.0547
42	C	2.7067	-4.6924	-0.0839
43	C	4.1102	-5.2136	-0.1238
44	H	4.5473	-5.2779	0.8738
45	H	4.1497	-6.2126	-0.5667
46	H	4.7555	-4.5758	-0.7352
47	Ir	-0.0627	-0.2832	-0.1475
48	C	-1.5051	-1.8026	-0.0902
49	P	1.8019	1.2071	-0.1611
50	C	1.4636	2.8235	0.6134
51	C	3.2937	0.5767	0.6802
52	C	2.3533	1.6032	-1.8589
53	C	1.4616	2.9270	2.0105
54	C	1.0737	3.9241	-0.1528
55	C	4.2332	1.4502	1.2404
56	C	3.5529	-0.7981	0.6919
57	C	3.7025	1.7823	-2.1761
58	C	1.3872	1.7507	-2.8626
59	C	1.0779	4.1101	2.6251
60	H	1.7598	2.0776	2.6177
61	C	0.6942	5.1105	0.4691
62	H	1.0735	3.8610	-1.2371
63	C	5.3978	0.9547	1.8149
64	H	4.0540	2.5211	1.2283
65	C	4.7207	-1.2883	1.2637
66	H	2.8408	-1.4920	0.2554
67	C	4.0776	2.1055	-3.4755
68	H	4.4643	1.6608	-1.4128
69	C	1.7687	2.0827	-4.1572
70	H	0.3348	1.6188	-2.6236
71	C	0.6899	5.2043	1.8557
72	H	1.0794	4.1771	3.7080

73	H	0.4075	5.9648	-0.1359
74	C	5.6426	-0.4144	1.8297
75	H	6.1156	1.6414	2.2505
76	H	4.9059	-2.3575	1.2697
77	C	3.1138	2.2580	-4.4658
78	H	5.1277	2.2370	-3.7132
79	H	1.0136	2.2009	-4.9272
80	H	0.3949	6.1304	2.3376
81	H	6.5512	-0.7983	2.2811
82	H	3.4107	2.5101	-5.4781
83	C	-0.7972	0.6189	1.5214
84	C	-1.6195	1.7571	1.2963
85	C	-0.5310	0.2759	2.8526
86	C	-2.1560	2.4886	2.3681
87	C	-1.0665	1.0090	3.9069
88	H	0.1220	-0.5633	3.0728
89	C	-1.8870	2.1140	3.6717
90	H	-2.7802	3.3559	2.1722
91	H	-0.8404	0.7177	4.9288
92	H	-2.3000	2.6761	4.5018
93	C	-1.8250	2.1386	-0.0880
94	H	0.8206	-1.2761	-1.3597
95	H	0.3333	-0.7747	-1.8434
96	H	0.7833	-1.2809	0.7172
97	C	-2.5646	3.3785	-0.4672
98	H	-1.9762	4.2541	-0.1742
99	H	-2.7312	3.4048	-1.5430
100	H	-3.5225	3.4470	0.0516
101	O	-1.3316	1.4289	-0.9937

Nitrobenzene

$PhNO_2$

1	C	-1.8776	-1.2081	-0.0004
2	C	-0.4895	-1.2162	-0.0004
3	C	0.1795	-0.0000	0.0000
4	C	-0.4895	1.2162	0.0005
5	C	-1.8776	1.2081	0.0005
6	C	-2.5697	0.0000	0.0001
7	H	-2.4203	-2.1469	-0.0007
8	H	0.0835	-2.1346	-0.0008
9	H	0.0835	2.1346	0.0008
10	H	-2.4203	2.1469	0.0009
11	H	-3.6545	0.0000	0.0001
12	O	2.2219	1.0852	0.0004
13	O	2.2219	-1.0852	-0.0004
14	N	1.6591	-0.0000	0.0000

$Ir(H)_2(CHCl_3)(PhNO_2)(IMes)(PPh_3)$

1	C	2.3695	2.7427	2.0391
2	C	2.0322	2.8952	0.6908
3	C	2.9746	2.7834	-0.3372
4	C	4.2807	2.4539	0.0218
5	C	4.6598	2.2707	1.3520
6	C	3.6937	2.4290	2.3449
7	H	3.9730	2.2968	3.3883
8	C	6.0731	1.9172	1.6997
9	H	6.1804	1.6728	2.7580
10	H	6.4258	1.0624	1.1155
11	H	6.7546	2.7452	1.4834
12	C	1.3362	2.8922	3.1130
13	H	0.4886	2.2189	2.9457
14	H	1.7562	2.6738	4.0960
15	H	0.9274	3.9072	3.1465
16	C	0.2352	4.5621	0.3774

17	C	-1.0783	4.5285	0.0671
18	H	0.8982	5.3807	0.6031
19	H	-1.8111	5.3114	-0.0359
20	C	2.6120	3.0570	-1.7647
21	H	3.3460	2.6262	-2.4477
22	H	1.6275	2.6638	-2.0287
23	H	2.5843	4.1350	-1.9583
24	H	5.0247	2.3435	-0.7638
25	N	0.6776	3.2514	0.3676
26	N	-1.4111	3.1983	-0.1282
27	C	-4.4742	2.4976	-2.0804
28	C	-5.0078	2.4199	0.2551
29	H	-4.7863	2.4439	-3.1206
30	C	-3.1321	2.7443	-1.7959
31	C	-2.1295	2.9654	-2.8855
32	H	-2.5794	2.8237	-3.8685
33	H	-1.7194	3.9805	-2.8528
34	H	-1.2801	2.2819	-2.7956
35	C	-2.7598	2.8132	-0.4500
36	C	-3.6801	2.6726	0.5944
37	C	-3.2758	2.8448	2.0271
38	H	-3.3350	3.8965	2.3283
39	H	-3.9352	2.2822	2.6904
40	H	-2.2493	2.5200	2.2127
41	H	-5.7367	2.2953	1.0524
42	C	-5.4246	2.3304	-1.0722
43	C	-6.8482	2.0088	-1.4032
44	H	-7.5429	2.4754	-0.7021
45	H	-7.1142	2.3294	-2.4117
46	H	-7.0206	0.9279	-1.3492
47	Ir	-0.0879	0.3440	-0.2511
48	H	-0.9620	0.4992	-1.5462
49	H	1.1062	0.7701	-1.1406
50	C	-0.3335	2.3806	0.0575
51	P	0.4575	-1.8579	-0.9215
52	C	-0.9243	-2.8755	-1.5645
53	C	1.6995	-1.9769	-2.2622
54	C	1.1609	-2.8912	0.4226
55	C	-1.6467	-2.3727	-2.6531
56	C	-1.2736	-4.1218	-1.0330
57	C	2.7305	-1.0360	-2.3529
58	C	1.6761	-3.0475	-3.1628
59	C	2.3923	-3.5418	0.3080
60	C	0.4528	-2.9904	1.6269
61	C	-2.6938	-3.1018	-3.1994
62	H	-1.3868	-1.4022	-3.0684
63	C	-2.3405	-4.8379	-1.5693
64	H	-0.7115	-4.5395	-0.2041
65	C	3.7120	-1.1620	-3.3285
66	H	2.7722	-0.2122	-1.6495
67	C	2.6580	-3.1665	-4.1397
68	H	0.8880	-3.7920	-3.1050
69	C	2.9000	-4.2764	1.3755
70	H	2.9623	-3.4677	-0.6127
71	C	0.9564	-3.7344	2.6868
72	H	-0.4952	-2.4697	1.7405
73	C	-3.0502	-4.3309	-2.6521
74	H	-3.2441	-2.7024	-4.0448
75	H	-2.6068	-5.8006	-1.1460
76	C	3.6769	-2.2242	-4.2256
77	H	4.5064	-0.4247	-3.3886
78	H	2.6246	-3.9981	-4.8353
79	C	2.1853	-4.3758	2.5643
80	H	3.8575	-4.7765	1.2734
81	H	0.3935	-3.8046	3.6120
82	H	-3.8767	-4.8939	-3.0720

83	H	4.4405	-2.3174	-4.9904
84	H	2.5847	-4.9497	3.3940
85	Cl	-2.1610	-0.3083	1.3940
86	C	-3.3096	-1.3839	0.4614
87	H	-2.7214	-2.2502	0.1623
88	Cl	-4.6101	-1.8942	1.5218
89	C	2.7663	-0.6916	3.0403
90	C	4.0971	-1.0953	3.0826
91	C	1.9607	-0.6504	4.1747
92	C	4.6350	-1.4651	4.3056
93	H	4.6773	-1.1155	2.1688
94	C	2.5167	-1.0237	5.3888
95	H	0.9268	-0.3400	4.0914
96	C	3.8485	-1.4292	5.4551
97	H	5.6695	-1.7840	4.3631
98	H	1.9098	-1.0010	6.2867
99	H	4.2753	-1.7198	6.4089
100	O	1.0049	0.0316	1.7381
101	O	2.9065	-0.3593	0.7565
102	N	2.2114	-0.3134	1.7540
103	Cl	-3.8850	-0.5642	-0.9727

Ir(H)$_2$(DCM)(PhNO$_2$)(IMes)(PPh3)

1	C	-0.1088	2.8975	3.1077
2	C	0.3122	3.1328	1.7961
3	C	1.6562	3.3204	1.4580
4	C	2.6004	3.2058	2.4757
5	C	2.2322	2.9188	3.7904
6	C	0.8770	2.7794	4.0860
7	H	0.5732	2.5744	5.1098
8	C	3.2625	2.7642	4.8666
9	H	3.1766	1.7904	5.3601
10	H	4.2766	2.8542	4.4734
11	H	3.1410	3.5197	5.6480
12	C	-1.5595	2.8274	3.4753
13	H	-2.1890	2.4904	2.6488
14	H	-1.7196	2.1560	4.3211
15	H	-1.9295	3.8145	3.7737
16	C	-1.3217	4.4290	0.4616
17	C	-2.1263	4.1784	-0.5946
18	H	-1.1470	5.3250	1.0337
19	H	-2.8072	4.8092	-1.1415
20	C	2.0562	3.6447	0.0508
21	H	1.7546	4.6617	-0.2220
22	H	3.1384	3.5818	-0.0784
23	H	1.5872	2.9738	-0.6747
24	H	3.6533	3.3378	2.2341
25	N	-0.6692	3.2437	0.7533
26	N	-1.9473	2.8477	-0.9258
27	C	-2.7961	1.6951	-4.3386
28	C	-4.5378	1.0224	-2.8339
29	H	-2.3797	1.7347	-5.3425
30	C	-2.0829	2.2853	-3.2935
31	C	-0.7573	2.9425	-3.5235
32	H	0.0409	2.4271	-2.9777
33	H	-0.4949	2.9409	-4.5816
34	H	-0.7497	3.9811	-3.1781
35	C	-2.6450	2.2243	-2.0178
36	C	-3.8713	1.6025	-1.7593
37	C	-4.4222	1.5454	-0.3683
38	H	-5.3931	1.0500	-0.3477
39	H	-3.7472	0.9961	0.2988
40	H	-4.5458	2.5432	0.0643
41	H	-5.4905	0.5301	-2.6552
42	C	-4.0204	1.0623	-4.1309
43	C	-4.7745	0.4403	-5.2658

44	H	-5.7369	0.9364	-5.4209
45	H	-4.2194	0.5009	-6.2035
46	H	-4.9930	-0.6134	-5.0691
47	Ir	-0.4644	0.2472	-0.2473
48	H	-1.8923	0.0281	-0.8522
49	H	-0.0016	0.5808	-1.6963
50	C	-1.0429	2.2416	-0.0985
51	P	-0.0931	-1.9913	-0.8518
52	C	-0.7739	-3.2415	0.3040
53	C	-0.8498	-2.4745	-2.4442
54	C	1.6709	-2.4277	-1.0624
55	C	-2.0847	-3.0345	0.7536
56	C	-0.0819	-4.3783	0.7323
57	C	-0.8700	-3.8276	-2.8045
58	C	-1.3719	-1.5245	-3.3238
59	C	2.2440	-2.4832	-2.3363
60	C	2.5029	-2.5639	0.0556
61	C	-2.6804	-3.9319	1.6298
62	H	-2.6358	-2.1610	0.4130
63	C	-0.6794	-5.2717	1.6169
64	H	0.9218	-4.5759	0.3701
65	C	-1.3973	-4.2203	-4.0265
66	H	-0.4717	-4.5770	-2.1255
67	C	-1.8958	-1.9233	-4.5500
68	H	-1.3751	-0.4740	-3.0516
69	C	3.6126	-2.6775	-2.4850
70	H	1.6193	-2.3701	-3.2166
71	C	3.8689	-2.7665	-0.0947
72	H	2.0826	-2.5192	1.0581
73	C	-1.9739	-5.0477	2.0722
74	H	-3.6971	-3.7615	1.9677
75	H	-0.1325	-6.1502	1.9427
76	C	-1.9088	-3.2669	-4.9034
77	H	-1.4100	-5.2709	-4.2956
78	H	-2.2928	-1.1748	-5.2282
79	C	4.4278	-2.8202	-1.3678
80	H	4.0419	-2.7204	-3.4802
81	H	4.4977	-2.8739	0.7841
82	H	-2.4367	-5.7466	2.7606
83	H	-2.3186	-3.5749	-5.8596
84	H	5.4948	-2.9756	-1.4869
85	Cl	-1.2568	-0.2421	2.3177
86	C	-0.0553	-1.3955	2.9959
87	H	-0.5902	-2.3133	3.2326
88	H	0.6945	-1.5435	2.2216
89	Cl	0.7222	-0.7791	4.4513
90	C	3.8625	0.3975	0.9788
91	C	5.0823	0.5830	0.3369
92	C	3.7679	0.1141	2.3385
93	C	6.2444	0.4786	1.0871
94	H	5.1005	0.7932	-0.7252
95	C	4.9410	0.0028	3.0690
96	H	2.7975	0.0002	2.8054
97	C	6.1754	0.1855	2.4475
98	H	7.2065	0.6222	0.6090
99	H	4.8915	-0.2218	4.1288
100	H	7.0883	0.0998	3.0267
101	O	1.5635	0.3856	0.7985
102	O	2.7273	0.6768	-1.0197
103	N	2.6515	0.4952	0.1807

$Ir(H)_2(PhNO_2)_2(IMes)(PPh_3)$

1	C	-3.7105	2.4148	-0.5510
2	C	-2.9959	2.6491	0.6264
3	C	-3.4667	2.2666	1.8853
4	C	-4.6933	1.6073	1.9401

5	C	-5.4388	1.3394	0.7912
6	C	-4.9337	1.7552	-0.4417
7	H	-5.5037	1.5580	-1.3471
8	C	-6.7654	0.6491	0.8796
9	H	-6.8642	0.0766	1.8036
10	H	-7.5865	1.3729	0.8615
11	H	-6.9278	-0.0289	0.0377
12	C	-3.1608	2.8316	-1.8808
13	H	-3.7726	2.4463	-2.6980
14	H	-3.1252	3.9212	-1.9814
15	H	-2.1351	2.4727	-2.0196
16	C	-1.5062	4.6369	0.5104
17	C	-0.1758	4.8366	0.3722
18	H	-2.3274	5.3287	0.5978
19	H	0.4107	5.7383	0.3140
20	C	-2.6506	2.5134	3.1143
21	H	-2.3970	3.5717	3.2292
22	H	-3.1810	2.1961	4.0123
23	H	-1.7026	1.9661	3.0667
24	H	-5.0746	1.2920	2.9083
25	N	-1.7030	3.2683	0.5304
26	N	0.4107	3.5842	0.3094
27	C	3.9899	2.9779	1.0368
28	C	3.7021	3.2106	-1.3318
29	H	4.6314	2.7984	1.8962
30	C	2.6252	3.1779	1.2503
31	C	2.0384	3.1268	2.6262
32	H	1.4390	2.2195	2.7590
33	H	2.8196	3.1258	3.3869
34	H	1.3746	3.9740	2.8221
35	C	1.8225	3.3871	0.1275
36	C	2.3335	3.4053	-1.1760
37	C	1.4302	3.5439	-2.3604
38	H	0.8328	2.6340	-2.4895
39	H	0.7266	4.3747	-2.2555
40	H	2.0028	3.7016	-3.2750
41	H	4.1196	3.2108	-2.3362
42	C	4.5459	2.9918	-0.2410
43	C	6.0130	2.7757	-0.4501
44	H	6.5238	3.7188	-0.6684
45	H	6.4894	2.3446	0.4323
46	H	6.2011	2.1109	-1.2980
47	Ir	-0.2572	0.5432	0.4649
48	H	0.0176	0.6596	1.9869
49	H	-1.7513	0.4689	0.9157
50	C	-0.5214	2.5913	0.4024
51	P	-0.1659	-1.7831	0.8273
52	C	1.1138	-2.6515	-0.1557
53	C	0.1609	-2.3433	2.5396
54	C	-1.7459	-2.6360	0.4479
55	C	1.0193	-2.6284	-1.5524
56	C	2.2480	-3.2179	0.4317
57	C	0.8946	-1.5479	3.4234
58	C	-0.2632	-3.6113	2.9543
59	C	-2.8940	-2.1559	1.0924
60	C	-1.8502	-3.7576	-0.3768
61	C	2.0260	-3.1600	-2.3457
62	H	0.1319	-2.2144	-2.0195
63	C	3.2593	-3.7483	-0.3651
64	H	2.3485	-3.2358	1.5126
65	C	1.1956	-2.0122	4.6994
66	H	1.2431	-0.5721	3.1047
67	C	0.0379	-4.0696	4.2303
68	H	-0.8337	-4.2427	2.2794
69	C	-4.1147	-2.7940	0.9249
70	H	-2.8250	-1.2865	1.7412

71	C	-3.0813	-4.3835	-0.5573
72	H	-0.9694	-4.1601	-0.8669
73	C	3.1547	-3.7179	-1.7507
74	H	1.9312	-3.1320	-3.4270
75	H	4.1356	-4.1846	0.1036
76	C	0.7672	-3.2707	5.1054
77	H	1.7653	-1.3862	5.3780
78	H	-0.2996	-5.0523	4.5421
79	C	-4.2124	-3.9090	0.0956
80	H	-4.9921	-2.4197	1.4437
81	H	-3.1493	-5.2550	-1.2005
82	H	3.9495	-4.1260	-2.3662
83	H	0.9991	-3.6290	6.1027
84	H	-5.1667	-4.4090	-0.0357
85	C	-1.8734	-0.8296	-3.0504
86	C	-2.0470	-1.7010	-4.1234
87	C	-2.8443	-0.6455	-2.0702
88	C	-3.2352	-2.4086	-4.2113
89	H	-1.2621	-1.8027	-4.8622
90	C	-4.0351	-1.3463	-2.1894
91	H	-2.6680	0.0289	-1.2388
92	C	-4.2283	-2.2273	-3.2499
93	H	-3.3913	-3.0964	-5.0342
94	H	-4.8060	-1.2112	-1.4392
95	H	-5.1585	-2.7805	-3.3271
96	O	-0.4134	0.6148	-1.9610
97	O	1.8348	0.5099	-0.4589
98	C	3.9693	-0.2903	-0.7364
99	C	5.1305	-0.7605	-0.1331
100	C	3.8444	-0.1484	-2.1159
101	C	6.2036	-1.1005	-0.9435
102	H	5.1707	-0.8540	0.9452
103	C	4.9291	-0.4929	-2.9089
104	H	2.9147	0.2136	-2.5398
105	C	6.1033	-0.9679	-2.3268
106	H	7.1199	-1.4681	-0.4959
107	H	4.8567	-0.3951	-3.9863
108	H	6.9457	-1.2358	-2.9555
109	O	0.1559	-0.1249	-3.9062
110	O	2.8909	-0.1750	1.3163
111	N	-0.6273	-0.0728	-2.9727
112	N	2.8379	0.0390	0.1154

$Ir(H)_2(PhNO_2)(IMes)(PPh_3)$

1	C	-3.6205	-1.8359	1.7361
2	C	-3.1111	-2.2022	0.4890
3	C	-3.7439	-1.8765	-0.7157
4	C	-4.9374	-1.1638	-0.6406
5	C	-5.4944	-0.7852	0.5824
6	C	-4.8190	-1.1224	1.7553
7	H	-5.2360	-0.8265	2.7149
8	C	-6.8017	-0.0556	0.6276
9	H	-6.9509	0.4537	1.5816
10	H	-6.8789	0.6835	-0.1735
11	H	-7.6396	-0.7482	0.4991
12	C	-2.8970	-2.1916	2.9976
13	H	-3.3600	-1.7195	3.8644
14	H	-2.8959	-3.2724	3.1728
15	H	-1.8482	-1.8792	2.9584
16	C	-1.7968	-4.3114	0.5041
17	C	-0.4912	-4.6393	0.3761
18	H	-2.6765	-4.9197	0.6323
19	H	0.0113	-5.5922	0.3741
20	C	-3.1339	-2.2538	-2.0301
21	H	-3.7879	-1.9850	-2.8596
22	H	-2.1738	-1.7464	-2.1809

23	H	-2.9335	-3.3278	-2.0945
24	H	-5.4462	-0.8970	-1.5634
25	N	-1.8729	-2.9321	0.4341
26	N	0.2019	-3.4494	0.2304
27	C	3.5333	-3.3553	-1.3779
28	C	3.7983	-3.0189	0.9815
29	H	3.9625	-3.4297	-2.3744
30	C	2.1532	-3.5023	-1.2328
31	C	1.2762	-3.8086	-2.4075
32	H	0.9757	-4.8617	-2.4075
33	H	0.3584	-3.2152	-2.4011
34	H	1.7957	-3.6179	-3.3468
35	C	1.6236	-3.3635	0.0550
36	C	2.4238	-3.1314	1.1795
37	C	1.8104	-2.9339	2.5320
38	H	1.1197	-3.7406	2.7940
39	H	2.5745	-2.8787	3.3081
40	H	1.2223	-2.0068	2.5689
41	H	4.4369	-2.8270	1.8405
42	C	4.3706	-3.1146	-0.2881
43	C	5.8492	-2.9711	-0.4736
44	H	6.3550	-3.9345	-0.3536
45	H	6.0963	-2.6022	-1.4709
46	H	6.2837	-2.2898	0.2612
47	Ir	-0.2413	-0.3454	0.1160
48	H	-1.7473	-0.2310	-0.2058
49	H	-0.8241	-0.1953	1.5658
50	C	-0.6395	-2.3745	0.2624
51	P	-0.1922	2.0052	0.0948
52	C	0.8499	2.7269	-1.2203
53	C	-1.8497	2.7303	-0.1727
54	C	0.3690	2.8080	1.6360
55	C	0.5535	2.3808	-2.5462
56	C	1.9686	3.5211	-0.9580
57	C	-2.0627	3.8337	-1.0031
58	C	-2.9276	2.1888	0.5375
59	C	0.2277	4.1898	1.8078
60	C	0.9403	2.0491	2.6591
61	C	1.3557	2.8292	-3.5870
62	H	-0.3103	1.7558	-2.7609
63	C	2.7667	3.9728	-2.0053
64	H	2.2194	3.7894	0.0636
65	C	-3.3363	4.3803	-1.1262
66	H	-1.2366	4.2674	-1.5585
67	C	-4.1935	2.7461	0.4197
68	H	-2.7755	1.3288	1.1847
69	C	0.6671	4.7988	2.9754
70	H	-0.2336	4.7886	1.0266
71	C	1.3801	2.6610	3.8285
72	H	1.0177	0.9712	2.5423
73	C	2.4646	3.6285	-3.3175
74	H	1.1146	2.5557	-4.6088
75	H	3.6286	4.5967	-1.7911
76	C	-4.4014	3.8400	-0.4151
77	H	-3.4930	5.2334	-1.7777
78	H	-5.0187	2.3229	0.9828
79	C	1.2471	4.0355	3.9855
80	H	0.5511	5.8698	3.1015
81	H	1.8156	2.0626	4.6221
82	H	3.0880	3.9838	-4.1313
83	H	-5.3920	4.2719	-0.5102
84	H	1.5844	4.5129	4.8992
85	C	2.8060	0.0522	-1.3591
86	C	3.9212	0.7061	-1.8724
87	C	2.5975	-0.0787	0.0124
88	C	4.8254	1.2743	-0.9891

89	H	4.0441	0.7763	-2.9458
90	C	3.5218	0.4819	0.8842
91	H	1.8491	-0.7700	0.4214
92	C	4.6234	1.1699	0.3856
93	H	5.6874	1.8069	-1.3745
94	H	3.3916	0.3565	1.9536
95	H	5.3368	1.6120	1.0725
96	O	0.6779	-0.7137	-1.9398
97	N	1.8548	-0.4978	-2.3146
98	O	2.2203	-0.7216	-3.4510

Ir(H)$_2$(PhNO$_2$)(IMes)(PPh$_3$) Rotation TS2 ***Imaginary Frequency: -49.8 cm^{-1}***

1	C	-3.7736	-1.4924	0.8893
2	C	-3.0647	-1.9834	-0.2091
3	C	-3.3756	-1.6356	-1.5290
4	C	-4.4418	-0.7629	-1.7293
5	C	-5.1803	-0.2446	-0.6634
6	C	-4.8308	-0.6206	0.6328
7	H	-5.3897	-0.2159	1.4728
8	C	-6.3348	0.6754	-0.9130
9	H	-6.5940	1.2489	-0.0214
10	H	-6.1217	1.3788	-1.7213
11	H	-7.2263	0.1132	-1.2090
12	C	-3.3969	-1.8710	2.2878
13	H	-3.9400	-1.2680	3.0165
14	H	-3.6213	-2.9220	2.4977
15	H	-2.3252	-1.7364	2.4660
16	C	-1.9416	-4.1920	0.1372
17	C	-0.6618	-4.5972	0.3012
18	H	-2.8642	-4.7462	0.0910
19	H	-0.2333	-5.5758	0.4387
20	C	-2.5517	-2.1438	-2.6719
21	H	-2.9763	-1.8391	-3.6288
22	H	-1.5247	-1.7625	-2.6223
23	H	-2.4783	-3.2357	-2.6712
24	H	-4.6994	-0.4765	-2.7460
25	N	-1.9127	-2.8150	0.0198
26	N	0.1229	-3.4531	0.2777
27	C	3.6824	-4.1002	-0.4795
28	C	3.5118	-3.2599	1.7606
29	H	4.2857	-4.4755	-1.3024
30	C	2.3073	-3.9694	-0.6640
31	C	1.6567	-4.3478	-1.9595
32	H	1.1456	-5.3133	-1.8864
33	H	0.9051	-3.6141	-2.2658
34	H	2.3943	-4.4281	-2.7579
35	C	1.5518	-3.4804	0.4113
36	C	2.1302	-3.1089	1.6305
37	C	1.2995	-2.5881	2.7637
38	H	0.4061	-3.1980	2.9268
39	H	1.8717	-2.5686	3.6917
40	H	0.9420	-1.5684	2.5747
41	H	3.9790	-2.9915	2.7049
42	C	4.3019	-3.7629	0.7270
43	C	5.7848	-3.8988	0.8830
44	H	6.1012	-3.7331	1.9136
45	H	6.1304	-4.8898	0.5795
46	H	6.3149	-3.1777	0.2515
47	Ir	-0.1631	-0.3271	0.0851
48	H	-1.5291	-0.1107	-0.5958
49	H	-0.9199	-0.2511	1.4530
50	C	-0.6372	-2.3368	0.1045
51	P	-0.1893	2.0029	0.2434
52	C	0.1516	2.9133	-1.3013
53	C	-1.8824	2.5212	0.7167
54	C	0.8712	2.7363	1.5269

55	C	-0.2913	2.3447	-2.5026
56	C	0.8700	4.1104	-1.3364
57	C	-2.7518	3.1458	-0.1780
58	C	-2.3390	2.1937	1.9990
59	C	0.5985	3.9810	2.1055
60	C	1.9847	2.0112	1.9559
61	C	-0.0177	2.9640	-3.7159
62	H	-0.8411	1.4055	-2.4844
63	C	1.1505	4.7226	-2.5541
64	H	1.2294	4.5567	-0.4141
65	C	-4.0586	3.4374	0.2049
66	H	-2.4132	3.4130	-1.1741
67	C	-3.6386	2.4953	2.3795
68	H	-1.6707	1.7052	2.7038
69	C	1.4387	4.4921	3.0864
70	H	-0.2789	4.5419	1.7943
71	C	2.8252	2.5247	2.9370
72	H	2.1934	1.0375	1.5132
73	C	0.7106	4.1509	-3.7435
74	H	-0.3663	2.5174	-4.6411
75	H	1.7138	5.6498	-2.5730
76	C	-4.5035	3.1145	1.4803
77	H	-4.7267	3.9253	-0.4973
78	H	-3.9785	2.2466	3.3798
79	C	2.5520	3.7655	3.5018
80	H	1.2221	5.4564	3.5333
81	H	3.6878	1.9529	3.2644
82	H	0.9329	4.6295	-4.6911
83	H	-5.5204	3.3494	1.7777
84	H	3.2016	4.1655	4.2730
85	C	3.0086	0.1298	-1.3925
86	C	3.0734	1.4883	-1.6616
87	C	3.7970	-0.4949	-0.4395
88	C	3.9760	2.2495	-0.9269
89	H	2.4609	1.9306	-2.4388
90	C	4.6832	0.2884	0.2915
91	H	3.7276	-1.5655	-0.2927
92	C	4.7760	1.6547	0.0443
93	H	4.0539	3.3131	-1.1239
94	H	5.3070	-0.1798	1.0464
95	H	5.4756	2.2607	0.6094
96	O	0.9033	-0.7295	-1.9649
97	N	2.1246	-0.7219	-2.2140
98	O	2.6282	-1.3847	-3.0921

$Ir(H)_2(PhNO_2)(IMes)(PPh_3)$ C-H Activation TS1 ***Imaginary Frequency:*** ***$-572.6\ cm^{-1}$***

1	Ir	0.2724	-0.2466	0.1184
2	H	1.2888	0.3961	-0.9812
3	P	-0.6038	1.9779	0.1630
4	C	3.2324	-3.2428	-0.7417
5	C	2.1806	-4.0581	-0.5046
6	H	4.2529	-3.4518	-1.0160
7	H	2.0860	-5.1308	-0.5243
8	N	2.7823	-1.9455	-0.5683
9	N	1.1092	-3.2417	-0.1867
10	C	1.4655	-1.9298	-0.2288
11	H	1.3679	0.3354	1.1006
12	C	-2.5410	-1.2307	0.1797
13	C	-1.5705	-0.7778	1.0920
14	C	-2.0169	-0.6360	2.4147
15	C	-3.3193	-0.9453	2.7841
16	C	-4.2437	-1.4150	1.8464
17	C	-3.8541	-1.5518	0.5305
18	H	0.0889	-1.0194	1.5105
19	H	-1.3283	-0.2769	3.1724
20	H	-3.6183	-0.8251	3.8207

21	H	-5.2567	-1.6589	2.1448
22	H	-4.5341	-1.8771	-0.2491
23	O	-1.0218	-0.9397	-1.5360
24	C	-0.1994	-3.7408	0.1403
25	C	-0.5741	-3.8505	1.4821
26	C	-1.0414	-4.1246	-0.9135
27	C	-1.8593	-4.3223	1.7535
28	C	-2.3114	-4.5891	-0.5843
29	C	-2.7410	-4.6913	0.7410
30	H	-2.1775	-4.3989	2.7907
31	H	-2.9882	-4.8669	-1.3885
32	C	3.6271	-0.7948	-0.7515
33	C	4.3260	-0.2949	0.3494
34	C	3.7215	-0.2382	-2.0298
35	C	5.1549	0.8056	0.1363
36	C	4.5639	0.8601	-2.1887
37	C	5.2963	1.3878	-1.1243
38	H	5.7138	1.2097	0.9766
39	H	4.6527	1.3126	-3.1730
40	C	2.9257	-0.7938	-3.1693
41	H	3.1702	-1.8429	-3.3640
42	H	1.8510	-0.7554	-2.9570
43	H	3.1077	-0.2348	-4.0871
44	C	6.2408	2.5309	-1.3374
45	H	7.2476	2.1654	-1.5637
46	H	5.9358	3.1586	-2.1773
47	H	6.3266	3.1599	-0.4485
48	C	4.1893	-0.9248	1.7011
49	H	3.1466	-0.9371	2.0351
50	H	4.5289	-1.9657	1.6991
51	H	4.7756	-0.3881	2.4471
52	C	-0.5955	-4.0352	-2.3409
53	H	0.1106	-4.8349	-2.5885
54	H	-1.4436	-4.1262	-3.0197
55	H	-0.0937	-3.0877	-2.5546
56	C	0.3356	-3.4774	2.6134
57	H	0.4237	-4.3017	3.3257
58	H	1.3418	-3.2137	2.2846
59	H	-0.0672	-2.6270	3.1756
60	C	-4.1124	-5.1994	1.0615
61	H	-4.8534	-4.8382	0.3441
62	H	-4.1448	-6.2928	1.0233
63	H	-4.4302	-4.9013	2.0622
64	C	0.5219	3.2579	-0.4902
65	C	-0.0068	4.4863	-0.9054
66	C	1.9068	3.0774	-0.4758
67	C	0.8373	5.5085	-1.3168
68	H	-1.0819	4.6416	-0.9172
69	C	2.7471	4.1098	-0.8789
70	H	2.3342	2.1317	-0.1568
71	C	2.2164	5.3221	-1.3024
72	H	0.4175	6.4524	-1.6469
73	H	3.8212	3.9599	-0.8611
74	H	2.8747	6.1234	-1.6207
75	C	-2.0299	3.6268	1.9519
76	C	-0.3124	2.2393	2.9220
77	C	-2.2837	4.1790	3.2015
78	H	-2.5994	3.9649	1.0917
79	C	-0.5632	2.8002	4.1684
80	H	0.4623	1.4856	2.8117
81	C	-1.5537	3.7658	4.3110
82	H	-3.0504	4.9389	3.3064
83	H	0.0169	2.4839	5.0288
84	H	-1.7522	4.2014	5.2842
85	C	-2.1003	2.0685	-0.8650
86	C	-1.9690	2.2298	-2.2495

87	C	-3.3616	1.7936	-0.3254
88	C	-3.0833	2.1423	-3.0728
89	H	-0.9907	2.4193	-2.6823
90	C	-4.4741	1.7078	-1.1549
91	H	-3.4749	1.6408	0.7448
92	C	-4.3375	1.8834	-2.5273
93	H	-2.9718	2.2723	-4.1436
94	H	-5.4485	1.5003	-0.7248
95	H	-5.2060	1.8138	-3.1731
96	C	-1.0495	2.6401	1.8044
97	N	-2.2001	-1.2958	-1.2136
98	O	-2.9924	-1.6636	-2.0537

Ir(H)(H_2)($PhNO_2$)(IMes)(PPh_3)

1	C	-3.7793	-2.1136	0.8451
2	C	-2.9711	-2.2702	-0.2832
3	C	-3.3757	-1.8790	-1.5649
4	C	-4.6273	-1.2850	-1.6889
5	C	-5.4618	-1.0833	-0.5872
6	C	-5.0229	-1.5082	0.6653
7	H	-5.6615	-1.3612	1.5329
8	C	-6.7713	-0.3788	-0.7464
9	H	-7.2778	-0.6691	-1.6691
10	H	-7.4444	-0.5762	0.0892
11	H	-6.6159	0.7043	-0.7958
12	C	-3.3261	-2.5715	2.1965
13	H	-3.3777	-3.6613	2.2920
14	H	-2.2886	-2.2857	2.3947
15	H	-3.9503	-2.1470	2.9837
16	C	-1.5779	-4.3035	-0.0777
17	C	-0.2563	-4.5795	-0.0204
18	H	-2.4444	-4.9434	-0.0911
19	H	0.2791	-5.5126	0.0341
20	C	-2.4811	-2.0653	-2.7503
21	H	-3.0127	-1.8573	-3.6790
22	H	-1.6224	-1.3853	-2.7028
23	H	-2.0826	-3.0830	-2.8091
24	H	-4.9559	-0.9584	-2.6719
25	N	-1.6975	-2.9250	-0.1374
26	N	0.4037	-3.3648	-0.0460
27	C	3.8928	-3.0499	-1.2335
28	C	3.8724	-2.8942	1.1595
29	H	4.4389	-3.0444	-2.1735
30	C	2.5138	-3.2434	-1.2607
31	C	1.7846	-3.4679	-2.5506
32	H	1.4953	-4.5179	-2.6643
33	H	0.8594	-2.8871	-2.6131
34	H	2.4082	-3.2089	-3.4067
35	C	1.8357	-3.2373	-0.0362
36	C	2.4903	-3.0811	1.1876
37	C	1.7341	-3.1140	2.4791
38	H	0.7854	-2.5744	2.4095
39	H	1.4897	-4.1415	2.7693
40	H	2.3192	-2.6787	3.2904
41	H	4.4009	-2.7618	2.1004
42	C	4.5892	-2.8680	-0.0360
43	C	6.0717	-2.6554	-0.0461
44	H	6.4662	-2.5027	0.9595
45	H	6.5922	-3.5145	-0.4781
46	H	6.3461	-1.7874	-0.6528
47	Ir	0.0861	-0.2991	-0.1607
48	C	-0.4783	-2.3225	-0.1179
49	P	1.0111	1.9089	-0.1845
50	C	-0.0992	3.1339	0.5813
51	C	2.6123	2.0860	0.6674
52	C	1.3068	2.5059	-1.8863

53	C	-0.1169	3.2744	1.9752
54	C	-1.0537	3.8052	-0.1864
55	C	3.0136	3.3188	1.1961
56	C	3.4966	1.0033	0.7185
57	C	2.4102	3.3019	-2.2068
58	C	0.3914	2.1664	-2.8913
59	C	-1.0688	4.0800	2.5837
60	H	0.6140	2.7501	2.5839
61	C	-2.0098	4.6059	0.4303
62	H	-1.0576	3.7012	-1.2674
63	C	4.2677	3.4572	1.7785
64	H	2.3431	4.1719	1.1530
65	C	4.7512	1.1481	1.2985
66	H	3.2080	0.0412	0.3059
67	C	2.5907	3.7533	-3.5093
68	H	3.1360	3.5639	-1.4438
69	C	0.5734	2.6289	-4.1890
70	H	-0.4738	1.5528	-2.6541
71	C	-2.0202	4.7436	1.8129
72	H	-1.0719	4.1848	3.6637
73	H	-2.7473	5.1213	-0.1754
74	C	5.1371	2.3726	1.8328
75	H	4.5659	4.4156	2.1894
76	H	5.4255	0.2987	1.3355
77	C	1.6739	3.4207	-4.5001
78	H	3.4524	4.3668	-3.7490
79	H	-0.1455	2.3677	-4.9582
80	H	-2.7657	5.3699	2.2911
81	H	6.1144	2.4833	2.2902
82	H	1.8182	3.7758	-5.5147
83	C	-1.0213	0.1616	1.4751
84	C	-2.2722	0.7574	1.2381
85	C	-0.6742	0.0148	2.8262
86	C	-3.1532	1.1725	2.2383
87	C	-1.5320	0.4214	3.8409
88	H	0.2918	-0.4080	3.0848
89	C	-2.7762	0.9943	3.5543
90	H	-4.0986	1.6228	1.9577
91	H	-1.2299	0.2939	4.8761
92	H	-3.4347	1.3049	4.3572
93	H	1.3574	-0.7677	-1.3348
94	H	0.7019	-0.5560	-1.8350
95	H	1.2938	-0.7730	0.7178
96	O	-1.8305	0.5911	-1.0263
97	N	-2.6470	0.9800	-0.1321
98	O	-3.6869	1.5273	-0.4311

Ir(H)$_2$(κ^2-PhNO$_2$)(IMes)(PPh$_3$)

1	C	-3.3640	-1.8636	2.1726
2	C	-3.1182	-2.2621	0.8553
3	C	-3.9887	-1.9737	-0.1973
4	C	-5.1419	-1.2487	0.1021
5	C	-5.4303	-0.8264	1.3993
6	C	-4.5296	-1.1427	2.4189
7	H	-4.7401	-0.8201	3.4355
8	C	-6.6857	-0.0677	1.7037
9	H	-6.4874	0.8038	2.3333
10	H	-7.1835	0.2741	0.7947
11	H	-7.3995	-0.6918	2.2499
12	C	-2.3973	-2.1924	3.2678
13	H	-2.7181	-1.7653	4.2180
14	H	-2.2945	-3.2735	3.4067
15	H	-1.3962	-1.8086	3.0437
16	C	-1.7941	-4.3564	0.6366
17	C	-0.5135	-4.6626	0.3288
18	H	-2.6344	-4.9790	0.8950

19	H	0.0019	-5.6064	0.2643
20	C	-3.6773	-2.4112	-1.5950
21	H	-4.4504	-2.0861	-2.2914
22	H	-2.7205	-2.0009	-1.9356
23	H	-3.5963	-3.5002	-1.6711
24	H	-5.8319	-1.0078	-0.7028
25	N	-1.9045	-2.9794	0.5715
26	N	0.1322	-3.4616	0.0828
27	C	3.2685	-3.4850	-1.8773
28	C	3.7913	-3.0332	0.4196
29	H	3.5897	-3.6218	-2.9074
30	C	1.9093	-3.5581	-1.5835
31	C	0.8874	-3.7952	-2.6509
32	H	0.3718	-4.7513	-2.5176
33	H	0.1156	-3.0186	-2.6395
34	H	1.3465	-3.8044	-3.6401
35	C	1.5243	-3.3651	-0.2521
36	C	2.4417	-3.0970	0.7671
37	C	1.9805	-2.8390	2.1672
38	H	1.3221	-3.6318	2.5351
39	H	2.8241	-2.7545	2.8526
40	H	1.4056	-1.9067	2.2213
41	H	4.5216	-2.8201	1.1964
42	C	4.2241	-3.2311	-0.8908
43	C	5.6795	-3.1765	-1.2393
44	H	6.1071	-4.1827	-1.2951
45	H	5.8423	-2.7091	-2.2137
46	H	6.2547	-2.6233	-0.4942
47	Ir	-0.3666	-0.3843	0.0128
48	H	-1.8298	-0.3379	-0.4933
49	H	-1.1487	-0.2141	1.3561
50	C	-0.7153	-2.4012	0.2269
51	P	-0.3202	1.9676	-0.0327
52	C	0.8838	2.6296	-1.2412
53	C	-1.9106	2.7558	-0.4517
54	C	0.1783	2.7314	1.5484
55	C	0.6282	2.4613	-2.6086
56	C	2.1070	3.1734	-0.8406
57	C	-1.9817	3.9456	-1.1831
58	C	-3.0904	2.1730	0.0254
59	C	-0.0726	4.0821	1.8089
60	C	0.8640	1.9660	2.4955
61	C	1.5675	2.8518	-3.5525
62	H	-0.3128	2.0222	-2.9308
63	C	3.0510	3.5551	-1.7907
64	H	2.3236	3.3020	0.2162
65	C	-3.2139	4.5375	-1.4382
66	H	-1.0745	4.4092	-1.5594
67	C	-4.3175	2.7719	-0.2287
68	H	-3.0485	1.2472	0.5928
69	C	0.3608	4.6564	2.9969
70	H	-0.6141	4.6841	1.0846
71	C	1.2987	2.5453	3.6826
72	H	1.0496	0.9129	2.3005
73	C	2.7837	3.3976	-3.1446
74	H	1.3519	2.7306	-4.6092
75	H	3.9958	3.9800	-1.4676
76	C	-4.3820	3.9524	-0.9623
77	H	-3.2596	5.4579	-2.0104
78	H	-5.2261	2.3096	0.1430
79	C	1.0482	3.8897	3.9334
80	H	0.1587	5.7037	3.1942
81	H	1.8278	1.9444	4.4148
82	H	3.5191	3.6987	-3.8835
83	H	-5.3418	4.4153	-1.1650
84	H	1.3827	4.3401	4.8619

85	O	2.0938	-0.2063	0.1003
86	O	1.1253	-0.5205	-1.7910
87	C	3.3986	-0.0274	-1.8205
88	C	3.4269	-0.1096	-3.2112
89	C	4.5245	0.2949	-1.0646
90	C	4.6262	0.1427	-3.8584
91	H	2.5244	-0.3648	-3.7524
92	C	5.7109	0.5501	-1.7321
93	H	4.4499	0.3432	0.0146
94	C	5.7629	0.4749	-3.1236
95	H	4.6744	0.0850	-4.9397
96	H	6.5999	0.8063	-1.1674
97	H	6.6965	0.6747	-3.6382
98	N	2.1622	-0.2735	-1.1406

Methyl Benzoate

$PhCO_2Me$

1	C	-2.1109	-1.3163	0.1691
2	C	-0.7384	-1.1247	0.0823
3	C	-0.2276	0.1520	-0.1643
4	C	-1.1013	1.2300	-0.3225
5	C	-2.4717	1.0346	-0.2351
6	C	-2.9778	-0.2390	0.0108
7	H	-2.5070	-2.3080	0.3606
8	H	-0.0545	-1.9562	0.2038
9	H	-0.6792	2.2108	-0.5131
10	H	-3.1480	1.8738	-0.3583
11	H	-4.0501	-0.3923	0.0793
12	C	1.2255	0.4228	-0.2683
13	O	1.7130	1.5082	-0.4801
14	O	1.9627	-0.6971	-0.0973
15	C	3.3736	-0.4952	-0.1873
16	H	3.8250	-1.4722	-0.0305
17	H	3.6456	-0.1012	-1.1685
18	H	3.7123	0.2094	0.5747

$Ir(H)_2(CHCl_3)(PhCO_2Me)(IMes)(PPh_3)$

1	C	-2.1208	-2.9220	-2.3071
2	C	-0.8183	-3.1907	-1.8699
3	C	0.2782	-3.1963	-2.7323
4	C	0.0418	-2.8863	-4.0730
5	C	-1.2323	-2.5918	-4.5510
6	C	-2.3039	-2.6314	-3.6545
7	H	-3.3068	-2.4102	-4.0138
8	C	-1.4545	-2.2195	-5.9837
9	H	-2.0133	-1.2821	-6.0636
10	H	-0.5118	-2.0949	-6.5189
11	H	-2.0384	-2.9810	-6.5092
12	C	-3.2610	-2.8898	-1.3371
13	H	-3.3399	-3.8137	-0.7565
14	H	-3.1343	-2.0768	-0.6115
15	H	-4.2127	-2.7381	-1.8487
16	C	-0.7900	-4.7129	0.0948
17	C	-0.6292	-4.5583	1.4289
18	H	-1.0074	-5.5815	-0.5043
19	H	-0.6773	-5.2649	2.2407
20	C	1.6561	-3.5342	-2.2516
21	H	2.4137	-3.1708	-2.9487
22	H	1.8641	-3.1124	-1.2647
23	H	1.7900	-4.6179	-2.1650
24	H	0.8858	-2.8522	-4.7580
25	N	-0.6252	-3.4611	-0.4725
26	N	-0.3666	-3.2186	1.6464
27	C	1.1991	-1.8419	4.7060

28	C	-1.1796	-1.5873	4.8640
29	H	2.1921	-1.7039	5.1270
30	C	1.0769	-2.4798	3.4730
31	C	2.2763	-2.9733	2.7244
32	H	2.2331	-4.0554	2.5656
33	H	2.3479	-2.5114	1.7341
34	H	3.1961	-2.7546	3.2675
35	C	-0.2114	-2.6330	2.9504
36	C	-1.3584	-2.2220	3.6356
37	C	-2.7322	-2.4960	3.1033
38	H	-2.7803	-2.4327	2.0141
39	H	-3.0594	-3.5053	3.3767
40	H	-3.4631	-1.7986	3.5151
41	H	-2.0573	-1.2519	5.4111
42	C	0.0862	-1.3804	5.4110
43	C	0.2388	-0.7126	6.7433
44	H	1.2437	-0.3098	6.8839
45	H	-0.4795	0.1015	6.8701
46	H	0.0601	-1.4196	7.5596
47	Ir	0.2371	-0.5393	0.2321
48	H	0.9039	-0.7153	1.6237
49	H	1.5575	-1.1532	-0.3022
50	C	-0.3629	-2.5124	0.4745
51	P	1.2681	1.5647	0.2087
52	C	0.7770	2.6925	-1.1416
53	C	0.8657	2.4892	1.7438
54	C	3.0949	1.5978	0.1715
55	C	-0.5900	2.9449	-1.3079
56	C	1.6848	3.2422	-2.0494
57	C	0.3426	3.7854	1.7442
58	C	1.0406	1.8267	2.9691
59	C	3.8221	0.5685	-0.4310
60	C	3.7818	2.7041	0.6846
61	C	-1.0408	3.7455	-2.3483
62	H	-1.3034	2.5026	-0.6155
63	C	1.2291	4.0330	-3.0999
64	H	2.7476	3.0462	-1.9456
65	C	-0.0287	4.3937	2.9417
66	H	0.2093	4.3204	0.8095
67	C	0.6777	2.4410	4.1603
68	H	1.4474	0.8196	2.9882
69	C	5.2074	0.6456	-0.5247
70	H	3.3043	-0.3003	-0.8255
71	C	5.1660	2.7763	0.5949
72	H	3.2308	3.5126	1.1569
73	C	-0.1290	4.2891	-3.2496
74	H	-2.1033	3.9399	-2.4594
75	H	1.9414	4.4490	-3.8047
76	C	0.1291	3.7225	4.1484
77	H	-0.4471	5.3945	2.9260
78	H	0.8146	1.9134	5.0985
79	C	5.8809	1.7484	-0.0118
80	H	5.7616	-0.1614	-0.9925
81	H	5.6873	3.6369	1.0001
82	H	-0.4784	4.9091	-4.0686
83	H	-0.1689	4.1963	5.0775
84	H	6.9620	1.8053	-0.0801
85	Cl	-2.2763	0.2972	1.0930
86	C	-2.4272	1.6999	2.2427
87	H	-1.4121	1.9849	2.5030
88	Cl	-3.2141	3.0536	1.4339
89	C	-0.9661	0.3141	-2.8772
90	C	0.2239	0.4953	-3.7232
91	C	0.1472	1.1445	-4.9592
92	C	1.4553	0.0124	-3.2711
93	C	1.2910	1.3016	-5.7307

94	H	-0.8065	1.5256	-5.3046
95	C	2.5956	0.1744	-4.0418
96	H	1.4942	-0.5001	-2.3152
97	C	2.5141	0.8183	-5.2749
98	H	1.2297	1.8048	-6.6895
99	H	3.5495	-0.2017	-3.6854
100	H	3.4051	0.9437	-5.8811
101	O	-0.9829	-0.1452	-1.7394
102	O	-2.0928	0.7204	-3.4690
103	C	-3.2761	0.6674	-2.6642
104	H	-3.4933	-0.3611	-2.3712
105	H	-3.1542	1.2753	-1.7642
106	H	-4.0732	1.0635	-3.2884
107	Cl	-3.2747	1.2187	3.7013

$Ir(H)_2(DCM)(PhCO_2Me)(IMes)(PPh_3)$

1	C	-1.6840	-2.5713	-3.1385
2	C	-0.3608	-2.8487	-2.7820
3	C	0.7107	-2.6611	-3.6599
4	C	0.4310	-2.1254	-4.9167
5	C	-0.8691	-1.7975	-5.3046
6	C	-1.9100	-2.0370	-4.4060
7	H	-2.9304	-1.8031	-4.6995
8	C	-1.1433	-1.2438	-6.6690
9	H	-1.2269	-2.0475	-7.4074
10	H	-2.0803	-0.6847	-6.6986
11	H	-0.3400	-0.5860	-7.0084
12	C	-2.8073	-2.8223	-2.1820
13	H	-3.7688	-2.5668	-2.6278
14	H	-2.8479	-3.8725	-1.8771
15	H	-2.6881	-2.2322	-1.2674
16	C	0.0552	-4.6777	-1.1513
17	C	0.3875	-4.7446	0.1572
18	H	-0.1001	-5.4469	-1.8895
19	H	0.5866	-5.5834	0.8034
20	C	2.1056	-3.0430	-3.2657
21	H	2.3967	-2.6070	-2.3046
22	H	2.2051	-4.1274	-3.1523
23	H	2.8299	-2.7343	-4.0215
24	H	1.2511	-1.9668	-5.6139
25	N	-0.0796	-3.3354	-1.4597
26	N	0.4497	-3.4407	0.6189
27	C	2.6575	-2.6117	3.4595
28	C	0.3840	-2.5531	4.2162
29	H	3.7205	-2.4930	3.6593
30	C	2.2488	-2.9600	2.1768
31	C	3.2303	-3.1262	1.0582
32	H	3.1256	-4.0916	0.5536
33	H	3.0808	-2.3569	0.2908
34	H	4.2577	-3.0504	1.4177
35	C	0.8749	-3.0954	1.9467
36	C	-0.0780	-2.9121	2.9487
37	C	-1.5397	-3.0953	2.6787
38	H	-1.7926	-4.1528	2.5476
39	H	-2.1412	-2.7159	3.5063
40	H	-1.8517	-2.5839	1.7628
41	H	-0.3420	-2.3736	5.0058
42	C	1.7396	-2.3892	4.4897
43	C	2.2103	-1.9594	5.8442
44	H	2.8366	-1.0645	5.7746
45	H	1.3754	-1.7329	6.5090
46	H	2.8180	-2.7335	6.3216
47	Ir	-0.1489	-0.4959	-0.2478
48	H	-0.9557	-0.6718	-1.5638
49	H	-1.5110	-0.8762	0.3930
50	C	0.1650	-2.5422	-0.3700

51	P	-0.8806	1.7371	-0.3002
52	C	-0.0908	2.8682	0.9010
53	C	-0.6090	2.5436	-1.9262
54	C	-2.6726	2.0109	-0.0234
55	C	1.3002	3.0264	0.8448
56	C	-0.8058	3.5047	1.9186
57	C	-0.1451	3.8559	-2.0529
58	C	-0.9682	1.8374	-3.0819
59	C	-3.2932	3.1581	-0.5313
60	C	-3.4230	1.1319	0.7616
61	C	1.9590	3.8067	1.7860
62	H	1.8659	2.5389	0.0532
63	C	-0.1412	4.2813	2.8626
64	H	-1.8831	3.3877	1.9840
65	C	-0.0362	4.4454	-3.3086
66	H	0.1251	4.4247	-1.1687
67	C	-0.8562	2.4292	-4.3338
68	H	-1.3484	0.8220	-2.9996
69	C	-4.6303	3.4170	-0.2577
70	H	-2.7292	3.8535	-1.1458
71	C	-4.7599	1.3955	1.0394
72	H	-2.9623	0.2328	1.1577
73	C	1.2388	4.4325	2.8004
74	H	3.0362	3.9279	1.7281
75	H	-0.7071	4.7660	3.6513
76	C	-0.3891	3.7353	-4.4498
77	H	0.3240	5.4653	-3.3922
78	H	-1.1411	1.8699	-5.2197
79	C	-5.3662	2.5380	0.5303
80	H	-5.0984	4.3080	-0.6622
81	H	-5.3299	0.7022	1.6494
82	H	1.7541	5.0392	3.5376
83	H	-0.3020	4.1977	-5.4271
84	H	-6.4105	2.7409	0.7423
85	Cl	2.3327	0.0649	-1.3095
86	C	2.1765	0.4100	-3.0771
87	H	2.8201	-0.3029	-3.5877
88	H	1.1254	0.2708	-3.3257
89	Cl	2.6752	2.0407	-3.4793
90	C	1.5091	0.4082	2.6420
91	C	0.4511	0.7538	3.6026
92	C	0.7388	1.4237	4.7958
93	C	-0.8689	0.4043	3.3058
94	C	-0.2866	1.7331	5.6792
95	H	1.7617	1.7017	5.0202
96	C	-1.8905	0.7186	4.1878
97	H	-1.0716	-0.1251	2.3800
98	C	-1.5997	1.3825	5.3777
99	H	-0.0627	2.2521	6.6048
100	H	-2.9145	0.4463	3.9516
101	H	-2.3975	1.6274	6.0711
102	O	1.3435	-0.1061	1.5393
103	O	2.7324	0.7144	3.0811
104	C	3.8047	0.4890	2.1603
105	H	3.6712	1.1021	1.2657
106	H	4.7101	0.7795	2.6873
107	H	3.8487	-0.5627	1.8719

$Ir(H)_2(PhCO_2Me)_2(IMes)(PPh_3)$

1	C	-3.1904	-3.1843	-0.0117
2	C	-2.1793	-3.1024	-0.9694
3	C	-2.4268	-2.7386	-2.2991
4	C	-3.7360	-2.4215	-2.6461
5	C	-4.7804	-2.4761	-1.7190
6	C	-4.4893	-2.8682	-0.4147
7	H	-5.2885	-2.9053	0.3216

8	C	-6.1768	-2.1189	-2.1271
9	H	-6.8505	-2.0762	-1.2697
10	H	-6.2094	-1.1483	-2.6317
11	H	-6.5854	-2.8507	-2.8303
12	C	-2.8823	-3.5568	1.4061
13	H	-3.7258	-3.3337	2.0614
14	H	-2.6626	-4.6249	1.5065
15	H	-2.0046	-3.0179	1.7777
16	C	-0.3598	-4.7189	-0.5624
17	C	0.9319	-4.6576	-0.1741
18	H	-0.9890	-5.5523	-0.8272
19	H	1.6742	-5.4243	-0.0262
20	C	-1.3118	-2.6629	-3.2940
21	H	-0.5821	-1.8980	-3.0059
22	H	-0.7623	-3.6070	-3.3626
23	H	-1.6871	-2.4209	-4.2892
24	H	-3.9482	-2.1272	-3.6716
25	N	-0.8326	-3.4188	-0.5815
26	N	1.2192	-3.3195	0.0383
27	C	4.7711	-2.3111	-0.1486
28	C	4.0900	-2.3399	2.1502
29	H	5.5305	-2.1280	-0.9052
30	C	3.4930	-2.6931	-0.5582
31	C	3.1585	-2.8850	-2.0055
32	H	2.2450	-2.3463	-2.2804
33	H	3.9685	-2.5327	-2.6453
34	H	2.9826	-3.9394	-2.2436
35	C	2.5270	-2.8781	0.4313
36	C	2.7984	-2.7121	1.7929
37	C	1.7109	-2.8798	2.8070
38	H	1.2465	-3.8698	2.7512
39	H	2.0938	-2.7512	3.8219
40	H	0.9122	-2.1477	2.6383
41	H	4.3264	-2.2046	3.2038
42	C	5.0875	-2.1249	1.1945
43	C	6.4525	-1.6770	1.6155
44	H	6.9491	-2.4309	2.2332
45	H	7.0943	-1.4735	0.7570
46	H	6.3963	-0.7635	2.2163
47	Ir	-0.0640	-0.4670	-0.1607
48	H	0.5220	-0.4403	-1.6094
49	H	-1.4177	-0.7518	-0.8511
50	C	0.1353	-2.5229	-0.2039
51	P	-0.4703	1.8380	-0.4938
52	C	-0.3573	3.0031	0.9168
53	C	0.7000	2.6154	-1.6856
54	C	-2.1280	2.1246	-1.2457
55	C	-0.0299	2.5177	2.1836
56	C	-0.5314	4.3841	0.7505
57	C	1.7904	3.3619	-1.2268
58	C	0.5712	2.3888	-3.0593
59	C	-2.5371	1.2687	-2.2790
60	C	-3.0087	3.1241	-0.8250
61	C	0.0621	3.3784	3.2742
62	H	0.1854	1.4613	2.2993
63	C	-0.4369	5.2428	1.8374
64	H	-0.7249	4.7898	-0.2384
65	C	2.7166	3.8855	-2.1218
66	H	1.9231	3.5391	-0.1636
67	C	1.4975	2.9148	-3.9516
68	H	-0.2653	1.8122	-3.4415
69	C	-3.7703	1.4351	-2.8956
70	H	-1.8917	0.4536	-2.5928
71	C	-4.2461	3.2846	-1.4407
72	H	-2.7536	3.7686	0.0075
73	C	-0.1535	4.7402	3.1052

74	H	0.3056	2.9836	4.2568
75	H	-0.5781	6.3089	1.6949
76	C	2.5713	3.6669	-3.4859
77	H	3.5567	4.4613	-1.7471
78	H	1.3739	2.7427	-5.0159
79	C	-4.6285	2.4495	-2.4825
80	H	-4.0614	0.7658	-3.6994
81	H	-4.9165	4.0639	-1.0927
82	H	-0.0850	5.4134	3.9530
83	H	3.2921	4.0789	-4.1843
84	H	-5.5920	2.5811	-2.9640
85	C	3.0849	0.6078	1.0146
86	C	3.8876	0.8600	-0.1947
87	C	3.3806	0.4750	-1.4377
88	C	5.1550	1.4489	-0.1167
89	C	4.1310	0.6627	-2.5873
90	H	2.3948	0.0255	-1.4839
91	C	5.9017	1.6389	-1.2712
92	H	5.5495	1.7493	0.8463
93	C	5.3939	1.2434	-2.5061
94	H	3.7275	0.3627	-3.5491
95	H	6.8838	2.0952	-1.2088
96	H	5.9823	1.3926	-3.4057
97	C	-1.7841	-0.0218	2.7457
98	C	-3.0391	0.5631	2.2549
99	C	-3.7262	1.5339	2.9952
100	C	-3.5719	0.1004	1.0487
101	C	-4.9311	2.0327	2.5239
102	H	-3.3086	1.8906	3.9294
103	C	-4.7930	0.5800	0.5999
104	H	-3.0283	-0.6430	0.4781
105	C	-5.4698	1.5491	1.3331
106	H	-5.4589	2.7921	3.0903
107	H	-5.2042	0.2090	-0.3332
108	H	-6.4184	1.9351	0.9736
109	O	-0.9402	-0.6116	2.0746
110	O	1.9520	0.1417	1.0603
111	O	3.7428	0.9478	2.1326
112	C	3.0652	0.7237	3.3681
113	H	2.2506	1.4420	3.4887
114	H	2.6684	-0.2930	3.4077
115	H	3.8106	0.8741	4.1460
116	O	-1.6301	0.1352	4.0634
117	C	-0.4314	-0.4035	4.6291
118	H	-0.4081	-0.0542	5.6584
119	H	-0.4566	-1.4948	4.6009
120	H	0.4442	-0.0561	4.0762

Ir(H)$_2$(PhCO$_2$Me)(IMes)(PPh$_3$)

1	C	-1.5484	3.8604	-0.7944
2	C	-0.9331	3.5421	0.4216
3	C	-1.6583	3.3050	1.5949
4	C	-3.0480	3.3866	1.5188
5	C	-3.7065	3.6867	0.3259
6	C	-2.9402	3.9234	-0.8155
7	H	-3.4398	4.1485	-1.7549
8	C	-5.2010	3.7459	0.2637
9	H	-5.5796	3.2863	-0.6528
10	H	-5.6615	3.2388	1.1135
11	H	-5.5566	4.7810	0.2710
12	C	-0.7392	4.1282	-2.0268
13	H	-1.3545	4.0417	-2.9246
14	H	-0.3225	5.1408	-2.0183
15	H	0.1056	3.4389	-2.1154
16	C	1.3841	4.4777	0.5981
17	C	2.6251	3.9386	0.5765

18	H	1.0487	5.4965	0.6987
19	H	3.6001	4.3910	0.6499
20	C	-0.9628	2.9574	2.8755
21	H	-0.2155	3.7092	3.1474
22	H	-1.6727	2.8785	3.6994
23	H	-0.4272	2.0050	2.7938
24	H	-3.6327	3.2017	2.4170
25	N	0.4953	3.4238	0.4614
26	N	2.4663	2.5717	0.4268
27	C	5.0886	0.1656	1.4350
28	C	4.9762	0.2364	-0.9599
29	H	5.5191	-0.2407	2.3471
30	C	4.0817	1.1255	1.5382
31	C	3.5612	1.5782	2.8667
32	H	2.4767	1.4389	2.9340
33	H	4.0257	1.0236	3.6823
34	H	3.7515	2.6421	3.0400
35	C	3.5457	1.6257	0.3503
36	C	3.9696	1.1960	-0.9122
37	C	3.3170	1.7147	-2.1565
38	H	3.8300	1.3534	-3.0486
39	H	2.2699	1.3921	-2.2104
40	H	3.3120	2.8088	-2.1906
41	H	5.3194	-0.1151	-1.9297
42	C	5.5479	-0.2910	0.2006
43	C	6.6247	-1.3277	0.1106
44	H	6.9292	-1.6822	1.0966
45	H	6.2952	-2.1927	-0.4723
46	H	7.5148	-0.9332	-0.3877
47	Ir	0.3726	0.3321	0.1814
48	H	1.8378	-0.1146	0.0129
49	H	0.7497	0.1016	1.6882
50	C	1.1474	2.2317	0.3518
51	P	-0.2000	-1.9402	0.1750
52	C	-1.1404	-2.5024	-1.2880
53	C	1.2733	-3.0288	0.2016
54	C	-1.1877	-2.5053	1.6038
55	C	-0.5469	-2.3319	-2.5464
56	C	-2.4437	-2.9978	-1.2134
57	C	1.3312	-4.2273	-0.5161
58	C	2.3622	-2.6599	1.0003
59	C	-1.3669	-3.8684	1.8630
60	C	-1.7846	-1.5670	2.4487
61	C	-1.2405	-2.6630	-3.7030
62	H	0.4670	-1.9418	-2.6122
63	C	-3.1348	-3.3291	-2.3753
64	H	-2.9240	-3.1230	-0.2478
65	C	2.4612	-5.0354	-0.4422
66	H	0.4959	-4.5301	-1.1404
67	C	3.4857	-3.4726	1.0745
68	H	2.3308	-1.7311	1.5630
69	C	-2.1402	-4.2811	2.9399
70	H	-0.8945	-4.6083	1.2222
71	C	-2.5606	-1.9818	3.5258
72	H	-1.6234	-0.5064	2.2675
73	C	-2.5377	-3.1638	-3.6192
74	H	-0.7672	-2.5386	-4.6720
75	H	-4.1460	-3.7168	-2.3046
76	C	3.5396	-4.6597	0.3507
77	H	2.4965	-5.9612	-1.0066
78	H	4.3228	-3.1740	1.6974
79	C	-2.7405	-3.3383	3.7704
80	H	-2.2711	-5.3399	3.1357
81	H	-3.0157	-1.2458	4.1808
82	H	-3.0779	-3.4271	-4.5224
83	H	4.4196	-5.2919	0.4057

84	H	-3.3402	-3.6632	4.6139
85	C	-1.3862	0.7409	-2.5128
86	C	-2.6493	0.4513	-1.8233
87	C	-3.7802	0.0464	-2.5403
88	C	-2.7181	0.5458	-0.4291
89	C	-4.9465	-0.2878	-1.8673
90	H	-3.7252	-0.0249	-3.6199
91	C	-3.8837	0.2093	0.2414
92	H	-1.8908	0.9808	0.1407
93	C	-4.9966	-0.2177	-0.4781
94	H	-5.8168	-0.6132	-2.4267
95	H	-3.9322	0.3015	1.3212
96	H	-5.9093	-0.4813	0.0461
97	O	-0.2815	0.8085	-1.9725
98	O	-1.5195	0.9255	-3.8216
99	C	-0.3002	1.1122	-4.5574
100	H	0.3682	0.2631	-4.4031
101	H	-0.5981	1.1847	-5.5997
102	H	0.2023	2.0265	-4.2377

Ir(H)$_2$(PhCO$_2$Me)(IMes)(PPh$_3$) C-C Rotation TS2 *Imaginary Frequency:* ***-61.9 cm^{-1}***

1	C	-2.4711	-2.5670	-1.8662
2	C	-1.1264	-2.7082	-2.2207
3	C	-0.6506	-2.4385	-3.5052
4	C	-1.5751	-2.0036	-4.4550
5	C	-2.9257	-1.8423	-4.1490
6	C	-3.3531	-2.1301	-2.8508
7	H	-4.4038	-2.0122	-2.5972
8	C	-3.9042	-1.3880	-5.1882
9	H	-3.4021	-1.0258	-6.0868
10	H	-4.5643	-2.2062	-5.4918
11	H	-4.5487	-0.5891	-4.8122
12	C	-2.9296	-2.8593	-0.4719
13	H	-4.0085	-2.7321	-0.3760
14	H	-2.6850	-3.8828	-0.1683
15	H	-2.4466	-2.1960	0.2539
16	C	0.2930	-4.3892	-1.0338
17	C	1.1553	-4.3501	0.0070
18	H	-0.0223	-5.2027	-1.6655
19	H	1.7579	-5.1200	0.4569
20	C	0.8005	-2.5909	-3.8405
21	H	1.1125	-3.6403	-3.8312
22	H	1.0192	-2.1934	-4.8318
23	H	1.4343	-2.0670	-3.1174
24	H	-1.2260	-1.7810	-5.4603
25	N	-0.1895	-3.1040	-1.2044
26	N	1.1760	-3.0388	0.4592
27	C	3.4268	-0.9524	2.5310
28	C	2.3585	-2.5627	3.9380
29	H	4.1127	-0.1183	2.3989
30	C	2.7973	-1.4829	1.4062
31	C	3.0831	-0.9083	0.0525
32	H	2.4357	-0.0474	-0.2159
33	H	4.0836	-0.4695	0.0195
34	H	3.0041	-1.6454	-0.7476
35	C	1.9171	-2.5565	1.5859
36	C	1.7058	-3.1336	2.8469
37	C	0.8178	-4.3264	3.0470
38	H	-0.0688	-4.3003	2.4082
39	H	1.3447	-5.2604	2.8267
40	H	0.4891	-4.3937	4.0860
41	H	2.1928	-2.9919	4.9235
42	C	3.2121	-1.4663	3.8075
43	C	3.9006	-0.8866	5.0044
44	H	4.3465	0.0849	4.7846
45	H	3.2122	-0.7634	5.8444

46	H	4.7049	-1.5417	5.3524
47	Ir	0.2530	-0.1876	-0.3354
48	H	0.3615	-0.3719	-1.8838
49	H	-1.2873	-0.1791	-0.5519
50	C	0.3504	-2.2451	-0.2949
51	P	0.3086	2.1197	-0.7229
52	C	-0.5277	2.7125	-2.2312
53	C	-0.3835	3.1932	0.5914
54	C	2.0450	2.6710	-0.8587
55	C	-1.1792	1.8165	-3.0827
56	C	-0.5415	4.0800	-2.5320
57	C	-1.6107	2.8128	1.1430
58	C	0.2350	4.3578	1.0539
59	C	2.6298	3.0429	-2.0699
60	C	2.8437	2.5937	0.2914
61	C	-1.8247	2.2825	-4.2240
62	H	-1.1849	0.7565	-2.8501
63	C	-1.1839	4.5391	-3.6733
64	H	-0.0483	4.7873	-1.8699
65	C	-2.2006	3.5650	2.1503
66	H	-2.0994	1.9186	0.7630
67	C	-0.3584	5.1129	2.0620
68	H	1.1824	4.6780	0.6316
69	C	3.9875	3.3466	-2.1268
70	H	2.0257	3.0966	-2.9704
71	C	4.1943	2.9066	0.2320
72	H	2.3982	2.2814	1.2352
73	C	-1.8257	3.6397	-4.5215
74	H	-2.3271	1.5785	-4.8795
75	H	-1.1881	5.5997	-3.9006
76	C	-1.5698	4.7154	2.6169
77	H	-3.1521	3.2578	2.5730
78	H	0.1287	6.0170	2.4120
79	C	4.7692	3.2839	-0.9797
80	H	4.4328	3.6370	-3.0723
81	H	4.8017	2.8577	1.1303
82	H	-2.3295	4.0014	-5.4116
83	H	-2.0268	5.3044	3.4048
84	H	5.8250	3.5276	-1.0274
85	C	-0.6107	0.1666	2.8596
86	C	-1.7911	-0.7392	2.8794
87	C	-3.0414	-0.3263	2.4207
88	C	-1.6318	-2.0211	3.4028
89	C	-4.1195	-1.2021	2.4727
90	H	-3.1794	0.6787	2.0399
91	C	-2.7110	-2.8958	3.4429
92	H	-0.6661	-2.3160	3.7974
93	C	-3.9559	-2.4877	2.9768
94	H	-5.0920	-0.8755	2.1202
95	H	-2.5807	-3.8916	3.8543
96	H	-4.8007	-3.1669	3.0167
97	O	0.2041	0.2659	1.9507
98	O	-0.4929	0.8598	3.9803
99	C	0.6170	1.7784	4.0401
100	H	0.5443	2.2542	5.0140
101	H	1.5587	1.2350	3.9404
102	H	0.5300	2.5177	3.2410

$Ir(H)_2(PhCO_2Me)(IMes)(PPh_3)$ C-H Activation TS1 ***Imaginary Frequency: -640.9 cm^{-1}***

1	Ir	-0.1530	-0.2952	-0.1709
2	H	-1.6121	-0.5589	0.4992
3	P	-0.6757	2.0146	0.0088
4	C	-0.8782	-4.4958	-0.7187
5	C	0.4530	-4.5680	-0.4987
6	H	-1.6109	-5.2557	-0.9330
7	H	1.1331	-5.4032	-0.4778

8	N	-1.2281	-3.1607	-0.6216
9	N	0.8885	-3.2727	-0.2749
10	C	-0.1418	-2.3848	-0.3422
11	H	-1.0849	-0.2494	-1.4454
12	C	2.6347	0.3655	0.7399
13	C	1.9229	0.3627	-0.4862
14	C	2.5912	0.8934	-1.5971
15	C	3.8924	1.3772	-1.5066
16	C	4.5813	1.3501	-0.2958
17	C	3.9453	0.8502	0.8260
18	H	0.7691	-0.3898	-1.4697
19	H	2.0914	0.9333	-2.5603
20	H	4.3729	1.7764	-2.3946
21	H	5.5963	1.7262	-0.2303
22	H	4.4398	0.8424	1.7924
23	C	1.9146	-0.0495	1.9317
24	O	0.7279	-0.4177	1.8908
25	C	2.2564	-2.9476	0.0231
26	C	3.1088	-2.5773	-1.0217
27	C	2.6895	-3.0606	1.3471
28	C	4.4255	-2.2640	-0.6948
29	C	4.0172	-2.7348	1.6203
30	C	4.8966	-2.3290	0.6171
31	H	5.0999	-1.9498	-1.4878
32	H	4.3699	-2.7948	2.6473
33	C	-2.5800	-2.7003	-0.7981
34	C	-3.0141	-2.3746	-2.0853
35	C	-3.4056	-2.6140	0.3253
36	C	-4.3321	-1.9454	-2.2293
37	C	-4.7155	-2.1809	0.1271
38	C	-5.1993	-1.8511	-1.1394
39	H	-4.6927	-1.6873	-3.2218
40	H	-5.3762	-2.1031	0.9869
41	C	-2.8846	-2.9511	1.6873
42	H	-2.0536	-2.2916	1.9630
43	H	-3.6626	-2.8487	2.4439
44	H	-2.5034	-3.9762	1.7356
45	C	-6.6262	-1.4384	-1.3337
46	H	-7.2388	-2.2905	-1.6452
47	H	-7.0660	-1.0500	-0.4129
48	H	-6.7270	-0.6781	-2.1118
49	C	-2.0947	-2.4904	-3.2619
50	H	-1.1645	-1.9351	-3.1031
51	H	-1.8083	-3.5303	-3.4500
52	H	-2.5654	-2.1100	-4.1687
53	C	1.7582	-3.5245	2.4250
54	H	0.8408	-2.9277	2.4537
55	H	1.4513	-4.5638	2.2699
56	H	2.2368	-3.4734	3.4042
57	C	2.6317	-2.5395	-2.4404
58	H	3.3509	-2.0300	-3.0828
59	H	2.4923	-3.5506	-2.8382
60	H	1.6671	-2.0333	-2.5404
61	C	6.3177	-1.9805	0.9337
62	H	6.9849	-2.8247	0.7326
63	H	6.6704	-1.1458	0.3232
64	H	6.4443	-1.7135	1.9846
65	C	0.0833	2.7474	1.4936
66	C	-0.5954	2.6984	2.7169
67	C	1.4046	3.2075	1.4657
68	C	0.0268	3.1306	3.8817
69	H	-1.6159	2.3281	2.7549
70	C	2.0254	3.6318	2.6355
71	H	1.9512	3.2354	0.5270
72	C	1.3376	3.5984	3.8436
73	H	-0.5152	3.1050	4.8214

74	H	3.0487	3.9911	2.5986
75	H	1.8199	3.9394	4.7536
76	C	-2.8410	3.6914	0.5509
77	C	-3.4361	1.4888	-0.2362
78	C	-4.1831	4.0409	0.5934
79	H	-2.0884	4.4184	0.8437
80	C	-4.7800	1.8475	-0.1984
81	H	-3.1558	0.4908	-0.5569
82	C	-5.1558	3.1183	0.2182
83	H	-4.4705	5.0353	0.9178
84	H	-5.5327	1.1245	-0.4939
85	H	-6.2046	3.3934	0.2504
86	C	-0.1526	3.0599	-1.3968
87	C	0.1802	4.4087	-1.2381
88	C	-0.1871	2.5162	-2.6843
89	C	0.4984	5.1872	-2.3448
90	H	0.1958	4.8558	-0.2491
91	C	0.1222	3.2996	-3.7892
92	H	-0.4592	1.4731	-2.8196
93	C	0.4729	4.6346	-3.6205
94	H	0.7619	6.2305	-2.2085
95	H	0.0893	2.8666	-4.7834
96	H	0.7194	5.2456	-4.4822
97	C	-2.4558	2.4075	0.1443
98	O	2.5718	0.0207	3.0758
99	C	1.8153	-0.2697	4.2645
100	H	0.9638	0.4080	4.3389
101	H	1.4606	-1.3004	4.2486
102	H	2.5047	-0.1136	5.0898

Ir(H)(H_2)($PhCO_2Me$)(IMes)(PPh_3)

1	C	-0.3868	-4.0035	0.9833
2	C	0.1114	-3.5983	-0.2617
3	C	-0.5928	-3.7958	-1.4517
4	C	-1.8683	-4.3582	-1.3599
5	C	-2.4145	-4.7543	-0.1427
6	C	-1.6487	-4.5860	1.0144
7	H	-2.0483	-4.9218	1.9690
8	C	-3.7827	-5.3585	-0.0669
9	H	-4.2541	-5.4186	-1.0488
10	H	-3.7485	-6.3701	0.3471
11	H	-4.4403	-4.7766	0.5861
12	C	0.4244	-3.8267	2.2299
13	H	-0.1802	-3.9861	3.1243
14	H	1.2541	-4.5402	2.2725
15	H	0.8719	-2.8298	2.2908
16	C	2.5899	-3.7099	-0.3663
17	C	3.5954	-2.8076	-0.3824
18	H	2.5918	-4.7868	-0.3953
19	H	4.6650	-2.9279	-0.4292
20	C	-0.0113	-3.4699	-2.7949
21	H	0.8473	-2.7985	-2.7364
22	H	0.3288	-4.3821	-3.2956
23	H	-0.7588	-3.0141	-3.4493
24	H	-2.4410	-4.5029	-2.2729
25	N	1.4120	-2.9870	-0.3034
26	N	3.0114	-1.5535	-0.3289
27	C	4.7751	1.4821	-1.5057
28	C	5.1403	1.2310	0.8503
29	H	4.9473	2.0221	-2.4335
30	C	4.0031	0.3235	-1.5392
31	C	3.4604	-0.2122	-2.8288
32	H	3.9117	-1.1787	-3.0777
33	H	2.3795	-0.3732	-2.7851
34	H	3.6685	0.4685	-3.6550
35	C	3.7908	-0.3440	-0.3279

36	C	4.3573	0.0776	0.8765
37	C	4.1055	-0.6648	2.1518
38	H	4.5559	-1.6627	2.1403
39	H	4.5162	-0.1266	3.0065
40	H	3.0328	-0.8016	2.3260
41	H	5.5814	1.5827	1.7797
42	C	5.3544	1.9505	-0.3236
43	C	6.2096	3.1797	-0.3304
44	H	5.7742	3.9708	-0.9451
45	H	6.3602	3.5742	0.6756
46	H	7.1991	2.9658	-0.7466
47	Ir	0.1971	-0.1411	-0.3336
48	C	1.6527	-1.6421	-0.2759
49	P	-1.5220	1.4986	-0.5395
50	C	-2.1274	2.0955	1.0758
51	C	-1.1489	3.0177	-1.4723
52	C	-2.9839	0.7573	-1.3569
53	C	-1.4095	3.0958	1.7428
54	C	-3.2038	1.4827	1.7217
55	C	-1.9166	4.1745	-1.2858
56	C	-0.1290	3.0239	-2.4276
57	C	-3.7533	1.4560	-2.2901
58	C	-3.3274	-0.5660	-1.0414
59	C	-1.7621	3.4698	3.0319
60	H	-0.5687	3.5784	1.2534
61	C	-3.5512	1.8599	3.0154
62	H	-3.7799	0.7175	1.2098
63	C	-1.6625	5.3131	-2.0394
64	H	-2.7120	4.1838	-0.5462
65	C	0.1193	4.1648	-3.1823
66	H	0.4741	2.1336	-2.5771
67	C	-4.8473	0.8447	-2.8947
68	H	-3.4944	2.4753	-2.5579
69	C	-4.4245	-1.1681	-1.6450
70	H	-2.7383	-1.1254	-0.3179
71	C	-2.8295	2.8483	3.6739
72	H	-1.1961	4.2444	3.5386
73	H	-4.3942	1.3834	3.5057
74	C	-0.6445	5.3098	-2.9883
75	H	-2.2606	6.2047	-1.8850
76	H	0.9143	4.1595	-3.9203
77	C	-5.1857	-0.4644	-2.5736
78	H	-5.4338	1.3953	-3.6223
79	H	-4.6805	-2.1922	-1.3910
80	H	-3.1025	3.1406	4.6822
81	H	-0.4466	6.2009	-3.5743
82	H	-6.0384	-0.9371	-3.0491
83	C	0.8884	0.7822	1.3514
84	C	0.2994	0.3680	2.5709
85	C	1.8227	1.8211	1.4196
86	C	0.6535	0.9389	3.8008
87	C	2.1745	2.3872	2.6405
88	H	2.2875	2.1891	0.5090
89	C	1.6029	1.9444	3.8358
90	H	0.1665	0.5958	4.7077
91	H	2.9089	3.1882	2.6619
92	H	1.8893	2.3951	4.7794
93	C	-0.7662	-0.6085	2.4421
94	H	-0.1930	-0.6570	-2.0058
95	H	-0.6874	-1.1586	-1.5330
96	H	1.1873	0.6916	-1.2173
97	O	-1.0918	-1.0919	1.3425
98	O	-1.3990	-0.9484	3.5555
99	C	-2.5355	-1.8103	3.3980
100	H	-2.2485	-2.7417	2.9086
101	H	-2.9034	-1.9955	4.4037

102	H	-3.3001	-1.3142	2.7960

Ir(H)$_2$(κ^2-PhCO$_2$Me)(IMes)(PPh$_3$)

1	Ir	-0.5165	-0.2571	0.3467
2	P	0.2604	1.8875	0.8452
3	C	-2.7311	-3.8915	0.0726
4	C	-1.4812	-4.3830	-0.0983
5	H	-3.6897	-4.3820	0.1057
6	H	-1.1230	-5.3896	-0.2359
7	N	-2.6080	-2.5200	0.2152
8	N	-0.6219	-3.2955	-0.0572
9	C	-1.3059	-2.1323	0.1356
10	C	0.8063	-3.3482	-0.1828
11	C	1.3631	-3.5418	-1.4521
12	C	1.5904	-3.1908	0.9672
13	C	2.7531	-3.5969	-1.5446
14	C	2.9764	-3.2307	0.8135
15	C	3.5757	-3.4467	-0.4273
16	H	3.2026	-3.7647	-2.5206
17	H	3.6027	-3.1163	1.6956
18	C	-3.6882	-1.5957	0.4259
19	C	-4.2147	-0.9225	-0.6813
20	C	-4.1091	-1.3470	1.7331
21	C	-5.2027	0.0289	-0.4446
22	C	-5.1013	-0.3837	1.9162
23	C	-5.6575	0.3140	0.8450
24	H	-5.6242	0.5676	-1.2896
25	H	-5.4429	-0.1704	2.9262
26	C	-3.4948	-2.0706	2.8907
27	H	-3.7054	-3.1443	2.8576
28	H	-2.4049	-1.9635	2.8923
29	H	-3.8736	-1.6899	3.8395
30	C	-6.7244	1.3422	1.0630
31	H	-6.4912	2.2767	0.5456
32	H	-7.6881	1.0000	0.6739
33	H	-6.8611	1.5657	2.1222
34	C	-3.6953	-1.1922	-2.0600
35	H	-4.2571	-0.6330	-2.8085
36	H	-2.6399	-0.9079	-2.1459
37	H	-3.7557	-2.2540	-2.3184
38	C	0.9685	-3.0113	2.3202
39	H	0.4438	-2.0535	2.4131
40	H	0.2249	-3.7878	2.5250
41	H	1.7244	-3.0523	3.1055
42	C	0.4993	-3.6626	-2.6698
43	H	-0.0138	-4.6286	-2.7123
44	H	-0.2756	-2.8901	-2.6865
45	H	1.0933	-3.5683	-3.5806
46	C	5.0656	-3.5499	-0.5537
47	H	5.3963	-4.5874	-0.4422
48	H	5.4178	-3.2151	-1.5330
49	H	5.5821	-2.9702	0.2146
50	C	-1.0291	3.1142	1.2550
51	C	-0.8204	4.4848	1.0661
52	C	-2.2482	2.6749	1.7830
53	C	-1.8112	5.3981	1.4071
54	H	0.1156	4.8394	0.6432
55	C	-3.2347	3.5925	2.1230
56	H	-2.4269	1.6124	1.9174
57	C	-3.0183	4.9538	1.9369
58	H	-1.6410	6.4586	1.2547
59	H	-4.1770	3.2393	2.5299
60	H	-3.7910	5.6687	2.1989
61	C	2.5077	3.3061	-0.1807
62	C	0.7486	2.7667	-1.7401
63	C	3.2287	3.9074	-1.2090

64	H	2.9168	3.2902	0.8257
65	C	1.4699	3.3690	-2.7631
66	H	-0.2212	2.3192	-1.9467
67	C	2.7141	3.9370	-2.5002
68	H	4.1921	4.3595	-0.9970
69	H	1.0615	3.3976	-3.7682
70	H	3.2761	4.4083	-3.2994
71	C	1.3692	1.8275	2.2971
72	C	1.4823	2.8658	3.2235
73	C	2.1395	0.6716	2.4705
74	C	2.3591	2.7517	4.2978
75	H	0.8772	3.7610	3.1112
76	C	3.0177	0.5611	3.5412
77	H	2.0449	-0.1495	1.7603
78	C	3.1288	1.6040	4.4564
79	H	2.4377	3.5608	5.0160
80	H	3.6075	-0.3416	3.6679
81	H	3.8081	1.5185	5.2976
82	C	1.2617	2.7281	-0.4374
83	H	-1.1296	-0.2304	1.7960
84	H	-1.8836	0.3424	-0.0435
85	O	2.3349	-0.3023	-0.8009
86	O	0.3839	-0.4005	-1.7679
87	C	2.1930	-0.3638	-3.2687
88	C	1.3945	0.1188	-4.3140
89	C	3.4430	-0.9212	-3.5618
90	C	1.8505	0.0728	-5.6215
91	H	0.4177	0.5266	-4.0782
92	C	3.8862	-0.9839	-4.8758
93	H	4.0546	-1.3428	-2.7739
94	C	3.0972	-0.4793	-5.9046
95	H	1.2319	0.4589	-6.4239
96	H	4.8494	-1.4296	-5.0981
97	H	3.4514	-0.5225	-6.9288
98	C	1.6036	-0.3478	-1.9187
99	C	3.6988	0.1480	-0.7569
100	H	3.9222	0.8191	-1.5871
101	H	3.7978	0.6868	0.1861
102	H	4.3714	-0.7119	-0.7564

2-Phenylpyridine

2-PhPy

1	C	-3.5315	0.0272	0.0169
2	C	-2.8040	1.2010	0.1897
3	C	-1.4173	1.1728	0.1821
4	C	-0.7262	-0.0327	0.0087
5	C	-1.4697	-1.2054	-0.1710
6	C	-2.8579	-1.1755	-0.1675
7	H	-4.6164	0.0498	0.0209
8	H	-3.3218	2.1442	0.3316
9	H	-0.8378	2.0799	0.3080
10	H	-0.9643	-2.1507	-0.3388
11	H	-3.4158	-2.0943	-0.3162
12	C	0.7527	-0.0359	0.0131
13	C	1.4943	-1.2129	0.1814
14	C	2.8787	-1.1520	0.1706
15	H	0.9943	-2.1618	0.3374
16	C	2.6835	1.1981	-0.1416
17	C	3.4975	0.0799	0.0003
18	H	3.4670	-2.0547	0.3017
19	H	3.1260	2.1852	-0.2673
20	H	4.5773	0.1756	-0.0150
21	N	1.3541	1.1575	-0.1374

Ir(H)$_2$(CHCl$_3$)(PhPy)(IMes)(PPh$_3$)

1	C	1.7511	-2.0354	3.4331
2	C	0.4381	-2.3001	3.0319
3	C	-0.6725	-1.9651	3.8086
4	C	-0.4385	-1.3168	5.0224
5	C	0.8521	-1.0239	5.4618
6	C	1.9307	-1.3876	4.6522
7	H	2.9426	-1.1694	4.9845
8	C	1.0898	-0.3636	6.7855
9	H	1.7944	0.4682	6.6997
10	H	0.1644	0.0161	7.2228
11	H	1.5219	-1.0677	7.5032
12	C	2.9141	-2.4428	2.5823
13	H	3.8559	-2.1038	3.0149
14	H	2.9726	-3.5308	2.4737
15	H	2.8347	-2.0307	1.5706
16	C	0.1526	-4.3521	1.6824
17	C	-0.0949	-4.6381	0.3858
18	H	0.2866	-4.9869	2.5425
19	H	-0.2178	-5.5749	-0.1317
20	C	-2.0570	-2.2983	3.3468
21	H	-2.3185	-1.7499	2.4342
22	H	-2.1596	-3.3627	3.1121
23	H	-2.7978	-2.0496	4.1065
24	H	-1.2897	-1.0469	5.6440
25	N	0.2165	-2.9746	1.7818
26	N	-0.1797	-3.4262	-0.2783
27	C	-1.9714	-3.0784	-3.4942
28	C	0.3718	-3.3255	-3.9358
29	H	-2.9887	-2.9389	-3.8520
30	C	-1.7518	-3.1773	-2.1209
31	C	-2.8877	-3.1313	-1.1453
32	H	-2.6510	-2.5248	-0.2663
33	H	-3.7881	-2.7254	-1.6110
34	H	-3.1336	-4.1342	-0.7789
35	C	-0.4325	-3.3329	-1.6877
36	C	0.6408	-3.4692	-2.5772
37	C	2.0186	-3.8163	-2.1004
38	H	2.7806	-3.4550	-2.7931
39	H	2.2397	-3.4114	-1.1101
40	H	2.1345	-4.9035	-2.0301
41	H	1.1970	-3.3887	-4.6418
42	C	-0.9240	-3.1170	-4.4129
43	C	-1.1734	-2.9393	-5.8789
44	H	-0.8422	-3.8103	-6.4508
45	H	-2.2318	-2.7840	-6.0942
46	H	-0.6196	-2.0808	-6.2722
47	Ir	0.1045	-0.3058	0.2982
48	H	-0.7368	-0.2696	1.5920
49	H	1.3941	-0.4109	1.2104
50	C	0.0100	-2.3693	0.5704
51	P	0.5027	2.0131	0.4334
52	C	-0.6880	3.0951	-0.4387
53	C	0.5713	2.6915	2.1310
54	C	2.1339	2.5228	-0.2464
55	C	-0.7759	2.9716	-1.8307
56	C	-1.5775	3.9489	0.2187
57	C	0.2297	1.9147	3.2395
58	C	0.9679	4.0213	2.3242
59	C	3.2580	1.7729	0.1253
60	C	2.3029	3.6134	-1.1060
61	C	-1.7187	3.6916	-2.5498
62	H	-0.0921	2.3082	-2.3562
63	C	-2.5302	4.6626	-0.5027
64	H	-1.5312	4.0585	1.2981
65	C	0.2585	2.4674	4.5168

66	H	-0.0523	0.8743	3.1096
67	C	1.0032	4.5660	3.5999
68	H	1.2494	4.6324	1.4702
69	C	4.5144	2.0899	-0.3737
70	H	3.1388	0.9255	0.7953
71	C	3.5647	3.9256	-1.6083
72	H	1.4510	4.2219	-1.3908
73	C	-2.6020	4.5382	-1.8849
74	H	-1.7656	3.5888	-3.6296
75	H	-3.2144	5.3219	0.0205
76	C	0.6417	3.7904	4.6991
77	H	-0.0205	1.8550	5.3684
78	H	1.3126	5.5964	3.7382
79	C	4.6697	3.1611	-1.2511
80	H	5.3745	1.4954	-0.0839
81	H	3.6803	4.7710	-2.2781
82	H	-3.3417	5.1010	-2.4442
83	H	0.6653	4.2188	5.6955
84	H	5.6497	3.4008	-1.6493
85	Cl	2.0347	-0.6385	-1.7610
86	C	2.6028	0.5269	-3.0247
87	H	2.7793	1.4739	-2.5191
88	Cl	1.3634	0.7821	-4.2650
89	C	-1.5142	-0.0887	-2.3398
90	C	-3.0522	0.2953	-0.6607
91	C	-2.4213	0.2097	-3.3406
92	H	-0.5102	-0.4129	-2.5951
93	C	-4.0181	0.6080	-1.6234
94	C	-3.7056	0.5813	-2.9729
95	H	-2.1157	0.1362	-4.3787
96	H	-5.0106	0.8861	-1.2878
97	H	-4.4517	0.8386	-3.7172
98	C	-3.4789	0.2753	0.7589
99	C	-4.6018	-0.4882	1.1030
100	C	-2.8710	1.0483	1.7516
101	C	-5.0887	-0.4918	2.4047
102	H	-5.0898	-1.0902	0.3413
103	C	-3.3592	1.0490	3.0524
104	H	-2.0298	1.6791	1.4976
105	C	-4.4698	0.2794	3.3826
106	H	-5.9552	-1.0954	2.6530
107	H	-2.8752	1.6667	3.8030
108	H	-4.8557	0.2844	4.3967
109	N	-1.7865	-0.0326	-1.0243
110	Cl	4.1068	-0.0521	-3.7122

$Ir(H)_2(DCM)(PhPy)(IMes)(PPh_3)$

1	C	-1.2622	-3.9446	0.9130
2	C	-0.6465	-3.7018	-0.3207
3	C	-1.3579	-3.6733	-1.5192
4	C	-2.7401	-3.8553	-1.4552
5	C	-3.4009	-4.0676	-0.2474
6	C	-2.6403	-4.1290	0.9223
7	H	-3.1392	-4.3022	1.8729
8	C	-4.8902	-4.2086	-0.1896
9	H	-5.3389	-3.3737	0.3580
10	H	-5.3350	-4.2303	-1.1853
11	H	-5.1868	-5.1231	0.3308
12	C	-0.4726	-3.9248	2.1846
13	H	-1.0943	-4.1948	3.0393
14	H	0.3792	-4.6111	2.1575
15	H	-0.0625	-2.9242	2.3700
16	C	1.7521	-4.3439	-0.4430
17	C	2.9319	-3.6934	-0.3322
18	H	1.5227	-5.3861	-0.5906
19	H	3.9498	-4.0454	-0.3569

20	C	-0.6668	-3.4427	-2.8265
21	H	0.0838	-2.6507	-2.7514
22	H	-0.1452	-4.3431	-3.1693
23	H	-1.3806	-3.1632	-3.6035
24	H	-3.3152	-3.8201	-2.3773
25	N	0.7585	-3.3913	-0.3287
26	N	2.6318	-2.3524	-0.1607
27	C	5.2202	0.2756	-0.5688
28	C	4.9679	-0.2280	1.7582
29	H	5.6939	0.8450	-1.3655
30	C	4.2514	-0.6705	-0.9090
31	C	3.9038	-0.8932	-2.3482
32	H	3.1562	-1.6756	-2.4847
33	H	3.5188	0.0235	-2.8063
34	H	4.7907	-1.1786	-2.9207
35	C	3.6362	-1.3689	0.1320
36	C	4.0035	-1.1903	1.4753
37	C	3.4310	-2.0401	2.5686
38	H	3.9952	-2.9743	2.6663
39	H	3.4817	-1.5285	3.5312
40	H	2.3909	-2.3172	2.3854
41	H	5.2553	-0.0665	2.7942
42	C	5.5787	0.5247	0.7525
43	C	6.6043	1.5606	1.0958
44	H	6.7929	2.2365	0.2598
45	H	6.2947	2.1600	1.9565
46	H	7.5598	1.0992	1.3635
47	Ir	0.0796	-0.4429	0.0373
48	H	-0.5218	-0.8806	-1.3212
49	H	-0.9231	-1.4562	0.6793
50	C	1.2814	-2.1325	-0.1524
51	P	-1.7406	1.0204	0.2607
52	C	-1.4054	2.5857	1.1537
53	C	-2.5720	1.5260	-1.2879
54	C	-3.1331	0.2812	1.2091
55	C	-0.5440	3.5255	0.5698
56	C	-1.8812	2.8213	2.4473
57	C	-3.0221	2.8266	-1.5253
58	C	-2.8799	0.5177	-2.2092
59	C	-4.4626	0.5917	0.9057
60	C	-2.8698	-0.5751	2.2838
61	C	-0.1668	4.6689	1.2637
62	H	-0.1776	3.3678	-0.4413
63	C	-1.4983	3.9665	3.1413
64	H	-2.5567	2.1110	2.9144
65	C	-3.7657	3.1138	-2.6668
66	H	-2.8130	3.6164	-0.8107
67	C	-3.6381	0.8052	-3.3350
68	H	-2.5414	-0.5001	-2.0298
69	C	-5.5018	0.0477	1.6533
70	H	-4.6939	1.2545	0.0780
71	C	-3.9096	-1.1108	3.0338
72	H	-1.8445	-0.8271	2.5337
73	C	-0.6378	4.8889	2.5562
74	H	0.4943	5.3898	0.7930
75	H	-1.8818	4.1400	4.1416
76	C	-4.0821	2.1042	-3.5673
77	H	-4.1130	4.1271	-2.8399
78	H	-3.8769	0.0138	-4.0383
79	C	-5.2291	-0.8042	2.7177
80	H	-6.5281	0.2925	1.4008
81	H	-3.6868	-1.7732	3.8645
82	H	-0.3431	5.7805	3.0991
83	H	-4.6739	2.3275	-4.4490
84	H	-6.0417	-1.2272	3.2989
85	Cl	0.8086	0.1023	2.7358

86	C	1.2348	1.7864	3.2225
87	H	0.9841	2.4377	2.3869
88	H	0.6460	2.0111	4.1077
89	Cl	2.9453	1.9684	3.6080
90	C	2.3722	1.7050	0.0728
91	C	1.3708	1.9472	-1.9934
92	C	3.0353	2.9163	-0.0214
93	H	2.5364	1.0656	0.9344
94	C	2.0337	3.1709	-2.1650
95	C	2.8557	3.6736	-1.1709
96	H	3.6884	3.2322	0.7844
97	H	1.9099	3.7033	-3.1008
98	H	3.3654	4.6220	-1.3042
99	C	0.5316	1.4429	-3.0958
100	C	-0.2405	2.3370	-3.8488
101	C	0.5812	0.1021	-3.4891
102	C	-0.9306	1.9052	-4.9718
103	H	-0.3145	3.3756	-3.5382
104	C	-0.0990	-0.3256	-4.6226
105	H	1.1868	-0.5912	-2.9161
106	C	-0.8512	0.5745	-5.3695
107	H	-1.5338	2.6084	-5.5360
108	H	-0.0228	-1.3610	-4.9373
109	H	-1.3749	0.2401	-6.2589
110	N	1.5302	1.2213	-0.8584

$Ir(H)_2(PhPy)_2(IMes)(PPh_3)$

1	C	0.5119	3.7314	2.0748
2	C	1.4995	3.4439	1.1190
3	C	2.8554	3.3882	1.4432
4	C	3.2069	3.5815	2.7823
5	C	2.2569	3.8010	3.7740
6	C	0.9117	3.8779	3.3986
7	H	0.1568	4.0833	4.1542
8	C	2.6556	3.9690	5.2079
9	H	2.5061	4.9994	5.5441
10	H	2.0535	3.3360	5.8664
11	H	3.7063	3.7219	5.3681
12	C	-0.9155	3.9180	1.6674
13	H	-1.5532	4.0772	2.5383
14	H	-1.0260	4.7866	1.0083
15	H	-1.2987	3.0580	1.1125
16	C	1.1674	4.2880	-1.1735
17	C	0.5311	3.8551	-2.2819
18	H	1.6716	5.2134	-0.9494
19	H	0.3607	4.3241	-3.2367
20	C	3.9173	3.0655	0.4376
21	H	4.2756	2.0383	0.5730
22	H	3.5702	3.1556	-0.5922
23	H	4.7841	3.7187	0.5603
24	H	4.2581	3.5263	3.0560
25	N	1.0731	3.2674	-0.2384
26	N	0.0579	2.5854	-2.0045
27	C	-1.1307	0.3662	-4.7386
28	C	-3.0159	1.4042	-3.6865
29	H	-0.7201	-0.2991	-5.4951
30	C	-0.2514	1.0462	-3.8983
31	C	1.2284	0.9240	-4.0719
32	H	1.7527	0.9785	-3.1172
33	H	1.4992	-0.0166	-4.5565
34	H	1.6178	1.7342	-4.6995
35	C	-0.8006	1.8794	-2.9205
36	C	-2.1768	2.0997	-2.8159
37	C	-2.7162	3.0522	-1.7984
38	H	-2.1937	4.0135	-1.8256
39	H	-3.7776	3.2397	-1.9551
40	H	-2.5927	2.6536	-0.7854
41	H	-4.0900	1.5610	-3.6132
42	C	-2.5140	0.5271	-4.6478
43	C	-3.4255	-0.2018	-5.5871
44	H	-3.3195	0.1730	-6.6097
45	H	-3.1969	-1.2707	-5.6199
46	H	-4.4741	-0.0864	-5.3066
47	Ir	0.0657	0.2326	-0.0743
48	H	-1.3062	0.3819	-0.8031
49	H	0.8110	-0.0220	-1.4154
50	C	0.3840	2.1878	-0.7267
51	P	-0.3617	-2.0821	-0.0757
52	C	-0.2528	-2.8945	1.5543
53	C	-1.9815	-2.6176	-0.7736
54	C	0.6800	-3.1055	-1.1964
55	C	-1.3721	-3.0141	2.3850
56	C	1.0118	-3.1830	2.0833
57	C	-2.5181	-3.8730	-0.4650
58	C	-2.5930	-1.8503	-1.7689
59	C	1.0262	-4.4270	-0.9020
60	C	0.9367	-2.6080	-2.4781
61	C	-1.2308	-3.4341	3.7031
62	H	-2.3587	-2.7634	2.0052
63	C	1.1521	-3.5924	3.4036
64	H	1.8922	-3.0855	1.4531
65	C	-3.6654	-4.3274	-1.1033

66	H	-2.0345	-4.5062	0.2727
67	C	-3.7340	-2.3142	-2.4148
68	H	-2.1790	-0.8838	-2.0427
69	C	1.6349	-5.2255	-1.8651
70	H	0.8067	-4.8462	0.0749
71	C	1.5258	-3.4142	-3.4422
72	H	0.6509	-1.5897	-2.7271
73	C	0.0292	-3.7219	4.2168
74	H	-2.1094	-3.5283	4.3330
75	H	2.1392	-3.8175	3.7952
76	C	-4.2795	-3.5476	-2.0779
77	H	-4.0745	-5.2983	-0.8447
78	H	-4.1977	-1.7016	-3.1822
79	C	1.8802	-4.7246	-3.1381
80	H	1.9003	-6.2493	-1.6222
81	H	1.7144	-3.0146	-4.4342
82	H	0.1364	-4.0490	5.2456
83	H	-5.1730	-3.9057	-2.5781
84	H	2.3417	-5.3543	-3.8917
85	C	-0.5426	-0.1005	3.1977
86	C	-2.3968	1.0663	2.5064
87	C	-0.7770	0.1677	4.5369
88	C	-2.6870	1.3986	3.8356
89	C	-1.8598	0.9719	4.8609
90	H	-0.1276	-0.2599	5.2926
91	H	-3.5978	1.9447	4.0531
92	H	-2.0867	1.2211	5.8923
93	N	-1.2741	0.3782	2.1799
94	C	3.2732	-0.7942	0.8685
95	C	2.1867	0.5626	2.3751
96	C	4.1728	-1.1866	1.8676
97	C	3.0518	0.2353	3.4075
98	C	4.0549	-0.6877	3.1540
99	H	4.9911	-1.8463	1.6028
100	H	2.9311	0.7065	4.3768
101	H	4.7557	-0.9840	3.9275
102	N	2.2374	0.0415	1.1386
103	H	0.2849	-0.7402	2.9181
104	H	1.3921	1.2791	2.5471
105	C	3.5585	-1.2130	-0.5151
106	C	3.5294	-0.2730	-1.5491
107	C	4.0473	-2.4980	-0.7836
108	C	4.0155	-0.5999	-2.8094
109	H	3.1638	0.7282	-1.3377
110	C	4.5095	-2.8288	-2.0482
111	H	4.0568	-3.2453	0.0054
112	C	4.5109	-1.8740	-3.0606
113	H	4.0262	0.1498	-3.5938
114	H	4.8719	-3.8322	-2.2441
115	H	4.8961	-2.1243	-4.0436
116	C	-3.4011	1.3655	1.4700
117	C	-3.6944	0.4065	0.4957
118	C	-4.1818	2.5278	1.5277
119	C	-4.7454	0.5988	-0.3916
120	H	-3.1135	-0.5085	0.4646
121	C	-5.2302	2.7203	0.6390
122	H	-3.9616	3.2890	2.2706
123	C	-5.5201	1.7517	-0.3179
124	H	-4.9689	-0.1677	-1.1269
125	H	-5.8243	3.6263	0.6945
126	H	-6.3494	1.8964	-1.0030

$Ir(H)_2(PhPy)(IMes)(PPh_3)$

1	C	1.1502	-3.7221	-1.7620
2	C	1.0443	-3.5581	-0.3803
3	C	2.1640	-3.5349	0.4633

4	C	3.4183	-3.6563	-0.1231
5	C	3.5764	-3.7793	-1.5062
6	C	2.4350	-3.8147	-2.3029
7	H	2.5408	-3.9228	-3.3800
8	C	4.9455	-3.8466	-2.1078
9	H	5.4883	-2.9095	-1.9481
10	H	5.5449	-4.6388	-1.6516
11	H	4.9084	-4.0308	-3.1822
12	C	-0.0609	-3.8341	-2.6350
13	H	-0.3819	-4.8779	-2.7229
14	H	-0.9111	-3.2742	-2.2400
15	H	0.1459	-3.4780	-3.6462
16	C	-1.0020	-4.4971	0.6570
17	C	-2.1060	-3.9866	1.2450
18	H	-0.6726	-5.5119	0.5077
19	H	-2.9512	-4.4627	1.7135
20	C	2.0061	-3.3081	1.9355
21	H	2.9620	-3.3942	2.4531
22	H	1.5997	-2.3079	2.1387
23	H	1.3087	-4.0173	2.3918
24	H	4.2998	-3.6252	0.5130
25	N	-0.2524	-3.4176	0.2192
26	N	-2.0059	-2.6095	1.1596
27	C	-3.8529	-0.4627	3.5427
28	C	-5.0227	-0.4809	1.4506
29	H	-3.7705	-0.1279	4.5739
30	C	-2.8707	-1.3123	3.0338
31	C	-1.7254	-1.7772	3.8781
32	H	-1.7502	-2.8618	4.0276
33	H	-0.7646	-1.5456	3.4085
34	H	-1.7455	-1.3079	4.8620
35	C	-3.0006	-1.7257	1.7055
36	C	-4.0714	-1.3336	0.8966
37	C	-4.2043	-1.8358	-0.5078
38	H	-4.8492	-1.1860	-1.1013
39	H	-3.2340	-1.9135	-1.0056
40	H	-4.6457	-2.8382	-0.5311
41	H	-5.8593	-0.1589	0.8354
42	C	-4.9348	-0.0388	2.7711
43	C	-5.9970	0.8461	3.3478
44	H	-6.2772	1.6433	2.6544
45	H	-6.9083	0.2777	3.5583
46	H	-5.6795	1.3042	4.2862
47	Ir	-0.2626	-0.2591	0.1966
48	H	-1.7805	0.0637	0.2731
49	H	-0.3665	-0.1138	1.7773
50	C	-0.8582	-2.2269	0.5180
51	P	0.0779	2.0648	0.1700
52	C	0.4335	2.7289	-1.4934
53	C	-1.3830	3.0173	0.7321
54	C	1.4045	2.7164	1.2483
55	C	-0.6112	2.7777	-2.4265
56	C	1.7307	3.0255	-1.9165
57	C	-1.6250	4.3177	0.2758
58	C	-2.2155	2.4746	1.7158
59	C	1.8455	4.0397	1.1337
60	C	1.9375	1.9079	2.2541
61	C	-0.3640	3.1311	-3.7455
62	H	-1.6252	2.5420	-2.1111
63	C	1.9755	3.3823	-3.2395
64	H	2.5569	2.9715	-1.2138
65	C	-2.6855	5.0554	0.7876
66	H	-0.9892	4.7550	-0.4885
67	C	-3.2672	3.2217	2.2331
68	H	-2.0401	1.4660	2.0792
69	C	2.8204	4.5314	1.9912

70	H	1.4273	4.6885	0.3692
71	C	2.9100	2.4040	3.1159
72	H	1.5788	0.8878	2.3608
73	C	0.9321	3.4363	-4.1555
74	H	-1.1839	3.1723	-4.4553
75	H	2.9880	3.6199	-3.5510
76	C	-3.5066	4.5104	1.7694
77	H	-2.8675	6.0601	0.4211
78	H	-3.8982	2.7919	3.0041
79	C	3.3572	3.7128	2.9814
80	H	3.1581	5.5574	1.8912
81	H	3.3157	1.7679	3.8961
82	H	1.1242	3.7204	-5.1848
83	H	-4.3296	5.0911	2.1723
84	H	4.1164	4.1003	3.6524
85	C	1.1577	-0.4243	-2.6705
86	C	-1.1583	-0.5551	-2.7824
87	C	1.2269	-0.5490	-4.0639
88	C	-1.1551	-0.6397	-4.1621
89	H	-2.0885	-0.5510	-2.2236
90	C	0.0713	-0.6509	-4.8164
91	H	2.1952	-0.5737	-4.5483
92	H	-2.0916	-0.7055	-4.7025
93	H	0.1258	-0.7401	-5.8959
94	C	2.3692	-0.2164	-1.8604
95	C	3.5296	0.3146	-2.4441
96	C	2.3994	-0.4772	-0.4838
97	C	4.6583	0.5833	-1.6855
98	H	3.5324	0.5701	-3.4979
99	C	3.5312	-0.2126	0.2789
100	H	1.6164	-1.0897	-0.0074
101	C	4.6625	0.3282	-0.3161
102	H	5.5355	1.0077	-2.1621
103	H	3.5300	-0.4484	1.3379
104	H	5.5429	0.5434	0.2796
105	N	-0.0427	-0.4590	-2.0389

Ir(H)$_2$(PhPy)$_2$(IMes)(PPh$_3$) C-C Rotation TS2 ***Imaginary Frequency: -57.1 cm^{-1}***

1	C	1.2943	-4.1368	-1.6378
2	C	1.1081	-3.7361	-0.3116
3	C	2.1036	-3.8729	0.6637
4	C	3.3117	-4.4444	0.2746
5	C	3.5472	-4.8555	-1.0392
6	C	2.5319	-4.6833	-1.9807
7	H	2.7000	-4.9949	-3.0086
8	C	4.8773	-5.4175	-1.4334
9	H	5.5976	-4.6138	-1.6236
10	H	5.3009	-6.0425	-0.6448
11	H	4.8132	-6.0144	-2.3443
12	C	0.1949	-4.0144	-2.6477
13	H	-0.5141	-4.8459	-2.5714
14	H	-0.3822	-3.0960	-2.5115
15	H	0.5920	-4.0223	-3.6639
16	C	-1.1402	-4.3520	0.3829
17	C	-2.2429	-3.7351	0.8521
18	H	-0.9114	-5.3923	0.2223
19	H	-3.1947	-4.1190	1.1792
20	C	1.8717	-3.4147	2.0695
21	H	2.7099	-3.6810	2.7139
22	H	1.7450	-2.3266	2.1151
23	H	0.9631	-3.8508	2.4969
24	H	4.0926	-4.5731	1.0204
25	N	-0.2109	-3.3579	0.1120
26	N	-1.9667	-2.3812	0.8641
27	C	-3.9842	-0.3673	3.2157
28	C	-4.8015	0.0103	0.9966

29	H	-4.0438	-0.1857	4.2863
30	C	-2.9890	-1.2156	2.7355
31	C	-1.9981	-1.8588	3.6549
32	H	-2.0709	-2.9511	3.6342
33	H	-0.9724	-1.6073	3.3649
34	H	-2.1497	-1.5363	4.6852
35	C	-2.9370	-1.4399	1.3554
36	C	-3.8397	-0.8482	0.4660
37	C	-3.8078	-1.1276	-1.0069
38	H	-2.9078	-1.6658	-1.3100
39	H	-4.6676	-1.7337	-1.3095
40	H	-3.8612	-0.1990	-1.5834
41	H	-5.5069	0.4867	0.3195
42	C	-4.8970	0.2572	2.3651
43	C	-5.9497	1.1784	2.8994
44	H	-5.8367	2.1866	2.4889
45	H	-6.9526	0.8375	2.6285
46	H	-5.9079	1.2536	3.9872
47	Ir	-0.0149	-0.1556	0.2064
48	H	-1.5188	0.1555	0.0624
49	H	-0.2612	-0.0613	1.7748
50	C	-0.6994	-2.1124	0.4025
51	P	0.0319	2.1979	0.1484
52	C	-0.4293	2.7447	-1.5390
53	C	-1.2842	2.9832	1.1631
54	C	1.4699	3.1827	0.7005
55	C	-1.7857	2.6843	-1.8870
56	C	0.5041	3.0216	-2.5424
57	C	-1.5684	4.3396	0.9554
58	C	-1.9529	2.2971	2.1754
59	C	1.8437	4.4060	0.1379
60	C	2.1509	2.7113	1.8275
61	C	-2.1946	2.9102	-3.1956
62	H	-2.5276	2.4660	-1.1220
63	C	0.0931	3.2473	-3.8522
64	H	1.5618	3.0783	-2.3079
65	C	-2.5034	4.9928	1.7439
66	H	-1.0569	4.8875	0.1685
67	C	-2.8805	2.9606	2.9749
68	H	-1.7505	1.2442	2.3400
69	C	2.8939	5.1348	0.6870
70	H	1.3120	4.8002	-0.7228
71	C	3.1833	3.4531	2.3865
72	H	1.8650	1.7574	2.2653
73	C	-1.2561	3.1926	-4.1831
74	H	-3.2510	2.8736	-3.4424
75	H	0.8326	3.4717	-4.6141
76	C	-3.1587	4.3038	2.7621
77	H	-2.7165	6.0421	1.5702
78	H	-3.3852	2.4184	3.7682
79	C	3.5615	4.6630	1.8125
80	H	3.1812	6.0813	0.2414
81	H	3.6982	3.0828	3.2668
82	H	-1.5751	3.3761	-5.2035
83	H	-3.8817	4.8172	3.3876
84	H	4.3718	5.2405	2.2444
85	C	1.7560	-0.3327	-2.4353
86	C	-0.5085	-0.3248	-2.9212
87	C	2.0611	-0.4288	-3.7918
88	C	-0.2749	-0.3967	-4.2835
89	H	-1.5174	-0.2760	-2.5245
90	C	1.0387	-0.4621	-4.7292
91	H	3.1023	-0.4604	-4.0930
92	H	-1.1103	-0.3999	-4.9734
93	H	1.2641	-0.5270	-5.7882
94	C	2.8437	-0.2311	-1.4250

95	C	3.2861	1.0203	-0.9880
96	C	3.5048	-1.3743	-0.9728
97	C	4.3512	1.1272	-0.1026
98	H	2.8106	1.9191	-1.3611
99	C	4.5608	-1.2679	-0.0739
100	H	3.2051	-2.3425	-1.3511
101	C	4.9850	-0.0186	0.3650
102	H	4.6837	2.1093	0.2157
103	H	5.0615	-2.1681	0.2690
104	H	5.8182	0.0632	1.0549
105	N	0.4750	-0.3029	-2.0050

$Ir(H)_2(PhPy)_2(IMes)(PPh_3)$ C-H Activation TS1 *Imaginary Frequency: -726.9 cm^{-1}*

1	Ir	-0.0280	0.3504	-0.1239
2	H	1.2232	1.2931	0.3321
3	P	1.5579	-1.3868	-0.4435
4	C	-1.6184	4.3470	-0.1915
5	C	-2.7099	3.7279	0.3089
6	H	-1.4296	5.3788	-0.4364
7	H	-3.6786	4.1013	0.5965
8	N	-0.6548	3.3712	-0.3771
9	N	-2.3900	2.3846	0.4177
10	C	-1.1145	2.1431	0.0040
11	H	0.4782	0.8621	-1.5495
12	C	-1.8820	-1.7056	1.0915
13	C	-1.5428	-1.2463	-0.2118
14	C	-2.0902	-1.9395	-1.3004
15	C	-2.9600	-3.0111	-1.1354
16	C	-3.3111	-3.4310	0.1428
17	C	-2.7656	-2.7844	1.2379
18	H	-1.1456	0.0035	-1.2128
19	H	-1.8375	-1.6343	-2.3118
20	H	-3.3640	-3.5135	-2.0086
21	H	-3.9921	-4.2632	0.2845
22	H	-3.0193	-3.1386	2.2322
23	C	-1.2246	-1.1207	2.2543
24	C	-3.3312	1.3951	0.8680
25	C	-4.0938	0.7169	-0.0932
26	C	-3.4920	1.1876	2.2378
27	C	-5.0016	-0.2332	0.3589
28	C	-4.4158	0.2199	2.6386
29	C	-5.1705	-0.5033	1.7195
30	H	-5.5867	-0.7871	-0.3712
31	H	-4.5424	0.0282	3.7018
32	C	0.6326	3.6509	-0.9557
33	C	0.7697	3.5636	-2.3449
34	C	1.6879	4.0050	-0.1147
35	C	2.0247	3.8304	-2.8852
36	C	2.9258	4.2600	-0.7051
37	C	3.1142	4.1804	-2.0841
38	H	2.1541	3.7679	-3.9628
39	H	3.7641	4.5297	-0.0674
40	C	1.4920	4.1079	1.3654
41	H	0.9291	5.0080	1.6350
42	H	0.9243	3.2560	1.7521
43	H	2.4477	4.1516	1.8888
44	C	4.4446	4.4825	-2.7027
45	H	4.7403	3.7109	-3.4185
46	H	4.4156	5.4267	-3.2549
47	H	5.2311	4.5702	-1.9512
48	C	-0.3917	3.1889	-3.2128
49	H	-1.2410	3.8650	-3.0721
50	H	-0.1183	3.2128	-4.2679
51	H	-0.7533	2.1814	-2.9805
52	C	-2.7276	1.9848	3.2494
53	H	-1.7329	2.2644	2.8944

54	H	-3.2504	2.9167	3.4914
55	H	-2.6144	1.4313	4.1834
56	C	-3.9521	1.0225	-1.5519
57	H	-4.5107	0.3083	-2.1579
58	H	-4.3282	2.0242	-1.7872
59	H	-2.9085	1.0011	-1.8796
60	C	-6.1442	-1.5485	2.1674
61	H	-7.1761	-1.2348	1.9839
62	H	-5.9993	-2.4845	1.6202
63	H	-6.0512	-1.7598	3.2341
64	C	1.5021	-2.5953	0.9185
65	C	2.2061	-2.3364	2.1009
66	C	0.6158	-3.6768	0.8782
67	C	2.0420	-3.1555	3.2105
68	H	2.8889	-1.4919	2.1494
69	C	0.4572	-4.4961	1.9902
70	H	0.0402	-3.8747	-0.0220
71	C	1.1693	-4.2388	3.1565
72	H	2.5991	-2.9488	4.1185
73	H	-0.2281	-5.3363	1.9418
74	H	1.0479	-4.8823	4.0216
75	C	4.3340	-1.7572	-0.3005
76	C	3.6205	0.4640	-0.9173
77	C	5.6601	-1.3704	-0.4395
78	H	4.1022	-2.7761	-0.0034
79	C	4.9508	0.8441	-1.0608
80	H	2.8315	1.1855	-1.1038
81	C	5.9710	-0.0680	-0.8190
82	H	6.4521	-2.0875	-0.2520
83	H	5.1861	1.8608	-1.3581
84	H	7.0079	0.2323	-0.9267
85	C	1.3781	-2.3635	-1.9793
86	C	1.8769	-3.6677	-2.0698
87	C	0.8324	-1.7658	-3.1182
88	C	1.8072	-4.3643	-3.2696
89	H	2.3172	-4.1454	-1.1999
90	C	0.7705	-2.4631	-4.3190
91	H	0.4575	-0.7479	-3.0630
92	C	1.2523	-3.7649	-4.3954
93	H	2.1916	-5.3771	-3.3253
94	H	0.3455	-1.9876	-5.1967
95	H	1.2008	-4.3103	-5.3316
96	C	3.3014	-0.8393	-0.5279
97	N	-0.3250	-0.1396	1.9887
98	C	-1.4289	-1.5323	3.5777
99	H	-2.1506	-2.3101	3.7931
100	C	-0.7059	-0.9588	4.6057
101	H	-0.8592	-1.2857	5.6286
102	C	0.3789	0.4127	2.9916
103	H	1.0930	1.1742	2.6972
104	C	0.2240	0.0350	4.3106
105	H	0.8189	0.5076	5.0823

Ir(H)(H_2)(PhPy)(IMes)(PPh_3)

1	Ir	0.1198	-0.1846	0.3614
2	H	1.1301	0.4677	1.6994
3	P	-1.3536	1.6843	0.3427
4	C	2.9033	-3.4248	1.0248
5	C	1.7676	-4.1432	0.8968
6	H	3.9163	-3.7151	1.2485
7	H	1.5738	-5.1991	0.9856
8	N	2.5594	-2.1015	0.8059
9	N	0.7561	-3.2466	0.6016
10	C	1.2264	-1.9654	0.5373
11	H	0.4573	0.1190	2.0904
12	C	-0.0101	-0.2377	-2.5977

13	C	-0.6960	-0.7001	-1.4464
14	C	-1.8978	-1.3876	-1.6376
15	C	-2.3812	-1.6600	-2.9128
16	C	-1.6681	-1.2543	-4.0399
17	C	-0.4925	-0.5403	-3.8796
18	H	-1.0009	-0.9946	1.1381
19	H	-2.4632	-1.7285	-0.7752
20	H	-3.3167	-2.2012	-3.0266
21	H	-2.0362	-1.4812	-5.0345
22	H	0.0444	-0.2000	-4.7598
23	C	1.1264	0.6516	-2.3850
24	C	-0.5969	-3.6829	0.3782
25	C	-1.5124	-3.6412	1.4340
26	C	-0.9221	-4.1917	-0.8825
27	C	-2.8165	-4.0573	1.1678
28	C	-2.2278	-4.6331	-1.0826
29	C	-3.1952	-4.5493	-0.0820
30	H	-3.5502	-4.0179	1.9696
31	H	-2.5030	-5.0207	-2.0604
32	C	3.5396	-1.0518	0.8286
33	C	3.7897	-0.3638	2.0187
34	C	4.2678	-0.8148	-0.3451
35	C	4.7794	0.6236	1.9957
36	C	5.2638	0.1545	-0.3038
37	C	5.5309	0.8904	0.8536
38	H	4.9843	1.1772	2.9090
39	H	5.8334	0.3572	-1.2076
40	C	3.9627	-1.5692	-1.6021
41	H	4.2703	-2.6178	-1.5302
42	H	2.8892	-1.5744	-1.8174
43	H	4.4794	-1.1327	-2.4577
44	C	6.6142	1.9244	0.8635
45	H	6.5902	2.5431	-0.0372
46	H	6.5413	2.5820	1.7308
47	H	7.6024	1.4552	0.8928
48	C	3.0839	-0.6898	3.3009
49	H	2.7744	0.2175	3.8266
50	H	2.2024	-1.3151	3.1528
51	H	3.7524	-1.2302	3.9785
52	C	0.0824	-4.2043	-1.9919
53	H	0.9561	-4.8191	-1.7534
54	H	-0.3556	-4.5890	-2.9133
55	H	0.4505	-3.1919	-2.1945
56	C	-1.1085	-3.2265	2.8158
57	H	-0.6631	-4.0685	3.3574
58	H	-0.3713	-2.4214	2.8120
59	H	-1.9712	-2.8932	3.3954
60	C	-4.6140	-4.9437	-0.3528
61	H	-5.1205	-5.2874	0.5509
62	H	-5.1865	-4.0917	-0.7371
63	H	-4.6792	-5.7351	-1.1017
64	C	-1.1870	2.7048	-1.1681
65	C	-0.3364	3.8132	-1.2123
66	C	-1.8274	2.2920	-2.3433
67	C	-0.1322	4.4954	-2.4066
68	H	0.1593	4.1592	-0.3108
69	C	-1.6225	2.9791	-3.5330
70	H	-2.4862	1.4279	-2.3298
71	C	-0.7725	4.0798	-3.5689
72	H	0.5239	5.3595	-2.4244
73	H	-2.1296	2.6486	-4.4338
74	H	-0.6165	4.6176	-4.4982
75	C	-2.1283	3.3862	2.4749
76	C	0.2380	3.1958	2.0688
77	C	-1.8696	4.2625	3.5242
78	H	-3.1537	3.1198	2.2422

79	C	0.4926	4.0802	3.1084
80	H	1.0692	2.7777	1.5058
81	C	-0.5634	4.6136	3.8412
82	H	-2.6963	4.6706	4.0958
83	H	1.5152	4.3506	3.3497
84	H	-0.3667	5.2982	4.6590
85	C	-3.1370	1.2925	0.4115
86	C	-4.0779	2.2081	-0.0774
87	C	-3.5900	0.1119	1.0065
88	C	-5.4374	1.9378	0.0145
89	H	-3.7456	3.1358	-0.5335
90	C	-4.9511	-0.1565	1.0948
91	H	-2.8747	-0.6065	1.3937
92	C	-5.8768	0.7532	0.5969
93	H	-6.1544	2.6548	-0.3708
94	H	-5.2867	-1.0816	1.5526
95	H	-6.9387	0.5417	0.6629
96	C	-1.0757	2.8439	1.7332
97	N	1.4792	0.8426	-1.0844
98	C	1.7996	1.3411	-3.3996
99	H	1.5063	1.1943	-4.4316
100	C	2.8122	2.2283	-3.0838
101	H	3.3249	2.7734	-3.8691
102	C	2.4670	1.6987	-0.7891
103	H	2.7235	1.7837	0.2642
104	C	3.1562	2.4168	-1.7490
105	H	3.9461	3.0961	-1.4511

Methylphenylimidazole

PhMeIm

1	C	-2.7581	-1.1441	-0.3935
2	C	-1.3754	-1.2325	-0.3734
3	C	-0.5934	-0.1395	0.0281
4	C	-1.2441	1.0334	0.4329
5	C	-2.6313	1.1185	0.4076
6	C	-3.3939	0.0351	-0.0117
7	H	-3.3459	-1.9992	-0.7113
8	H	-0.8671	-2.1465	-0.6595
9	H	-0.6720	1.8732	0.8107
10	H	-3.1175	2.0330	0.7316
11	H	-4.4764	0.1043	-0.0309
12	C	3.0218	0.1091	-0.0423
13	C	2.7904	-1.2222	0.1761
14	H	3.9231	0.6909	-0.1555
15	H	3.5192	-2.0094	0.3022
16	N	1.4535	-1.4762	0.2261
17	C	0.8565	-0.3074	0.0495
18	N	1.7814	0.6975	-0.1251
19	C	1.5629	2.0875	-0.4691
20	H	2.4200	2.4523	-1.0353
21	H	0.6711	2.1843	-1.0886
22	H	1.4429	2.7136	0.4198

Ir(H)$_2$(CHCl$_3$)(PhMeIm)(IMes)(PPh$_3$)

1	C	-3.2168	-3.0122	0.4545
2	C	-2.5456	-2.8963	-0.7696
3	C	-3.1847	-2.4894	-1.9443
4	C	-4.5368	-2.1584	-1.8605
5	C	-5.2329	-2.2047	-0.6542
6	C	-4.5566	-2.6371	0.4902
7	H	-5.0926	-2.6952	1.4348
8	C	-6.6717	-1.7978	-0.5735
9	H	-6.8178	-1.0092	0.1706
10	H	-7.0413	-1.4265	-1.5302

11	H	-7.3066	-2.6361	-0.2728
12	C	-2.5248	-3.5564	1.6672
13	H	-2.4527	-4.6486	1.6182
14	H	-1.5039	-3.1784	1.7680
15	H	-3.0715	-3.3091	2.5786
16	C	-0.7667	-4.5767	-1.0758
17	C	0.5805	-4.5920	-1.0025
18	H	-1.4845	-5.3539	-1.2792
19	H	1.2964	-5.3869	-1.1296
20	C	-2.4236	-2.3613	-3.2269
21	H	-3.0801	-2.0681	-4.0465
22	H	-1.6316	-1.6086	-3.1381
23	H	-1.9316	-3.2975	-3.5079
24	H	-5.0499	-1.8236	-2.7584
25	N	-1.1623	-3.2726	-0.8274
26	N	0.9791	-3.2990	-0.7093
27	C	4.4639	-2.2236	-1.3542
28	C	4.2716	-2.9340	0.9276
29	H	5.0692	-1.8526	-2.1785
30	C	3.1172	-2.4947	-1.5839
31	C	2.4917	-2.2846	-2.9273
32	H	3.2064	-1.8566	-3.6317
33	H	2.1271	-3.2253	-3.3529
34	H	1.6296	-1.6129	-2.8624
35	C	2.3626	-2.9687	-0.5045
36	C	2.9133	-3.1958	0.7589
37	C	2.0645	-3.6944	1.8876
38	H	1.6270	-4.6744	1.6703
39	H	2.6469	-3.7853	2.8054
40	H	1.2264	-3.0146	2.0798
41	H	4.7213	-3.1090	1.9012
42	C	5.0599	-2.4418	-0.1103
43	C	6.5132	-2.1452	0.0965
44	H	7.1404	-2.7151	-0.5948
45	H	6.7319	-1.0871	-0.0822
46	H	6.8340	-2.3848	1.1117
47	Ir	0.0466	-0.3941	-0.3461
48	H	1.5542	-0.7054	-0.4231
49	H	0.0245	-0.3200	-1.9358
50	C	-0.0924	-2.4534	-0.5910
51	P	0.4784	1.9211	-0.4476
52	C	0.5797	2.7520	1.1828
53	C	2.0270	2.3987	-1.3044
54	C	-0.7834	2.9232	-1.3291
55	C	-0.5437	2.6411	2.0093
56	C	1.7150	3.4003	1.6716
57	C	2.2635	3.7433	-1.6166
58	C	2.9904	1.4458	-1.6451
59	C	-1.3281	2.4158	-2.5149
60	C	-1.2080	4.1755	-0.8689
61	C	-0.5429	3.1734	3.2900
62	H	-1.4227	2.1108	1.6508
63	C	1.7211	3.9265	2.9610
64	H	2.6031	3.4858	1.0524
65	C	3.4421	4.1244	-2.2435
66	H	1.5189	4.4961	-1.3716
67	C	4.1720	1.8309	-2.2708
68	H	2.8174	0.3972	-1.4250
69	C	-2.2807	3.1407	-3.2188
70	H	-1.0120	1.4402	-2.8736
71	C	-2.1684	4.8957	-1.5751
72	H	-0.7944	4.5894	0.0457
73	C	0.5956	3.8176	3.7699
74	H	-1.4291	3.0812	3.9112
75	H	2.6097	4.4272	3.3307
76	C	4.4004	3.1686	-2.5700

77	H	3.6119	5.1689	-2.4820
78	H	4.9108	1.0784	-2.5298
79	C	-2.7073	4.3792	-2.7478
80	H	-2.6991	2.7332	-4.1331
81	H	-2.4919	5.8633	-1.2062
82	H	0.6033	4.2367	4.7710
83	H	5.3191	3.4680	-3.0632
84	H	-3.4600	4.9386	-3.2928
85	Cl	-2.7717	0.2126	-0.4802
86	C	-3.6752	1.6783	0.0548
87	H	-3.0118	2.5348	-0.0592
88	Cl	-5.0791	1.9005	-0.9776
89	C	2.3912	-0.2627	2.7997
90	C	3.1783	-0.8750	3.7872
91	C	3.0435	0.3702	1.7374
92	C	4.5643	-0.8037	3.7389
93	H	2.7019	-1.4270	4.5911
94	C	4.4281	0.4395	1.6873
95	H	2.4593	0.8301	0.9556
96	C	5.1951	-0.1312	2.6971
97	H	5.1526	-1.2789	4.5168
98	H	4.9040	0.9510	0.8554
99	H	6.2776	-0.0645	2.6671
100	N	0.0257	-0.4870	1.9456
101	C	-1.2012	-0.5199	2.5591
102	C	-1.0572	-0.3076	3.8958
103	H	-2.1069	-0.6907	1.9978
104	H	-1.7729	-0.2374	4.6986
105	C	0.9305	-0.2805	2.9116
106	N	0.2881	-0.1551	4.1165
107	C	0.8471	0.2554	5.3950
108	H	1.7734	0.8039	5.2334
109	H	0.1331	0.9128	5.8916
110	H	1.0461	-0.6025	6.0410
111	Cl	-4.1353	1.5370	1.7609

IrH_2(DCM)(PhMeIm)(IMes)(PPh_3)

1	C	-3.4670	3.1637	-0.4181
2	C	-2.6562	2.9792	0.7036
3	C	-3.1829	2.6438	1.9620
4	C	-4.5587	2.4561	2.0593
5	C	-5.4068	2.6097	0.9568
6	C	-4.8416	2.9620	-0.2650
7	H	-5.4861	3.0855	-1.1317
8	C	-6.8815	2.3937	1.0950
9	H	-7.3223	3.1050	1.7992
10	H	-7.3964	2.5046	0.1400
11	H	-7.1017	1.3932	1.4779
12	C	-2.9200	3.5705	-1.7521
13	H	-1.8406	3.7293	-1.7325
14	H	-3.1409	2.8109	-2.5087
15	H	-3.3838	4.4991	-2.0963
16	C	-0.8015	4.5747	0.8536
17	C	0.5460	4.5214	0.8500
18	H	-1.4875	5.3935	0.9930
19	H	1.2930	5.2850	0.9899
20	C	-2.2942	2.5221	3.1602
21	H	-2.8705	2.2779	4.0528
22	H	-1.5369	1.7459	3.0167
23	H	-1.7548	3.4551	3.3552
24	H	-4.9832	2.1956	3.0258
25	N	-1.2529	3.2831	0.6286
26	N	0.8930	3.2002	0.6231
27	C	4.0909	1.8741	1.9363
28	C	4.4238	2.5725	-0.3294
29	H	4.4837	1.4676	2.8657

30	C	2.7513	2.2598	1.8885
31	C	1.8745	2.1622	3.0997
32	H	1.5051	3.1482	3.4013
33	H	0.9932	1.5397	2.9184
34	H	2.4208	1.7409	3.9448
35	C	2.2645	2.7700	0.6769
36	C	3.0893	2.9624	-0.4362
37	C	2.5826	3.6081	-1.6885
38	H	1.5298	3.3883	-1.8730
39	H	2.6783	4.6981	-1.6345
40	H	3.1582	3.2830	-2.5584
41	H	5.0758	2.7033	-1.1903
42	C	4.9417	2.0168	0.8397
43	C	6.3694	1.5715	0.9024
44	H	6.5508	0.7432	0.2096
45	H	7.0533	2.3755	0.6185
46	H	6.6457	1.2348	1.9027
47	Ir	-0.1864	0.3405	0.2118
48	H	1.0987	0.4226	1.0569
49	H	-0.9663	0.2002	1.5913
50	C	-0.2162	2.4020	0.4751
51	P	-0.0187	-1.9997	0.3176
52	C	0.5626	-2.8975	-1.1725
53	C	0.9782	-2.6687	1.7036
54	C	-1.6504	-2.7796	0.6519
55	C	0.0601	-2.4872	-2.4150
56	C	1.4468	-3.9773	-1.1267
57	C	0.7862	-3.9926	2.1220
58	C	1.9296	-1.8894	2.3645
59	C	-2.2087	-2.5991	1.9247
60	C	-2.3881	-3.4560	-0.3219
61	C	0.4150	-3.1567	-3.5791
62	H	-0.6214	-1.6426	-2.4669
63	C	1.8003	-4.6463	-2.2948
64	H	1.8718	-4.2960	-0.1808
65	C	1.5357	-4.5216	3.1645
66	H	0.0383	-4.6106	1.6326
67	C	2.6788	-2.4195	3.4095
68	H	2.0853	-0.8586	2.0660
69	C	-3.4761	-3.0872	2.2144
70	H	-1.6441	-2.0732	2.6902
71	C	-3.6653	-3.9320	-0.0317
72	H	-1.9721	-3.6150	-1.3114
73	C	1.2833	-4.2439	-3.5201
74	H	0.0051	-2.8343	-4.5316
75	H	2.4869	-5.4852	-2.2431
76	C	2.4853	-3.7355	3.8109
77	H	1.3737	-5.5477	3.4768
78	H	3.4113	-1.7966	3.9142
79	C	-4.2119	-3.7489	1.2329
80	H	-3.8926	-2.9492	3.2067
81	H	-4.2309	-4.4499	-0.7990
82	H	1.5558	-4.7738	-4.4271
83	H	3.0657	-4.1475	4.6296
84	H	-5.2052	-4.1232	1.4563
85	Cl	-2.4909	0.0632	-1.3475
86	C	-3.8450	-0.2735	-0.1936
87	H	-3.6250	-1.2273	0.2748
88	H	-3.8542	0.5506	0.5157
89	Cl	-5.3887	-0.3768	-1.0227
90	C	2.9517	-0.6927	-1.8814
91	C	3.5492	-1.6948	-2.6586
92	C	3.3376	-0.5617	-0.5437
93	C	4.5147	-2.5295	-2.1143
94	H	3.2226	-1.8587	-3.6794
95	C	4.3026	-1.4007	-0.0027

96	H	2.8880	0.2166	0.0592
97	C	4.8979	-2.3848	-0.7850
98	H	4.9546	-3.3092	-2.7273
99	H	4.5950	-1.2772	1.0359
100	H	5.6528	-3.0382	-0.3608
101	N	0.8231	0.6318	-1.8346
102	C	0.1337	1.3660	-2.7681
103	C	0.8142	1.3754	-3.9473
104	H	-0.7993	1.8500	-2.5255
105	H	0.6162	1.8505	-4.8939
106	C	1.9272	0.1836	-2.4435
107	N	1.9506	0.6361	-3.7369
108	C	3.0291	0.5277	-4.7055
109	H	3.9805	0.4140	-4.1876
110	H	2.8840	-0.3254	-5.3721
111	H	3.0570	1.4390	-5.3028

$Ir(H)_2(PhMeIm)_2(IMes)(PPh_3)$

1	C	3.5027	2.9327	-0.4134
2	C	2.3575	2.7890	-1.2011
3	C	2.4187	2.3004	-2.5160
4	C	3.6553	1.8782	-2.9939
5	C	4.8198	1.9726	-2.2283
6	C	4.7227	2.5185	-0.9530
7	H	5.6171	2.6030	-0.3403
8	C	6.1371	1.5217	-2.7805
9	H	6.5528	2.2648	-3.4685
10	H	6.8734	1.3634	-1.9905
11	H	6.0388	0.5911	-3.3456
12	C	3.4568	3.5113	0.9675
13	H	3.6055	4.5969	0.9503
14	H	2.5032	3.3278	1.4649
15	H	4.2563	3.0949	1.5859
16	C	0.8145	4.5933	-0.5858
17	C	-0.4644	4.7047	-0.1743
18	H	1.5549	5.3381	-0.8255
19	H	-1.0844	5.5657	0.0136
20	C	1.2019	2.2324	-3.3834
21	H	1.4747	2.0295	-4.4199
22	H	0.5204	1.4448	-3.0448
23	H	0.6390	3.1707	-3.3602
24	H	3.7132	1.4806	-4.0046
25	N	1.0879	3.2395	-0.6906
26	N	-0.9516	3.4146	-0.0249
27	C	-4.4968	2.8003	-0.8547
28	C	-4.3168	2.9838	1.5209
29	H	-5.0997	2.6508	-1.7473
30	C	-3.1329	3.0240	-0.9994
31	C	-2.5014	3.0893	-2.3569
32	H	-2.0097	4.0532	-2.5250
33	H	-1.7288	2.3222	-2.4824
34	H	-3.2477	2.9552	-3.1414
35	C	-2.3614	3.1846	0.1613
36	C	-2.9464	3.2434	1.4285
37	C	-2.1947	3.6208	2.6695
38	H	-2.7704	4.3460	3.2497
39	H	-2.0331	2.7568	3.3223
40	H	-1.2247	4.0717	2.4540
41	H	-4.7809	2.9993	2.5050
42	C	-5.1039	2.7390	0.4007
43	C	-6.5703	2.4575	0.5188
44	H	-6.8754	2.3135	1.5567
45	H	-7.1671	3.2799	0.1133
46	H	-6.8497	1.5635	-0.0472
47	Ir	0.0034	0.3777	-0.2159
48	H	-0.8215	0.2843	-1.5521

49	H	1.3136	0.4786	-1.0690
50	C	0.0006	2.4688	-0.3414
51	P	0.1770	-1.9587	-0.2804
52	C	-0.2365	-2.7085	1.3344
53	C	-0.8230	-2.9201	-1.4922
54	C	1.8001	-2.6518	-0.7942
55	C	-1.5278	-3.1735	1.6030
56	C	0.6787	-2.6263	2.3881
57	C	-1.0098	-4.2973	-1.3155
58	C	-1.2160	-2.3375	-2.6964
59	C	2.3931	-3.7672	-0.2018
60	C	2.3515	-2.1317	-1.9716
61	C	-1.8908	-3.5490	2.8910
62	H	-2.2516	-3.2413	0.7966
63	C	0.3178	-3.0111	3.6739
64	H	1.6841	-2.2679	2.1947
65	C	-1.6077	-5.0617	-2.3079
66	H	-0.6734	-4.7787	-0.4018
67	C	-1.7992	-3.1088	-3.6966
68	H	-1.0571	-1.2751	-2.8530
69	C	3.5213	-4.3478	-0.7749
70	H	1.9689	-4.1993	0.6996
71	C	3.4625	-2.7270	-2.5516
72	H	1.8951	-1.2617	-2.4390
73	C	-0.9703	-3.4702	3.9308
74	H	-2.8972	-3.9110	3.0813
75	H	1.0470	-2.9572	4.4767
76	C	-2.0028	-4.4688	-3.5037
77	H	-1.7499	-6.1264	-2.1541
78	H	-2.1000	-2.6396	-4.6281
79	C	4.0521	-3.8376	-1.9532
80	H	3.9775	-5.2117	-0.3021
81	H	3.8712	-2.3221	-3.4728
82	H	-1.2528	-3.7705	4.9343
83	H	-2.4587	-5.0689	-4.2843
84	H	4.9206	-4.3047	-2.4061
85	C	-1.9555	0.0992	2.4292
86	C	-3.1544	-0.2765	2.9534
87	N	-1.9786	-0.0038	1.0622
88	C	0.7640	1.7756	2.5671
89	C	1.5748	2.0021	3.6368
90	N	1.2976	0.8083	1.7537
91	C	3.4693	-0.4678	1.8069
92	C	3.8624	-0.4059	0.4674
93	C	4.1131	-1.3726	2.6644
94	C	4.9206	-1.1832	0.0156
95	H	3.3410	0.2638	-0.2071
96	C	5.1459	-2.1733	2.1984
97	H	3.7864	-1.4612	3.6970
98	C	5.5647	-2.0647	0.8754
99	H	5.2320	-1.1070	-1.0203
100	H	5.6272	-2.8777	2.8682
101	H	6.3825	-2.6785	0.5121
102	C	-3.6910	-0.9329	-0.5540
103	C	-3.5178	-0.1684	-1.7091
104	C	-4.3860	-2.1476	-0.6597
105	C	-4.0719	-0.5670	-2.9170
106	H	-2.9438	0.7401	-1.6415
107	C	-4.9230	-2.5570	-1.8728
108	H	-4.4951	-2.7858	0.2127
109	C	-4.7859	-1.7571	-3.0021
110	H	-3.9351	0.0519	-3.7990
111	H	-5.4448	-3.5058	-1.9363
112	H	-5.2146	-2.0726	-3.9475
113	H	-3.5211	-0.3197	3.9661
114	H	-1.0670	0.3925	2.9603

115	H	-0.1663	2.2510	2.3174
116	H	1.5050	2.6807	4.4713
117	N	-3.9328	-0.6478	1.8897
118	N	2.6501	1.1659	3.4749
119	C	2.4567	0.4555	2.3162
120	C	-3.1860	-0.4913	0.7490
121	C	-5.3401	-0.9862	2.0050
122	H	-5.7983	-0.3436	2.7582
123	H	-5.8403	-0.8238	1.0524
124	H	-5.4766	-2.0294	2.3022
125	C	3.8318	1.1966	4.3205
126	H	3.7498	0.4923	5.1511
127	H	4.7143	0.9463	3.7335
128	H	3.9483	2.2026	4.7235

Ir(H)$_2$(PhMeIm)(IMes)(PPh$_3$)

1	C	-2.4374	-3.4295	0.4924
2	C	-1.5997	-3.2550	-0.6120
3	C	-2.0935	-2.9666	-1.8910
4	C	-3.4709	-2.8328	-2.0382
5	C	-4.3434	-2.9567	-0.9555
6	C	-3.8086	-3.2608	0.2950
7	H	-4.4777	-3.3845	1.1438
8	C	-5.8112	-2.7212	-1.1296
9	H	-6.0319	-1.6479	-1.1180
10	H	-6.1735	-3.1092	-2.0837
11	H	-6.3952	-3.1827	-0.3317
12	C	-1.8898	-3.8149	1.8311
13	H	-2.6011	-3.5904	2.6287
14	H	-1.6835	-4.8896	1.8780
15	H	-0.9515	-3.3025	2.0550
16	C	0.4851	-4.6005	-0.5526
17	C	1.8033	-4.3267	-0.4402
18	H	-0.0447	-5.5268	-0.7003
19	H	2.6699	-4.9665	-0.4590
20	C	-1.1597	-2.7404	-3.0401
21	H	-0.4397	-3.5562	-3.1553
22	H	-1.7060	-2.6365	-3.9782
23	H	-0.5661	-1.8291	-2.8888
24	H	-3.8728	-2.6005	-3.0216
25	N	-0.1805	-3.3891	-0.4481
26	N	1.9163	-2.9574	-0.2767
27	C	5.0519	-1.1752	-1.1075
28	C	4.9180	-1.4398	1.2712
29	H	5.5789	-0.8150	-1.9879
30	C	3.8366	-1.8376	-1.2790
31	C	3.2562	-2.0678	-2.6392
32	H	3.8669	-1.6004	-3.4121
33	H	3.1875	-3.1358	-2.8715
34	H	2.2426	-1.6624	-2.7129
35	C	3.1793	-2.2860	-0.1304
36	C	3.7036	-2.1108	1.1541
37	C	2.9929	-2.6463	2.3583
38	H	3.3124	-2.1332	3.2669
39	H	1.9079	-2.5510	2.2658
40	H	3.2004	-3.7127	2.4998
41	H	5.3381	-1.2856	2.2621
42	C	5.6079	-0.9665	0.1544
43	C	6.9266	-0.2743	0.3146
44	H	6.8951	0.4696	1.1150
45	H	7.7144	-0.9867	0.5783
46	H	7.2375	0.2255	-0.6045
47	Ir	0.3537	-0.3094	-0.1263
48	H	1.7807	-0.2542	0.4678
49	H	1.1526	-0.2036	-1.5004
50	C	0.6907	-2.3476	-0.2738

51	P	0.3862	2.0379	-0.1542
52	C	2.0221	2.7149	0.3195
53	C	0.0944	2.8266	-1.7791
54	C	-0.7925	2.8740	0.9680
55	C	2.1691	3.8603	1.1065
56	C	3.1639	2.0913	-0.1974
57	C	0.2654	4.2070	-1.9352
58	C	-0.2922	2.0613	-2.8798
59	C	-0.6614	2.6459	2.3446
60	C	-1.8677	3.6378	0.5092
61	C	3.4364	4.3660	1.3791
62	H	1.2946	4.3608	1.5113
63	C	4.4253	2.6074	0.0677
64	H	3.0624	1.1996	-0.8107
65	C	0.0370	4.8079	-3.1654
66	H	0.5828	4.8126	-1.0901
67	C	-0.5227	2.6648	-4.1122
68	H	-0.3928	0.9855	-2.7681
69	C	-1.5795	3.1778	3.2398
70	H	0.1696	2.0500	2.7140
71	C	-2.7832	4.1753	1.4095
72	H	-1.9984	3.8104	-0.5546
73	C	4.5652	3.7430	0.8597
74	H	3.5389	5.2530	1.9953
75	H	5.3005	2.1196	-0.3488
76	C	-0.3613	4.0375	-4.2551
77	H	0.1750	5.8780	-3.2771
78	H	-0.8184	2.0603	-4.9636
79	C	-2.6435	3.9474	2.7731
80	H	-1.4583	3.0005	4.3041
81	H	-3.6101	4.7729	1.0391
82	H	5.5515	4.1435	1.0692
83	H	-0.5354	4.5082	-5.2168
84	H	-3.3549	4.3741	3.4729
85	C	-0.4292	-0.8201	3.0011
86	C	-1.4977	-0.8342	3.8491
87	C	-2.9539	0.2635	0.7127
88	C	-4.0954	1.0565	0.8942
89	C	-2.5103	0.0396	-0.6001
90	C	-4.7685	1.5922	-0.1952
91	H	-4.4287	1.3138	1.8917
92	C	-3.1818	0.5792	-1.6875
93	H	-1.7095	-0.6849	-0.7969
94	C	-4.3164	1.3586	-1.4897
95	H	-5.6408	2.2152	-0.0283
96	H	-2.8293	0.3713	-2.6926
97	H	-4.8435	1.7800	-2.3385
98	N	-0.8493	-0.4693	1.7486
99	H	-1.5873	-1.0763	4.8955
100	H	0.6080	-1.0264	3.2109
101	C	-2.1644	-0.2433	1.8244
102	N	-2.5942	-0.4815	3.1001
103	C	-3.9560	-0.4911	3.6042
104	H	-4.6411	-0.8053	2.8169
105	H	-4.0187	-1.2019	4.4277
106	H	-4.2542	0.4949	3.9693

$Ir(H)_2(PhMeIm)(IMes)(PPh_3)$ C-C Rotation TS2 *Imaginary Frequency: -33.3 cm^{-1}*

1	C	-2.2082	-4.2392	0.5145
2	C	-1.4081	-3.4868	-0.3556
3	C	-1.9606	-2.7816	-1.4350
4	C	-3.3254	-2.9167	-1.6713
5	C	-4.1569	-3.6684	-0.8398
6	C	-3.5785	-4.3016	0.2557
7	H	-4.2080	-4.8743	0.9327
8	C	-5.6248	-3.7690	-1.1176

9	H	-5.8162	-4.3032	-2.0528
10	H	-6.1521	-4.2991	-0.3233
11	H	-6.0824	-2.7803	-1.2243
12	C	-1.6508	-4.9508	1.7111
13	H	-2.4132	-5.0430	2.4868
14	H	-1.3240	-5.9677	1.4708
15	H	-0.7902	-4.4320	2.1395
16	C	0.7716	-4.6376	-0.1183
17	C	2.0482	-4.2612	0.1097
18	H	0.3339	-5.6081	-0.2769
19	H	2.9529	-4.8370	0.2111
20	C	-1.1490	-1.8656	-2.2972
21	H	-0.1436	-2.2349	-2.5039
22	H	-1.6477	-1.6742	-3.2503
23	H	-1.0566	-0.8557	-1.8391
24	H	-3.7539	-2.4067	-2.5315
25	N	0.0096	-3.4758	-0.1480
26	N	2.0490	-2.8815	0.2073
27	C	5.1573	-0.9853	-0.4470
28	C	4.6647	-0.8713	1.8938
29	H	5.7864	-0.7170	-1.2920
30	C	4.0328	-1.7749	-0.6747
31	C	3.6773	-2.2420	-2.0518
32	H	4.3103	-1.7662	-2.8020
33	H	3.7966	-3.3250	-2.1613
34	H	2.6343	-2.0118	-2.2920
35	C	3.2338	-2.0949	0.4262
36	C	3.5304	-1.6611	1.7213
37	C	2.6433	-1.9919	2.8825
38	H	3.1850	-1.9123	3.8260
39	H	1.7955	-1.2980	2.9363
40	H	2.2279	-3.0014	2.8152
41	H	4.9111	-0.5219	2.8936
42	C	5.4895	-0.5210	0.8245
43	C	6.7140	0.3120	1.0452
44	H	6.5111	1.1558	1.7094
45	H	7.5113	-0.2757	1.5113
46	H	7.1063	0.7068	0.1063
47	Ir	0.3639	-0.3659	-0.1302
48	H	1.3904	-0.0746	0.9957
49	H	1.5995	-0.3319	-1.1196
50	C	0.7950	-2.3678	0.0373
51	P	0.5054	1.9563	-0.3839
52	C	2.2807	2.4285	-0.4300
53	C	-0.1675	2.7349	-1.8952
54	C	-0.1509	2.9671	0.9940
55	C	2.8878	3.1534	0.5961
56	C	3.0565	2.0087	-1.5170
57	C	0.4335	3.8480	-2.4945
58	C	-1.3332	2.2088	-2.4562
59	C	-0.1529	2.4166	2.2806
60	C	-0.6522	4.2572	0.8046
61	C	4.2469	3.4531	0.5358
62	H	2.3035	3.4932	1.4453
63	C	4.4062	2.3230	-1.5817
64	H	2.5977	1.4364	-2.3194
65	C	-0.1253	4.4176	-3.6324
66	H	1.3433	4.2657	-2.0732
67	C	-1.8915	2.7814	-3.5931
68	H	-1.8137	1.3519	-1.9904
69	C	-0.6539	3.1416	3.3558
70	H	0.2275	1.4100	2.4334
71	C	-1.1608	4.9773	1.8805
72	H	-0.6641	4.6952	-0.1891
73	C	5.0062	3.0444	-0.5525
74	H	4.7072	4.0179	1.3400

75	H	4.9933	2.0017	-2.4365
76	C	-1.2863	3.8846	-4.1847
77	H	0.3495	5.2784	-4.0913
78	H	-2.7987	2.3623	-4.0171
79	C	-1.1659	4.4209	3.1559
80	H	-0.6480	2.7069	4.3501
81	H	-1.5531	5.9767	1.7223
82	H	6.0624	3.2883	-0.6019
83	H	-1.7160	4.3284	-5.0765
84	H	-1.5638	4.9845	3.9932
85	C	-1.3030	-1.4590	2.3651
86	C	-2.5171	-1.6023	2.9651
87	C	-3.3122	0.7611	0.3388
88	C	-4.0296	0.3214	-0.7778
89	C	-3.2798	2.1276	0.6326
90	C	-4.6708	1.2340	-1.6073
91	H	-4.0838	-0.7413	-0.9849
92	C	-3.9174	3.0381	-0.2028
93	H	-2.7664	2.4733	1.5236
94	C	-4.6084	2.5949	-1.3249
95	H	-5.2241	0.8815	-2.4722
96	H	-3.8765	4.0970	0.0307
97	H	-5.1042	3.3087	-1.9743
98	N	-1.4068	-0.5965	1.2987
99	H	-2.8432	-2.1820	3.8133
100	H	-0.3614	-1.9215	2.6172
101	C	-2.6803	-0.2066	1.2559
102	N	-3.3841	-0.8072	2.2571
103	C	-4.8132	-0.6961	2.4860
104	H	-5.3550	-1.4678	1.9322
105	H	-5.0193	-0.8083	3.5494
106	H	-5.1617	0.2824	2.1581

Ir(H)$_2$(PhMeIm)(IMes)(PPh$_3$) C-H Activation TS1 ***Imaginary Frequency: -702.8 cm^{-1}***

1	Ir	0.3731	-0.0324	0.1897
2	H	1.5526	0.9691	-0.3142
3	P	-1.0843	1.8365	0.1268
4	C	3.9972	-2.2490	0.8566
5	C	3.2272	-3.2825	0.4526
6	H	5.0250	-2.2036	1.1758
7	H	3.4367	-4.3333	0.3415
8	N	3.2044	-1.1158	0.8058
9	N	1.9776	-2.7581	0.1644
10	C	1.9439	-1.4109	0.3716
11	H	0.8528	0.5723	1.5897
12	C	-1.9186	-1.6962	-0.9669
13	C	-1.4873	-1.2497	0.3186
14	C	-2.3377	-1.5063	1.4006
15	C	-3.5380	-2.1959	1.2591
16	C	-3.9325	-2.6473	0.0067
17	C	-3.1314	-2.3865	-1.0939
18	H	-0.2564	-0.9279	1.3575
19	H	-2.0562	-1.1652	2.3929
20	H	-4.1590	-2.3779	2.1302
21	H	-4.8649	-3.1871	-0.1185
22	H	-3.4710	-2.7171	-2.0665
23	C	0.8801	-3.5726	-0.2811
24	C	-0.0162	-4.0737	0.6722
25	C	0.7881	-3.8881	-1.6374
26	C	-1.0649	-4.8657	0.2172
27	C	-0.2838	-4.6863	-2.0419
28	C	-1.2232	-5.1734	-1.1359
29	H	-1.7844	-5.2461	0.9382
30	H	-0.3728	-4.9449	-3.0952
31	C	3.6783	0.1816	1.2091
32	C	3.5423	0.5538	2.5497

33	C	4.2597	1.0118	0.2501
34	C	3.9996	1.8171	2.9155
35	C	4.7006	2.2675	0.6667
36	C	4.5817	2.6873	1.9911
37	H	3.9025	2.1285	3.9526
38	H	5.1500	2.9339	-0.0654
39	C	4.3895	0.5658	-1.1726
40	H	5.0815	-0.2771	-1.2712
41	H	3.4254	0.2295	-1.5680
42	H	4.7580	1.3718	-1.8077
43	C	5.0856	4.0296	2.4251
44	H	5.9952	3.9323	3.0257
45	H	5.3270	4.6673	1.5730
46	H	4.3547	4.5527	3.0474
47	C	2.9209	-0.3735	3.5478
48	H	3.4712	-1.3167	3.6245
49	H	2.8944	0.0768	4.5404
50	H	1.8948	-0.6323	3.2656
51	C	1.8193	-3.4229	-2.6185
52	H	2.7030	-4.0700	-2.5954
53	H	1.4326	-3.4441	-3.6389
54	H	2.1639	-2.4080	-2.4067
55	C	0.1671	-3.7948	2.1317
56	H	1.0183	-4.3535	2.5363
57	H	0.3694	-2.7384	2.3298
58	H	-0.7175	-4.0835	2.7004
59	C	-2.3819	-6.0050	-1.5917
60	H	-2.3708	-6.9951	-1.1277
61	H	-3.3333	-5.5408	-1.3119
62	H	-2.3800	-6.1485	-2.6738
63	C	-2.2102	1.7581	-1.3057
64	C	-1.7617	2.1897	-2.5593
65	C	-3.4417	1.1013	-1.2136
66	C	-2.5400	1.9899	-3.6926
67	H	-0.7990	2.6864	-2.6466
68	C	-4.2185	0.9039	-2.3499
69	H	-3.7914	0.7338	-0.2528
70	C	-3.7719	1.3498	-3.5896
71	H	-2.1854	2.3398	-4.6566
72	H	-5.1767	0.4012	-2.2615
73	H	-4.3849	1.2052	-4.4736
74	C	-0.9522	4.5738	-0.4959
75	C	1.0421	3.6328	0.4834
76	C	-0.3503	5.8251	-0.5190
77	H	-1.9645	4.4605	-0.8730
78	C	1.6374	4.8895	0.4640
79	H	1.5920	2.7822	0.8734
80	C	0.9463	5.9850	-0.0398
81	H	-0.8955	6.6769	-0.9113
82	H	2.6482	5.0070	0.8405
83	H	1.4145	6.9634	-0.0590
84	C	-2.1687	2.0677	1.5821
85	C	-3.3447	2.8207	1.4903
86	C	-1.7715	1.5811	2.8298
87	C	-4.1162	3.0595	2.6202
88	H	-3.6638	3.2193	0.5319
89	C	-2.5426	1.8272	3.9603
90	H	-0.8513	1.0110	2.9144
91	C	-3.7181	2.5615	3.8567
92	H	-5.0286	3.6399	2.5348
93	H	-2.2228	1.4449	4.9240
94	H	-4.3210	2.7511	4.7383
95	C	-0.2565	3.4631	-0.0039
96	N	-0.0137	-0.5574	-1.8800
97	C	0.5402	-0.2560	-3.0892
98	H	1.4237	0.3555	-3.1774

99	C	-0.2120	-0.8383	-4.0665
100	H	-0.1181	-0.8461	-5.1399
101	C	-1.1013	-1.3041	-2.0935
102	N	-1.2428	-1.5004	-3.4372
103	C	-2.2613	-2.2543	-4.1431
104	H	-2.2944	-3.2854	-3.7848
105	H	-3.2428	-1.7914	-4.0214
106	H	-2.0094	-2.2616	-5.2022

Ir(H)(H_2)(PhMeIm)(IMes)(PPh_3)

1	C	-3.6447	-1.7772	-1.6110
2	C	-2.3593	-2.2237	-1.9474
3	C	-1.8536	-2.1382	-3.2464
4	C	-2.6657	-1.5416	-4.2160
5	C	-3.9515	-1.0916	-3.9289
6	C	-4.4243	-1.2255	-2.6206
7	H	-5.4228	-0.8714	-2.3756
8	C	-4.8169	-0.4859	-4.9907
9	H	-5.1826	0.5017	-4.6955
10	H	-4.2841	-0.3780	-5.9361
11	H	-5.7011	-1.1020	-5.1777
12	C	-4.1468	-1.8794	-0.2041
13	H	-5.0769	-1.3231	-0.0790
14	H	-4.3427	-2.9175	0.0844
15	H	-3.4139	-1.4906	0.5104
16	C	-1.7535	-4.2154	-0.6312
17	C	-0.9041	-4.4987	0.3789
18	H	-2.4548	-4.8273	-1.1735
19	H	-0.7037	-5.4133	0.9115
20	C	-0.5232	-2.7106	-3.6354
21	H	0.0530	-2.0034	-4.2385
22	H	0.0838	-2.9974	-2.7757
23	H	-0.6578	-3.6062	-4.2499
24	H	-2.2881	-1.4596	-5.2325
25	N	-1.5868	-2.8718	-0.9232
26	N	-0.2374	-3.3251	0.6836
27	C	3.0013	-3.3140	2.4863
28	C	1.2186	-3.1051	4.0741
29	H	4.0632	-3.3971	2.2668
30	C	2.0925	-3.3913	1.4317
31	C	2.5587	-3.6616	0.0340
32	H	2.4330	-4.7203	-0.2193
33	H	2.0019	-3.0866	-0.7087
34	H	3.6183	-3.4270	-0.0800
35	C	0.7354	-3.2621	1.7420
36	C	0.2707	-3.1372	3.0542
37	C	-1.1901	-2.9890	3.3443
38	H	-1.6077	-2.1279	2.8101
39	H	-1.7651	-3.8668	3.0325
40	H	-1.3647	-2.8366	4.4097
41	H	0.8767	-2.9969	5.1004
42	C	2.5864	-3.1665	3.8107
43	C	3.5858	-3.0443	4.9191
44	H	3.8336	-1.9931	5.1048
45	H	3.2028	-3.4515	5.8566
46	H	4.5203	-3.5559	4.6822
47	Ir	0.1505	-0.3577	-0.2523
48	C	-0.6480	-2.2949	-0.1142
49	P	1.2728	1.7302	-0.3867
50	C	0.2538	3.1345	0.2054
51	C	2.8154	1.8932	0.5780
52	C	1.7321	2.1760	-2.1029
53	C	0.1720	3.3810	1.5820
54	C	-0.5255	3.8979	-0.6674
55	C	3.2996	3.1631	0.9197
56	C	3.5567	0.7667	0.9434

57	C	2.9882	2.6883	-2.4359
58	C	0.7899	1.9798	-3.1214
59	C	-0.6662	4.3758	2.0690
60	H	0.7642	2.7916	2.2765
61	C	-1.3666	4.8906	-0.1750
62	H	-0.4702	3.7314	-1.7383
63	C	4.4919	3.2973	1.6192
64	H	2.7396	4.0504	0.6391
65	C	4.7486	0.9046	1.6461
66	H	3.1954	-0.2231	0.6843
67	C	3.2912	3.0005	-3.7577
68	H	3.7385	2.8359	-1.6665
69	C	1.0911	2.3038	-4.4375
70	H	-0.1896	1.5737	-2.8787
71	C	-1.4379	5.1332	1.1921
72	H	-0.7117	4.5588	3.1381
73	H	-1.9560	5.4844	-0.8662
74	C	5.2170	2.1679	1.9870
75	H	4.8542	4.2862	1.8786
76	H	5.3098	0.0199	1.9291
77	C	2.3460	2.8131	-4.7584
78	H	4.2730	3.3907	-4.0034
79	H	0.3479	2.1549	-5.2140
80	H	-2.0848	5.9169	1.5733
81	H	6.1453	2.2745	2.5380
82	H	2.5863	3.0589	-5.7872
83	C	-0.2403	0.0998	1.7211
84	C	-1.4780	0.7433	2.0013
85	C	0.6370	-0.1007	2.7880
86	C	-1.8270	1.0754	3.3179
87	C	0.2985	0.2630	4.0889
88	H	1.6021	-0.5650	2.6048
89	C	-0.9426	0.8312	4.3590
90	H	-2.7838	1.5305	3.5416
91	H	1.0045	0.0872	4.8959
92	H	-1.2183	1.0932	5.3747
93	C	-2.6391	1.2083	-1.3231
94	C	-3.6071	1.9396	-0.6988
95	H	-2.5133	1.0056	-2.3757
96	H	-4.4430	2.5047	-1.0772
97	N	-1.8081	0.6801	-0.3790
98	H	0.8888	-0.8381	-1.7988
99	H	0.1119	-0.5625	-2.0302
100	H	1.5176	-1.0423	0.1673
101	C	-4.1254	2.5537	1.6667
102	H	-4.8687	3.1731	1.1676
103	H	-4.6413	1.8494	2.3224
104	H	-3.4770	3.1994	2.2616
105	N	-3.3540	1.8587	0.6536
106	C	-2.2451	1.0838	0.8211

2-Phenylpyrimidine

2PhPm

1	C	-0.7628	0.0000	0.0003
2	C	-2.7079	1.1772	0.0005
3	C	-3.4444	-0.0000	0.0003
4	C	-2.7079	-1.1772	0.0001
5	H	-3.2021	2.1478	0.0008
6	H	-4.5276	-0.0000	0.0004
7	H	-3.2021	-2.1479	-0.0001
8	C	0.7128	0.0000	0.0002
9	C	1.4216	-1.2067	-0.0000
10	C	1.4216	1.2067	0.0005
11	C	2.8090	-1.2043	-0.0000

12	H	0.8628	-2.1353	-0.0002
13	C	2.8089	1.2043	0.0004
14	H	0.8628	2.1353	0.0007
15	C	3.5071	0.0000	0.0002
16	H	3.3498	-2.1451	-0.0002
17	H	3.3498	2.1452	0.0006
18	H	4.5924	0.0000	0.0002
19	N	-1.3790	-1.1947	0.0001
20	N	-1.3790	1.1947	0.0005

$Ir(H)_2(CHCl_3)(PhPm)(IMes)(PPh_3)$

1	C	2.4966	-2.8988	-2.0018
2	C	1.8365	-3.3210	-0.8393
3	C	2.5227	-3.6611	0.3305
4	C	3.9107	-3.5229	0.3284
5	C	4.6078	-3.0720	-0.7908
6	C	3.8832	-2.7726	-1.9460
7	H	4.4136	-2.4404	-2.8358
8	C	6.0926	-2.8919	-0.7396
9	H	6.5953	-3.7977	-0.3912
10	H	6.5040	-2.6341	-1.7166
11	H	6.3626	-2.0921	-0.0422
12	C	1.7408	-2.6111	-3.2635
13	H	0.9835	-1.8343	-3.1186
14	H	2.4138	-2.2806	-4.0558
15	H	1.2136	-3.4999	-3.6255
16	C	-0.2272	-4.6389	-1.2032
17	C	-1.5552	-4.3889	-1.1906
18	H	0.3297	-5.5384	-1.4069
19	H	-2.4055	-5.0225	-1.3803
20	C	1.8212	-4.1973	1.5413
21	H	2.2543	-3.7929	2.4597
22	H	0.7527	-3.9759	1.5414
23	H	1.9235	-5.2862	1.5979
24	H	4.4606	-3.7687	1.2338
25	N	0.4053	-3.4512	-0.8805
26	N	-1.7056	-3.0542	-0.8569
27	C	-5.0002	-1.8862	0.3899
28	C	-4.6424	-1.0135	-1.8100
29	H	-5.6104	-1.9663	1.2863
30	C	-3.7687	-2.5443	0.3579
31	C	-3.3039	-3.2924	1.5712
32	H	-4.0615	-4.0043	1.9080
33	H	-2.3783	-3.8470	1.4061
34	H	-3.1395	-2.5984	2.4051
35	C	-2.9870	-2.4054	-0.7927
36	C	-3.4273	-1.6810	-1.9135
37	C	-2.6695	-1.6836	-3.2060
38	H	-1.5903	-1.7510	-3.0666
39	H	-2.9700	-2.5409	-3.8188
40	H	-2.8815	-0.7857	-3.7894
41	H	-4.9897	-0.4295	-2.6596
42	C	-5.4405	-1.0948	-0.6648
43	C	-6.7362	-0.3492	-0.5901
44	H	-7.4426	-0.7004	-1.3478
45	H	-7.2117	-0.4594	0.3852
46	H	-6.5860	0.7193	-0.7711
47	Ir	-0.0541	-0.4269	-0.3771
48	H	1.1789	-0.7602	-1.2352
49	H	-0.7833	-0.1844	-1.7511
50	C	-0.4954	-2.4437	-0.6578
51	P	0.4271	1.8602	-0.5893
52	C	-1.1032	2.8771	-0.7519
53	C	1.2817	2.7178	0.7800
54	C	1.3790	2.3067	-2.0876
55	C	-1.2358	4.1309	-0.1408

56	C	-2.1704	2.3853	-1.5143
57	C	0.7856	2.4968	2.0696
58	C	2.3324	3.6178	0.6026
59	C	2.2961	1.4065	-2.6366
60	C	1.2338	3.5674	-2.6776
61	C	-2.4096	4.8663	-0.2844
62	H	-0.4220	4.5388	0.4512
63	C	-3.3380	3.1236	-1.6572
64	H	-2.0903	1.4094	-1.9832
65	C	1.2994	3.1858	3.1584
66	H	0.0012	1.7587	2.2215
67	C	2.8552	4.3018	1.6975
68	H	2.7430	3.7887	-0.3879
69	C	3.0580	1.7603	-3.7436
70	H	2.4159	0.4228	-2.1963
71	C	1.9931	3.9171	-3.7879
72	H	0.5205	4.2787	-2.2717
73	C	-3.4622	4.3650	-1.0411
74	H	-2.4968	5.8348	0.1965
75	H	-4.1569	2.7227	-2.2465
76	C	2.3350	4.0983	2.9702
77	H	0.8996	3.0036	4.1512
78	H	3.6733	4.9996	1.5513
79	C	2.9077	3.0156	-4.3222
80	H	3.7665	1.0493	-4.1579
81	H	1.8668	4.8958	-4.2382
82	H	-4.3774	4.9376	-1.1466
83	H	2.7417	4.6411	3.8170
84	H	3.4972	3.2897	-5.1906
85	Cl	-2.4660	0.1633	0.9683
86	C	-2.7251	1.6145	2.0194
87	H	-2.0833	2.4069	1.6352
88	Cl	-4.3987	2.1236	1.8940
89	C	-0.0444	-1.5507	2.4992
90	C	1.9620	-0.4391	2.4566
91	C	0.0231	-1.6880	3.8706
92	H	-0.8792	-1.9615	1.9397
93	C	1.1094	-1.0810	4.4870
94	H	-0.7465	-2.2132	4.4226
95	H	1.2221	-1.0792	5.5696
96	C	3.1115	0.1528	1.7540
97	C	3.9566	1.0264	2.4534
98	C	3.4213	-0.1721	0.4306
99	C	5.0579	1.5890	1.8286
100	H	3.7278	1.2584	3.4864
101	C	4.5357	0.3823	-0.1868
102	H	2.8099	-0.8925	-0.0969
103	C	5.3497	1.2734	0.5040
104	H	5.6921	2.2782	2.3762
105	H	4.7757	0.1045	-1.2090
106	H	6.2158	1.7100	0.0175
107	N	0.8784	-0.8974	1.7704
108	N	2.0724	-0.4912	3.7930
109	Cl	-2.2609	1.2570	3.6883

$Ir(H)_2(DCM)(PhPm)(IMes)(PPh_3)$

1	C	-1.8863	-3.2291	1.6008
2	C	-1.2946	-3.4221	0.3438
3	C	-2.0465	-3.7064	-0.7999
4	C	-3.4353	-3.7351	-0.6702
5	C	-4.0703	-3.5009	0.5474
6	C	-3.2776	-3.2573	1.6705
7	H	-3.7554	-3.1000	2.6352
8	C	-5.5642	-3.4965	0.6366
9	H	-5.9089	-3.3560	1.6622

10	H	-5.9879	-2.6915	0.0276
11	H	-5.9906	-4.4318	0.2648
12	C	-1.0534	-3.0356	2.8319
13	H	-0.4517	-3.9254	3.0465
14	H	-0.3536	-2.2009	2.7305
15	H	-1.6822	-2.8464	3.7032
16	C	0.9004	-4.5494	0.3402
17	C	2.1943	-4.1661	0.3130
18	H	0.4431	-5.5226	0.4067
19	H	3.1112	-4.7304	0.3514
20	C	-1.4129	-4.0201	-2.1215
21	H	-1.9290	-3.5089	-2.9386
22	H	-0.3575	-3.7466	-2.1601
23	H	-1.4761	-5.0923	-2.3344
24	H	-4.0369	-3.9389	-1.5530
25	N	0.1411	-3.3957	0.2611
26	N	2.1991	-2.7832	0.2160
27	C	5.4296	-1.2407	-0.7647
28	C	5.1482	-1.0405	1.6058
29	H	6.0248	-1.0815	-1.6602
30	C	4.1860	-1.8659	-0.8894
31	C	3.7122	-2.3109	-2.2402
32	H	2.8593	-2.9907	-2.1863
33	H	3.4212	-1.4494	-2.8519
34	H	4.5104	-2.8234	-2.7819
35	C	3.4330	-2.0469	0.2735
36	C	3.9040	-1.6617	1.5393
37	C	3.1080	-1.9412	2.7760
38	H	2.9009	-3.0108	2.8865
39	H	3.6412	-1.6116	3.6680
40	H	2.1380	-1.4363	2.7506
41	H	5.5284	-0.7361	2.5779
42	C	5.9277	-0.8200	0.4651
43	C	7.2610	-0.1483	0.5719
44	H	7.7198	-0.0047	-0.4069
45	H	7.1740	0.8331	1.0465
46	H	7.9521	-0.7345	1.1843
47	Ir	0.2327	-0.3144	0.1762
48	H	-0.8609	-0.8880	1.0951
49	H	1.0690	-0.0640	1.4902
50	C	0.9288	-2.2741	0.1802
51	P	-0.6288	1.8300	0.6193
52	C	0.7228	3.0133	1.0150
53	C	-1.5721	2.6940	-0.6903
54	C	-1.6632	1.9646	2.1262
55	C	1.1058	4.0539	0.1663
56	C	1.4426	2.8018	2.1991
57	C	-1.0519	2.6774	-1.9916
58	C	-2.7702	3.3696	-0.4500
59	C	-1.8305	3.2134	2.7399
60	C	-2.3000	0.8538	2.6827
61	C	2.1986	4.8573	0.4880
62	H	0.5547	4.2445	-0.7487
63	C	2.5224	3.6124	2.5228
64	H	1.1499	1.9987	2.8707
65	C	-1.7027	3.3422	-3.0224
66	H	-0.1259	2.1445	-2.1953
67	C	-3.4203	4.0329	-1.4860
68	H	-3.2073	3.3712	0.5429
69	C	-2.6206	3.3439	3.8744
70	H	-1.3344	4.0885	2.3288
71	C	-3.0903	0.9853	3.8194
72	H	-2.1795	-0.1235	2.2286
73	C	2.9092	4.6385	1.6622
74	H	2.4891	5.6593	-0.1823
75	H	3.0645	3.4439	3.4475

76	C	-2.8881	4.0265	-2.7695
77	H	-1.2853	3.3239	-4.0240
78	H	-4.3526	4.5511	-1.2865
79	C	-3.2529	2.2290	4.4172
80	H	-2.7391	4.3173	4.3381
81	H	-3.5754	0.1094	4.2397
82	H	3.7569	5.2677	1.9111
83	H	-3.3980	4.5466	-3.5735
84	H	-3.8653	2.3310	5.3068
85	Cl	2.2043	0.8650	-1.4678
86	C	3.4847	1.5070	-0.3569
87	H	3.0288	2.3157	0.2042
88	H	3.7863	0.6734	0.2733
89	Cl	4.8613	2.1262	-1.2491
90	C	0.1581	-1.1586	-2.7913
91	C	-1.9706	-0.3388	-2.5586
92	C	0.0492	-1.1525	-4.1666
93	H	1.0626	-1.5155	-2.3083
94	C	-1.1421	-0.6508	-4.6764
95	H	0.8554	-1.5056	-4.7975
96	H	-1.3087	-0.5578	-5.7479
97	C	-3.1511	0.0270	-1.7610
98	C	-4.1673	0.7828	-2.3638
99	C	-3.3192	-0.3995	-0.4404
100	C	-5.3057	1.1248	-1.6515
101	H	-4.0421	1.0989	-3.3921
102	C	-4.4670	-0.0634	0.2657
103	H	-2.5651	-1.0284	0.0154
104	C	-5.4589	0.7068	-0.3323
105	H	-6.0768	1.7226	-2.1261
106	H	-4.5928	-0.4183	1.2842
107	H	-6.3530	0.9707	0.2233
108	N	-0.8054	-0.7153	-1.9627
109	N	-2.1406	-0.2802	-3.8898

$Ir(H)_2(PhPm)_2(IMes)(PPh_3)$

1	C	0.5073	3.7271	2.0701
2	C	1.4878	3.4178	1.1126
3	C	2.8417	3.3229	1.4360
4	C	3.1963	3.4840	2.7795
5	C	2.2506	3.7136	3.7735
6	C	0.9091	3.8411	3.3967
7	H	0.1601	4.0661	4.1528
8	C	2.6491	3.8414	5.2116
9	H	2.5017	4.8622	5.5761
10	H	2.0437	3.1924	5.8520
11	H	3.6986	3.5862	5.3664
12	C	-0.9080	3.9887	1.6594
13	H	-1.5555	4.1134	2.5280
14	H	-0.9739	4.9022	1.0577
15	H	-1.3138	3.1840	1.0411
16	C	1.1278	4.2940	-1.1662
17	C	0.4713	3.8747	-2.2683
18	H	1.6347	5.2172	-0.9395
19	H	0.2843	4.3553	-3.2142
20	C	3.8970	3.0018	0.4233
21	H	4.2823	1.9863	0.5713
22	H	3.5340	3.0660	-0.6028
23	H	4.7509	3.6762	0.5218
24	H	4.2454	3.3981	3.0534
25	N	1.0531	3.2604	-0.2442
26	N	0.0051	2.6006	-1.9993
27	C	-1.1629	0.4000	-4.7556
28	C	-3.0596	1.3975	-3.6847
29	H	-0.7453	-0.2495	-5.5217
30	C	-0.2909	1.0794	-3.9071

31	C	1.1905	0.9741	-4.0827
32	H	1.7161	1.0265	-3.1283
33	H	1.4700	0.0403	-4.5755
34	H	1.5713	1.7929	-4.7042
35	C	-0.8494	1.8943	-2.9190
36	C	-2.2284	2.0933	-2.8068
37	C	-2.7803	3.0246	-1.7760
38	H	-2.2934	4.0043	-1.8120
39	H	-3.8511	3.1744	-1.9094
40	H	-2.6218	2.6299	-0.7666
41	H	-4.1356	1.5373	-3.6062
42	C	-2.5479	0.5419	-4.6602
43	C	-3.4516	-0.1854	-5.6083
44	H	-3.3480	0.2016	-6.6265
45	H	-3.2125	-1.2516	-5.6527
46	H	-4.5015	-0.0832	-5.3277
47	Ir	0.0509	0.2232	-0.0961
48	H	-1.3240	0.3745	-0.8159
49	H	0.7690	-0.0176	-1.4552
50	C	0.3551	2.1870	-0.7339
51	P	-0.3634	-2.0955	-0.1075
52	C	-0.2817	-2.8893	1.5337
53	C	-1.9784	-2.6251	-0.8154
54	C	0.7116	-3.1184	-1.1941
55	C	-1.4125	-2.9692	2.3542
56	C	0.9704	-3.1986	2.0811
57	C	-2.5250	-3.8750	-0.5018
58	C	-2.5863	-1.8526	-1.8089
59	C	1.0445	-4.4406	-0.8866
60	C	1.0308	-2.6176	-2.4604
61	C	-1.2946	-3.3638	3.6827
62	H	-2.3899	-2.7090	1.9571
63	C	1.0870	-3.5829	3.4120
64	H	1.8600	-3.1462	1.4594
65	C	-3.6780	-4.3203	-1.1360
66	H	-2.0461	-4.5094	0.2380
67	C	-3.7342	-2.3072	-2.4494
68	H	-2.1642	-0.8908	-2.0866
69	C	1.7055	-5.2345	-1.8182
70	H	0.7772	-4.8637	0.0766
71	C	1.6729	-3.4191	-3.3941
72	H	0.7590	-1.5987	-2.7210
73	C	-0.0463	-3.6671	4.2166
74	H	-2.1820	-3.4285	4.3037
75	H	2.0643	-3.8291	3.8155
76	C	-4.2887	-3.5356	-2.1091
77	H	-4.0954	-5.2868	-0.8745
78	H	-4.1969	-1.6905	-3.2141
79	C	2.0174	-4.7283	-3.0743
80	H	1.9622	-6.2575	-1.5638
81	H	1.9124	-3.0156	-4.3733
82	H	0.0424	-3.9759	5.2528
83	H	-5.1875	-3.8861	-2.6050
84	H	2.5227	-5.3534	-3.8032
85	C	-0.5144	-0.0897	3.1939
86	C	-2.2715	1.1671	2.4798
87	C	-0.7271	0.3095	4.5021
88	C	-1.6717	1.3104	4.6805
89	H	-0.1513	-0.1103	5.3182
90	H	-1.8213	1.7930	5.6447
91	N	-1.2053	0.3925	2.1499
92	C	3.2102	-0.8747	0.8674
93	C	2.1857	0.5161	2.3570
94	C	3.0265	0.0881	3.3708
95	C	3.8650	-0.9703	3.0566
96	H	2.9833	0.5327	4.3581

97	H	4.4792	-1.4582	3.8113
98	N	2.2060	0.0092	1.1150
99	H	0.2609	-0.8103	2.9589
100	H	1.4359	1.2763	2.5468
101	C	3.5741	-1.2204	-0.5134
102	C	3.4874	-0.2638	-1.5294
103	C	4.1806	-2.4539	-0.7833
104	C	4.0213	-0.5285	-2.7843
105	H	3.0405	0.7011	-1.3089
106	C	4.6825	-2.7250	-2.0456
107	H	4.2637	-3.1840	0.0141
108	C	4.6172	-1.7574	-3.0450
109	H	3.9901	0.2330	-3.5565
110	H	5.1367	-3.6886	-2.2498
111	H	5.0364	-1.9600	-4.0253
112	C	-3.3471	1.3855	1.5033
113	C	-3.6421	0.4043	0.5510
114	C	-4.1817	2.5058	1.6126
115	C	-4.7433	0.5446	-0.2828
116	H	-3.0234	-0.4840	0.4919
117	C	-5.2733	2.6487	0.7698
118	H	-3.9688	3.2459	2.3756
119	C	-5.5588	1.6668	-0.1764
120	H	-4.9714	-0.2354	-1.0021
121	H	-5.9120	3.5211	0.8575
122	H	-6.4241	1.7730	-0.8232
123	N	3.9811	-1.4259	1.8140
124	N	-2.4658	1.7039	3.6915

Ir(H)$_2$(PhPm)(IMes)(PPh$_3$)

1	C	0.5017	-3.7880	-2.0768
2	C	0.7749	-3.5944	-0.7169
3	C	2.0762	-3.5647	-0.2100
4	C	3.1262	-3.6251	-1.1263
5	C	2.9023	-3.7392	-2.4971
6	C	1.5844	-3.8519	-2.9484
7	H	1.3952	-3.9830	-4.0116
8	C	4.0392	-3.7014	-3.4687
9	H	4.9841	-3.9821	-3.0011
10	H	3.8690	-4.3640	-4.3198
11	H	4.1600	-2.6880	-3.8687
12	C	-0.9054	-3.9472	-2.5663
13	H	-1.2850	-4.9519	-2.3510
14	H	-1.5952	-3.2491	-2.0826
15	H	-0.9684	-3.8040	-3.6461
16	C	-1.0242	-4.5017	0.7370
17	C	-2.0110	-3.9686	1.4910
18	H	-0.7469	-5.5236	0.5380
19	H	-2.7798	-4.4273	2.0903
20	C	2.3363	-3.4956	1.2646
21	H	3.3650	-3.1963	1.4696
22	H	1.6663	-2.7988	1.7783
23	H	2.1803	-4.4734	1.7331
24	H	4.1470	-3.5619	-0.7564
25	N	-0.3294	-3.4390	0.1863
26	N	-1.8971	-2.5942	1.3872
27	C	-3.3417	-0.4149	4.0055
28	C	-4.7947	-0.4016	2.0982
29	H	-3.1077	-0.0835	5.0141
30	C	-2.4630	-1.2841	3.3656
31	C	-1.2067	-1.7577	4.0275
32	H	-1.1782	-2.8484	4.1160
33	H	-0.3237	-1.4662	3.4478
34	H	-1.1074	-1.3401	5.0297
35	C	-2.7864	-1.6903	2.0669
36	C	-3.9448	-1.2699	1.4121

37	C	-4.2574	-1.7335	0.0229
38	H	-5.1790	-1.2826	-0.3456
39	H	-3.4517	-1.4748	-0.6727
40	H	-4.3751	-2.8210	-0.0262
41	H	-5.7022	-0.0606	1.6058
42	C	-4.5128	0.0357	3.3913
43	C	-5.4425	0.9587	4.1179
44	H	-6.2694	1.2834	3.4840
45	H	-5.8747	0.4725	4.9973
46	H	-4.9209	1.8499	4.4786
47	Ir	-0.2905	-0.2646	0.1871
48	H	-1.8107	-0.0163	-0.0290
49	H	-0.6881	-0.0059	1.6993
50	C	-0.8533	-2.2351	0.5779
51	P	-0.0377	2.0679	0.0699
52	C	0.6425	2.6575	-1.5189
53	C	-1.6215	2.9726	0.2476
54	C	1.0101	2.8398	1.3554
55	C	-0.1799	2.6210	-2.6534
56	C	1.9926	2.9800	-1.6741
57	C	-1.8126	4.2275	-0.3409
58	C	-2.6298	2.4392	1.0574
59	C	1.3948	4.1820	1.2557
60	C	1.3772	2.1104	2.4879
61	C	0.3336	2.9217	-3.9076
62	H	-1.2318	2.3647	-2.5472
63	C	2.5046	3.2803	-2.9328
64	H	2.6512	2.9901	-0.8104
65	C	-2.9953	4.9273	-0.1349
66	H	-1.0409	4.6573	-0.9727
67	C	-3.8096	3.1454	1.2621
68	H	-2.4925	1.4706	1.5299
69	C	2.1515	4.7716	2.2591
70	H	1.1047	4.7681	0.3882
71	C	2.1323	2.7043	3.4939
72	H	1.0601	1.0754	2.5793
73	C	1.6789	3.2540	-4.0499
74	H	-0.3175	2.9011	-4.7756
75	H	3.5547	3.5340	-3.0368
76	C	-3.9965	4.3873	0.6654
77	H	-3.1343	5.8965	-0.6018
78	H	-4.5862	2.7174	1.8877
79	C	2.5245	4.0323	3.3785
80	H	2.4471	5.8114	2.1704
81	H	2.4109	2.1289	4.3708
82	H	2.0795	3.4934	-5.0292
83	H	-4.9196	4.9347	0.8232
84	H	3.1137	4.4959	4.1625
85	C	1.6604	-0.4641	-2.3343
86	C	-0.5471	-0.7563	-2.9123
87	C	-0.2008	-0.8371	-4.2466
88	H	-1.5799	-0.8241	-2.5834
89	C	1.1510	-0.7084	-4.5441
90	H	-0.9505	-0.9811	-5.0144
91	H	1.5095	-0.7518	-5.5706
92	C	2.6865	-0.2168	-1.3113
93	C	3.9418	0.2634	-1.7061
94	C	2.4513	-0.4106	0.0563
95	C	4.9118	0.5700	-0.7643
96	H	4.1253	0.4084	-2.7644
97	C	3.4230	-0.1038	1.0013
98	H	1.5958	-1.0110	0.4136
99	C	4.6522	0.4008	0.5941
100	H	5.8749	0.9493	-1.0887
101	H	3.2236	-0.2804	2.0530
102	H	5.4097	0.6452	1.3308

103	N	0.3634	-0.5763	-1.9441
104	N	2.0703	-0.5226	-3.6058

$Ir(H)_2(PhPm)(IMes)(PPh_3)$ C-C Rotation TS2 ***Imaginary Frequency: -77.0 cm^{-1}***

1	C	0.0133	-3.2546	-2.5575
2	C	0.5650	-3.5901	-1.3149
3	C	1.8203	-4.1962	-1.1981
4	C	2.5805	-4.3405	-2.3603
5	C	2.1012	-3.9511	-3.6090
6	C	0.8041	-3.4396	-3.6874
7	H	0.3918	-3.1825	-4.6609
8	C	2.9468	-4.0778	-4.8374
9	H	3.3953	-3.1142	-5.1038
10	H	3.7651	-4.7853	-4.6970
11	H	2.3601	-4.4027	-5.6992
12	C	-1.3879	-2.7404	-2.6723
13	H	-2.0972	-3.3869	-2.1459
14	H	-1.4941	-1.7460	-2.2264
15	H	-1.6990	-2.6834	-3.7165
16	C	-0.8245	-4.4663	0.5184
17	C	-1.5068	-3.9492	1.5621
18	H	-0.7028	-5.4796	0.1738
19	H	-2.1072	-4.4158	2.3251
20	C	2.3256	-4.7393	0.1068
21	H	3.4164	-4.7106	0.1521
22	H	1.9297	-4.2016	0.9714
23	H	2.0351	-5.7887	0.2255
24	H	3.5684	-4.7879	-2.2853
25	N	-0.2280	-3.4006	-0.1341
26	N	-1.3160	-2.5816	1.5267
27	C	-1.9973	-0.3172	4.3821
28	C	-3.9834	-0.5322	3.0590
29	H	-1.4728	0.1101	5.2334
30	C	-1.3065	-1.1822	3.5343
31	C	0.1496	-1.4636	3.7202
32	H	0.3797	-2.5325	3.6666
33	H	0.7332	-0.9822	2.9253
34	H	0.5120	-1.0849	4.6772
35	C	-1.9988	-1.7058	2.4421
36	C	-3.3368	-1.3939	2.1777
37	C	-4.0227	-1.9177	0.9537
38	H	-5.0120	-1.4736	0.8370
39	H	-3.4420	-1.6939	0.0512
40	H	-4.1497	-3.0046	0.9810
41	H	-5.0228	-0.2738	2.8698
42	C	-3.3319	0.0210	4.1633
43	C	-4.0551	0.9630	5.0748
44	H	-4.4490	1.8217	4.5229
45	H	-4.9103	0.4768	5.5527
46	H	-3.4054	1.3429	5.8640
47	Ir	-0.1551	-0.1993	-0.0233
48	H	-1.5430	-0.2400	-0.7130
49	H	-1.0121	0.0767	1.2661
50	C	-0.5158	-2.2042	0.4745
51	P	-0.3573	2.1120	-0.3252
52	C	0.2706	2.7198	-1.9305
53	C	-2.0938	2.7049	-0.2801
54	C	0.3977	3.1737	0.9616
55	C	-0.5570	2.6619	-3.0589
56	C	1.6154	3.0659	-2.1026
57	C	-2.4066	3.9818	-0.7622
58	C	-3.0944	1.9484	0.3344
59	C	0.8380	4.4771	0.7118
60	C	0.4385	2.6784	2.2693
61	C	-0.0605	2.9800	-4.3182
62	H	-1.6010	2.3816	-2.9474

63	C	2.1108	3.3801	-3.3633
64	H	2.2814	3.1125	-1.2458
65	C	-3.6977	4.4794	-0.6506
66	H	-1.6384	4.5895	-1.2325
67	C	-4.3852	2.4544	0.4517
68	H	-2.8625	0.9672	0.7334
69	C	1.3316	5.2599	1.7495
70	H	0.7961	4.8862	-0.2932
71	C	0.9179	3.4699	3.3056
72	H	0.0939	1.6669	2.4700
73	C	1.2729	3.3444	-4.4733
74	H	-0.7210	2.9510	-5.1786
75	H	3.1535	3.6595	-3.4746
76	C	-4.6910	3.7158	-0.0437
77	H	-3.9283	5.4675	-1.0340
78	H	-5.1529	1.8557	0.9322
79	C	1.3729	4.7587	3.0467
80	H	1.6773	6.2675	1.5446
81	H	0.9413	3.0758	4.3169
82	H	1.6572	3.6026	-5.4544
83	H	-5.6985	4.1078	0.0450
84	H	1.7535	5.3747	3.8543
85	C	2.5776	-0.1842	-1.3811
86	C	1.0451	-0.2838	-3.0834
87	C	2.0582	-0.0689	-4.0017
88	H	0.0074	-0.3934	-3.3853
89	C	3.3411	0.0799	-3.4979
90	H	1.8478	-0.0103	-5.0626
91	H	4.1895	0.2559	-4.1556
92	C	2.8324	-0.2434	0.0857
93	C	3.0394	-1.4870	0.6915
94	C	2.9611	0.9176	0.8529
95	C	3.3808	-1.5695	2.0338
96	H	2.9725	-2.3761	0.0789
97	C	3.2938	0.8318	2.2018
98	H	2.8313	1.8910	0.3916
99	C	3.5106	-0.4078	2.7906
100	H	3.5580	-2.5399	2.4870
101	H	3.3962	1.7416	2.7834
102	H	3.7860	-0.4704	3.8382
103	N	1.2948	-0.3537	-1.7684
104	N	3.6088	0.0282	-2.1892

$Ir(H)_2(PhPm)(IMes)(PPh_3)$ C-H Activation TS1 *Imaginary Frequency: -669.1 cm^{-1}*

1	Ir	-0.2082	-0.2386	0.3115
2	H	0.3714	-0.3481	1.8276
3	P	-0.6790	2.0418	0.7243
4	C	0.5812	-4.4531	0.8652
5	C	-0.2494	-4.5315	-0.1965
6	H	1.0595	-5.2139	1.4590
7	H	-0.6514	-5.3756	-0.7317
8	N	0.7639	-3.1067	1.1303
9	N	-0.5575	-3.2322	-0.5589
10	C	0.0653	-2.3264	0.2524
11	H	-1.5592	-0.5224	1.1197
12	C	0.8121	0.2624	-2.4827
13	C	-0.4509	0.1963	-1.8393
14	C	-1.5732	0.5237	-2.6082
15	C	-1.4658	0.8620	-3.9534
16	C	-0.2251	0.8637	-4.5854
17	C	0.9082	0.5725	-3.8459
18	H	-1.3964	-0.5560	-0.7156
19	H	-2.5607	0.5071	-2.1572
20	H	-2.3635	1.1114	-4.5117
21	H	-0.1424	1.1134	-5.6376
22	H	1.8985	0.6181	-4.2880

23	C	2.0276	0.1960	-1.6850
24	C	-1.3314	-2.9310	-1.7334
25	C	-2.7094	-2.7320	-1.6141
26	C	-0.6613	-2.8766	-2.9608
27	C	-3.4177	-2.4433	-2.7801
28	C	-1.4181	-2.5954	-4.0945
29	C	-2.7929	-2.3705	-4.0252
30	H	-4.4891	-2.2717	-2.7104
31	H	-0.9145	-2.5278	-5.0555
32	C	1.6778	-2.6472	2.1408
33	C	1.1875	-2.3194	3.4100
34	C	3.0365	-2.5957	1.8211
35	C	2.1048	-1.8694	4.3550
36	C	3.9167	-2.1529	2.8103
37	C	3.4717	-1.7779	4.0767
38	H	1.7443	-1.5962	5.3439
39	H	4.9787	-2.1029	2.5813
40	C	3.5315	-3.0084	0.4680
41	H	2.9515	-2.5477	-0.3387
42	H	4.5789	-2.7361	0.3314
43	H	3.4539	-4.0910	0.3237
44	C	4.4323	-1.3194	5.1299
45	H	5.4202	-1.1125	4.7164
46	H	4.0777	-0.4164	5.6326
47	H	4.5561	-2.0817	5.9051
48	C	-0.2653	-2.4685	3.7370
49	H	-0.5575	-3.5235	3.7636
50	H	-0.4955	-2.0408	4.7139
51	H	-0.9000	-1.9834	2.9897
52	C	0.8155	-3.1097	-3.0489
53	H	1.3597	-2.5544	-2.2788
54	H	1.0665	-4.1670	-2.9102
55	H	1.2049	-2.8021	-4.0201
56	C	-3.4005	-2.8458	-0.2900
57	H	-2.8699	-2.3043	0.4982
58	H	-4.4191	-2.4592	-0.3442
59	H	-3.4655	-3.8897	0.0351
60	C	-3.5786	-2.0648	-5.2624
61	H	-3.9564	-2.9820	-5.7257
62	H	-4.4455	-1.4368	-5.0466
63	H	-2.9664	-1.5582	-6.0117
64	C	0.6662	3.0954	0.0916
65	C	1.6819	3.5330	0.9481
66	C	0.8008	3.3024	-1.2874
67	C	2.8043	4.1745	0.4352
68	H	1.5933	3.3755	2.0193
69	C	1.9258	3.9411	-1.7942
70	H	0.0241	2.9635	-1.9670
71	C	2.9302	4.3770	-0.9349
72	H	3.5804	4.5199	1.1101
73	H	2.0159	4.0969	-2.8645
74	H	3.8058	4.8789	-1.3326
75	C	-1.1311	3.9003	2.7451
76	C	-0.8135	1.6389	3.5227
77	C	-1.3012	4.3278	4.0537
78	H	-1.1932	4.6194	1.9324
79	C	-0.9896	2.0714	4.8340
80	H	-0.6250	0.5919	3.3155
81	C	-1.2304	3.4128	5.1015
82	H	-1.4932	5.3756	4.2576
83	H	-0.9399	1.3540	5.6470
84	H	-1.3676	3.7482	6.1239
85	C	-2.2231	2.6409	-0.0478
86	C	-2.3239	3.8794	-0.6857
87	C	-3.3616	1.8333	0.0555
88	C	-3.5351	4.2877	-1.2355

89	H	-1.4578	4.5291	-0.7595
90	C	-4.5715	2.2505	-0.4825
91	H	-3.2962	0.8699	0.5546
92	C	-4.6580	3.4748	-1.1388
93	H	-3.5995	5.2479	-1.7361
94	H	-5.4482	1.6176	-0.3921
95	H	-5.6011	3.7974	-1.5668
96	C	-0.8788	2.5502	2.4684
97	N	1.8447	0.1248	-0.3385
98	C	4.2854	0.3809	-1.4648
99	H	5.2540	0.4425	-1.9564
100	C	2.9156	0.2654	0.4539
101	H	2.7283	0.2440	1.5230
102	C	4.1829	0.4142	-0.0777
103	H	5.0474	0.5332	0.5629
104	N	3.2278	0.2848	-2.2636

Ir(H)(H$_2$)(PhPm)(IMes)(PPh$_3$)

1	Ir	0.1180	-0.1800	0.3731
2	H	1.1220	0.4773	1.7121
3	P	-1.3558	1.6899	0.3498
4	C	2.9093	-3.4130	1.0334
5	C	1.7749	-4.1340	0.9069
6	H	3.9234	-3.7013	1.2544
7	H	1.5842	-5.1906	0.9951
8	N	2.5615	-2.0901	0.8171
9	N	0.7604	-3.2399	0.6149
10	C	1.2275	-1.9581	0.5512
11	H	0.4493	0.1254	2.1013
12	C	0.0026	-0.2644	-2.5843
13	C	-0.6950	-0.7065	-1.4353
14	C	-1.9022	-1.3808	-1.6377
15	C	-2.3721	-1.6444	-2.9206
16	C	-1.6487	-1.2413	-4.0447
17	C	-0.4655	-0.5439	-3.8759
18	H	-1.0055	-0.9882	1.1437
19	H	-2.4817	-1.7170	-0.7827
20	H	-3.3126	-2.1752	-3.0440
21	H	-2.0190	-1.4576	-5.0408
22	H	0.1025	-0.1768	-4.7245
23	C	1.1500	0.6027	-2.3808
24	C	-0.5928	-3.6772	0.3924
25	C	-1.5073	-3.6350	1.4490
26	C	-0.9191	-4.1855	-0.8683
27	C	-2.8116	-4.0511	1.1843
28	C	-2.2253	-4.6263	-1.0670
29	C	-3.1916	-4.5430	-0.0652
30	H	-3.5444	-4.0121	1.9870
31	H	-2.5015	-5.0137	-2.0445
32	C	3.5380	-1.0369	0.8339
33	C	3.7841	-0.3382	2.0188
34	C	4.2676	-0.8064	-0.3407
35	C	4.7696	0.6536	1.9890
36	C	5.2598	0.1674	-0.3056
37	C	5.5222	0.9142	0.8462
38	H	4.9714	1.2146	2.8983
39	H	5.8319	0.3632	-1.2095
40	C	3.9688	-1.5720	-1.5926
41	H	4.2948	-2.6146	-1.5170
42	H	2.8946	-1.5992	-1.8033
43	H	4.4741	-1.1328	-2.4536
44	C	6.6011	1.9527	0.8488
45	H	6.5619	2.5782	-0.0469
46	H	6.5374	2.6038	1.7216
47	H	7.5915	1.4877	0.8602
48	C	3.0802	-0.6574	3.3037

49	H	2.7539	0.2519	3.8157
50	H	2.2103	-1.3001	3.1627
51	H	3.7567	-1.1758	3.9904
52	C	0.0844	-4.2000	-1.9786
53	H	0.9575	-4.8157	-1.7404
54	H	-0.3546	-4.5851	-2.8992
55	H	0.4535	-3.1885	-2.1834
56	C	-1.1016	-3.2196	2.8302
57	H	-0.6445	-4.0578	3.3677
58	H	-0.3730	-2.4066	2.8253
59	H	-1.9653	-2.8972	3.4145
60	C	-4.6104	-4.9380	-0.3346
61	H	-5.1174	-5.2769	0.5706
62	H	-5.1823	-4.0877	-0.7235
63	H	-4.6757	-5.7330	-1.0796
64	C	-1.1705	2.7222	-1.1496
65	C	-0.3465	3.8513	-1.1660
66	C	-1.7730	2.3022	-2.3426
67	C	-0.1310	4.5464	-2.3514
68	H	0.1168	4.2043	-0.2500
69	C	-1.5547	3.0008	-3.5228
70	H	-2.4142	1.4248	-2.3492
71	C	-0.7314	4.1220	-3.5313
72	H	0.5015	5.4282	-2.3478
73	H	-2.0273	2.6616	-4.4387
74	H	-0.5634	4.6665	-4.4543
75	C	-2.1574	3.3803	2.4788
76	C	0.2146	3.1873	2.1097
77	C	-1.9140	4.2517	3.5359
78	H	-3.1794	3.1173	2.2275
79	C	0.4543	4.0667	3.1571
80	H	1.0545	2.7709	1.5581
81	C	-0.6124	4.5988	3.8752
82	H	-2.7489	4.6593	4.0956
83	H	1.4734	4.3347	3.4157
84	H	-0.4274	5.2795	4.6990
85	C	-3.1383	1.2932	0.3973
86	C	-4.0761	2.1947	-0.1221
87	C	-3.5939	0.1216	1.0082
88	C	-5.4355	1.9186	-0.0451
89	H	-3.7420	3.1156	-0.5904
90	C	-4.9546	-0.1522	1.0821
91	H	-2.8811	-0.5862	1.4191
92	C	-5.8774	0.7430	0.5532
93	H	-6.1503	2.6242	-0.4545
94	H	-5.2922	-1.0702	1.5526
95	H	-6.9391	0.5271	0.6076
96	C	-1.0941	2.8386	1.7522
97	N	1.4882	0.8339	-1.0795
98	C	2.7578	2.0119	-3.1627
99	H	3.2497	2.4576	-4.0253
100	C	2.4839	1.6933	-0.8422
101	H	2.7563	1.8446	0.2005
102	C	3.1525	2.3348	-1.8687
103	H	3.9544	3.0333	-1.6652
104	N	1.7776	1.1549	-3.4241

1-Phenylpyrazole

PhPz

1	C	-2.4668	1.2166	-0.1046
2	C	-1.0796	1.1913	-0.1014
3	C	-0.4152	-0.0303	0.0032
4	C	-1.1413	-1.2163	0.1094
5	C	-2.5294	-1.1755	0.0966

6	C	-3.1997	0.0376	-0.0106
7	H	-2.9788	2.1697	-0.1861
8	H	-0.4919	2.0970	-0.1743
9	H	-0.6311	-2.1662	0.2189
10	H	-3.0878	-2.1018	0.1810
11	H	-4.2838	0.0641	-0.0164
12	N	0.9995	-0.0526	0.0012
13	C	1.8248	-1.1262	-0.1631
14	C	3.1120	-0.6527	-0.0975
15	H	1.4403	-2.1187	-0.3311
16	C	2.9662	0.7314	0.1082
17	H	4.0207	-1.2249	-0.1918
18	H	3.7358	1.4817	0.2195
19	N	1.6934	1.0988	0.1669

Ir(H)$_2$(CHCl$_3$)(PhPz)(IMes)(PPh$_3$)

1	C	1.7290	-3.1273	-2.4577
2	C	2.1551	-2.9051	-1.1421
3	C	3.4737	-2.5695	-0.8282
4	C	4.3613	-2.3799	-1.8895
5	C	3.9760	-2.5669	-3.2152
6	C	2.6596	-2.9555	-3.4764
7	H	2.3434	-3.1111	-4.5053
8	C	4.9451	-2.3711	-4.3402
9	H	5.9000	-1.9779	-3.9897
10	H	5.1452	-3.3135	-4.8583
11	H	4.5518	-1.6800	-5.0915
12	C	0.3182	-3.5353	-2.7491
13	H	-0.4019	-2.8992	-2.2247
14	H	0.1107	-3.4848	-3.8194
15	H	0.1210	-4.5624	-2.4227
16	C	1.0538	-4.3630	0.5197
17	C	0.0367	-4.2403	1.4001
18	H	1.6800	-5.1999	0.2588
19	H	-0.4169	-4.9495	2.0723
20	C	3.9384	-2.4657	0.5907
21	H	4.7719	-1.7665	0.6865
22	H	3.1423	-2.1456	1.2657
23	H	4.2929	-3.4368	0.9537
24	H	5.3864	-2.0937	-1.6674
25	N	1.2111	-3.1247	-0.0817
26	N	-0.4050	-2.9326	1.3190
27	C	-2.4499	-1.4353	4.0237
28	C	-3.8882	-2.0696	2.2136
29	H	-2.3143	-1.0047	5.0130
30	C	-1.3311	-1.9031	3.3379
31	C	0.0340	-1.8445	3.9497
32	H	0.2726	-2.7765	4.4740
33	H	0.8080	-1.6915	3.1951
34	H	0.1042	-1.0362	4.6805
35	C	-1.5331	-2.4457	2.0661
36	C	-2.7996	-2.5477	1.4840
37	C	-2.9694	-3.1374	0.1186
38	H	-4.0236	-3.2518	-0.1344
39	H	-2.5093	-2.5013	-0.6469
40	H	-2.4930	-4.1194	0.0357
41	H	-4.8826	-2.1417	1.7780
42	C	-3.7346	-1.5141	3.4840
43	C	-4.9167	-1.0344	4.2696
44	H	-5.1123	-1.6890	5.1243
45	H	-4.7511	-0.0320	4.6740
46	H	-5.8253	-1.0124	3.6648
47	Ir	0.0226	-0.1810	0.0790
48	H	-1.4783	-0.5599	0.1123
49	H	-0.0139	-0.1009	1.6587
50	C	0.3163	-2.2118	0.4028

51	P	-0.5503	2.0965	0.1768
52	C	-0.5871	2.9823	-1.4236
53	C	-2.1590	2.4812	0.9621
54	C	0.5797	3.0751	1.2463
55	C	-1.7500	3.5372	-1.9662
56	C	0.5841	2.9870	-2.1932
57	C	-2.4947	3.8190	1.2122
58	C	-3.0482	1.4758	1.3446
59	C	1.4063	4.1026	0.7848
60	C	0.6112	2.7342	2.6054
61	C	-1.7367	4.0927	-3.2419
62	H	-2.6737	3.5337	-1.3958
63	C	0.5985	3.5558	-3.4591
64	H	1.4904	2.5357	-1.7961
65	C	-3.7047	4.1414	1.8095
66	H	-1.8014	4.6105	0.9381
67	C	-4.2636	1.8031	1.9399
68	H	-2.7902	0.4328	1.1896
69	C	2.2647	4.7609	1.6634
70	H	1.3785	4.4017	-0.2574
71	C	1.4572	3.4030	3.4806
72	H	-0.0272	1.9345	2.9755
73	C	-0.5643	4.1084	-3.9878
74	H	-2.6471	4.5189	-3.6496
75	H	1.5161	3.5576	-4.0383
76	C	-4.5954	3.1323	2.1696
77	H	-3.9537	5.1803	1.9971
78	H	-4.9476	1.0097	2.2251
79	C	2.2951	4.4116	3.0096
80	H	2.9038	5.5555	1.2930
81	H	1.4714	3.1288	4.5302
82	H	-0.5558	4.5478	-4.9795
83	H	-5.5421	3.3858	2.6349
84	H	2.9621	4.9281	3.6913
85	Cl	2.6981	0.5361	-0.0253
86	C	3.4026	1.1906	1.5307
87	H	2.9702	2.1791	1.6587
88	Cl	2.9428	0.1909	2.8988
89	C	-2.3092	-0.6413	-2.7738
90	C	-2.9186	-1.7305	-3.3953
91	C	-3.0211	0.1501	-1.8784
92	C	-4.2471	-2.0258	-3.1136
93	H	-2.3493	-2.3407	-4.0889
94	C	-4.3406	-0.1683	-1.5828
95	H	-2.5499	1.0171	-1.4357
96	C	-4.9571	-1.2508	-2.2027
97	H	-4.7227	-2.8697	-3.6009
98	H	-4.8908	0.4508	-0.8812
99	H	-5.9925	-1.4866	-1.9814
100	N	0.0662	-0.2417	-2.2336
101	C	1.1583	-0.0833	-2.9845
102	C	0.8582	-0.0715	-4.3485
103	C	-0.5049	-0.2357	-4.4029
104	N	-0.9634	-0.3273	-3.1280
105	H	2.1280	-0.0117	-2.5118
106	H	1.5393	0.0469	-5.1751
107	H	-1.1957	-0.2743	-5.2307
108	Cl	5.1399	1.3386	1.3510

Ir(H)$_2$(DCM)(PhPz)(IMes)(PPh$_3$)

1	C	-1.0410	-1.6533	3.4862
2	C	-1.1103	-2.4340	2.3305
3	C	-2.3248	-2.7754	1.7290
4	C	-3.4932	-2.2590	2.2842
5	C	-3.4699	-1.4380	3.4117
6	C	-2.2371	-1.1575	4.0016
7	H	-2.2033	-0.5316	4.8904
8	C	-4.7370	-0.8988	4.0000
9	H	-5.0546	-1.4953	4.8615
10	H	-4.6108	0.1276	4.3519
11	H	-5.5559	-0.9116	3.2782
12	C	0.2701	-1.3692	4.1494
13	H	0.5709	-2.2001	4.7973
14	H	1.0681	-1.2260	3.4185
15	H	0.2152	-0.4762	4.7754
16	C	0.6882	-4.1032	1.9485
17	C	1.7241	-4.2265	1.0901
18	H	0.3114	-4.7635	2.7118
19	H	2.4449	-5.0136	0.9434
20	C	-2.3560	-3.6748	0.5327
21	H	-1.6670	-3.3310	-0.2448
22	H	-2.0467	-4.6936	0.7895
23	H	-3.3583	-3.7368	0.1078
24	H	-4.4469	-2.5022	1.8205
25	N	0.1019	-2.8783	1.6948
26	N	1.7465	-3.0706	0.3252
27	C	3.1479	-3.2344	-3.0904
28	C	4.7988	-2.2888	-1.6328
29	H	2.8382	-3.5556	-4.0820
30	C	2.2440	-3.3407	-2.0399
31	C	0.8584	-3.8625	-2.2514
32	H	0.1220	-3.1305	-1.9047
33	H	0.6684	-4.0679	-3.3057
34	H	0.6751	-4.7839	-1.6885
35	C	2.6577	-2.9060	-0.7732
36	C	3.9394	-2.4061	-0.5368
37	C	4.4022	-2.0584	0.8430
38	H	4.8626	-2.9268	1.3271
39	H	5.1576	-1.2698	0.8210
40	H	3.5831	-1.7315	1.4855
41	H	5.7950	-1.8836	-1.4732
42	C	4.4263	-2.6991	-2.9109
43	C	5.3666	-2.5786	-4.0702
44	H	6.2869	-2.0622	-3.7950
45	H	5.6426	-3.5631	-4.4588
46	H	4.9090	-2.0334	-4.9008
47	Ir	0.2183	-0.2786	0.1137
48	H	-1.2283	-0.8360	0.1305
49	H	0.0557	-0.0793	1.6680
50	C	0.7477	-2.2074	0.6831
51	P	-0.6895	1.8908	0.1089
52	C	-0.8279	2.8731	-1.4301
53	C	-2.3806	1.9571	0.8088
54	C	0.2531	3.0059	1.2229
55	C	-1.9262	3.6939	-1.7041
56	C	0.2166	2.8010	-2.3573
57	C	-2.7952	2.9741	1.6749
58	C	-3.3062	0.9909	0.4085
59	C	0.8986	4.1600	0.7705
60	C	0.3399	2.6663	2.5795
61	C	-1.9795	4.4199	-2.8891
62	H	-2.7481	3.7568	-0.9976
63	C	0.1618	3.5284	-3.5407
64	H	1.0770	2.1689	-2.1507
65	C	-4.1055	3.0089	2.1408

66	H	-2.0973	3.7443	1.9877
67	C	-4.6171	1.0348	0.8652
68	H	-2.9974	0.1968	-0.2625
69	C	1.6236	4.9533	1.6570
70	H	0.8334	4.4479	-0.2739
71	C	1.0506	3.4678	3.4633
72	H	-0.1563	1.7696	2.9427
73	C	-0.9393	4.3366	-3.8103
74	H	-2.8362	5.0537	-3.0932
75	H	0.9787	3.4610	-4.2521
76	C	-5.0189	2.0408	1.7371
77	H	-4.4132	3.7996	2.8169
78	H	-5.3216	0.2727	0.5445
79	C	1.7006	4.6107	3.0031
80	H	2.1198	5.8471	1.2937
81	H	1.1071	3.1933	4.5117
82	H	-0.9849	4.9034	-4.7343
83	H	-6.0399	2.0699	2.1041
84	H	2.2595	5.2341	3.6928
85	Cl	2.8075	0.8062	0.0156
86	C	3.3931	1.5867	1.5352
87	H	4.4659	1.7079	1.4049
88	H	2.8767	2.5396	1.6289
89	Cl	3.0925	0.6214	2.9703
90	C	-1.8091	-0.2671	-2.9193
91	C	-2.5883	0.8026	-3.3491
92	C	-2.3930	-1.3950	-2.3506
93	C	-3.9688	0.7422	-3.1981
94	H	-2.1100	1.6732	-3.7876
95	C	-3.7735	-1.4451	-2.2041
96	H	-1.7648	-2.2239	-2.0481
97	C	-4.5623	-0.3790	-2.6284
98	H	-4.5782	1.5781	-3.5242
99	H	-4.2355	-2.3268	-1.7729
100	H	-5.6403	-0.4240	-2.5175
101	N	0.5247	-0.4624	-2.1814
102	C	1.7031	-0.3776	-2.8092
103	C	1.5468	-0.0911	-4.1677
104	C	0.1859	-0.0032	-4.3507
105	N	-0.4040	-0.2277	-3.1505
106	H	2.6179	-0.5373	-2.2555
107	H	2.3174	0.0166	-4.9134
108	H	-0.4168	0.1681	-5.2290

Ir(H)$_2$(PhPz)$_2$(IMes)(PPh$_3$)

1	C	-2.5028	-3.8832	0.2982
2	C	-1.7388	-3.3069	-0.7237
3	C	-2.3205	-2.8185	-1.9025
4	C	-3.7114	-2.8418	-1.9959
5	C	-4.5140	-3.3708	-0.9845
6	C	-3.8891	-3.8977	0.1444
7	H	-4.4961	-4.3299	0.9365
8	C	-6.0068	-3.3735	-1.1000
9	H	-6.4092	-4.3892	-1.0548
10	H	-6.4669	-2.8162	-0.2782
11	H	-6.3412	-2.9252	-2.0368
12	C	-1.8732	-4.5121	1.5030
13	H	-2.5222	-4.4247	2.3765
14	H	-1.7021	-5.5821	1.3414
15	H	-0.9084	-4.0679	1.7504
16	C	0.3914	-4.5097	-0.8086
17	C	1.7011	-4.2058	-0.7282
18	H	-0.1180	-5.4421	-0.9861
19	H	2.5865	-4.8112	-0.8280
20	C	-1.4805	-2.3339	-3.0435
21	H	-2.1051	-1.9626	-3.8574

22	H	-0.7975	-1.5366	-2.7385
23	H	-0.8603	-3.1431	-3.4445
24	H	-4.1793	-2.4531	-2.8974
25	N	-0.3046	-3.3281	-0.6199
26	N	1.7861	-2.8414	-0.4890
27	C	4.8805	-1.5261	-1.9998
28	C	5.2423	-1.7789	0.3526
29	H	5.2516	-1.2749	-2.9900
30	C	3.5450	-1.8922	-1.8586
31	C	2.6278	-1.9466	-3.0404
32	H	2.2228	-2.9523	-3.1977
33	H	1.7686	-1.2810	-2.9019
34	H	3.1462	-1.6479	-3.9523
35	C	3.0858	-2.2204	-0.5763
36	C	3.9209	-2.1810	0.5442
37	C	3.4447	-2.5657	1.9103
38	H	4.2654	-2.9537	2.5161
39	H	3.0395	-1.6963	2.4403
40	H	2.6638	-3.3297	1.8765
41	H	5.9045	-1.7418	1.2151
42	C	5.7468	-1.4699	-0.9100
43	C	7.1896	-1.1117	-1.0913
44	H	7.3725	-0.6608	-2.0682
45	H	7.5327	-0.4118	-0.3246
46	H	7.8271	-1.9983	-1.0150
47	Ir	-0.0372	-0.3054	-0.0420
48	H	0.4620	0.0728	-1.4916
49	H	-1.3838	-0.7140	-0.6843
50	C	0.5447	-2.2642	-0.4104
51	P	-0.8255	1.9338	0.0195
52	C	-1.1114	2.8504	1.5870
53	C	0.3389	3.1024	-0.7961
54	C	-2.3748	2.1336	-0.9508
55	C	-1.0707	2.1963	2.8169
56	C	-1.3095	4.2389	1.5746
57	C	1.3764	3.6594	-0.0399
58	C	0.2707	3.3840	-2.1622
59	C	-3.4041	3.0036	-0.5867
60	C	-2.5186	1.3771	-2.1223
61	C	-1.2413	2.8969	4.0072
62	H	-0.8942	1.1292	2.8427
63	C	-1.4920	4.9381	2.7597
64	H	-1.3042	4.7768	0.6309
65	C	2.3259	4.4771	-0.6401
66	H	1.4401	3.4571	1.0268
67	C	1.2202	4.2079	-2.7579
68	H	-0.5329	2.9706	-2.7644
69	C	-4.5418	3.1234	-1.3794
70	H	-3.3405	3.5761	0.3316
71	C	-3.6527	1.5025	-2.9141
72	H	-1.7337	0.6845	-2.4104
73	C	-1.4595	4.2680	3.9804
74	H	-1.1995	2.3678	4.9547
75	H	-1.6489	6.0112	2.7321
76	C	2.2513	4.7532	-2.0010
77	H	3.1272	4.8989	-0.0405
78	H	1.1498	4.4273	-3.8185
79	C	-4.6695	2.3787	-2.5449
80	H	-5.3341	3.8008	-1.0768
81	H	-3.7413	0.9147	-3.8228
82	H	-1.5926	4.8183	4.9058
83	H	2.9927	5.3916	-2.4695
84	H	-5.5562	2.4781	-3.1623
85	C	1.9457	0.7587	2.3689
86	C	2.9896	1.4925	2.9363
87	C	3.8083	1.7877	1.8751

88	N	2.0915	0.5971	1.0502
89	C	-0.1380	-1.8162	2.8404
90	C	-0.8281	-2.1039	4.0200
91	C	-2.0756	-1.5549	3.8307
92	N	-0.8895	-1.1197	1.9801
93	C	-3.2225	-0.2924	2.0518
94	C	-3.6700	-0.5754	0.7666
95	C	-3.9205	0.5932	2.8720
96	C	-4.8295	0.0326	0.3032
97	H	-3.1253	-1.2740	0.1490
98	C	-5.0808	1.1917	2.3989
99	H	-3.5480	0.8178	3.8659
100	C	-5.5382	0.9105	1.1153
101	H	-5.1737	-0.1844	-0.7033
102	H	-5.6231	1.8826	3.0349
103	H	-6.4422	1.3825	0.7454
104	C	3.8093	1.3804	-0.5509
105	C	3.0040	1.2894	-1.6773
106	C	5.1656	1.6821	-0.6840
107	C	3.5494	1.5111	-2.9344
108	H	1.9560	1.0574	-1.5594
109	C	5.6975	1.9179	-1.9440
110	H	5.8148	1.7060	0.1839
111	C	4.8933	1.8357	-3.0764
112	H	2.9053	1.4471	-3.8061
113	H	6.7524	2.1534	-2.0377
114	H	5.3134	2.0229	-4.0587
115	N	3.2617	1.2385	0.7566
116	H	4.7116	2.3716	1.8208
117	H	3.1210	1.7805	3.9659
118	H	1.0851	0.3328	2.8576
119	N	-2.0946	-0.9766	2.6023
120	H	0.8739	-2.0676	2.5581
121	H	-0.4798	-2.6563	4.8769
122	H	-2.9643	-1.5585	4.4422

$Ir(H)_2(PhPz)(IMes)(PPh_3)$

1	C	-1.7099	-3.5960	1.6866
2	C	-1.4410	-3.4593	0.3233
3	C	-2.4545	-3.3216	-0.6352
4	C	-3.7704	-3.3047	-0.1862
5	C	-4.0868	-3.3940	1.1718
6	C	-3.0469	-3.5414	2.0870
7	H	-3.2783	-3.6283	3.1462
8	C	-5.5105	-3.3014	1.6248
9	H	-5.6183	-3.5523	2.6808
10	H	-5.8978	-2.2869	1.4835
11	H	-6.1587	-3.9685	1.0514
12	C	-0.6155	-3.8368	2.6794
13	H	-0.3768	-4.9041	2.7424
14	H	0.3086	-3.3207	2.4102
15	H	-0.9090	-3.5138	3.6795
16	C	0.6038	-4.6208	-0.4719
17	C	1.8176	-4.2315	-0.9200
18	H	0.1576	-5.5953	-0.3641
19	H	2.6608	-4.7966	-1.2810
20	C	-2.1177	-3.1244	-2.0816
21	H	-3.0158	-3.1288	-2.7002
22	H	-1.6026	-2.1670	-2.2384
23	H	-1.4423	-3.8977	-2.4596
24	H	-4.5714	-3.1907	-0.9130
25	N	-0.0796	-3.4654	-0.1293
26	N	1.8485	-2.8502	-0.8467
27	C	4.1962	-0.9192	-2.9592
28	C	5.0479	-0.9929	-0.7193
29	H	4.2903	-0.5974	-3.9936

30	C	3.0819	-1.6743	-2.5914
31	C	2.0286	-2.0512	-3.5860
32	H	2.2093	-1.5762	-4.5505
33	H	1.9993	-3.1335	-3.7509
34	H	1.0322	-1.7578	-3.2417
35	C	2.9861	-2.0716	-1.2562
36	C	3.9579	-1.7511	-0.3027
37	C	3.8245	-2.2189	1.1131
38	H	2.8118	-2.0533	1.4929
39	H	4.0163	-3.2936	1.2011
40	H	4.5298	-1.7060	1.7681
41	H	5.8110	-0.7278	0.0082
42	C	5.1876	-0.5709	-2.0429
43	C	6.3880	0.2204	-2.4627
44	H	7.2889	-0.4007	-2.4594
45	H	6.2763	0.6244	-3.4704
46	H	6.5782	1.0530	-1.7798
47	Ir	0.2389	-0.3295	-0.1217
48	H	1.7767	-0.1442	-0.0957
49	H	0.4752	-0.2392	-1.6894
50	C	0.6759	-2.3472	-0.3515
51	P	0.0952	2.0136	-0.1838
52	C	-0.3999	2.7809	1.3977
53	C	1.6932	2.8121	-0.5921
54	C	-1.0333	2.7288	-1.4326
55	C	0.4956	2.7074	2.4739
56	C	-1.6755	3.3055	1.6134
57	C	2.0487	4.0629	-0.0771
58	C	2.5379	2.1918	-1.5187
59	C	-1.2374	4.1120	-1.5008
60	C	-1.6605	1.9025	-2.3662
61	C	0.1265	3.1638	3.7309
62	H	1.4898	2.2950	2.3185
63	C	-2.0417	3.7656	2.8753
64	H	-2.3911	3.3495	0.7976
65	C	3.2315	4.6754	-0.4756
66	H	1.4063	4.5595	0.6441
67	C	3.7123	2.8144	-1.9229
68	H	2.2757	1.2190	-1.9252
69	C	-2.0708	4.6511	-2.4708
70	H	-0.7418	4.7695	-0.7914
71	C	-2.4940	2.4446	-3.3394
72	H	-1.4803	0.8314	-2.3329
73	C	-1.1450	3.6965	3.9344
74	H	0.8316	3.1071	4.5538
75	H	-3.0339	4.1795	3.0267
76	C	4.0638	4.0545	-1.4007
77	H	3.5001	5.6426	-0.0642
78	H	4.3521	2.3258	-2.6506
79	C	-2.7032	3.8173	-3.3898
80	H	-2.2234	5.7242	-2.5149
81	H	-2.9741	1.7941	-4.0634
82	H	-1.4306	4.0617	4.9153
83	H	4.9829	4.5380	-1.7145
84	H	-3.3510	4.2412	-4.1497
85	C	0.6486	-0.6384	3.1120
86	C	-0.0365	-0.5647	4.3297
87	C	-1.3425	-0.3129	3.9860
88	C	-2.5254	0.0708	1.8245
89	C	-3.6131	0.7342	2.3935
90	C	-2.5443	-0.2531	0.4687
91	C	-4.7036	1.0730	1.6065
92	H	-3.5877	1.0254	3.4368
93	C	-3.6382	0.1019	-0.3135
94	H	-1.8063	-0.9429	0.0384
95	C	-4.7180	0.7696	0.2475

96	H	-5.5400	1.5960	2.0571
97	H	-3.6485	-0.1718	-1.3632
98	H	-5.5681	1.0452	-0.3661
99	N	-0.1746	-0.4523	2.0812
100	N	-1.4035	-0.2419	2.6282
101	H	-2.2283	-0.2004	4.5890
102	H	0.3628	-0.6858	5.3228
103	H	1.6980	-0.8089	2.9231

$Ir(H)_2(PhPz)(IMes)(PPh_3)$ C-H Activation TS1 ***Imaginary Frequency: -676.4 cm^{-1}***

1	Ir	0.1346	-0.3467	0.0199
2	H	1.2106	-0.8691	-1.0824
3	P	1.0848	1.8159	-0.2344
4	C	-0.0002	-4.6349	0.3410
5	C	-1.3183	-4.3997	0.5210
6	H	0.5692	-5.5493	0.3294
7	H	-2.1482	-5.0631	0.6985
8	N	0.6034	-3.4041	0.1518
9	N	-1.4919	-3.0280	0.4395
10	C	-0.3105	-2.3918	0.2039
11	H	1.4007	-0.6225	0.9493
12	C	-2.5744	1.0014	0.1273
13	C	-1.5341	0.7000	1.0336
14	C	-1.6840	1.2177	2.3277
15	C	-2.7969	1.9573	2.7132
16	C	-3.8115	2.2133	1.7991
17	C	-3.6934	1.7413	0.5017
18	H	-0.3598	-0.3243	1.5429
19	H	-0.9068	1.0350	3.0641
20	H	-2.8667	2.3316	3.7289
21	H	-4.6851	2.7879	2.0864
22	H	-4.4681	1.9605	-0.2258
23	C	-2.7712	-2.3939	0.6088
24	C	-3.1320	-1.9491	1.8880
25	C	-3.6271	-2.3017	-0.4895
26	C	-4.3753	-1.3447	2.0325
27	C	-4.8601	-1.6759	-0.2925
28	C	-5.2479	-1.1873	0.9526
29	H	-4.6669	-0.9745	3.0123
30	H	-5.5361	-1.5777	-1.1390
31	C	2.0227	-3.2540	-0.0318
32	C	2.8274	-3.0900	1.0996
33	C	2.5388	-3.2848	-1.3279
34	C	4.1969	-2.9389	0.8976
35	C	3.9165	-3.1278	-1.4767
36	C	4.7607	-2.9568	-0.3801
37	H	4.8420	-2.8091	1.7630
38	H	4.3397	-3.1418	-2.4781
39	C	1.6374	-3.4698	-2.5083
40	H	2.1822	-3.3484	-3.4449
41	H	1.1810	-4.4651	-2.5171
42	H	0.8149	-2.7471	-2.4927
43	C	6.2418	-2.8202	-0.5583
44	H	6.5104	-2.6413	-1.6008
45	H	6.6470	-2.0036	0.0449
46	H	6.7603	-3.7303	-0.2415
47	C	2.2317	-3.0732	2.4734
48	H	3.0004	-2.9370	3.2344
49	H	1.5010	-2.2646	2.5818
50	H	1.7017	-4.0044	2.6982
51	C	-3.2645	-2.8900	-1.8184
52	H	-3.5162	-3.9558	-1.8531
53	H	-3.8115	-2.4045	-2.6280
54	H	-2.1961	-2.8100	-2.0321
55	C	-2.2203	-2.1394	3.0602
56	H	-2.5882	-1.6015	3.9346

57	H	-2.1366	-3.1974	3.3317
58	H	-1.2027	-1.7942	2.8533
59	C	-6.5701	-0.5113	1.1422
60	H	-7.2399	-1.1159	1.7608
61	H	-6.4541	0.4489	1.6536
62	H	-7.0753	-0.3320	0.1917
63	C	0.0031	2.9104	-1.2092
64	C	0.0463	2.8574	-2.6077
65	C	-0.9944	3.6690	-0.5872
66	C	-0.8735	3.5696	-3.3664
67	H	0.8081	2.2606	-3.1024
68	C	-1.9140	4.3789	-1.3512
69	H	-1.0567	3.7016	0.4971
70	C	-1.8539	4.3335	-2.7399
71	H	-0.8235	3.5290	-4.4495
72	H	-2.6770	4.9707	-0.8557
73	H	-2.5671	4.8952	-3.3342
74	C	3.1595	3.0438	-1.6714
75	C	3.5453	0.7418	-1.0583
76	C	4.4228	3.1028	-2.2446
77	H	2.5209	3.9221	-1.6934
78	C	4.8119	0.8088	-1.6287
79	H	3.2102	-0.1829	-0.5994
80	C	5.2511	1.9847	-2.2251
81	H	4.7610	4.0239	-2.7068
82	H	5.4528	-0.0664	-1.6093
83	H	6.2375	2.0329	-2.6740
84	C	1.4237	2.7221	1.3179
85	C	1.5399	4.1167	1.3236
86	C	1.6753	2.0164	2.4971
87	C	1.8786	4.7886	2.4910
88	H	1.3600	4.6831	0.4148
89	C	2.0200	2.6915	3.6626
90	H	1.6054	0.9328	2.5001
91	C	2.1171	4.0780	3.6629
92	H	1.9601	5.8702	2.4836
93	H	2.2143	2.1320	4.5716
94	H	2.3834	4.6048	4.5729
95	C	2.7050	1.8581	-1.0816
96	N	-1.3068	-0.0526	-1.5778
97	C	-3.2242	0.8125	-2.3051
98	H	-4.1682	1.3248	-2.2228
99	C	-1.3839	-0.2545	-2.8932
100	H	-0.5769	-0.7573	-3.4039
101	C	-2.5798	0.2686	-3.3944
102	H	-2.9270	0.2593	-4.4142
103	N	-2.4329	0.6096	-1.2211

Ir(H)(H_2)(PhPz)(IMes)(PPh_3)

1	C	-3.5483	2.5845	-0.5544
2	C	-3.3730	1.3222	-1.1218
3	C	-3.2483	1.1235	-2.5025
4	C	-3.2561	2.2484	-3.3182
5	C	-3.3940	3.5370	-2.7945
6	C	-3.5432	3.6820	-1.4182
7	H	-3.6586	4.6770	-0.9952
8	C	-3.3728	4.7264	-3.7036
9	H	-2.4605	4.7466	-4.3084
10	H	-4.2105	4.7060	-4.4066
11	H	-3.4309	5.6638	-3.1486
12	C	-3.7366	2.7600	0.9198
13	H	-3.5912	3.8006	1.2118
14	H	-4.7461	2.4712	1.2319
15	H	-3.0365	2.1474	1.4944
16	C	-4.5961	-0.3811	0.1785
17	C	-4.2795	-1.4963	0.8713

18	H	-5.5439	0.0773	-0.0490
19	H	-4.8912	-2.2175	1.3869
20	C	-3.1132	-0.2549	-3.0712
21	H	-2.2238	-0.7614	-2.6806
22	H	-3.9706	-0.8854	-2.8125
23	H	-3.0367	-0.2260	-4.1590
24	H	-3.1466	2.1178	-4.3921
25	N	-3.4039	0.1630	-0.2713
26	N	-2.9023	-1.6107	0.8301
27	C	-1.1523	-4.8351	1.3124
28	C	-1.0003	-3.5600	3.3362
29	H	-0.9247	-5.7338	0.7452
30	C	-1.9157	-3.8351	0.7090
31	C	-2.4405	-3.9932	-0.6856
32	H	-1.9603	-4.8288	-1.1957
33	H	-3.5182	-4.1875	-0.6844
34	H	-2.2924	-3.0924	-1.2890
35	C	-2.1831	-2.6859	1.4567
36	C	-1.7518	-2.5292	2.7783
37	C	-2.0786	-1.2963	3.5623
38	H	-2.0750	-0.4004	2.9360
39	H	-3.0746	-1.3646	4.0132
40	H	-1.3652	-1.1477	4.3753
41	H	-0.6497	-3.4547	4.3605
42	C	-0.6780	-4.7131	2.6179
43	C	0.1703	-5.7803	3.2373
44	H	1.1981	-5.4312	3.3808
45	H	-0.2045	-6.0687	4.2225
46	H	0.2123	-6.6766	2.6175
47	Ir	-0.2570	-0.3230	-0.0956
48	H	-0.2277	-1.7582	-1.1769
49	C	-2.3350	-0.5850	0.1221
50	P	2.1228	-0.1956	0.0380
51	C	2.7784	-0.4497	1.7245
52	C	2.9912	-1.4619	-0.9588
53	C	2.8190	1.3924	-0.5384
54	C	2.1932	-1.4301	2.5348
55	C	3.9104	0.2263	2.1899
56	C	4.1539	-2.0897	-0.5038
57	C	2.4974	-1.7862	-2.2272
58	C	3.3407	1.5227	-1.8294
59	C	2.7262	2.5288	0.2757
60	C	2.7166	-1.7110	3.7899
61	H	1.3283	-1.9836	2.1811
62	C	4.4259	-0.0524	3.4517
63	H	4.3968	0.9690	1.5661
64	C	4.8039	-3.0238	-1.3032
65	H	4.5514	-1.8560	0.4787
66	C	3.1538	-2.7124	-3.0275
67	H	1.5974	-1.2984	-2.5922
68	C	3.7625	2.7630	-2.2949
69	H	3.4296	0.6534	-2.4731
70	C	3.1535	3.7644	-0.1933
71	H	2.3160	2.4503	1.2785
72	C	3.8294	-1.0170	4.2550
73	H	2.2523	-2.4751	4.4057
74	H	5.3001	0.4848	3.8034
75	C	4.3079	-3.3358	-2.5638
76	H	5.7023	-3.5090	-0.9373
77	H	2.7638	-2.9505	-4.0115
78	C	3.6695	3.8854	-1.4799
79	H	4.1720	2.8482	-3.2960
80	H	3.0796	4.6340	0.4518
81	H	4.2339	-1.2314	5.2384
82	H	4.8184	-4.0645	-3.1840
83	H	4.0062	4.8509	-1.8427

84	C	-0.3477	1.5448	0.7485
85	C	-0.3508	2.6344	-0.1383
86	C	-0.3597	1.8555	2.1132
87	C	-0.4259	3.9563	0.2906
88	C	-0.4071	3.1725	2.5632
89	H	-0.3091	1.0500	2.8397
90	C	-0.4557	4.2240	1.6519
91	H	-0.4503	4.7702	-0.4273
92	H	-0.4101	3.3785	3.6287
93	H	-0.5090	5.2503	1.9976
94	C	0.0190	1.0152	-3.1995
95	C	0.1662	2.3171	-3.6871
96	H	0.0512	0.0759	-3.7318
97	C	0.0145	3.1309	-2.5858
98	H	0.3566	2.6251	-4.7018
99	H	0.0592	4.2027	-2.4818
100	N	-0.2123	1.0255	-1.8849
101	H	-0.1156	-2.0752	-0.3893
102	H	-0.2272	-0.9704	1.3410
103	N	-0.2165	2.3314	-1.5163

2-Phenyloxazoline

2-PhOx

1	C	-1.4311	1.5908	0.8290
2	C	-0.0868	2.2902	-0.7528
3	C	-2.2774	2.0459	-0.3719
4	H	-1.5848	0.5351	1.0770
5	H	-1.6501	2.1601	1.7388
6	H	-2.9384	2.8892	-0.1557
7	H	-2.8705	1.2475	-0.8254
8	N	-0.0463	1.8095	0.4288
9	C	1.0733	2.6666	-1.5605
10	C	2.3572	2.5026	-1.0308
11	C	0.9170	3.1851	-2.8483
12	C	3.4678	2.8537	-1.7820
13	H	2.4553	2.0984	-0.0292
14	C	2.0342	3.5352	-3.5963
15	H	-0.0813	3.3092	-3.2516
16	C	3.3093	3.3708	-3.0662
17	H	4.4623	2.7251	-1.3677
18	H	1.9091	3.9377	-4.5961
19	H	4.1805	3.6450	-3.6524
20	O	-1.3014	2.4809	-1.3394

$Ir(H)_2(CHCl_3)(PhOx)(IMes)(PPh_3)$

1	C	2.8446	-3.2405	0.6752
2	C	1.5013	-3.3303	1.0668
3	C	1.1171	-3.4918	2.3997
4	C	2.1248	-3.4742	3.3676
5	C	3.4716	-3.3638	3.0286
6	C	3.8112	-3.2703	1.6754
7	H	4.8603	-3.2201	1.3914
8	C	4.5386	-3.3516	4.0795
9	H	5.2834	-4.1314	3.8996
10	H	5.0793	-2.3996	4.0874
11	H	4.1271	-3.5053	5.0774
12	C	3.2231	-3.1536	-0.7714
13	H	4.2638	-2.8454	-0.8865
14	H	3.1123	-4.1221	-1.2710
15	H	2.5908	-2.4473	-1.3173
16	C	0.1151	-4.5238	-0.5934
17	C	-0.7569	-4.1699	-1.5623
18	H	0.4985	-5.4833	-0.2885
19	H	-1.2955	-4.7565	-2.2878
20	C	-0.3097	-3.7419	2.7783
21	H	-0.5287	-4.8153	2.7543
22	H	-0.5202	-3.3937	3.7917
23	H	-1.0122	-3.2571	2.0975
24	H	1.8455	-3.5740	4.4134
25	N	0.5022	-3.3526	0.0383
26	N	-0.8858	-2.7950	-1.5008
27	C	-3.7012	-0.9873	-3.0938
28	C	-1.7666	-0.8276	-4.5026
29	H	-4.7452	-0.7550	-2.8974
30	C	-3.0026	-1.7628	-2.1713
31	C	-3.6650	-2.2960	-0.9389
32	H	-4.5844	-1.7508	-0.7169
33	H	-3.9342	-3.3511	-1.0588
34	H	-3.0086	-2.2310	-0.0675
35	C	-1.6602	-2.0376	-2.4461
36	C	-1.0221	-1.5975	-3.6088
37	C	0.4118	-1.9410	-3.8701
38	H	0.7459	-1.5459	-4.8297
39	H	1.0667	-1.5297	-3.0936
40	H	0.5730	-3.0240	-3.8770
41	H	-1.2875	-0.4716	-5.4120
42	C	-3.1031	-0.5094	-4.2619
43	C	-3.8928	0.3121	-5.2347
44	H	-3.2749	0.6684	-6.0607
45	H	-4.7139	-0.2673	-5.6667
46	H	-4.3462	1.1817	-4.7498
47	Ir	-0.1179	-0.2367	-0.0332
48	H	-0.1109	-0.0553	-1.5711
49	H	-1.6824	-0.4190	-0.1530
50	C	-0.1100	-2.2580	-0.5052
51	P	-0.5190	2.0723	0.1842
52	C	-2.0839	2.5918	0.9978
53	C	0.8113	2.9071	1.1149
54	C	-0.6748	2.9741	-1.4051
55	C	-2.1955	3.8140	1.6720
56	C	-3.2247	1.7933	0.8533
57	C	0.7642	2.9918	2.5120
58	C	2.0067	3.2363	0.4628
59	C	-0.2907	4.3105	-1.5464
60	C	-1.2823	2.3191	-2.4826
61	C	-3.4073	4.2003	2.2317
62	H	-1.3329	4.4667	1.7642
63	C	-4.4367	2.1862	1.4105
64	H	-3.1566	0.8526	0.3144
65	C	1.8737	3.4171	3.2346

66	H	-0.1442	2.7248	3.0450
67	C	3.1164	3.6551	1.1869
68	H	2.0721	3.1644	-0.6198
69	C	-0.4793	4.9692	-2.7563
70	H	0.1616	4.8402	-0.7133
71	C	-1.4787	2.9862	-3.6859
72	H	-1.5969	1.2832	-2.3821
73	C	-4.5278	3.3841	2.1103
74	H	-3.4768	5.1445	2.7611
75	H	-5.3086	1.5496	1.3010
76	C	3.0528	3.7484	2.5746
77	H	1.8149	3.4890	4.3155
78	H	4.0321	3.9124	0.6639
79	C	-1.0700	4.3086	-3.8282
80	H	-0.1679	6.0033	-2.8588
81	H	-1.9460	2.4654	-4.5158
82	H	-5.4708	3.6866	2.5526
83	H	3.9168	4.0836	3.1386
84	H	-1.2169	4.8264	-4.7701
85	Cl	-0.1640	-0.4836	2.7350
86	C	-1.8331	-0.0307	3.2971
87	H	-2.0507	0.9334	2.8420
88	Cl	-3.0267	-1.1872	2.7181
89	C	3.0723	0.3339	-2.1128
90	C	4.0747	-0.2320	-2.9087
91	C	2.0575	1.0822	-2.7139
92	C	4.0327	-0.0812	-4.2879
93	H	4.8810	-0.7856	-2.4407
94	C	2.0299	1.2470	-4.0918
95	H	1.3010	1.5491	-2.0976
96	C	3.0128	0.6581	-4.8812
97	H	4.8054	-0.5310	-4.9016
98	H	1.2419	1.8424	-4.5421
99	H	2.9931	0.7841	-5.9587
100	N	2.1557	0.0367	0.1737
101	C	2.7397	0.0320	1.5260
102	C	4.2460	0.1282	1.2955
103	H	2.4566	-0.8876	2.0496
104	H	4.7975	-0.7597	1.6122
105	C	3.1318	0.1912	-0.6565
106	O	4.3729	0.2398	-0.1422
107	H	2.3445	0.8794	2.0952
108	H	4.7053	1.0145	1.7366
109	Cl	-1.8396	0.1308	5.0421

Ir(H)$_2$(DCM)(PhOx)(IMes)(PPh$_3$)

1	C	-4.0059	-2.3474	0.8188
2	C	-3.2405	-2.4108	-0.3480
3	C	-3.7589	-2.0596	-1.6046
4	C	-5.0713	-1.5964	-1.6581
5	C	-5.8647	-1.4884	-0.5115
6	C	-5.3128	-1.8663	0.7100
7	H	-5.9180	-1.7938	1.6102
8	C	-7.2738	-0.9919	-0.6029
9	H	-7.3387	-0.0805	-1.2024
10	H	-7.9212	-1.7326	-1.0822
11	H	-7.6901	-0.7770	0.3821
12	C	-3.4683	-2.7627	2.1537
13	H	-2.6321	-3.4596	2.0688
14	H	-3.1192	-1.8925	2.7195
15	H	-4.2449	-3.2404	2.7538
16	C	-1.7339	-4.3459	-0.4743
17	C	-0.4026	-4.5497	-0.5539
18	H	-2.5683	-5.0260	-0.5192
19	H	0.1772	-5.4483	-0.6846
20	C	-2.9299	-2.1929	-2.8437

21	H	-3.5027	-1.9210	-3.7304
22	H	-2.0445	-1.5515	-2.7975
23	H	-2.5686	-3.2177	-2.9788
24	H	-5.4887	-1.3203	-2.6233
25	N	-1.9207	-2.9814	-0.3101
26	N	0.1998	-3.3083	-0.4386
27	C	3.4647	-2.6446	-2.0545
28	C	3.8530	-3.2463	0.2291
29	H	3.8455	-2.3630	-3.0339
30	C	2.0891	-2.7958	-1.8907
31	C	1.1482	-2.6182	-3.0434
32	H	0.5983	-3.5418	-3.2532
33	H	0.3983	-1.8461	-2.8452
34	H	1.6887	-2.3431	-3.9503
35	C	1.6190	-3.1408	-0.6150
36	C	2.4843	-3.3970	0.4516
37	C	1.9859	-3.8602	1.7864
38	H	0.9528	-3.5589	1.9693
39	H	2.0152	-4.9530	1.8579
40	H	2.6141	-3.4749	2.5933
41	H	4.5377	-3.4183	1.0566
42	C	4.3629	-2.8613	-1.0083
43	C	5.8375	-2.6960	-1.2067
44	H	6.3038	-2.2150	-0.3433
45	H	6.3293	-3.6652	-1.3363
46	H	6.0625	-2.0982	-2.0919
47	Ir	-0.2706	-0.2864	-0.1362
48	H	0.9013	-0.6854	-1.0526
49	H	-1.1054	-0.0258	-1.4656
50	C	-0.7299	-2.3082	-0.2794
51	P	0.4311	1.9535	-0.3197
52	C	-0.9607	3.1213	-0.5900
53	C	1.3110	2.6190	1.1447
54	C	1.5048	2.3559	-1.7491
55	C	-1.5280	3.8822	0.4345
56	C	-1.5443	3.1524	-1.8638
57	C	0.7348	2.3981	2.4028
58	C	2.5516	3.2581	1.0779
59	C	1.7438	3.7006	-2.0646
60	C	2.0787	1.3670	-2.5500
61	C	-2.6655	4.6487	0.1927
62	H	-1.0852	3.8843	1.4249
63	C	-2.6712	3.9286	-2.1039
64	H	-1.1128	2.5641	-2.6694
65	C	1.3726	2.8207	3.5620
66	H	-0.2210	1.8837	2.4707
67	C	3.1907	3.6767	2.2403
68	H	3.0364	3.4127	0.1200
69	C	2.5539	4.0421	-3.1388
70	H	1.2860	4.4843	-1.4666
71	C	2.8872	1.7103	-3.6292
72	H	1.8871	0.3205	-2.3397
73	C	-3.2401	4.6727	-1.0726
74	H	-3.1004	5.2305	0.9985
75	H	-3.1075	3.9510	-3.0970
76	C	2.6050	3.4624	3.4823
77	H	0.9075	2.6497	4.5280
78	H	4.1574	4.1648	2.1716
79	C	3.1302	3.0462	-3.9229
80	H	2.7307	5.0873	-3.3688
81	H	3.3208	0.9282	-4.2450
82	H	-4.1252	5.2718	-1.2573
83	H	3.1077	3.7900	4.3861
84	H	3.7581	3.3140	-4.7660
85	Cl	-2.4237	0.5714	1.4085
86	C	-3.6540	1.1121	0.1896

87	H	-3.1947	1.9193	-0.3736
88	H	-3.8712	0.2403	-0.4226
89	Cl	-5.1183	1.6886	0.9618
90	C	3.2451	-0.0551	1.7511
91	C	4.2869	0.3791	2.5812
92	C	3.3994	0.0156	0.3643
93	C	5.4479	0.8984	2.0262
94	H	4.1715	0.3216	3.6567
95	C	4.5614	0.5355	-0.1850
96	H	2.6167	-0.3726	-0.2743
97	C	5.5856	0.9848	0.6439
98	H	6.2467	1.2406	2.6752
99	H	4.6692	0.5801	-1.2646
100	H	6.4948	1.3919	0.2135
101	N	0.8439	-0.7157	1.8377
102	C	-0.0404	-1.1837	2.9130
103	C	0.8819	-1.4185	4.1044
104	H	-0.5846	-2.0784	2.5925
105	H	1.0180	-2.4730	4.3579
106	C	2.0208	-0.5681	2.3583
107	O	2.1509	-0.9047	3.6564
108	H	-0.7918	-0.4141	3.1267
109	H	0.5969	-0.8739	5.0057

$Ir(H)_2(PhOx)_2(IMes)(PPh_3)$

1	C	2.4613	3.0736	-1.0416
2	C	1.3235	2.7342	-1.7861
3	C	1.4068	2.2210	-3.0795
4	C	2.6815	2.0374	-3.6219
5	C	3.8387	2.3405	-2.9108
6	C	3.7058	2.8630	-1.6219
7	H	4.5969	3.1123	-1.0510
8	C	5.1975	2.1138	-3.4990
9	H	5.7778	1.4082	-2.8949
10	H	5.1406	1.7164	-4.5138
11	H	5.7767	3.0405	-3.5378
12	C	2.3279	3.6132	0.3468
13	H	3.3040	3.8266	0.7839
14	H	1.7325	4.5320	0.3737
15	H	1.8194	2.8866	0.9905
16	C	-0.6331	4.1724	-1.3457
17	C	-1.7107	4.1326	-0.5349
18	H	-0.2744	4.9313	-2.0211
19	H	-2.4921	4.8502	-0.3469
20	C	0.1909	1.8263	-3.8544
21	H	-0.6669	2.4662	-3.6326
22	H	0.3768	1.8659	-4.9293
23	H	-0.1032	0.7979	-3.6111
24	H	2.7635	1.6404	-4.6312
25	N	0.0392	2.9734	-1.1792
26	N	-1.6785	2.9067	0.1165
27	C	-4.6984	2.0009	2.0968
28	C	-2.9147	2.5615	3.5866
29	H	-5.7339	1.7124	1.9295
30	C	-3.8985	2.2749	0.9853
31	C	-4.5122	2.2670	-0.3797
32	H	-5.1568	3.1440	-0.5026
33	H	-3.7742	2.2929	-1.1789
34	H	-5.1474	1.3905	-0.5303
35	C	-2.5626	2.6218	1.2145
36	C	-2.0721	2.8354	2.5106
37	C	-0.6977	3.3895	2.7285
38	H	0.0786	2.7161	2.3538
39	H	-0.5655	4.3371	2.1966
40	H	-0.5144	3.5778	3.7876
41	H	-2.5428	2.7227	4.5964

42	C	-4.2239	2.1129	3.4018
43	C	-5.0941	1.7676	4.5703
44	H	-4.8228	2.3376	5.4606
45	H	-6.1488	1.9509	4.3576
46	H	-5.0008	0.7070	4.8300
47	Ir	-0.0565	0.1730	0.0350
48	H	-0.3589	0.0373	-1.5036
49	H	1.4017	0.6462	-0.2875
50	C	-0.5887	2.1584	-0.2701
51	P	0.6326	-2.0653	-0.1882
52	C	1.5832	-2.8157	1.1933
53	C	-0.7322	-3.2520	-0.5096
54	C	1.7159	-2.3606	-1.6375
55	C	0.9918	-2.8658	2.4601
56	C	2.9118	-3.2306	1.0648
57	C	-1.1249	-4.2588	0.3752
58	C	-1.4233	-3.1056	-1.7182
59	C	1.9177	-3.6674	-2.0999
60	C	2.3881	-1.3077	-2.2625
61	C	1.6989	-3.3118	3.5679
62	H	-0.0420	-2.5523	2.5744
63	C	3.6251	-3.6718	2.1751
64	H	3.4039	-3.1915	0.0979
65	C	-2.1942	-5.0946	0.0609
66	H	-0.5829	-4.4187	1.3009
67	C	-2.4762	-3.9524	-2.0363
68	H	-1.1288	-2.3257	-2.4181
69	C	2.7791	-3.9139	-3.1602
70	H	1.3966	-4.4951	-1.6258
71	C	3.2558	-1.5597	-3.3219
72	H	2.2261	-0.2880	-1.9261
73	C	3.0253	-3.7104	3.4276
74	H	1.2169	-3.3489	4.5402
75	H	4.6579	-3.9823	2.0576
76	C	-2.8715	-4.9443	-1.1429
77	H	-2.4824	-5.8785	0.7537
78	H	-2.9905	-3.8341	-2.9844
79	C	3.4534	-2.8596	-3.7713
80	H	2.9250	-4.9300	-3.5110
81	H	3.7714	-0.7310	-3.7979
82	H	3.5862	-4.0514	4.2910
83	H	-3.6946	-5.6058	-1.3918
84	H	4.1268	-3.0543	-4.5994
85	C	-2.6430	-0.9078	1.7018
86	C	-3.9999	-1.5908	1.5739
87	N	-2.2912	-0.4934	0.3395
88	C	-0.0824	0.1987	3.4571
89	C	0.7738	0.6344	4.6378
90	N	0.7945	0.3238	2.2818
91	C	3.2715	0.7231	2.0565
92	C	3.5944	0.0430	0.8805
93	C	4.2346	1.5572	2.6427
94	C	4.8432	0.1967	0.2958
95	H	2.8756	-0.6261	0.4360
96	C	5.4757	1.7239	2.0456
97	H	3.9990	2.0743	3.5654
98	C	5.7851	1.0423	0.8715
99	H	5.0752	-0.3518	-0.6125
100	H	6.2086	2.3785	2.5047
101	H	6.7611	1.1654	0.4127
102	C	-3.5199	-0.5734	-1.8438
103	C	-2.7988	0.3625	-2.5919
104	C	-4.5073	-1.3380	-2.4806
105	C	-3.0465	0.5097	-3.9501
106	H	-2.0615	0.9851	-2.1005
107	C	-4.7454	-1.1907	-3.8392

108	H	-5.0742	-2.0568	-1.9017
109	C	-4.0128	-0.2692	-4.5793
110	H	-2.4904	1.2444	-4.5219
111	H	-5.5073	-1.7945	-4.3202
112	H	-4.2008	-0.1497	-5.6410
113	H	-4.7754	-1.1687	2.2161
114	H	-2.6710	-0.0260	2.3517
115	H	-0.9739	0.8116	3.3303
116	H	0.5190	1.6262	5.0244
117	C	1.9836	0.5586	2.7431
118	C	-3.3105	-0.7708	-0.4064
119	O	2.0988	0.6992	4.0808
120	O	-4.3722	-1.3398	0.2061
121	H	-0.4147	-0.8388	3.5547
122	H	0.7938	-0.0720	5.4685
123	H	-3.9630	-2.6739	1.7180
124	H	-1.8702	-1.5842	2.0817

Ir(H)$_2$(PhOx)(IMes)(PPh$_3$)

1	Ir	0.3681	-0.2805	0.0796
2	H	1.7570	-0.1916	-0.5978
3	P	0.3342	2.0621	0.1362
4	C	1.8548	-4.2829	0.3387
5	C	0.5369	-4.5783	0.3812
6	H	2.7293	-4.9109	0.3786
7	H	0.0149	-5.5164	0.4717
8	N	1.9535	-2.9078	0.2196
9	N	-0.1426	-3.3740	0.2834
10	C	0.7199	-2.3180	0.1814
11	H	1.2508	-0.1930	1.4041
12	C	-3.0616	0.0583	-0.5732
13	C	-2.5548	-0.0243	0.7280
14	C	-3.3154	0.3894	1.8122
15	C	-4.6013	0.8780	1.6097
16	C	-5.1313	0.9302	0.3218
17	C	-4.3691	0.5251	-0.7627
18	H	-1.5982	-0.5154	0.9474
19	H	-2.9070	0.3169	2.8144
20	H	-5.1946	1.2061	2.4565
21	H	-6.1392	1.2982	0.1641
22	H	-4.7615	0.5887	-1.7712
23	C	-1.5735	-3.2775	0.3589
24	C	-2.1587	-3.0728	1.6145
25	C	-2.3330	-3.4243	-0.8044
26	C	-3.5479	-3.0112	1.6792
27	C	-3.7200	-3.3265	-0.6893
28	C	-4.3460	-3.1258	0.5399
29	H	-4.0193	-2.8398	2.6439
30	H	-4.3261	-3.4106	-1.5886
31	C	3.2103	-2.2098	0.1833
32	C	3.8109	-1.8517	1.3920
33	C	3.7830	-1.9190	-1.0588
34	C	5.0275	-1.1714	1.3290
35	C	4.9974	-1.2390	-1.0677
36	C	5.6374	-0.8605	0.1144
37	H	5.5118	-0.8805	2.2581
38	H	5.4582	-1.0017	-2.0233
39	C	3.0940	-2.3131	-2.3281
40	H	3.6721	-2.0128	-3.2023
41	H	2.9342	-3.3945	-2.3868
42	H	2.1057	-1.8457	-2.3957
43	C	6.9601	-0.1589	0.0702
44	H	7.2211	0.2769	1.0362
45	H	7.7627	-0.8524	-0.1992
46	H	6.9689	0.6374	-0.6785
47	C	3.1672	-2.1865	2.7013

48	H	3.7153	-1.7451	3.5340
49	H	2.1353	-1.8236	2.7400
50	H	3.1282	-3.2681	2.8672
51	C	-1.6921	-3.7038	-2.1285
52	H	-1.6292	-4.7814	-2.3145
53	H	-2.2775	-3.2773	-2.9464
54	H	-0.6732	-3.3148	-2.1830
55	C	-1.3103	-2.8618	2.8309
56	H	-0.5743	-3.6602	2.9659
57	H	-0.7360	-1.9283	2.7560
58	H	-1.9203	-2.8090	3.7333
59	C	-5.8377	-3.0445	0.6384
60	H	-6.2827	-2.6719	-0.2863
61	H	-6.2727	-4.0306	0.8303
62	H	-6.1521	-2.3918	1.4554
63	C	-0.5462	2.7980	-1.2862
64	C	0.1267	2.9523	-2.5045
65	C	-1.9254	3.0265	-1.2432
66	C	-0.5621	3.3427	-3.6471
67	H	1.1985	2.7766	-2.5540
68	C	-2.6134	3.4099	-2.3899
69	H	-2.4667	2.9054	-0.3088
70	C	-1.9347	3.5714	-3.5929
71	H	-0.0242	3.4776	-4.5802
72	H	-3.6827	3.5886	-2.3383
73	H	-2.4707	3.8822	-4.4835
74	C	2.1155	4.2068	-0.2210
75	C	3.1201	2.1274	0.4785
76	C	3.3590	4.8225	-0.1792
77	H	1.2426	4.7825	-0.5163
78	C	4.3629	2.7510	0.5256
79	H	3.0325	1.0771	0.7393
80	C	4.4852	4.0954	0.1963
81	H	3.4492	5.8723	-0.4368
82	H	5.2353	2.1797	0.8253
83	H	5.4553	4.5797	0.2331
84	C	-0.4500	2.7944	1.6204
85	C	-0.9694	4.0933	1.6262
86	C	-0.4746	2.0487	2.8027
87	C	-1.5222	4.6232	2.7853
88	H	-0.9509	4.6924	0.7206
89	C	-1.0173	2.5861	3.9643
90	H	-0.0619	1.0427	2.8062
91	C	-1.5488	3.8706	3.9556
92	H	-1.9280	5.6292	2.7763
93	H	-1.0251	2.0000	4.8777
94	H	-1.9765	4.2893	4.8603
95	C	1.9851	2.8512	0.1034
96	N	-0.9456	-0.3933	-1.7091
97	C	-0.4929	-0.5306	-3.0936
98	C	-1.7642	-0.3198	-3.9194
99	H	0.2768	0.2191	-3.3016
100	H	-2.0025	-1.1384	-4.5995
101	C	-2.2258	-0.2266	-1.7297
102	O	-2.8222	-0.2454	-2.9300
103	H	-1.7692	0.6220	-4.4734
104	H	-0.0397	-1.5158	-3.2445

Ir(H)$_2$(PhOx)(IMes)(PPh$_3$) C-C Rotation TS2 ***Imaginary Frequency: -51.2 cm^{-1}***

1	Ir	0.2884	-0.2665	0.1974
2	H	1.3625	0.0010	-0.8912
3	P	0.4435	2.0711	0.3826
4	C	1.8423	-4.1906	-0.2788
5	C	0.5676	-4.5448	-0.0194
6	H	2.7258	-4.7830	-0.4481
7	H	0.1095	-5.5106	0.1051

8	N	1.8801	-2.8078	-0.2903
9	N	-0.1621	-3.3671	0.1116
10	C	0.6454	-2.2716	-0.0415
11	H	1.4527	-0.3348	1.2699
12	C	-3.4880	0.4464	-0.2869
13	C	-3.7085	1.6988	0.2875
14	C	-4.6401	1.8421	1.3077
15	C	-5.3669	0.7422	1.7496
16	C	-5.1612	-0.5029	1.1668
17	C	-4.2268	-0.6535	0.1492
18	H	-3.1605	2.5675	-0.0573
19	H	-4.8012	2.8194	1.7492
20	H	-6.1024	0.8584	2.5384
21	H	-5.7364	-1.3613	1.4974
22	H	-4.0732	-1.6231	-0.3157
23	C	-1.5582	-3.4056	0.4427
24	C	-2.0314	-2.7712	1.6000
25	C	-2.4037	-4.1937	-0.3537
26	C	-3.3251	-3.0671	2.0235
27	C	-3.7006	-4.4290	0.1006
28	C	-4.1676	-3.9167	1.3101
29	H	-3.6894	-2.5986	2.9341
30	H	-4.3594	-5.0435	-0.5084
31	C	3.1032	-2.0636	-0.4382
32	C	3.9135	-1.8998	0.6890
33	C	3.4529	-1.5530	-1.6929
34	C	5.1081	-1.1969	0.5310
35	C	4.6461	-0.8412	-1.7924
36	C	5.4909	-0.6579	-0.6963
37	H	5.7510	-1.0606	1.3972
38	H	4.9302	-0.4311	-2.7587
39	C	2.5931	-1.7861	-2.8960
40	H	2.7849	-1.0401	-3.6695
41	H	2.7886	-2.7698	-3.3370
42	H	1.5313	-1.7552	-2.6453
43	C	6.7731	0.1022	-0.8446
44	H	7.3171	0.1676	0.0992
45	H	7.4344	-0.3730	-1.5745
46	H	6.5936	1.1211	-1.2010
47	C	3.5066	-2.4506	2.0198
48	H	4.1814	-2.1149	2.8074
49	H	2.4923	-2.1354	2.2843
50	H	3.5098	-3.5454	2.0261
51	C	-1.9627	-4.7677	-1.6688
52	H	-1.5664	-5.7833	-1.5712
53	H	-2.8090	-4.8293	-2.3552
54	H	-1.1827	-4.1662	-2.1417
55	C	-1.2270	-1.7531	2.3449
56	H	-0.1848	-2.0378	2.4969
57	H	-1.2556	-0.7761	1.8084
58	H	-1.6688	-1.5355	3.3197
59	C	-5.5461	-4.2379	1.7989
60	H	-6.3066	-3.9819	1.0550
61	H	-5.6558	-5.3074	1.9996
62	H	-5.7828	-3.7058	2.7217
63	C	0.3557	2.7721	-1.3130
64	C	1.5139	2.8015	-2.0990
65	C	-0.8655	3.1023	-1.9098
66	C	1.4492	3.1565	-3.4419
67	H	2.4741	2.5479	-1.6580
68	C	-0.9279	3.4642	-3.2511
69	H	-1.7789	3.0876	-1.3250
70	C	0.2292	3.4878	-4.0232
71	H	2.3584	3.1838	-4.0334
72	H	-1.8844	3.7318	-3.6895
73	H	0.1822	3.7740	-5.0685

74	C	2.2248	3.9331	1.5321
75	C	3.2103	1.8476	0.8322
76	C	3.4751	4.3885	1.9331
77	H	1.3599	4.5747	1.6650
78	C	4.4597	2.3105	1.2300
79	H	3.1151	0.8507	0.4125
80	C	4.5954	3.5790	1.7829
81	H	3.5715	5.3776	2.3679
82	H	5.3270	1.6681	1.1156
83	H	5.5698	3.9345	2.1007
84	C	-0.7414	3.0217	1.3935
85	C	-1.1567	4.3210	1.0831
86	C	-1.2042	2.4220	2.5688
87	C	-2.0304	4.9972	1.9281
88	H	-0.7935	4.8082	0.1829
89	C	-2.0627	3.1055	3.4200
90	H	-0.8796	1.4143	2.8182
91	C	-2.4818	4.3922	3.0973
92	H	-2.3490	6.0039	1.6786
93	H	-2.4107	2.6318	4.3319
94	H	-3.1570	4.9258	3.7581
95	C	2.0783	2.6567	0.9794
96	N	-1.3396	-0.1152	-1.4020
97	C	-0.8883	-0.2134	-2.7984
98	C	-2.1263	0.1326	-3.6254
99	H	-0.0551	0.4794	-2.9615
100	H	-2.5149	-0.6982	-4.2178
101	C	-2.5699	0.2597	-1.4280
102	O	-3.1293	0.4600	-2.6297
103	H	-2.0001	1.0032	-4.2699
104	H	-0.5184	-1.2261	-2.9859

Ir(H)$_2$(PhOx)(IMes)(PPh$_3$) C-H Activation TS1 ***Imaginary Frequency: -689.5 cm^{-1}***

1	Ir	-0.1471	-0.3384	-0.0287
2	H	-1.2001	-0.5626	1.1984
3	P	-0.6682	1.9673	0.0511
4	C	-1.0015	-4.5168	-0.5975
5	C	0.3043	-4.6585	-0.2831
6	H	-1.7553	-5.2366	-0.8695
7	H	0.9377	-5.5276	-0.2190
8	N	-1.2868	-3.1647	-0.5182
9	N	0.7887	-3.3878	-0.0204
10	C	-0.1825	-2.4431	-0.1584
11	H	-1.5128	-0.4123	-0.8356
12	C	2.7660	0.3850	-0.5140
13	C	1.5986	0.2328	-1.3053
14	C	1.7180	0.5818	-2.6572
15	C	2.9212	1.0251	-3.1993
16	C	4.0605	1.1378	-2.4062
17	C	3.9754	0.8265	-1.0595
18	H	0.1565	-0.4748	-1.5910
19	H	0.8545	0.5104	-3.3131
20	H	2.9668	1.2789	-4.2536
21	H	4.9966	1.4796	-2.8329
22	H	4.8329	0.9392	-0.4020
23	C	2.1237	-3.1428	0.4454
24	C	3.1287	-2.8215	-0.4676
25	C	2.3591	-3.2649	1.8194
26	C	4.3988	-2.5579	0.0471
27	C	3.6443	-2.9944	2.2802
28	C	4.6731	-2.6213	1.4117
29	H	5.1925	-2.2814	-0.6431
30	H	3.8499	-3.0816	3.3450
31	C	-2.6031	-2.6331	-0.7530
32	C	-2.9476	-2.2339	-2.0490
33	C	-3.4798	-2.5240	0.3274

34	C	-4.2085	-1.6754	-2.2371
35	C	-4.7367	-1.9698	0.0822
36	C	-5.1151	-1.5277	-1.1840
37	H	-4.4931	-1.3494	-3.2347
38	H	-5.4367	-1.8792	0.9094
39	C	-3.0732	-2.9530	1.7022
40	H	-2.6789	-3.9737	1.7155
41	H	-2.2821	-2.3037	2.0935
42	H	-3.9151	-2.9094	2.3935
43	C	-6.4525	-0.8952	-1.4141
44	H	-6.3500	0.1761	-1.6151
45	H	-6.9603	-1.3304	-2.2782
46	H	-7.1071	-1.0067	-0.5485
47	C	-1.9781	-2.3768	-3.1810
48	H	-2.4010	-1.9994	-4.1127
49	H	-1.0491	-1.8287	-2.9847
50	H	-1.6919	-3.4203	-3.3447
51	C	1.2598	-3.6751	2.7515
52	H	0.9665	-4.7181	2.5933
53	H	1.5688	-3.5812	3.7938
54	H	0.3552	-3.0755	2.6034
55	C	2.8774	-2.7773	-1.9433
56	H	3.1076	-3.7439	-2.4045
57	H	1.8379	-2.5449	-2.1831
58	H	3.5105	-2.0296	-2.4263
59	C	6.0270	-2.2564	1.9348
60	H	6.1444	-1.1672	1.9656
61	H	6.1881	-2.6322	2.9466
62	H	6.8258	-2.6432	1.2987
63	C	0.5462	2.9299	1.0087
64	C	0.3506	3.1552	2.3760
65	C	1.7695	3.2888	0.4291
66	C	1.3513	3.7439	3.1416
67	H	-0.5915	2.8774	2.8408
68	C	2.7694	3.8700	1.1998
69	H	1.9412	3.1136	-0.6292
70	C	2.5622	4.1013	2.5559
71	H	1.1807	3.9302	4.1971
72	H	3.7118	4.1437	0.7365
73	H	3.3400	4.5645	3.1538
74	C	-2.5935	3.7188	1.0736
75	C	-3.3041	1.4212	0.8745
76	C	-3.8598	4.0725	1.5181
77	H	-1.8275	4.4830	0.9723
78	C	-4.5754	1.7835	1.3085
79	H	-3.0943	0.3866	0.6243
80	C	-4.8539	3.1047	1.6360
81	H	-4.0735	5.1063	1.7671
82	H	-5.3476	1.0251	1.3922
83	H	-5.8442	3.3844	1.9793
84	C	-0.7726	2.7692	-1.5924
85	C	-0.3695	4.0894	-1.8109
86	C	-1.3676	2.0599	-2.6416
87	C	-0.5242	4.6730	-3.0637
88	H	0.0680	4.6692	-1.0046
89	C	-1.5306	2.6501	-3.8882
90	H	-1.7078	1.0404	-2.4803
91	C	-1.0991	3.9546	-4.1054
92	H	-0.1988	5.6956	-3.2220
93	H	-1.9953	2.0893	-4.6927
94	H	-1.2196	4.4130	-5.0810
95	C	-2.3009	2.3856	0.7596
96	N	1.4949	-0.1596	1.4238
97	C	1.6235	-0.1276	2.8752
98	H	0.9096	0.5893	3.2948
99	C	3.0843	0.2941	3.0913

100	H	3.1937	1.2495	3.6079
101	C	2.6252	0.1956	0.9087
102	O	3.6227	0.4656	1.7514
103	H	1.3977	-1.1074	3.3046
104	H	3.6911	-0.4628	3.5921

Ir(H)(H_2)(PhOx)(IMes)(PPh_3)

1	C	0.3611	3.6494	2.4069
2	C	0.6399	3.5925	1.0321
3	C	1.9017	3.8844	0.5123
4	C	2.9118	4.2222	1.4202
5	C	2.6790	4.3043	2.7897
6	C	1.3940	4.0159	3.2622
7	H	1.1917	4.0822	4.3290
8	C	3.7701	4.6796	3.7439
9	H	4.6963	4.9277	3.2248
10	H	3.4859	5.5412	4.3536
11	H	3.9874	3.8634	4.4403
12	C	-1.0092	3.3420	2.9286
13	H	-0.9915	3.1699	4.0060
14	H	-1.7062	4.1659	2.7416
15	H	-1.4383	2.4571	2.4484
16	C	-1.3229	4.3701	-0.2201
17	C	-2.2692	3.8174	-1.0074
18	H	-1.1684	5.3818	0.1159
19	H	-3.1230	4.2434	-1.5074
20	C	2.1870	3.9001	-0.9596
21	H	1.3395	3.5663	-1.5596
22	H	2.4450	4.9106	-1.2899
23	H	3.0454	3.2672	-1.2040
24	H	3.9018	4.4534	1.0347
25	N	-0.4618	3.3467	0.1425
26	N	-1.9700	2.4710	-1.1097
27	C	-3.3291	0.3074	-3.7956
28	C	-4.8126	0.2478	-1.9142
29	H	-3.0814	0.0053	-4.8106
30	C	-2.4865	1.2028	-3.1403
31	C	-1.2971	1.7937	-3.8324
32	H	-1.0610	1.2471	-4.7464
33	H	-1.4868	2.8349	-4.1152
34	H	-0.4084	1.7941	-3.1969
35	C	-2.8213	1.5610	-1.8305
36	C	-3.9901	1.1208	-1.2036
37	C	-4.3363	1.5563	0.1870
38	H	-4.6256	2.6120	0.2218
39	H	-5.1684	0.9740	0.5839
40	H	-3.4878	1.4344	0.8683
41	H	-5.7209	-0.1151	-1.4394
42	C	-4.4901	-0.1878	-3.1985
43	C	-5.3548	-1.1796	-3.9135
44	H	-5.3206	-1.0411	-4.9957
45	H	-5.0230	-2.2038	-3.7106
46	H	-6.3965	-1.1129	-3.5949
47	Ir	0.1388	0.2989	-0.3325
48	C	-0.8461	2.1529	-0.3988
49	P	1.1135	-1.8610	-0.4878
50	C	1.3369	-2.6695	1.1406
51	C	0.1635	-3.0806	-1.4621
52	C	2.7847	-1.8628	-1.2345
53	C	0.2151	-3.1902	1.7990
54	C	2.5754	-2.6982	1.7883
55	C	0.2566	-4.4490	-1.1809
56	C	-0.6166	-2.6642	-2.5451
57	C	3.1790	-2.8180	-2.1744
58	C	3.7032	-0.8817	-0.8393
59	C	0.3306	-3.7082	3.0827

60	H	-0.7522	-3.1958	1.3043
61	C	2.6860	-3.2196	3.0740
62	H	3.4640	-2.3308	1.2841
63	C	-0.4302	-5.3750	-1.9571
64	H	0.8675	-4.7930	-0.3520
65	C	-1.3011	-3.5930	-3.3196
66	H	-0.6975	-1.6073	-2.7787
67	C	4.4644	-2.7899	-2.7064
68	H	2.4812	-3.5826	-2.4999
69	C	4.9893	-0.8641	-1.3624
70	H	3.4100	-0.1286	-0.1104
71	C	1.5641	-3.7183	3.7263
72	H	-0.5489	-4.1056	3.5787
73	H	3.6564	-3.2484	3.5589
74	C	-1.2127	-4.9492	-3.0253
75	H	-0.3523	-6.4318	-1.7257
76	H	-1.9064	-3.2530	-4.1540
77	C	5.3709	-1.8181	-2.3012
78	H	4.7558	-3.5327	-3.4410
79	H	5.6934	-0.1038	-1.0411
80	H	1.6525	-4.1265	4.7274
81	H	-1.7505	-5.6739	-3.6273
82	H	6.3724	-1.8007	-2.7173
83	C	-1.4363	-0.5635	0.6828
84	C	-1.3729	-0.4922	2.0963
85	C	-2.5168	-1.2590	0.1353
86	C	-2.3644	-1.0360	2.9188
87	C	-3.5020	-1.8112	0.9510
88	H	-2.5937	-1.3739	-0.9417
89	C	-3.4410	-1.6895	2.3401
90	H	-2.2643	-0.9651	3.9976
91	H	-4.3313	-2.3454	0.4951
92	H	-4.2184	-2.1198	2.9616
93	C	1.9937	0.7091	2.5619
94	C	1.5246	0.5491	4.0121
95	H	2.4103	1.6977	2.3530
96	H	2.1169	-0.1500	4.6029
97	N	0.7766	0.4767	1.7955
98	H	1.2836	0.8973	-1.5540
99	H	1.6378	1.1491	-0.8148
100	H	-0.5926	0.0241	-1.7090
101	C	-0.1298	0.0324	2.6068
102	O	0.1816	-0.0015	3.9036
103	H	1.4469	1.5006	4.5453
104	H	2.7469	-0.0407	2.2857

Energy Decomposition Analysis

**[IrH2(IMes)(PPh3)]**		**(optimised geometry)**		
1	C	-0.6610	-2.8670	0.1420
2	C	0.2512	-3.0092	-0.9245
3	C	-0.1635	-3.3593	-2.2162
4	C	-1.5216	-3.5952	-2.4156
5	C	-2.4604	-3.4792	-1.3892
6	C	-2.0106	-3.1093	-0.1240
7	H	-2.7264	-3.0317	0.6907
8	C	-3.9083	-3.7664	-1.6315
9	H	-4.5518	-3.1174	-1.0341
10	H	-4.1749	-3.6365	-2.6818
11	H	-4.1494	-4.7998	-1.3618
12	C	-0.2132	-2.5429	1.5445
13	H	-0.1365	-1.4571	1.7889
14	H	-0.9400	-2.9133	2.2706
15	H	0.7642	-2.9705	1.7773
16	C	2.7726	-3.4535	-0.8274

17	C	3.8080	-2.6429	-0.4955
18	H	2.7501	-4.4855	-1.1342
19	H	4.8688	-2.8274	-0.4618
20	C	0.8089	-3.4424	-3.3518
21	H	1.5103	-2.6028	-3.3449
22	H	1.4089	-4.3567	-3.3141
23	H	0.2876	-3.4421	-4.3090
24	H	-1.8599	-3.8625	-3.4136
25	N	1.6279	-2.6898	-0.6791
26	N	3.2733	-1.4085	-0.1533
27	C	5.2982	1.6811	-0.3241
28	C	4.8781	1.1551	1.9776
29	H	5.7365	2.3304	-1.0775
30	C	4.5840	0.5597	-0.7400
31	C	4.3998	0.2418	-2.1918
32	H	3.3414	0.1204	-2.4445
33	H	4.8071	1.0325	-2.8217
34	H	4.8989	-0.6913	-2.4720
35	C	4.0302	-0.2538	0.2518
36	C	4.1533	0.0219	1.6159
37	C	3.5087	-0.8543	2.6452
38	H	3.7357	-1.9127	2.4848
39	H	3.8381	-0.5893	3.6499
40	H	2.4173	-0.7563	2.6174
41	H	4.9901	1.3887	3.0332
42	C	5.4615	1.9928	1.0262
43	C	6.2662	3.1834	1.4457
44	H	6.2504	3.9697	0.6891
45	H	5.9056	3.6051	2.3856
46	H	7.3148	2.9093	1.6004
47	Ir	0.3340	-0.1816	0.0340
48	H	1.1746	0.8504	0.8282
49	H	0.7406	0.7443	-1.1444
50	C	1.9257	-1.4278	-0.2635
51	P	-1.5955	1.1312	0.2538
52	C	-2.3275	1.1033	1.9258
53	C	-1.4581	2.9009	-0.1579
54	C	-2.9140	0.4908	-0.8387
55	C	-2.8320	-0.1090	2.4129
56	C	-2.3210	2.2229	2.7598
57	C	-2.5986	3.6508	-0.4645
58	C	-0.2125	3.5318	-0.1150
59	C	-2.5331	-0.0198	-2.0851
60	C	-4.2699	0.5114	-0.4959
61	C	-3.3318	-0.1965	3.7044
62	H	-2.8511	-0.9812	1.7626
63	C	-2.8174	2.1297	4.0569
64	H	-1.9340	3.1701	2.3969
65	C	-2.4913	5.0111	-0.7214
66	H	-3.5718	3.1701	-0.5075
67	C	-0.1107	4.8955	-0.3683
68	H	0.6770	2.9529	0.1135
69	C	-3.4907	-0.4859	-2.9776
70	H	-1.4790	-0.0457	-2.3568
71	C	-5.2250	0.0433	-1.3916
72	H	-4.5798	0.8968	0.4712
73	C	-3.3224	0.9250	4.5304
74	H	-3.7339	-1.1365	4.0680
75	H	-2.8123	3.0056	4.6966
76	C	-1.2479	5.6350	-0.6715
77	H	-3.3791	5.5854	-0.9629
78	H	0.8598	5.3787	-0.3331
79	C	-4.8378	-0.4510	-2.6334
80	H	-3.1837	-0.8769	-3.9426
81	H	-6.2752	0.0705	-1.1206
82	H	-3.7120	0.8582	5.5403

83	H	-1.1665	6.6975	-0.8740
84	H	-5.5866	-0.8129	-3.3304

**Acetophenone**		**fragment from [Ir(H)$_2$(PhAc)(IMes)(PPh$_3$)]**		
1	C	-1.6980	0.7722	-2.3055
2	C	-2.7856	0.4675	-1.3630
3	C	-4.0590	0.1127	-1.8289
4	C	-2.5653	0.4911	0.0223
5	C	-5.0641	-0.2424	-0.9417
6	H	-4.2547	0.0843	-2.8953
7	C	-3.5703	0.1432	0.9115
8	H	-1.6511	0.9322	0.4415
9	C	-4.8196	-0.2356	0.4285
10	H	-6.0389	-0.5321	-1.3184
11	H	-3.3888	0.1891	1.9802
12	H	-5.6065	-0.5109	1.1226
13	O	-0.5101	0.7585	-1.9542
14	C	-2.0200	1.0866	-3.7314
15	H	-2.7924	1.8564	-3.8059
16	H	-1.1200	1.4092	-4.2513
17	H	-2.4066	0.1925	-4.2311

**[IrH$_2$(IMes)(PPh$_3$)]**		**fragment from [Ir(H)$_2$(PhAc)(IMes)(PPh$_3$)]**		
1	C	-1.3649	3.8649	-1.3431
2	C	-0.9489	3.5660	-0.0405
3	C	-1.8424	3.4676	1.0298
4	C	-3.1981	3.6524	0.7604
5	C	-3.6639	3.9237	-0.5249
6	C	-2.7321	4.0286	-1.5601
7	H	-3.0780	4.2570	-2.5660
8	C	-5.1252	4.1112	-0.7914
9	H	-5.4190	3.6706	-1.7471
10	H	-5.7392	3.6635	-0.0081
11	H	-5.3840	5.1737	-0.8392
12	C	-0.3711	4.0048	-2.4547
13	H	-0.8705	4.1096	-3.4190
14	H	0.2594	4.8890	-2.3179
15	H	0.3017	3.1432	-2.5048
16	C	1.3325	4.4273	0.4290
17	C	2.5491	3.8729	0.6275
18	H	1.0048	5.4536	0.4239
19	H	3.5114	4.3148	0.8258
20	C	-1.3597	3.1148	2.4033
21	H	-0.5624	3.7831	2.7421
22	H	-2.1705	3.1608	3.1311
23	H	-0.9365	2.1016	2.4289
24	H	-3.9104	3.5632	1.5770
25	N	0.4519	3.3798	0.2097
26	N	2.3865	2.5031	0.5276
27	C	4.8671	0.2054	2.0301
28	C	5.1653	0.2259	-0.3504
29	H	5.1547	-0.1596	3.0130
30	C	3.8165	1.1170	1.9343
31	C	3.0765	1.5941	3.1451
32	H	1.9955	1.4668	3.0268
33	H	3.3874	1.0498	4.0371
34	H	3.2494	2.6591	3.3316
35	C	3.4701	1.5672	0.6586
36	C	4.1227	1.1372	-0.5008
37	C	3.6870	1.6199	-1.8498
38	H	4.3310	1.2259	-2.6362
39	H	2.6585	1.3084	-2.0669
40	H	3.7044	2.7123	-1.9170
41	H	5.6853	-0.1256	-1.2379
42	C	5.5573	-0.2444	0.9039
43	C	6.7139	-1.1867	1.0385

44	H	6.6260	-1.8143	1.9278
45	H	6.8108	-1.8362	0.1658
46	H	7.6559	-0.6364	1.1302
47	Ir	0.3555	0.2467	0.0723
48	H	1.8186	-0.1229	-0.2546
49	H	0.9042	-0.0282	1.5222
50	C	1.0874	2.1711	0.2677
51	P	-0.1389	-2.0472	0.0281
52	C	-1.3204	-2.5528	-1.2723
53	C	1.3347	-3.0939	-0.2586
54	C	-0.8442	-2.7356	1.5680
55	C	-0.9508	-2.3557	-2.6096
56	C	-2.6048	-3.0215	-0.9854
57	C	1.2937	-4.2494	-1.0442
58	C	2.5258	-2.7523	0.3924
59	C	-0.9955	-4.1181	1.7230
60	C	-1.2454	-1.8870	2.6011
61	C	-1.8415	-2.6432	-3.6360
62	H	0.0431	-1.9814	-2.8443
63	C	-3.4964	-3.3016	-2.0165
64	H	-2.9133	-3.1673	0.0453
65	C	2.4274	-5.0435	-1.1821
66	H	0.3774	-4.5311	-1.5541
67	C	3.6518	-3.5539	0.2582
68	H	2.5717	-1.8572	1.0071
69	C	-1.5510	-4.6367	2.8848
70	H	-0.6728	-4.7909	0.9327
71	C	-1.8032	-2.4080	3.7644
72	H	-1.0992	-0.8152	2.4957
73	C	-3.1186	-3.1159	-3.3412
74	H	-1.5357	-2.5064	-4.6687
75	H	-4.4896	-3.6697	-1.7803
76	C	3.6063	-4.6986	-0.5314
77	H	2.3862	-5.9354	-1.7983
78	H	4.5669	-3.2811	0.7730
79	C	-1.9587	-3.7818	3.9057
80	H	-1.6615	-5.7098	2.9974
81	H	-2.1063	-1.7411	4.5650
82	H	-3.8119	-3.3463	-4.1434
83	H	4.4880	-5.3215	-0.6390
84	H	-2.3883	-4.1893	4.8145

**Acetophenone**		**fragment from [Ir(H)$_2$(PhAc)$_2$(IMes)(PPh$_3$)]**		
1	C	2.8189	-0.1830	-0.8150
2	C	-1.1238	0.3026	2.6069
3	O	-0.3927	-0.2660	1.7852
4	O	1.8164	-0.3761	-0.1168
5	C	-2.5072	0.7377	2.2418
6	H	-2.7930	0.2945	1.2881
7	H	-3.2336	0.4621	3.0102
8	H	-2.5402	1.8285	2.1439
9	C	-0.5983	0.5731	3.9563
10	C	-1.3162	1.3366	4.8848
11	C	0.6800	0.1073	4.2973
12	C	-0.7656	1.6324	6.1245
13	H	-2.3006	1.7170	4.6342
14	C	1.2213	0.3919	5.5389
15	H	1.2327	-0.4697	3.5632
16	C	0.5003	1.1591	6.4537
17	H	-1.3239	2.2308	6.8357
18	H	2.2071	0.0225	5.8009
19	H	0.9266	1.3861	7.4252
20	C	2.7743	-0.2795	-2.3059
21	H	1.7888	-0.6186	-2.6240
22	H	2.9736	0.6975	-2.7569
23	H	3.5414	-0.9630	-2.6766

24	C	4.0834	0.1828	-0.1419
25	C	5.2288	0.5341	-0.8656
26	C	4.1316	0.1955	1.2596
27	C	6.3945	0.8969	-0.2027
28	H	5.2079	0.5357	-1.9506
29	C	5.2963	0.5527	1.9189
30	H	3.2406	-0.0944	1.8091
31	C	6.4302	0.9069	1.1879
32	H	7.2764	1.1728	-0.7704
33	H	5.3283	0.5569	3.0036
34	H	7.3417	1.1894	1.7041

$[IrH_2(IMes)(PPh_3)]$ fragment from $[Ir(H)_2(PhAc)_2(IMes)(PPh_3)]$

1	C	-3.9097	-2.2188	0.6508
2	C	-3.2740	-2.4643	-0.5702
3	C	-3.8205	-2.0786	-1.7953
4	C	-5.0469	-1.4145	-1.7722
5	C	-5.7151	-1.1392	-0.5797
6	C	-5.1304	-1.5493	0.6209
7	H	-5.6384	-1.3422	1.5603
8	C	-7.0363	-0.4328	-0.5796
9	H	-7.2890	-0.0469	-1.5685
10	H	-7.8439	-1.1054	-0.2756
11	H	-7.0454	0.4044	0.1240
12	C	-3.2731	-2.6418	1.9389
13	H	-2.2479	-2.2632	2.0191
14	H	-3.8423	-2.2856	2.7987
15	H	-3.2046	-3.7316	2.0191
16	C	-1.8581	-4.4948	-0.5321
17	C	-0.5359	-4.7539	-0.4343
18	H	-2.7125	-5.1483	-0.5907
19	H	0.0121	-5.6803	-0.3909
20	C	-3.0832	-2.3236	-3.0730
21	H	-2.1469	-1.7549	-3.0891
22	H	-2.8141	-3.3772	-3.1945
23	H	-3.6780	-2.0258	-3.9368
24	H	-5.4874	-1.0984	-2.7147
25	N	-1.9948	-3.1179	-0.5488
26	N	0.1061	-3.5271	-0.3894
27	C	3.6918	-3.3417	-1.2720
28	C	3.4661	-3.1567	1.1066
29	H	4.3192	-3.3719	-2.1603
30	C	2.3077	-3.4511	-1.4225
31	C	1.6739	-3.6013	-2.7714
32	H	1.2759	-4.6105	-2.9198
33	H	0.8325	-2.9121	-2.8996
34	H	2.3941	-3.4154	-3.5696
35	C	1.5298	-3.4023	-0.2668
36	C	2.0830	-3.2655	1.0116
37	C	1.2056	-3.2087	2.2214
38	H	0.5391	-4.0750	2.2830
39	H	1.7984	-3.1787	3.1371
40	H	0.5676	-2.3194	2.1951
41	H	3.9173	-3.0369	2.0891
42	C	4.2881	-3.1865	-0.0224
43	C	5.7711	-3.0370	0.1172
44	H	6.0232	-2.1410	0.6934
45	H	6.2094	-3.8878	0.6473
46	H	6.2646	-2.9620	-0.8533
47	Ir	-0.4743	-0.4446	-0.5097
48	H	-0.4255	-0.5443	-2.0803
49	H	-2.0155	-0.3923	-0.7011
50	C	-0.7798	-2.4891	-0.4587
51	P	-0.4278	1.8919	-0.7614
52	C	-0.0537	2.8318	0.7712
53	C	0.8526	2.4698	-1.9442

54	C	-1.9402	2.6881	-1.4189
55	C	1.0427	2.4155	1.5380
56	C	-0.8144	3.9173	1.2161
57	C	2.0446	3.0811	-1.5508
58	C	0.6328	2.2207	-3.3053
59	C	-1.8668	3.9709	-1.9781
60	C	-3.1846	2.0579	-1.3382
61	C	1.3901	3.0887	2.7020
62	H	1.6089	1.5414	1.2273
63	C	-0.4779	4.5739	2.3968
64	H	-1.6754	4.2508	0.6452
65	C	3.0003	3.4344	-2.5015
66	H	2.2317	3.2966	-0.5038
67	C	1.5802	2.5861	-4.2507
68	H	-0.2909	1.7434	-3.6229
69	C	-3.0117	4.6045	-2.4431
70	H	-0.9067	4.4743	-2.0522
71	C	-4.3299	2.6951	-1.8048
72	H	-3.2602	1.0577	-0.9251
73	C	0.6270	4.1677	3.1371
74	H	2.2471	2.7557	3.2797
75	H	-1.0791	5.4124	2.7328
76	C	2.7710	3.1908	-3.8503
77	H	3.9213	3.9115	-2.1818
78	H	1.3917	2.3994	-5.3027
79	C	-4.2464	3.9675	-2.3570
80	H	-2.9393	5.5963	-2.8765
81	H	-5.2872	2.1874	-1.7424
82	H	0.8880	4.6851	4.0545
83	H	3.5109	3.4774	-4.5901
84	H	-5.1390	4.4619	-2.7256

2-Phenylpyridine **fragment from [Ir(H)$_2$(PhPy)(IMes)(PPh$_3$)]**

1	C	1.1577	-0.4243	-2.6705
2	C	-1.1583	-0.5551	-2.7824
3	C	1.2269	-0.5490	-4.0639
4	C	-1.1551	-0.6397	-4.1621
5	H	-2.0885	-0.5510	-2.2236
6	C	0.0713	-0.6509	-4.8164
7	H	2.1952	-0.5737	-4.5483
8	H	-2.0916	-0.7055	-4.7025
9	H	0.1258	-0.7401	-5.8959
10	C	2.3692	-0.2164	-1.8604
11	C	3.5296	0.3146	-2.4441
12	C	2.3994	-0.4772	-0.4838
13	C	4.6583	0.5833	-1.6855
14	H	3.5324	0.5701	-3.4979
15	C	3.5312	-0.2126	0.2789
16	H	1.6164	-1.0897	-0.0074
17	C	4.6625	0.3282	-0.3161
18	H	5.5355	1.0077	-2.1621
19	H	3.5300	-0.4484	1.3379
20	H	5.5429	0.5434	0.2796
21	N	-0.0427	-0.4590	-2.0389

[IrH$_2$(IMes)(PPh$_3$)] **fragment from [Ir(H)$_2$(PhPy)(IMes)(PPh$_3$)]**

1	C	1.1502	-3.7221	-1.7620
2	C	1.0443	-3.5581	-0.3803
3	C	2.1640	-3.5349	0.4633
4	C	3.4183	-3.6563	-0.1231
5	C	3.5764	-3.7793	-1.5062
6	C	2.4350	-3.8147	-2.3029
7	H	2.5408	-3.9228	-3.3800
8	C	4.9455	-3.8466	-2.1078
9	H	5.4883	-2.9095	-1.9481
10	H	5.5449	-4.6388	-1.6516

11	H	4.9084	-4.0308	-3.1822
12	C	-0.0609	-3.8341	-2.6350
13	H	-0.3819	-4.8779	-2.7229
14	H	-0.9111	-3.2742	-2.2400
15	H	0.1459	-3.4780	-3.6462
16	C	-1.0020	-4.4971	0.6570
17	C	-2.1060	-3.9866	1.2450
18	H	-0.6726	-5.5119	0.5077
19	H	-2.9512	-4.4627	1.7135
20	C	2.0061	-3.3081	1.9355
21	H	2.9620	-3.3942	2.4531
22	H	1.5997	-2.3079	2.1387
23	H	1.3087	-4.0173	2.3918
24	H	4.2998	-3.6252	0.5130
25	N	-0.2524	-3.4176	0.2192
26	N	-2.0059	-2.6095	1.1596
27	C	-3.8529	-0.4627	3.5427
28	C	-5.0227	-0.4809	1.4506
29	H	-3.7705	-0.1280	4.5739
30	C	-2.8707	-1.3123	3.0338
31	C	-1.7254	-1.7772	3.8781
32	H	-1.7502	-2.8618	4.0276
33	H	-0.7646	-1.5456	3.4085
34	H	-1.7455	-1.3079	4.8620
35	C	-3.0006	-1.7257	1.7055
36	C	-4.0714	-1.3336	0.8966
37	C	-4.2043	-1.8358	-0.5078
38	H	-4.8492	-1.1860	-1.1013
39	H	-3.2340	-1.9135	-1.0056
40	H	-4.6457	-2.8382	-0.5311
41	H	-5.8593	-0.1589	0.8354
42	C	-4.9348	-0.0388	2.7711
43	C	-5.9970	0.8461	3.3478
44	H	-6.2772	1.6433	2.6544
45	H	-6.9083	0.2777	3.5583
46	H	-5.6795	1.3042	4.2862
47	Ir	-0.2626	-0.2591	0.1966
48	H	-1.7805	0.0637	0.2731
49	H	-0.3665	-0.1138	1.7773
50	C	-0.8582	-2.2269	0.5180
51	P	0.0779	2.0648	0.1700
52	C	0.4335	2.7289	-1.4934
53	C	-1.3830	3.0173	0.7321
54	C	1.4045	2.7164	1.2483
55	C	-0.6112	2.7777	-2.4265
56	C	1.7307	3.0255	-1.9165
57	C	-1.6250	4.3177	0.2758
58	C	-2.2155	2.4746	1.7158
59	C	1.8455	4.0397	1.1337
60	C	1.9375	1.9079	2.2541
61	C	-0.3640	3.1311	-3.7455
62	H	-1.6252	2.5420	-2.1111
63	C	1.9755	3.3823	-3.2395
64	H	2.5569	2.9715	-1.2138
65	C	-2.6855	5.0554	0.7876
66	H	-0.9892	4.7550	-0.4885
67	C	-3.2672	3.2217	2.2331
68	H	-2.0401	1.4660	2.0792
69	C	2.8204	4.5314	1.9912
70	H	1.4273	4.6885	0.3692
71	C	2.9100	2.4040	3.1159
72	H	1.5788	0.8878	2.3608
73	C	0.9321	3.4363	-4.1555
74	H	-1.1839	3.1723	-4.4553
75	H	2.9880	3.6199	-3.5510
76	C	-3.5066	4.5104	1.7694

77	H	-2.8675	6.0601	0.4211
78	H	-3.8982	2.7919	3.0041
79	C	3.3572	3.7128	2.9814
80	H	3.1581	5.5574	1.8912
81	H	3.3157	1.7679	3.8961
82	H	1.1242	3.7204	-5.1848
83	H	-4.3296	5.0911	2.1723
84	H	4.1164	4.1003	3.6524

2-Phenylpyridine fragment from [Ir(H)$_2$(PhPy)$_2$(IMes)(PPh$_3$)]

1	C	-0.5426	-0.1005	3.1977
2	C	-2.3968	1.0663	2.5064
3	C	-0.7770	0.1677	4.5369
4	C	-2.6870	1.3986	3.8356
5	C	-1.8598	0.9719	4.8609
6	H	-0.1276	-0.2599	5.2926
7	H	-3.5978	1.9447	4.0531
8	H	-2.0867	1.2211	5.8923
9	N	-1.2741	0.3782	2.1799
10	C	3.2732	-0.7942	0.8685
11	C	2.1867	0.5626	2.3751
12	C	4.1728	-1.1866	1.8676
13	C	3.0518	0.2353	3.4075
14	C	4.0549	-0.6877	3.1540
15	H	4.9911	-1.8463	1.6028
16	H	2.9311	0.7065	4.3768
17	H	4.7557	-0.9840	3.9275
18	N	2.2374	0.0415	1.1386
19	H	0.2849	-0.7402	2.9181
20	H	1.3921	1.2791	2.5471
21	C	3.5585	-1.2130	-0.5151
22	C	3.5294	-0.2730	-1.5491
23	C	4.0473	-2.4980	-0.7836
24	C	4.0155	-0.5999	-2.8094
25	H	3.1638	0.7282	-1.3377
26	C	4.5095	-2.8288	-2.0482
27	H	4.0568	-3.2453	0.0054
28	C	4.5109	-1.8740	-3.0606
29	H	4.0262	0.1498	-3.5938
30	H	4.8719	-3.8322	-2.2441
31	H	4.8961	-2.1243	-4.0436
32	C	-3.4011	1.3655	1.4700
33	C	-3.6944	0.4065	0.4957
34	C	-4.1818	2.5278	1.5277
35	C	-4.7454	0.5988	-0.3916
36	H	-3.1135	-0.5085	0.4646
37	C	-5.2302	2.7203	0.6390
38	H	-3.9616	3.2890	2.2706
39	C	-5.5201	1.7517	-0.3179
40	H	-4.9689	-0.1677	-1.1269
41	H	-5.8243	3.6263	0.6945
42	H	-6.3494	1.8964	-1.0030

[IrH$_2$(IMes)(PPh$_3$)] fragment from [Ir(H)$_2$(PhPy)$_2$(IMes)(PPh$_3$)]

1	C	0.5119	3.7314	2.0748
2	C	1.4995	3.4439	1.1190
3	C	2.8554	3.3882	1.4432
4	C	3.2069	3.5815	2.7823
5	C	2.2569	3.8010	3.7740
6	C	0.9117	3.8779	3.3986
7	H	0.1568	4.0833	4.1542
8	C	2.6556	3.9690	5.2079
9	H	2.5061	4.9994	5.5441
10	H	2.0535	3.3360	5.8664
11	H	3.7063	3.7219	5.3681
12	C	-0.9155	3.9180	1.6674

13	H	-1.5532	4.0772	2.5383
14	H	-1.0260	4.7866	1.0083
15	H	-1.2987	3.0580	1.1125
16	C	1.1674	4.2880	-1.1735
17	C	0.5311	3.8551	-2.2819
18	H	1.6716	5.2134	-0.9494
19	H	0.3607	4.3241	-3.2367
20	C	3.9173	3.0655	0.4376
21	H	4.2756	2.0383	0.5730
22	H	3.5702	3.1556	-0.5922
23	H	4.7841	3.7187	0.5603
24	H	4.2581	3.5263	3.0560
25	N	1.0731	3.2674	-0.2384
26	N	0.0579	2.5854	-2.0045
27	C	-1.1307	0.3662	-4.7386
28	C	-3.0159	1.4042	-3.6865
29	H	-0.7201	-0.2991	-5.4951
30	C	-0.2514	1.0462	-3.8983
31	C	1.2284	0.9240	-4.0719
32	H	1.7527	0.9785	-3.1172
33	H	1.4992	-0.0166	-4.5565
34	H	1.6178	1.7342	-4.6995
35	C	-0.8006	1.8794	-2.9205
36	C	-2.1768	2.0997	-2.8159
37	C	-2.7162	3.0522	-1.7984
38	H	-2.1937	4.0135	-1.8256
39	H	-3.7776	3.2397	-1.9551
40	H	-2.5927	2.6536	-0.7854
41	H	-4.0900	1.5610	-3.6132
42	C	-2.5140	0.5271	-4.6478
43	C	-3.4255	-0.2018	-5.5871
44	H	-3.3195	0.1730	-6.6097
45	H	-3.1969	-1.2707	-5.6199
46	H	-4.4741	-0.0864	-5.3066
47	Ir	0.0657	0.2326	-0.0743
48	H	-1.3062	0.3819	-0.8031
49	H	0.8110	-0.0220	-1.4154
50	C	0.3840	2.1878	-0.7267
51	P	-0.3617	-2.0821	-0.0757
52	C	-0.2528	-2.8945	1.5543
53	C	-1.9815	-2.6176	-0.7736
54	C	0.6800	-3.1055	-1.1964
55	C	-1.3721	-3.0141	2.3850
56	C	1.0118	-3.1830	2.0833
57	C	-2.5181	-3.8730	-0.4650
58	C	-2.5930	-1.8503	-1.7689
59	C	1.0262	-4.4270	-0.9020
60	C	0.9367	-2.6080	-2.4781
61	C	-1.2308	-3.4341	3.7031
62	H	-2.3587	-2.7634	2.0052
63	C	1.1521	-3.5924	3.4036
64	H	1.8922	-3.0855	1.4531
65	C	-3.6654	-4.3274	-1.1033
66	H	-2.0345	-4.5062	0.2727
67	C	-3.7340	-2.3142	-2.4148
68	H	-2.1790	-0.8838	-2.0427
69	C	1.6349	-5.2255	-1.8651
70	H	0.8067	-4.8462	0.0749
71	C	1.5258	-3.4142	-3.4422
72	H	0.6509	-1.5897	-2.7271
73	C	0.0292	-3.7219	4.2168
74	H	-2.1094	-3.5283	4.3330
75	H	2.1392	-3.8175	3.7952
76	C	-4.2795	-3.5476	-2.0779
77	H	-4.0745	-5.2983	-0.8447
78	H	-4.1977	-1.7016	-3.1822

79	C	1.8802	-4.7246	-3.1381
80	H	1.9003	-6.2493	-1.6222
81	H	1.7144	-3.0146	-4.4342
82	H	0.1364	-4.0490	5.2456
83	H	-5.1730	-3.9057	-2.5781
84	H	2.3417	-5.3543	-3.8917

Acetophenone **fragment from $[Ir(H)_2(PhAc)(IMes)(PPh_3)]$ C-C Rotation TS2**

1	C	-1.8958	1.1426	-2.2850
2	C	-3.0089	0.4768	-1.5521
3	C	-3.8442	1.2250	-0.7214
4	C	-3.2476	-0.8879	-1.7331
5	C	-4.8872	0.6034	-0.0434
6	H	-3.6655	2.2875	-0.5974
7	C	-4.3106	-1.4955	-1.0762
8	H	-2.6206	-1.4617	-2.4064
9	C	-5.1255	-0.7548	-0.2257
10	H	-5.5209	1.1871	0.6175
11	H	-4.4982	-2.5534	-1.2282
12	H	-5.9471	-1.2370	0.2930
13	O	-0.7146	0.9569	-1.9962
14	C	-2.2732	2.0560	-3.4016
15	H	-2.9058	2.8599	-3.0080
16	H	-1.3965	2.4775	-3.8904
17	H	-2.8916	1.5216	-4.1288

$[IrH_2(IMes)(PPh_3)]$ **fragment from $[Ir(H)_2(PhAc)(IMes)(PPh_3)]$ C-C Rotation TS2**

1	C	-1.3789	4.3586	-0.5635
2	C	-0.7801	3.6262	0.4711
3	C	-1.4786	3.2493	1.6235
4	C	-2.8131	3.6460	1.7278
5	C	-3.4425	4.3973	0.7371
6	C	-2.7114	4.7336	-0.4055
7	H	-3.1894	5.3116	-1.1936
8	C	-4.8791	4.7995	0.8661
9	H	-5.5074	4.2555	0.1522
10	H	-5.2675	4.6001	1.8657
11	H	-5.0191	5.8626	0.6563
12	C	-0.6022	4.7393	-1.7874
13	H	-1.2662	5.0689	-2.5878
14	H	0.0919	5.5618	-1.5882
15	H	0.0023	3.9049	-2.1572
16	C	1.6120	4.2953	0.5151
17	C	2.7943	3.6584	0.3562
18	H	1.3776	5.3255	0.7248
19	H	3.8082	4.0211	0.3867
20	C	-0.8176	2.4761	2.7239
21	H	0.1638	2.8886	2.9757
22	H	-1.4305	2.4794	3.6259
23	H	-0.6451	1.4288	2.4472
24	H	-3.3689	3.3736	2.6217
25	N	0.6208	3.3339	0.3707
26	N	2.5031	2.3273	0.1221
27	C	4.8859	-0.4378	0.7281
28	C	4.6382	-0.1253	-1.6366
29	H	5.3105	-0.9835	1.5670
30	C	4.0224	0.6250	0.9885
31	C	3.6692	1.0174	2.3893
32	H	2.5845	1.0747	2.5276
33	H	4.0646	0.2994	3.1086
34	H	4.0727	2.0014	2.6498
35	C	3.4773	1.2944	-0.1093
36	C	3.7672	0.9415	-1.4318
37	C	3.1123	1.6518	-2.5765
38	H	3.5138	1.3117	-3.5315
39	H	2.0296	1.4756	-2.5823

**2-Phenylpyridine**		**fragment from [Ir(H)$_2$(PhPy)(IMes)(PPh$_3$)] C-C Rotation TS2**		
1	C	1.7560	-0.3327	-2.4353
2	C	-0.5085	-0.3248	-2.9212
3	C	2.0611	-0.4288	-3.7918
4	C	-0.2749	-0.3967	-4.2835
5	H	-1.5174	-0.2760	-2.5245
6	C	1.0387	-0.4621	-4.7292
7	H	3.1023	-0.4604	-4.0930
8	H	-1.1103	-0.3999	-4.9734
9	H	1.2642	-0.5270	-5.7882
10	C	2.8437	-0.2311	-1.4250
11	C	3.2861	1.0203	-0.9880
12	C	3.5048	-1.3743	-0.9728
13	C	4.3512	1.1272	-0.1026
14	H	2.8106	1.9191	-1.3611
15	C	4.5608	-1.2679	-0.0739
16	H	3.2051	-2.3425	-1.3511
17	C	4.9850	-0.0186	0.3650
18	H	4.6837	2.1093	0.2157
19	H	5.0615	-2.1681	0.2690
20	H	5.8182	0.0632	1.0549
21	N	0.4750	-0.3029	-2.0050

**[IrH$_2$(IMes)(PPh$_3$)]**		**fragment from [Ir(H)$_2$(PhPy)(IMes)(PPh$_3$)] C-C Rotation TS2**		
1	C	1.2943	-4.1368	-1.6378
2	C	1.1081	-3.7361	-0.3116
3	C	2.1036	-3.8729	0.6637
4	C	3.3117	-4.4444	0.2746
5	C	3.5472	-4.8555	-1.0392
6	C	2.5319	-4.6833	-1.9807
7	H	2.7000	-4.9949	-3.0086
8	C	4.8773	-5.4175	-1.4334
9	H	5.5976	-4.6138	-1.6236
10	H	5.3009	-6.0425	-0.6448
11	H	4.8132	-6.0144	-2.3443
12	C	0.1949	-4.0144	-2.6477
13	H	-0.5141	-4.8459	-2.5714
14	H	-0.3822	-3.0960	-2.5116
15	H	0.5920	-4.0223	-3.6639
16	C	-1.1402	-4.3520	0.3829
17	C	-2.2429	-3.7351	0.8521
18	H	-0.9114	-5.3923	0.2223
19	H	-3.1947	-4.1190	1.1792
20	C	1.8717	-3.4147	2.0695
21	H	2.7099	-3.6810	2.7139
22	H	1.7450	-2.3266	2.1151
23	H	0.9631	-3.8508	2.4969
24	H	4.0926	-4.5731	1.0204
25	N	-0.2109	-3.3579	0.1120
26	N	-1.9667	-2.3812	0.8641
27	C	-3.9842	-0.3673	3.2157
28	C	-4.8015	0.0103	0.9966
29	H	-4.0438	-0.1857	4.2863
30	C	-2.9890	-1.2156	2.7355
31	C	-1.9981	-1.8588	3.6549
32	H	-2.0709	-2.9511	3.6342
33	H	-0.9724	-1.6073	3.3649
34	H	-2.1497	-1.5363	4.6852
35	C	-2.9370	-1.4399	1.3554
36	C	-3.8397	-0.8482	0.4660
37	C	-3.8078	-1.1276	-1.0069
38	H	-2.9078	-1.6658	-1.3100
39	H	-4.6676	-1.7336	-1.3095
40	H	-3.8612	-0.1990	-1.5834
41	H	-5.5069	0.4867	0.3195

42	C	-4.8970	0.2572	2.3651
43	C	-5.9497	1.1784	2.8994
44	H	-5.8367	2.1866	2.4889
45	H	-6.9526	0.8375	2.6285
46	H	-5.9079	1.2536	3.9872
47	Ir	-0.0149	-0.1556	0.2064
48	H	-1.5188	0.1555	0.0624
49	H	-0.2612	-0.0613	1.7748
50	C	-0.6994	-2.1124	0.4025
51	P	0.0319	2.1979	0.1484
52	C	-0.4293	2.7447	-1.5390
53	C	-1.2842	2.9832	1.1631
54	C	1.4699	3.1827	0.7005
55	C	-1.7857	2.6843	-1.8870
56	C	0.5041	3.0216	-2.5424
57	C	-1.5684	4.3396	0.9554
58	C	-1.9529	2.2971	2.1754
59	C	1.8437	4.4060	0.1379
60	C	2.1509	2.7113	1.8275
61	C	-2.1946	2.9102	-3.1956
62	H	-2.5276	2.4660	-1.1220
63	C	0.0931	3.2472	-3.8522
64	H	1.5618	3.0783	-2.3079
65	C	-2.5034	4.9928	1.7439
66	H	-1.0569	4.8875	0.1685
67	C	-2.8805	2.9606	2.9749
68	H	-1.7505	1.2442	2.3400
69	C	2.8939	5.1348	0.6870
70	H	1.3120	4.8002	-0.7228
71	C	3.1833	3.4531	2.3865
72	H	1.8650	1.7574	2.2653
73	C	-1.2561	3.1926	-4.1831
74	H	-3.2510	2.8736	-3.4424
75	H	0.8326	3.4717	-4.6141
76	C	-3.1587	4.3038	2.7621
77	H	-2.7165	6.0421	1.5702
78	H	-3.3852	2.4184	3.7682
79	C	3.5615	4.6630	1.8125
80	H	3.1812	6.0813	0.2414
81	H	3.6982	3.0828	3.2668
82	H	-1.5751	3.3761	-5.2035
83	H	-3.8817	4.8172	3.3876
84	H	4.3718	5.2405	2.2444
