# Supplementary material for: Probing substrate binding and release events in iridium-catalysed hydrogen isotope exchange reactions
Source: Chem Sci. 2025 Jul 1;16(29):13532–42. doi: 10.1039/d5sc00759c (PMC12209880; doi:10.1039/d5sc00759c)
Supplement: SC-016-D5SC00759C-s002 [file SC-016-D5SC00759C-s002.pdf]

Supplementary Information (SI) for Chemical Science.
This journal is © The Royal Society of Chemistry 2025

**- Supplementary Information -**

# Probing Substrate Binding and Release Events in Iridium-Catalysed Hydrogen Isotope Exchange Reactions

Daria S. Timofeeva, William J. Kerr,* David M. Lindsay,* and David J. Nelson*

Department of Pure and Applied Chemistry, University of Strathclyde,
295 Cathedral Street, Glasgow, G1 1XL, UK

david.lindsay@strath.ac.uk, w.kerr@strath.ac.uk, david.nelson@strath.ac.uk

**Table of Contents**

| | | | |
|---|---|---|---|
| **1.** | General experimental details | | **S2** |
| **2.** | Synthesis and characterisation of compounds | | **S3** |
| **3.** | Kinetic data | | |
| | **3.1** | General kinetic protocol for reaction monitoring by sampling | **S12** |
| | **3.2** | Mass spectrometry analysis for isotopologue distribution over time | **S13** |
| | **3.3** | Kinetic data/isotopologue distribution for substituted phenylpyridines | **S22** |
| | **3.4** | Hammett plot for substituted phenylpyridines | **S37** |
| **4.** | Computational chemistry | | |
| | **4.1** | General computational details | **S38** |
| | **4.2** | Energies of optimised structures | **S39** |
| | **4.3** | Energy decomposition analysis | **S41** |
| | **4.4** | IRC electronic energy plots | **S42** |
| | **4.5** | Coordinates of optimised structures | **S42** |
| **5.** | Literature data on Lewis basicity | | **S43** |
| **6.** | References | | **S44** |

# 1. General experimental details

### General

For the synthetic procedures, standard Schlenk techniques using an inert gas atmosphere (argon or nitrogen) were used, unless otherwise stated. Materials obtained from commercial sources (acetophenone, ethyl benzoate, nitrobenzene, 2-phenylpyridine, 1-phenylpyrazole, 2-phenyloxozoline) were used without further purification. All glassware was flame dried and cooled under a stream of nitrogen or argon prior to use.

### Materials

(1,3-bis-(2,4,6-trimethylphenyl)imidazolium chloride,[1] 2-phenylpyrimidine,[2] 1-methyl-2-phenylimidazole[3] and 4-substituted 2-phenylpyridines[4] were synthesised according to literature procedures. Anhydrous Na[$BAr^F{}_{24}$] ($BAr^F{}_{24}$ = tetrakis[3,5-bis(trifluoromethyl)phenyl]borate)) was obtained following Bergman's synthesis,[5] followed by recrystallising the crude Na[$BArF_{24}$]·*x*(solvent) prior to drying.[6] [Ir(COD)(IMes)($PPh_3$)][$BAr^F{}_{24}$] was synthesised from [IrCl(COD)(IMes)][7] in a procedure adapted from that published before for the preparation of the corresponding complexes with $BF_4$ and OTf counterions.[8]

Flash column chromatography was carried out using silica gel (230-400 mesh). Thin layer chromatography (TLC) was performed using Merck silica plates coated with fluorescent indicator and visualised by UV light (254 nm).

### Analysis

$^1$H (400 MHz) and $^{13}$C{$^1$H} (101 MHz) NMR spectra were obtained on a Bruker AV3-400 instrument with a liquid nitrogen Prodigy cryoprobe. The chemical shifts (δ) are reported in ppm relative to the residual protonated solvent for $^1$H NMR or the solvent signal for $^{13}$C{$^1$H} NMR ($CDCl_3$: $\delta_H$ 7.26 ppm and $\delta_C$ 77.16 ppm).[S9] Multiplicities are expressed as s (singlet), d (doublet), t (triplet), q (quartet), m (multiplet), or br (broad signal). If no multiplicity is given for $^{13}$C{$^1$H} NMR data, the signal is a singlet. NMR assignments were made using additional 2D NMR experiments where necessary.

LC-MS analyses were carried out using Agilent 6130 with 1200 series LC and UV at 254 nm, with Agilent Poroshell 120 LC column (EC C18 2.7µm x 4.6mm x75mm). LC column conditions were as follows: mobile phase A: water + 5mM ammonium acetate; mobile phase B: acetonitrile + 5mM ammonium acetate; Flow rate: 1.000 mL/min.

| *Timetable:* | Time, min | 0 | 1.48 | 8.50 | 13.5 | 16.5 | 18.0 |
|---|---|---|---|---|---|---|---|
| | %A | 95 | 95 | 100 | 100 | 95 | 95 |
| | %B | 5 | 5 | 0 | 0 | 5 | 5 |

GC-MS analyses were carried out using an Agilent 7890A gas chromatograph fitted with a ZB-5 MS column (30 m x 0.25 mm I.D. x 0.25 µm) and an Agilent 5975C MSD running in EI mode.

## 2. Synthesis and characterisation

*General cross-coupling procedure for the synthesis of 4-X-2-Phenylpyridines*[4]

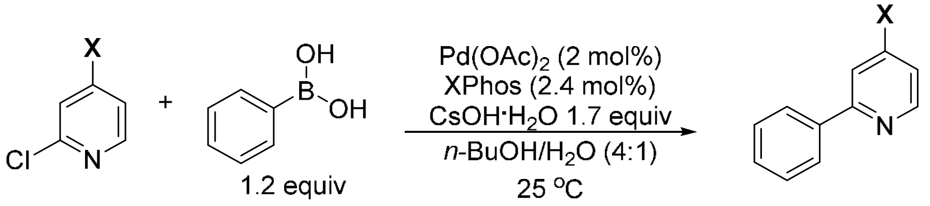


Under argon or nitro-gen, a 100 mL Schlenk flask containing a magnetic stirrer bar was sequentially charged with $Pd(OAc)_2$ (16 mg, 0.07 mmol), XPhos (39 mg, 0.08 mmol), 4-X-2-chloropyridine (4 mmol), phenylboronic acid (509 mg, 4.18 mmol), and degassed *n*-butanol (12 mL). The mixture was stirred at 25 °C for 15 min, and then a solution of $CsOH{\cdot}H_2O$ (887 mg, 5.92 mmol) in degassed $H_2O$ (3 mL) was added in one portion to initiate the Suzuki-Miyaura reaction. The reaction mixture was stirred vigorously at 25 °C until all the heteroaryl chloride was consumed (monitored by GC or TLC). At the end of the reaction, the organic phase was separated, and the aqueous phase was further extracted with ethyl acetate (20 mL x 3). The organic extracts were combined and concentrated on a rotary evaporator. The resulting residue was purified by silica gel flash chromatography to provide the desired coupling product.

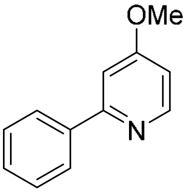


***4-methoxy-2-phenylpyridine*** was synthesized according general cross-coupling procedure using 2-chloro-4-methoxypyridine (500 mg, 3.48 mmol). After flash chromatography with ethyl acetate/hexane (1:8) as eluent, the title compound was isolated as a colourless oil (482 mg, 74% yield).

**$^1$H NMR** (400 MHz, $CDCl_3$) δ 8.52 (d, *J* = 5.7 Hz, 1H), 7.98 – 7.94 (m, 2H), 7.50 – 7.38 (m, 3H), 7.23 (d, *J* = 2.4 Hz, 1H), 6.77 (dd, *J* = 5.7, 2.4 Hz, 1H), 3.90 (s, 3H).

**$^{13}$C{$^1$H} NMR** (101 MHz, $CDCl_3$) δ 166.5, 159.4, 151.0, 139.6, 129.1, 128.8, 127.1, 108.2, 107.0, 55.3.

NMR data are consistent with the literature.[9]

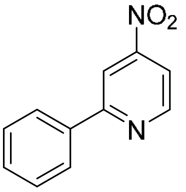


***4-nitro-2-phenylpyridine*** was synthesized according general cross-coupling procedure using 2-chloro-4-nitropyridine (552 mg, 3.48 mmol). After solvent evaporation, the title compound crystallised overnight, filtered and washed with pentane isolated as beige solid (340 mg, 49% yield).

**$^1$H NMR** (400 MHz, $CDCl_3$) δ 8.97 (d, *J* = 5.6 Hz, 1H), 8.45 (d, *J* = 1.6 Hz, 1H), 8.12 – 8.06 (m, 2H), 7.94 (dd, *J* = 5.3, 2.0 Hz, 1H), 7.59 – 7.48 (m, 3H).

**$^{13}$C{$^1$H} NMR** (101 MHz, $CDCl_3$) δ 160.6, 155.0, 151.9, 137.4, 130.6, 129.3, 127.3, 114.4, 112.9.

NMR data are consistent with the literature.[10]

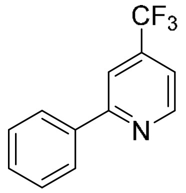

***4-trifluoromethyl-2-phenylpyridine*** was synthesized according general cross-coupling procedure using 2-chloro-4-trifluoromethyl pyridine (632 mg, 3.48 mmol). After flash chromatography with ethyl acetate/hexane (1:10) as eluent, the title compound was isolated as a colourless oil (605 mg, 78% yield).

**$^1$H NMR** (400 MHz, $CDCl_3$) δ 8.87 (d, $J$ = 5.1 Hz, 1H), 8.07 – 7.99 (m, 2H), 7.95 – 7.91 (m, 1H), 7.55 – 7.42 (m, 4H). **$^{13}$C{$^1$H} NMR** (101 MHz, $CDCl_3$) δ 159.0, 150.8, 139.3 (q, $^2J_{C\text{-}F}$ = 34.1 Hz), 138.2, 130.0, 129.1, 127.2, 123.1 (q, $^1J_{C\text{-}F}$ = 269.8 Hz), 117.7 (q, $^3J_{C\text{-}F}$ = 3.4 Hz), 116.2 (q, $^3J_{C\text{-}F}$ = 3.3 Hz). **$^{19}$F NMR** (376 MHz, $CDCl_3$) δ -64.83 (s).

NMR data are consistent with the literature.[11]

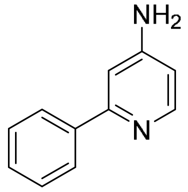

***4-amino-2-phenylpyridine*** was synthesized according general cross-coupling procedure using 2-chloro-4-aminopyridine (632 mg, 3.48 mmol). After flash chromatography starting with ethyl acetate/hexane (1:4) as eluent followed by ethyl acetate/hexane (1:2) and ethyl acetate/MeOH (10:1) mixture, the title compound was isolated as yellow solid (180 mg, 30% yield).

**$^1$H NMR** (400 MHz, $CDCl_3$) δ 8.26 (d, $J$ = 5.6 Hz, 1H), 7.86 (m, 2H), 7.45 – 7.37 (m, 3H), 6.91 (d, $J$ = 2.1 Hz, 1H), 6.46 (dd, $J$ = 5.6, 2.2 Hz, 1H), 4.33 (br. s, 2H). **$^{13}$C{$^1$H} NMR** (101 MHz, $CDCl_3$) δ 158.2, 153.8, 149.7, 139.5, 129.0, 128.7, , 127.1, 108.5, 106.7.

NMR data are consistent with the literature.[12]

*NMR spectra for substituted 2-phenylpyridine substrates*

## 4-methoxy-2-phenylpyidine: $^1$H

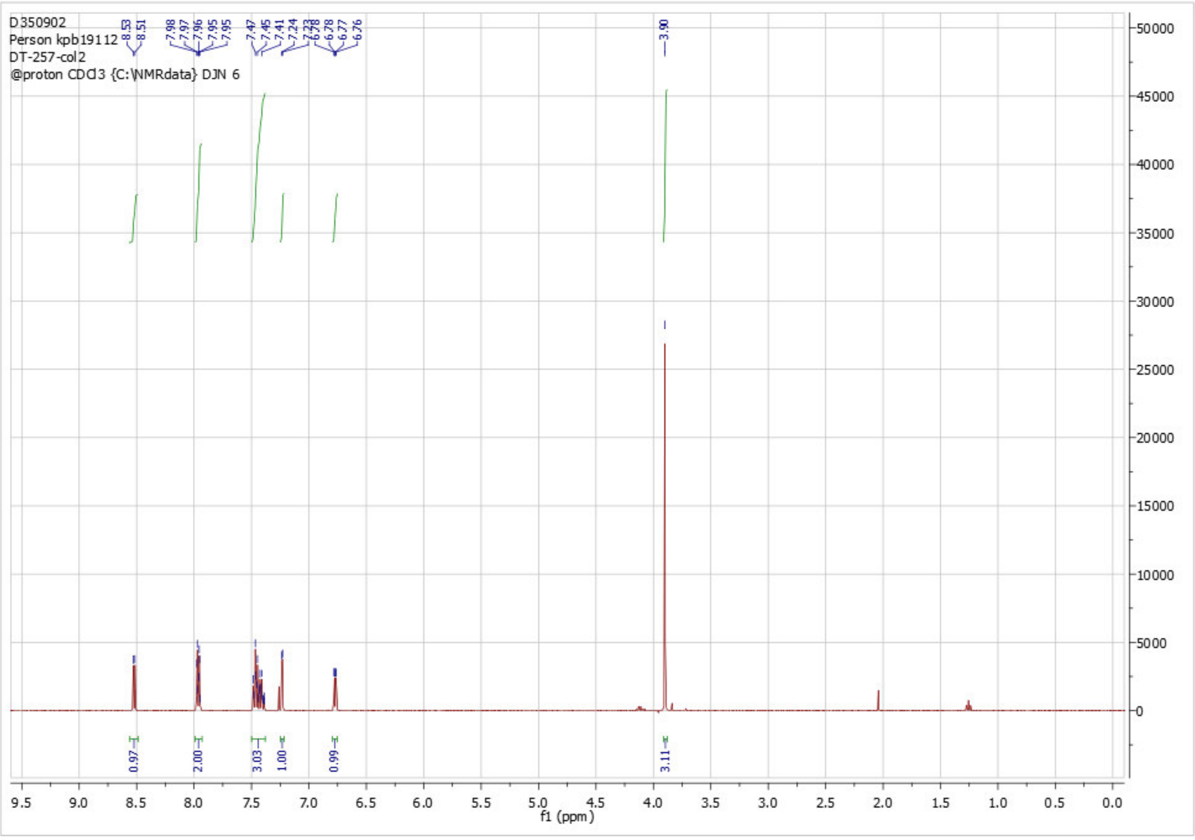


## 4-methoxy-2-phenylpyidine: $^{13}$C{$^1$H}

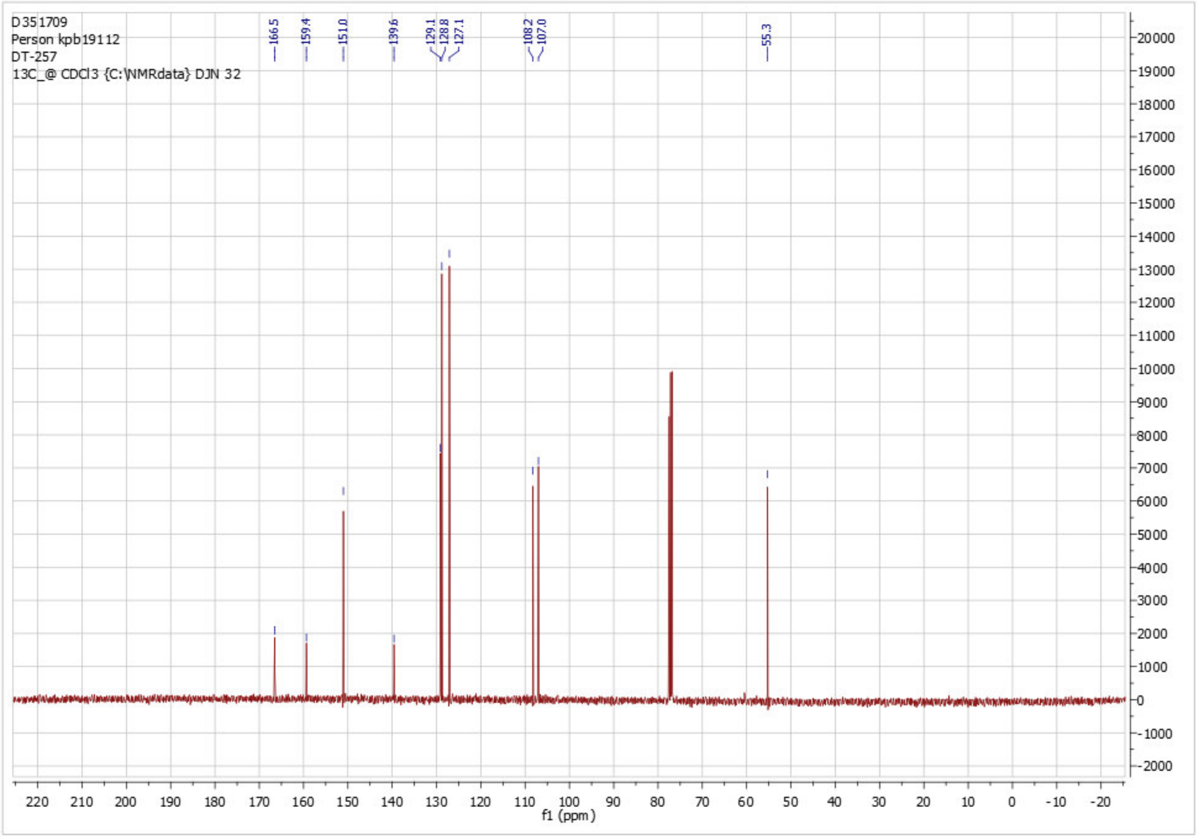

## 4-nitro-2-phenylpyidine: $^1H$

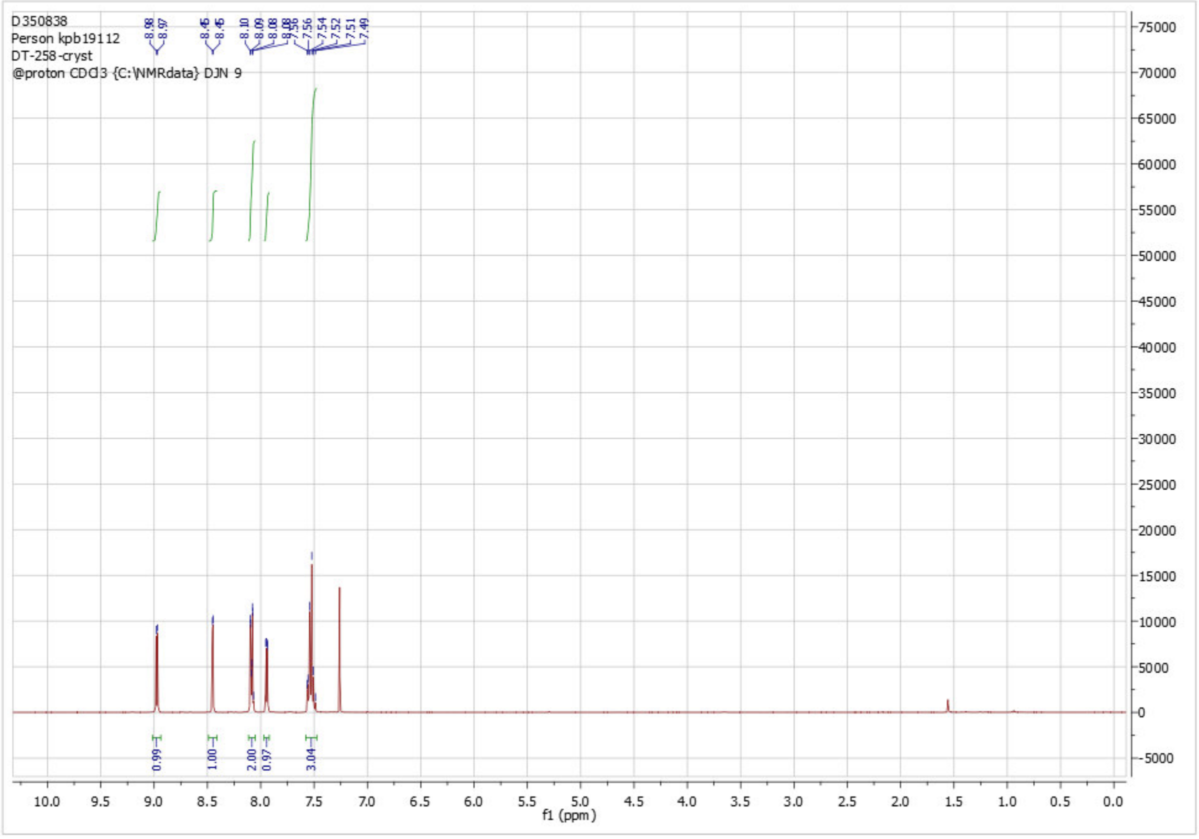


## 4-nitro-2-phenylpyidine: $^{13}C\{^1H\}$

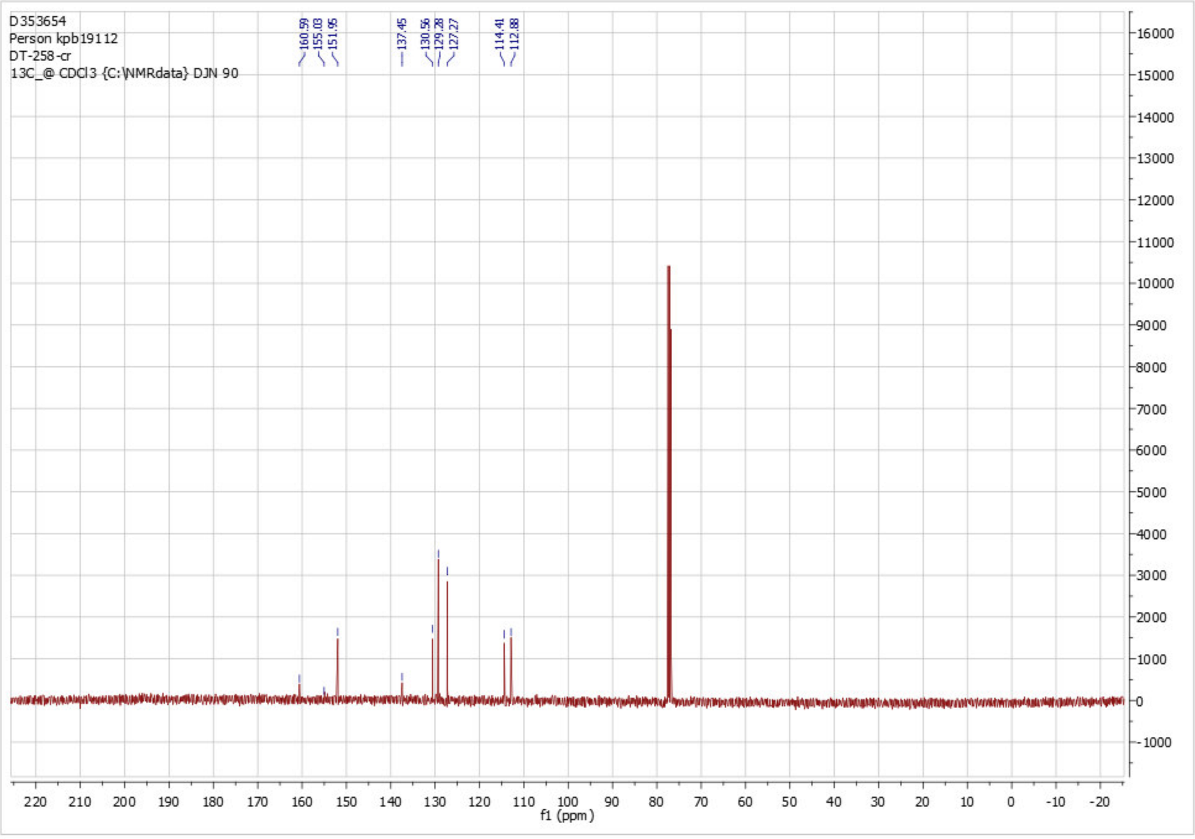

## 4-trifluoromethyl-2-phenylpyidine: $^{1}H$

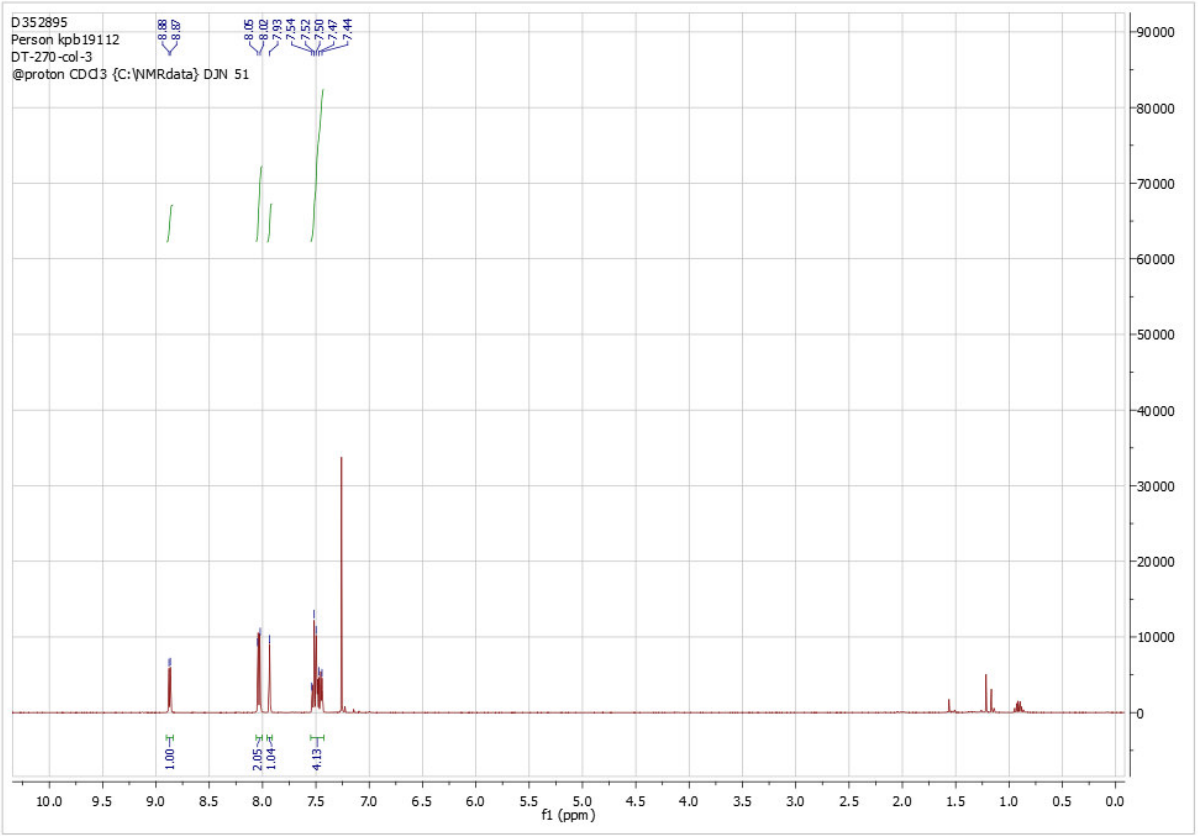


## 4-trifluoromethyl-2-phenylpyidine: $^{13}C\{^{1}H\}$

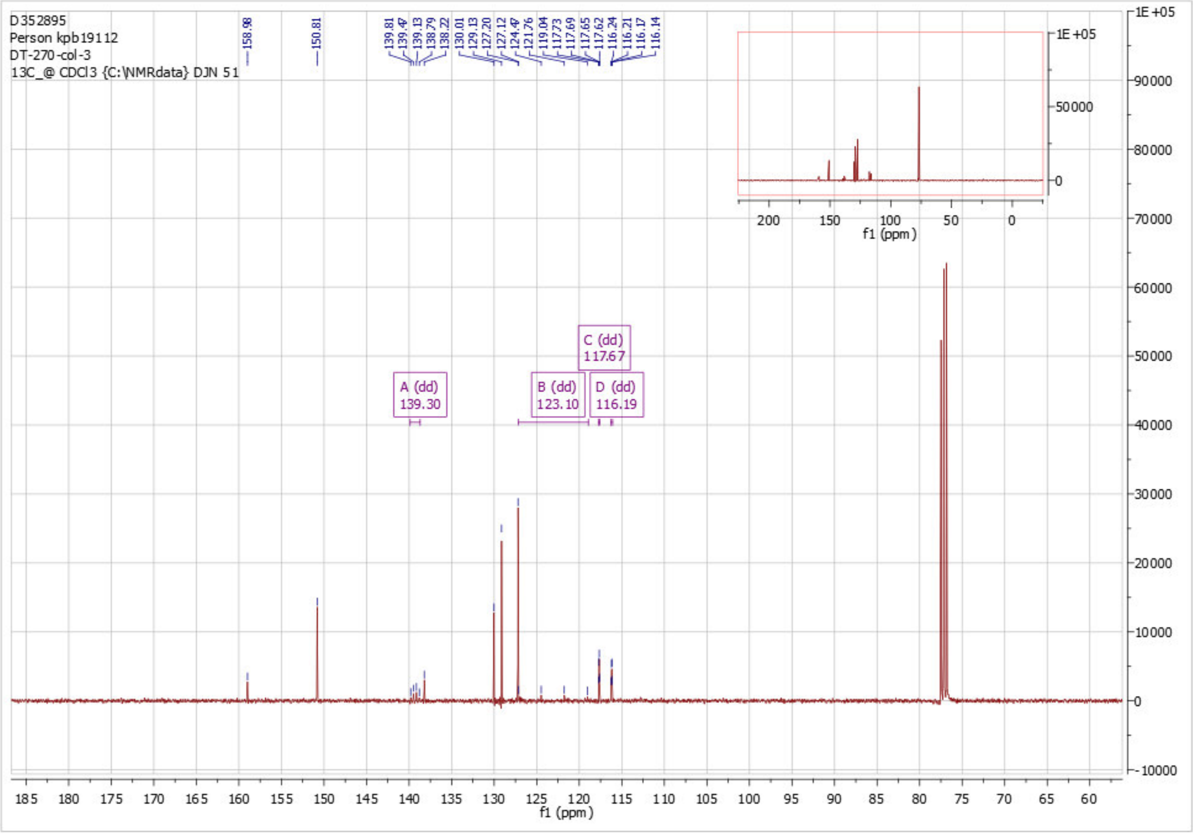

## 4-trifluoromethyl-2-phenylpyidine: $^{19}F$

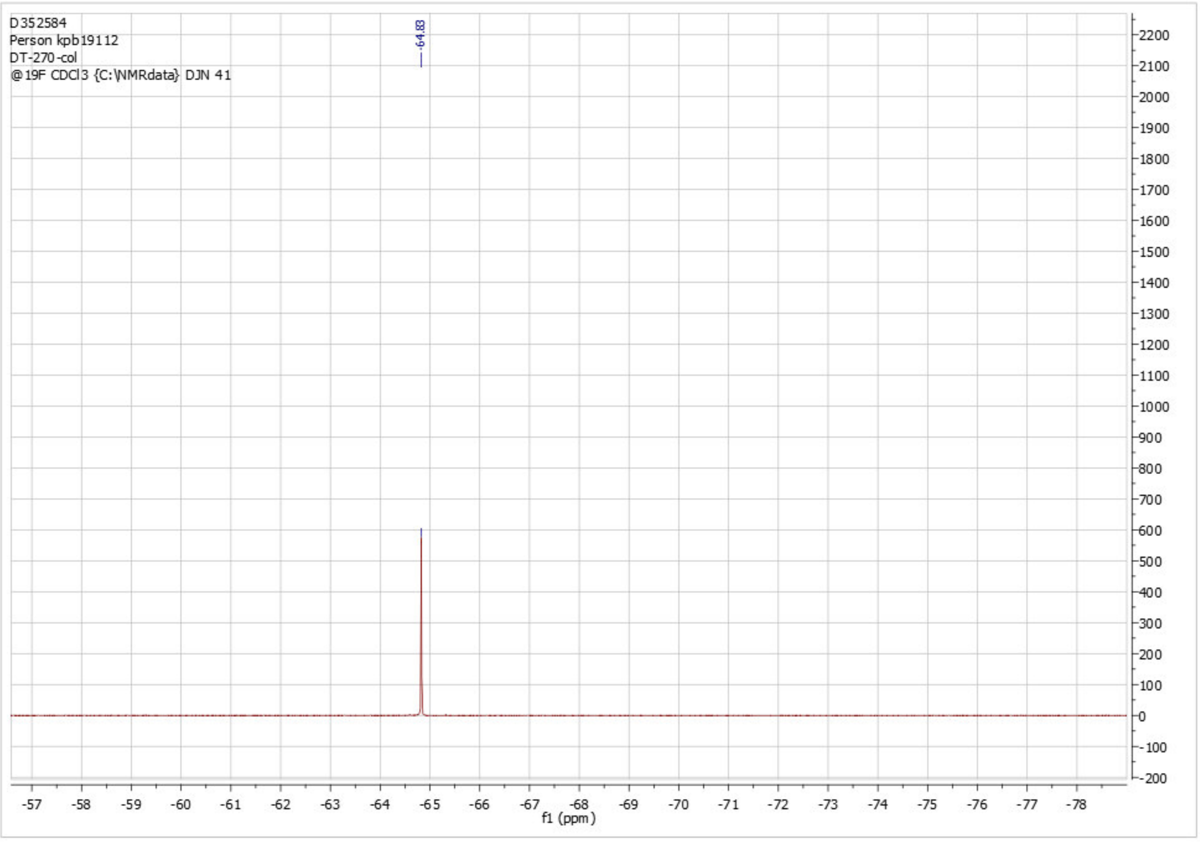


## 4-amino-2-phenylpyidine: $^{1}H$

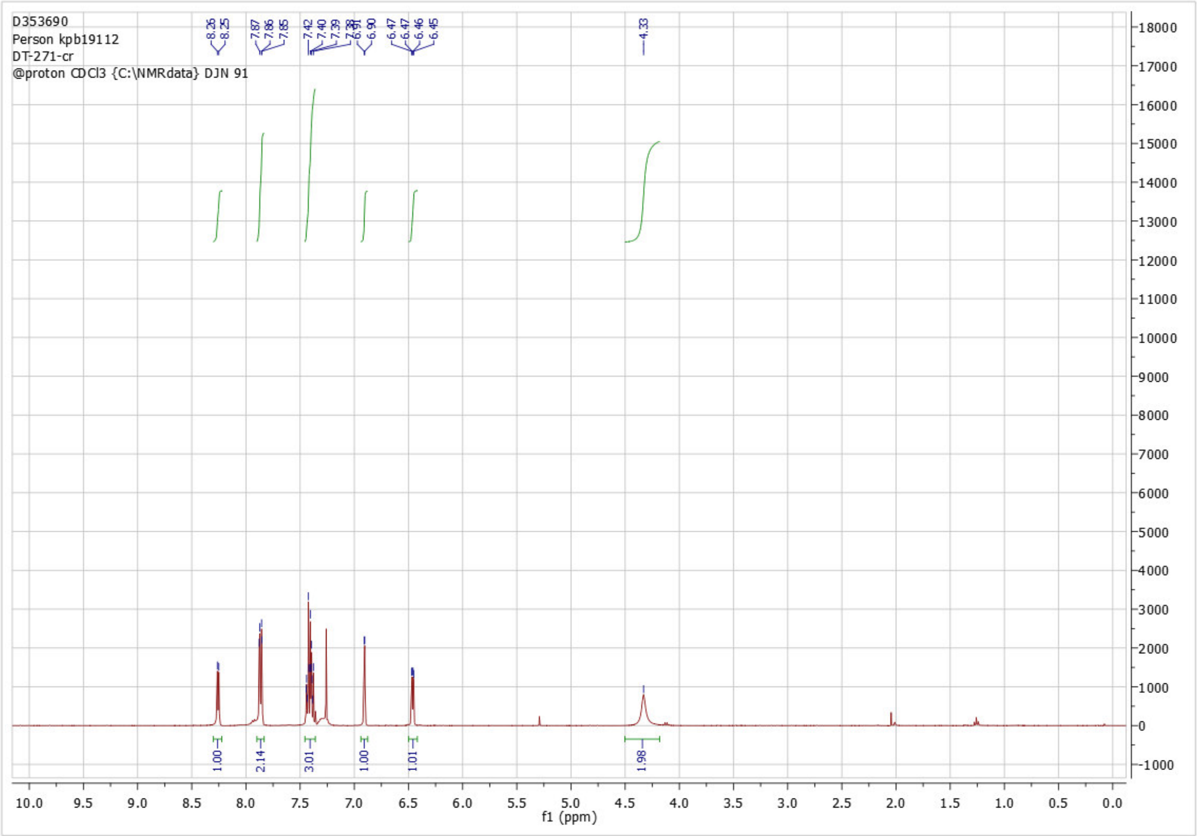

**4-amino-2-phenylpyidine: $^{13}C\{^1H\}$**

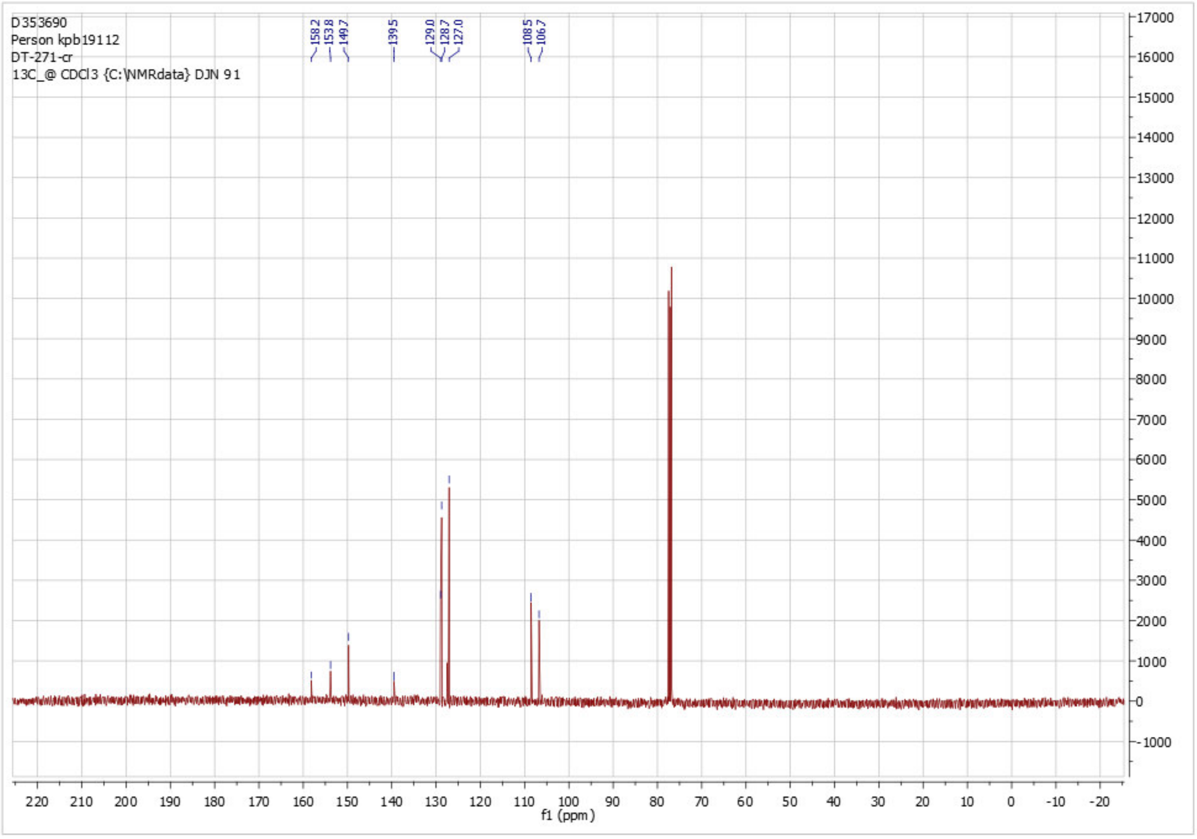

*Spectral details for unlabelled substrates used in this work:*

**Acetophenone**

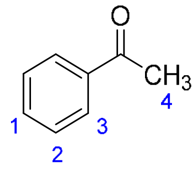


**$^{1}$H NMR** (400 MHz, $CDCl_3$) δ = 7.99 – 7.93 (m, 2H, H-3), 7.60 – 7.53 (m, 1H, H-1), 7.51 – 7.42 (m, 2H, H-2), 2.61 (s, 3H, H-4).
Incorporation expected at δ 7.99 – 7.93 ppm (H-3)
Determined against integral at δ 2.61 ppm (H-4)

**Ethylbenzoate**

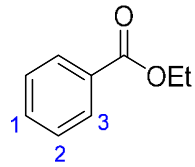


**$^{1}$H NMR** (400 MHz, $CDCl_3$) δ = 8.08 – 8.02 (m, 2H, H-3), 7.58 – 7.52 (m, 1H, H-1), 7.47 – 7.40 (m, 2H, H-2), 4.38 (q, *J* = 7.1 Hz, 2H, $CH_2$), 1.40 (t, *J* = 7.1 Hz, 3H, $CH_3$).
Incorporation expected at δ 8.07 – 8.03 ppm (H-3)
Determined against integral at δ 4.38 ppm ($OCH_2CH_3$)

**Nitrobenzene**

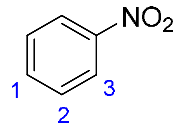


**$^{1}$H NMR** (400 MHz, $CDCl_3$) δ = 8.26 – 8.20 (m, 2H, H-3), 7.73 – 7.66 (m, 1H, H-1), 7.58 – 7.51 (m, 2H, H-2).
Incorporation expected at δ 8.26 – 8.20 ppm (H-3)
Determined against integral at δ 7.73 – 7.66 ppm (H-1)

**1-phenylpyrazole**

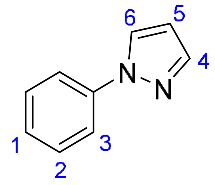


**$^{1}$H NMR** (400 MHz, $CDCl_3$) δ = 7.92 (d, *J* = 2.2 Hz, 1H, H-6), 7.75 – 7.68 (m, 3H, H-3 and H-4), 7.48 – 7.43 (m, 2H, H-2), 7.32 – 7.26 (m, 1H, H-1), 6.49 – 6.45 (m, 1H, H-5).
Incorporation expected at δ 7.75 – 7.68 ppm (H-3)
Determined against integral at δ 7.92 ppm (H-6)

**2-phenyloxozoline**

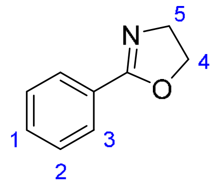


**$^{1}$H NMR** (400 MHz, $CDCl_3$) δ = 7.97 – 7.93 (m, 2H, H-3), 7.50 – 7.44 (m, 1H, H-1), 7.43 – 7.37 (m, 2H, H-2), 4.43 (t, *J* = 9.5 Hz, 2H, H-4), 4.06 (t, *J* = 9.5 Hz, 2H, H-5).
Incorporation expected at δ 7.97 – 7.93 ppm (H-3)
Determined against integral at δ 4.43 ppm (H-4)

**1-methyl-2-phenylimidazole**

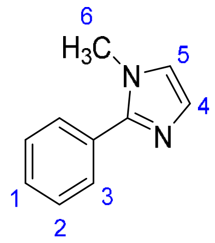


**$^{1}$H NMR** (400 MHz, $CDCl_3$) δ = 7.65 – 7.60 (m, 2H, H-3), 7.47 – 7.34 (m, 3H, H-1 and H-2), 7.12 (d, *J* = 1.2 Hz, 1H, H-5), 6.97 (d, *J* = 1.2 Hz, 1H, H-4), 3.19 (s, 3H, H-6).
Incorporation expected at δ 7.65 – 7.60 ppm (H-3)
Incorporation determined against δ 7.12 ppm (H-5)

**2-phenylpyrimidine**

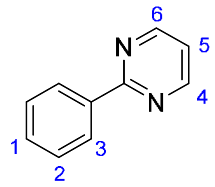


**$^{1}$H NMR (400 MHz, $CDCl_3$)**: δ 8.81 (d, 2H, *J* = 4.9 Hz, H-4), 8.48 – 8.43 (m, 2H, H-3), 7.52 – 7.48 (m, 3H, H-1 and H-2), 7.18 (t, 1H, *J* = 4.9 Hz, H-5)
Incorporation expected at δ 8.48 – 8.43 ppm (H-3)
Determined against integral at δ 7.18 ppm (H-5)

**2-phenylpyridines**

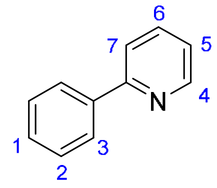


**$^1$H NMR** (400 MHz, $CDCl_3$) δ = 8.73 – 8.67 (m, 1H, H-4), 8.02 – 7.98 (m, 2H, H-3), 7.78 – 7.70 (m, 2H, H-6 and H-7), 7.51 – 7.45 (m, 2H, H-2), 7.45 – 7.39 (m, 1H, H-1), 7.25 – 7.21 (m, 1H, H-5).

Incorporation expected at δ 8.02 – 7.98 ppm (H-3)

Determined against integral at δ 8.73 – 8.67 ppm (H-4)

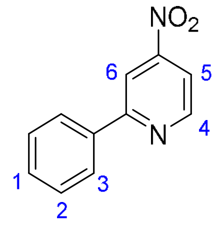


**$^1$H NMR** (400 MHz, $CDCl_3$) δ 8.97 (d, *J* = 5.3 Hz, 1H, H-4), 8.45 (d, *J* = 1.7 Hz, 1H, H-6), 8.12 – 8.06 (m, 2H, H-3), 7.94 (dd, *J* = 5.3, 2.0 Hz, 1H, H-5), 7.59 – 7.48 (m, 3H, H-1 and H-2).

Incorporation expected at δ 8.12 – 8.06 ppm (H-3)

Determined against integral at δ 8.97 (d, *J* = 5.3 Hz, 1H, H-4)

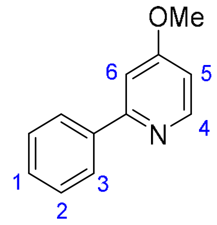


**$^1$H NMR** (400 MHz, $CDCl_3$) δ 8.52 (d, *J* = 5.7 Hz, 1H, H-4), 7.98 – 7.94 (m, 2H, H-3), 7.50 – 7.38 (m, 3H, H-1 and H-2), 7.23 (d, *J* = 2.4 Hz, 1H, H-6), 6.77 (dd, *J* = 5.7, 2.4 Hz, 1H, H-5), 3.90 (s, 3H, Me).

Incorporation expected at δ 7.98 – 7.94 ppm(H-3)

Determined against integral at δ 6.77 ppm (H-5)

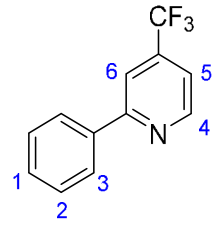


**$^1$H NMR** (400 MHz, $CDCl_3$) δ 8.87 (d, *J* = 5.1 Hz, 1H, H-4), 8.07 – 7.99 (m, 2H, H-3), 7.95 – 7.91 (m, 1H, H-6), 7.55 – 7.42 (m, 4H, H-1, H-2 and H-5).

Incorporation expected at δ 8.07 – 7.99 (H-3)

Determined against integral at δ 8.87 (H-4)

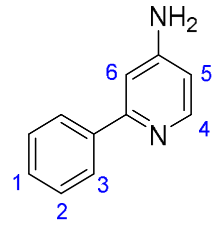


**$^1$H NMR** (400 MHz, $CDCl_3$) δ 8.26 (d, *J* = 5.6 Hz, 1H, H-4), 7.83 – 7.90 (m, 2H, H-3), 7.45 – 7.37 (m, 3H, H-1 and H-2), 6.91 (d, *J* = 2.1 Hz, 1H, H-6), 6.46 (dd, *J* = 5.6, 2.2 Hz, 1H, H-5).

Incorporation expected at δ 7.83 – 7.90 (H-3)

Determined against integral at δ 8.26 (H-4)

## 3. Kinetic data

### 3.1. General Kinetic Protocol for reaction monitoring by sampling

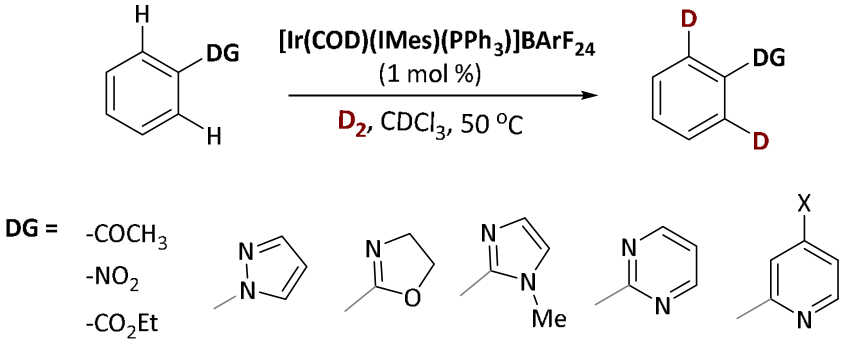


Ph-DG (0.50 mmol) and the iridium(I) pre-catalyst (0.005 mmol) were weighed into small vials. The solids were directly transferred to the reaction vial; any liquid substrates were first dissolved in a small amount of $CDCl_3$, the vial was washed with solvent, and the washings were transferred to the reaction vial. The reaction mixture was diluted using 2.5 mL (in total) of $CDCl_3$ ($[Ph\text{-}DG]_0$ = 0.20 mol/L). An aliquot was withdrawn to measure the initial spectrum and the vial was capped.

The solution was cooled in an acetone/dry ice bath and the headspace of the vial was evacuated and then refilled with deuterium gas (1 atm) from the balloon. After 3 vacuum/deuterium cycles, the reaction vial was removed from the cooling bath and placed in an aluminum block or thermostat-controlled water bath that had been preheated to 50 °C and the timer was started. The reaction mixture was then stirred vigorously (860 rpm) at 50 °C for 1-2 mins, allowing for catalyst activation and temperature equilibrium before the first aliquot was taken. The deuterium balloon was left in place for the duration of the reaction to ensure a continuous supply (and an excess) of $D_2$.
The aliquots (0.04 mL) of reaction mixture were removed at the specified intervals throughout the reaction *via* syringe to an NMR tube and diluted with 0.5 mL of $CDCl_3$. For GC-MS: the aliquots (0.005 mL) taken from the reaction were diluted with $CHCl_3$ prior to the analysis. For LC-MS: the $CDCl_3$ from the aliquots (0.005 mL) taken from the reaction was evaporated and the residue was dissolved in an acetonitrile/water mixture.

### 3.2. Mass spectrometry analysis for isotopologue distribution over time.

The distribution of $d_0$, $d_1$, and $d_2$ isotopologues in the products was determined by a liquid chromatography-mass spectrometry (LC-MS) for heterocyclic DGs and gas chromatography-mass spectrometry (GC-MS) for acetophenone, nitrobenzene and ethyl benzoate by analysing the aliquots taken from the HIE reactions performed following general procedure.

The distribution of $d_0$, $d_1$, and $d_2$ substrates was determined from the corresponding normalised relative abundances at peaks of [M] ($d_0$), [M+1] ($d_1$), [M+2] ($d_2$) for GC-MS and at peaks [M+H] ($d_0$), [M+H+1] ($d_1$), [M+H+2] ($d_2$) for positive ion mode LC-MS analysis for all the heterocyclic substrates apart from 4-nitro-2-phenylpyridine where the negative ion mode was used to determine the relative abundances at peaks of [M-H] ($d_0$), [M-H+1] ($d_1$), [M-H+2] ($d_2$).

The observed relative isotopic abundances were corrected with regard to the amount of $^{13}C$ isotope (1.1 % natural abundance). The normalised abundances were obtained by dividing those relative numbers by the total abundance.

The overall level of deuterium incorporation in each substrate was determined according to Equation 1, using the relative peak abundances ($d_0$, $d_1$, or $d_2$) for each substrate:

$$\% \, Deuteration \text{ (mass spectrometry)} = (0.5 \times d_1 ) + d_2 \quad (1)$$

The values were in good agreement with those obtained from $^1H$ NMR spectra (calculated using Equation 2).

$$\% \, Deuteration \text{ (NMR)} = 100 - [\left(\frac{residual\ integral}{number\ of\ labelling\ sites}\right) \times \ 100] \quad (2)$$

The levels of deuterium incorporation derived from both methods are in good agreement.

**Table S1**. The extent of deuterium labelling and the distribution of $d_0$, $d_1$, and $d_2$ in acetophenone as determined by GC-MS analysis.

Following the General Kinetic Protocol using 60.1 mg of acetophenone, 8.7 mg of Ir-catalyst.

| *Observed relative abundances* | | | | | | | | | |
|---|---|---|---|---|---|---|---|---|---|
| | | m/z | **Ph(H$_2$)DG** | **10 min** | **20 min** | **30 min** | **60 min** | **90 min** | **120 min** |
| M | $d_0$ | 120 | 100.0 | 100.0 | 80.5 | 37.6 | 35.0 | 21.9 | 15.8 |
| M+1 | $d_1$ | 121 | 8.8 | 35.5 | 100.0 | 100.0 | 100.0 | 94.6 | 79.8 |
| M+2 | $d_2$ | 122 | 0.6 | 9.0 | 29.6 | 53.0 | 79.7 | 100.0 | 100.0 |
| *Relative abundances adjusted for natural abundance of isotopes* | | | | | | | | | |
| | | m/z | **Ph(H$_2$)DG** | **10 min** | **20 min** | **30 min** | **60 min** | **90 min** | **120 min** |
| M | $d_0$ | 120 | 100.0 | 100.0 | 100.0 | 100.0 | 35.0 | 21.9 | 15.8 |
| M+1 | $d_1$ | 121 | 0.0 | 26.5 | 92.8 | 96.6 | 96.9 | 92.6 | 78.4 |
| M+2 | $d_2$ | 122 | 0.0 | 4.8 | 19.8 | 43.6 | 70.4 | 91.3 | 92.7 |
| Total abundance | | | 100.0 | 131.3 | 212.5 | 240.3 | 202.2 | 205.8 | 186.8 |
| *Normalised relative abundances* | | | | | | | | | |
| | | m/z | **Ph(H$_2$)DG** | **10 min** | **20 min** | **30 min** | **60 min** | **90 min** | **120 min** |
| M | $d_0$ | 120 | 100.0 | 76.1 | 47.0 | 41.6 | 17.3 | 10.6 | 8.5 |
| M+1 | $d_1$ | 121 | 0.0 | 20.2 | 43.6 | 40.2 | 47.9 | 45.0 | 41.9 |
| M+2 | $d_2$ | 122 | 0.0 | 3.6 | 9.3 | 18.2 | 34.8 | 44.4 | 49.6 |
| *Level of deuterium incorporation* | | | | | | | | | |
| | | | **Ph(H$_2$)DG** | **10 min** | **20 min** | **30 min** | **60 min** | **90 min** | **120 min** |
| %D by GC-MS | | | 0 | 14 | 31 | 38 | 59 | 67 | 71 |
| %D by NMR | | | 0 | 18 | 32 | 44 | 60 | 67 | 71 |

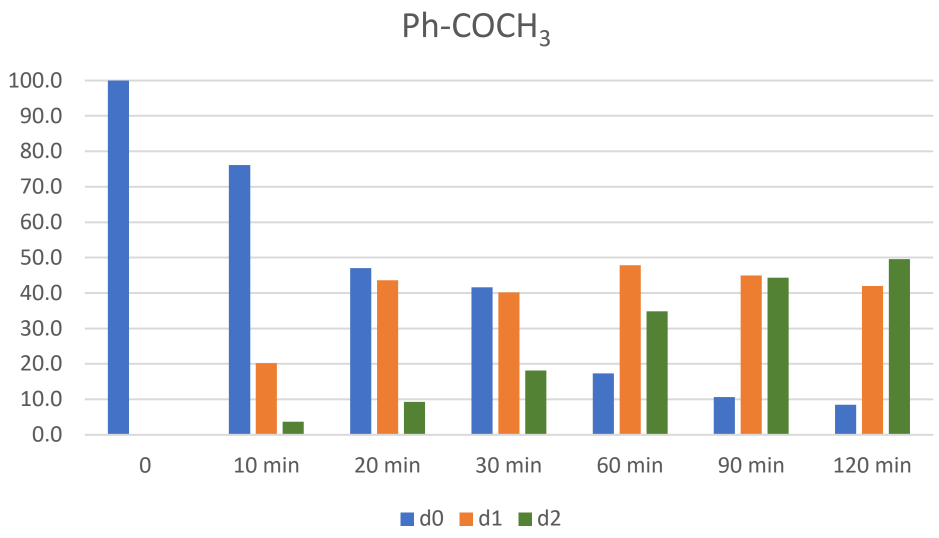


**Figure S1.** The distribution of $d_0$, $d_1$, and $d_2$ during acetophenone labelling as determined by GC-MS analysis**.**

**Table S2**. The extent of deuterium labelling and the distribution of $d_0$, $d_1$, and $d_2$ in nitrobenzene as determined by GC-MS analysis.

Following the General Kinetic Protocol using 61.5 mg of nitrobenzene, 8.7 mg of Ir-catalyst.

| *Observed relative abundances* | | | | | | | | | | | | |
|---|---|---|---|---|---|---|---|---|---|---|---|---|
| | | m/z | **Ph(H$_2$)DG** | **5 min** | **10 min** | **20 min** | **30 min** | **60 min** | **90 min** | **120 min** | **180 min** | **270 min** |
| M | $d_0$ | 123 | 100.0 | 100.0 | 100.0 | 100.0 | 98.9 | 57.7 | 34.5 | 28.2 | 11.2 | 8.1 |
| M+1 | $d_1$ | 124 | 7.1 | 32.6 | 41.2 | 66.6 | 100.0 | 100.0 | 100.0 | 100.0 | 81.8 | 62.4 |
| M+2 | $d_2$ | 125 | 0.8 | 3.5 | 5.9 | 13.9 | 24.8 | 40.8 | 64.3 | 89.7 | 100.0 | 100.0 |
| *Relative abundances adjusted for natural abundance of isotopes* | | | | | | | | | | | | |
| | | m/z | **Ph(H$_2$)DG** | **5 min** | **10 min** | **20 min** | **30 min** | **60 min** | **90 min** | **120 min** | **180 min** | **270 min** |
| M | $d_0$ | 123 | 100.0 | 100.0 | 100.0 | 100.0 | 98.9 | 57.7 | 34.5 | 28.2 | 11.2 | 8.1 |
| M+1 | $d_1$ | 124 | 0.0 | 25.5 | 34.1 | 59.5 | 93.0 | 95.9 | 97.6 | 98.0 | 81.0 | 61.8 |
| M+2 | $d_2$ | 125 | 0.0 | 0.4 | 2.2 | 8.4 | 17.0 | 33.4 | 57.1 | 82.5 | 94.2 | 95.6 |
| Total abundance | | | 100.0 | 126.0 | 136.3 | 168.0 | 208.9 | 187.0 | 189.1 | 208.7 | 186.5 | 165.5 |
| *Normalised relative abundances* | | | | | | | | | | | | |
| | % | m/z | **Ph(H$_2$)DG** | **5 min** | **10 min** | **20 min** | **30 min** | **60 min** | **90 min** | **120 min** | **180 min** | **270 min** |
| M | $d_0$ | 123 | 100.0 | 79.4 | 73.4 | 59.5 | 47.4 | 30.8 | 18.2 | 13.5 | 6.0 | 4.9 |
| M+1 | $d_1$ | 124 | 0.0 | 20.3 | 25.0 | 35.4 | 44.5 | 51.3 | 51.6 | 47.0 | 43.5 | 37.3 |
| M+2 | $d_2$ | 125 | 0.0 | 0.3 | 1.6 | 5.0 | 8.1 | 17.9 | 30.2 | 39.5 | 50.5 | 57.8 |
| *Level of deuterium incorporation* | | | | | | | | | | | | |
| | | | **Ph(H$_2$)DG** | **5 min** | **10 min** | **20 min** | **30 min** | **60 min** | **90 min** | **120 min** | **180 min** | **270 min** |
| %D by GC-MS | | | 0 | 10 | 14 | 23 | 30 | 43 | 56 | 63 | 72 | 76 |
| %D by NMR | | | 0 | 12 | 16 | 24 | 31 | 45 | 56 | 63 | 71 | 76 |

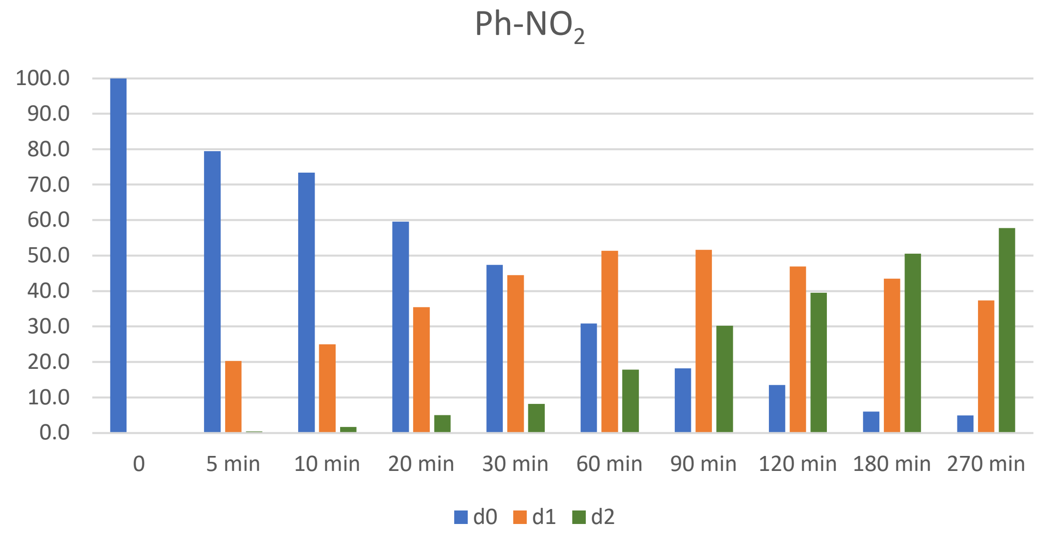


**Figure S2.** The distribution of $d_0$, $d_1$, and $d_2$ during nitrobenzene labelling as determined by GC-MS analysis**.**

**Table S3**. The extent of deuterium labelling and the distribution of $d_0$, $d_1$, and $d_2$ in ethyl benzoate as determined by GC-MS analysis.

Following the General Kinetic Protocol using 75.1 mg of ethyl benzoate, 8.7 mg of Ir-catalyst.

| *Observed relative abundances* | | | | | | | | | | |
|---|---|---|---|---|---|---|---|---|---|---|
| | | m/z | **Ph($H_2$)DG** | **10 min** | **20 min** | **30 min** | **60 min** | **90 min** | **120 min** | **200 min** |
| M | $d_0$ | 150 | 100.0 | 100.0 | 100.0 | 100.0 | 54.2 | 43.1 | 23.1 | 24.5 |
| M+1 | $d_1$ | 151 | 9.9 | 37.7 | 68.6 | 102.0 | 100.0 | 100.0 | 100.0 | 100.0 |
| M+2 | $d_2$ | 152 | 0.6 | 5.8 | 16.4 | 31.0 | 56.7 | 68.1 | 95.4 | 166.2 |
| *Relative abundances adjusted for natural abundance of isotopes* | | | | | | | | | | |
| | | m/z | **Ph($H_2$)DG** | **10 min** | **20 min** | **30 min** | **60 min** | **90 min** | **120 min** | **200 min** |
| M | $d_0$ | 150 | 100.0 | 100.0 | 100.0 | 100.0 | 54.2 | 43.1 | 23.1 | 24.5 |
| M+1 | $d_1$ | 151 | 0.0 | 27.7 | 58.6 | 92.0 | 94.6 | 95.7 | 97.7 | 97.6 |
| M+2 | $d_2$ | 152 | 0.0 | 1.1 | 8.5 | 19.8 | 46.1 | 57.6 | 85.2 | 156.0 |
| Total abundance | | | 100.0 | 128.7 | 167.1 | 211.9 | 194.9 | 196.4 | 206.0 | 278.0 |
| *Normalised relative abundances* | | | | | | | | | | |
| | | m/z | **Ph($H_2$)DG** | **10 min** | **20 min** | **30 min** | **60 min** | **90 min** | **120 min** | **200 min** |
| M | $d_0$ | 150 | 100.0 | 77.7 | 59.8 | 47.2 | 27.8 | 22.0 | 11.2 | 8.8 |
| M+1 | $d_1$ | 151 | 0.0 | 21.5 | 35.1 | 43.4 | 48.5 | 48.7 | 47.4 | 35.1 |
| M+2 | $d_2$ | 152 | 0.0 | 0.8 | 5.1 | 9.4 | 23.7 | 29.3 | 41.3 | 56.1 |
| *Level of deuterium incorporation* | | | | | | | | | | |
| | | | **Ph($H_2$)DG** | **10 min** | **20 min** | **30 min** | **60 min** | **90 min** | **120 min** | **200 min** |
| %D by GC-MS | | | 0 | 12 | 23 | 31 | 48 | 54 | 65 | 74 |
| %D by NMR | | | 0 | 14 | 25 | 33 | 50 | 58 | 65 | 78 |

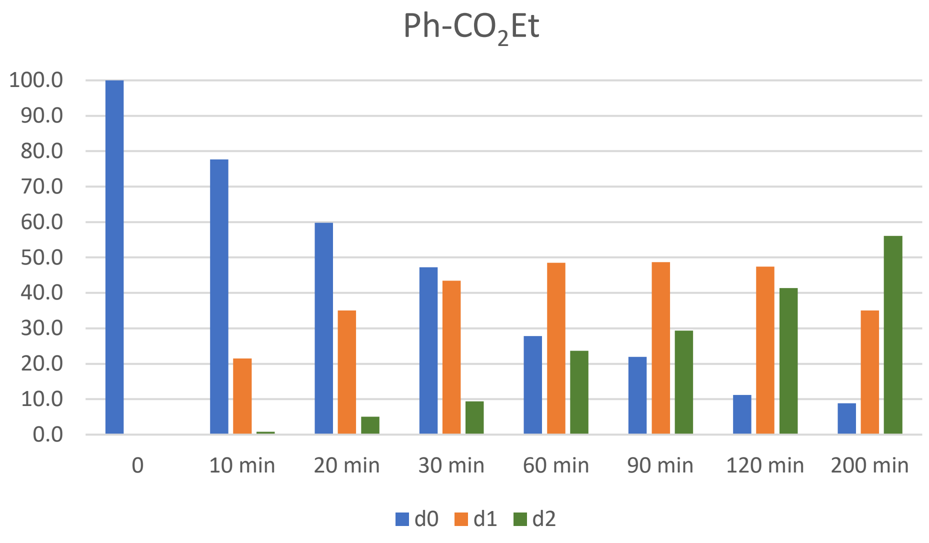


**Figure S3.** The distribution of $d_0$, $d_1$, and $d_2$ during ethyl benzoate labelling as determined by GC-MS analysis**.**

**Table S4**. The extent of deuterium labelling and the distribution of $d_0$, $d_1$, and $d_2$ in 2-phenylpyridine as determined by LC-MS analysis.

Following the General Kinetic Protocol using 77.6 mg of 2-phenylpyridine, 8.7 mg of Ir-catalyst.

| *Observed relative abundances* | | | | | | | | | |
|---|---|---|---|---|---|---|---|---|---|
| | | m/z | **Ph(H$_2$)DG** | **30 min** | **60 min** | **90 min** | **120 min** | **180 min** | **240 min** |
| (M+H) | $d_0$ | 156 | 100.0 | 100.0 | 100.0 | 100.0 | 100.0 | 52.5 | 36.6 |
| (M+H)+1 | $d_1$ | 157 | 13.3 | 23.5 | 34.3 | 42.2 | 59.3 | 51.7 | 55.0 |
| (M+H)+2 | $d_2$ | 158 | 0.9 | 18.6 | 43.4 | 66.1 | 95.5 | 100.0 | 100.0 |
| *Relative abundances adjusted for natural abundance of isotopes* | | | | | | | | | |
| | | m/z | **Ph(H$_2$)DG** | **30 min** | **60 min** | **90 min** | **120 min** | **180 min** | **240 min** |
| (M+H) | $d_0$ | 156 | 100.0 | 100.0 | 100.0 | 100.0 | 100.0 | 52.5 | 36.6 |
| (M+H)+1 | $d_1$ | 157 | 0.0 | 10.2 | 21.0 | 28.9 | 46.0 | 44.7 | 50.1 |
| (M+H)+2 | $d_2$ | 158 | 0.0 | 14.6 | 37.9 | 59.6 | 86.7 | 92.7 | 92.4 |
| Total abundance | | | 100.0 | 124.8 | 158.9 | 188.5 | 232.7 | 189.9 | 179.1 |
| *Normalised relative abundances* | | | | | | | | | |
| | | m/z | **Ph(H$_2$)DG** | **30 min** | **60 min** | **90 min** | **120 min** | **180 min** | **240 min** |
| (M+H) | $d_0$ | 156 | 100.0 | 80.1 | 62.9 | 53.1 | 43.0 | 27.7 | 20.4 |
| (M+H)+1 | $d_1$ | 157 | 0.0 | 8.2 | 13.2 | 15.3 | 19.8 | 23.6 | 28.0 |
| (M+H)+2 | $d_2$ | 158 | 0.0 | 11.7 | 23.9 | 31.6 | 37.3 | 48.8 | 51.6 |
| *Level of deuterium incorporation* | | | | | | | | | |
| | | | **Ph(H$_2$)DG** | **30 min** | **60 min** | **90 min** | **120 min** | **180 min** | **240 min** |
| %D by LC-MS | | | 0 | 16 | 30 | 39 | 47 | 61 | 66 |
| %D by NMR | | | 0 | 14 | 27 | 37 | 46 | 58 | 67 |

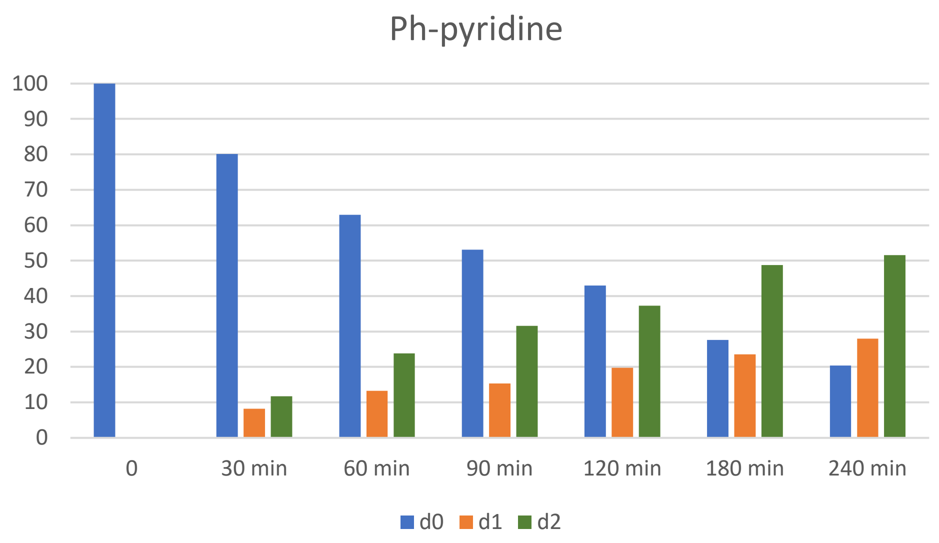


**Figure S4**. The distribution of $d_0$, $d_1$, and $d_2$ during 2-phenylpyridine labelling as determined by LC-MS analysis**.**

**Table S5**. The extent of deuterium labelling and the distribution of $d_0$, $d_1$, and $d_2$ in 1-methyl-2-phenylimidazole as determined by LC-MS analysis.

| Following the General Kinetic Protocol using 79.1 mg of 1-methyl-2-phenylimidazole, 8.7 mg of Ir-catalyst. *Observed relative abundances* | | | | | | | | | |
|---|---|---|---|---|---|---|---|---|---|
| | | m/z | **Ph(H$_2$)DG** | **10 min** | **20 min** | **30 min** | **60 min** | **90 min** | **120 min** |
| (M+H) | $d_0$ | 159 | 100.0 | 100.0 | 98.7 | 64.2 | 29.5 | 8.8 | 3.6 |
| (M+H)+1 | $d_1$ | 160 | 13.3 | 49.5 | 100.0 | 100.0 | 100.0 | 54.2 | 37.1 |
| (M+H)+2 | $d_2$ | 161 | 0.9 | 10.6 | 32.8 | 42.5 | 90.1 | 100.0 | 100.0 |
| *Relative abundances adjusted for natural abundance of isotopes* | | | | | | | | | |
| | | m/z | **Ph(H$_2$)DG** | **10 min** | **20 min** | **30 min** | **60 min** | **90 min** | **120 min** |
| (M+H) | $d_0$ | 159 | 100.0 | 100.0 | 98.7 | 64.2 | 29.5 | 8.8 | 3.6 |
| (M+H)+1 | $d_1$ | 160 | 0.0 | 37.5 | 88.2 | 92.3 | 96.5 | 53.1 | 36.7 |
| (M+H)+2 | $d_2$ | 161 | 0.0 | 3.7 | 19.8 | 29.9 | 77.8 | 93.4 | 95.5 |
| Total abundance | | | 100.0 | 141.2 | 206.7 | 186.4 | 203.8 | 155.4 | 135.8 |
| *Normalised relative abundances* | | | | | | | | | |
| | | m/z | **Ph(H$_2$)DG** | **10 min** | **20 min** | **30 min** | **60 min** | **90 min** | **120 min** |
| (M+H) | $d_0$ | 159 | 100.0 | 70.8 | 47.8 | 34.5 | 14.5 | 5.7 | 2.7 |
| (M+H)+1 | $d_1$ | 160 | 0.0 | 26.6 | 42.7 | 49.5 | 47.3 | 34.2 | 27.0 |
| (M+H)+2 | $d_2$ | 161 | 0.0 | 2.6 | 9.6 | 16.0 | 38.2 | 60.1 | 70.3 |
| *Level of deuterium incorporation* | | | | | | | | | |
| | | | **Ph(H$_2$)DG** | **10 min** | **20 min** | **30 min** | **60 min** | **90 min** | **120 min** |
| %D by LC-MS | | | 0 | 16 | 31 | 41 | 62 | 77 | 84 |
| %D by NMR | | | 0 | 11 | 25 | 36 | 57 | 72 | 83 |

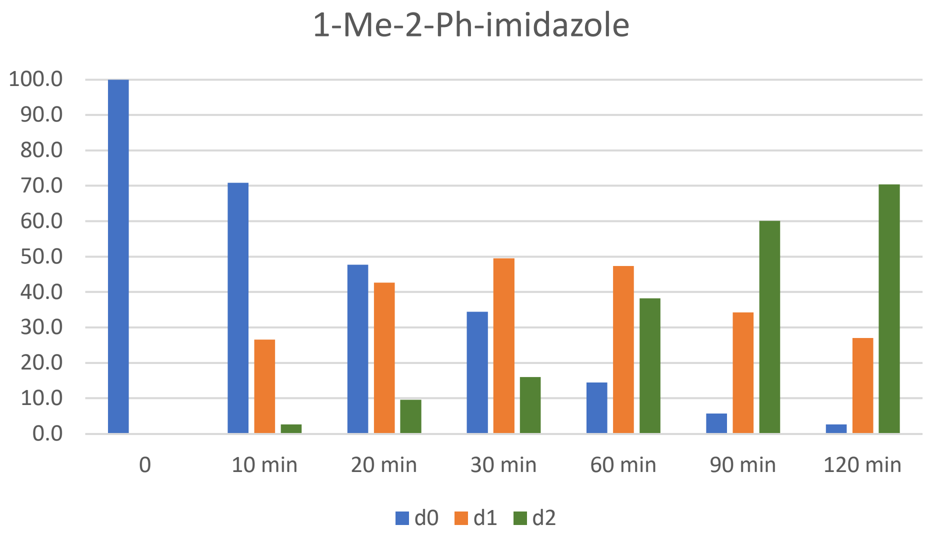


**Figure S5**. The distribution of $d_0$, $d_1$, and $d_2$ during 1-methyl-2-phenylimidazole labelling as determined by LC-MS analysis.

**Table S6**. The extent of deuterium labelling and the distribution of $d_0$, $d_1$, and $d_2$ in 2-phenylpyrimidine as determined by LC-MS analysis.

Following the General Kinetic Protocol using 78.1 mg of 2-phenylpyrimidine, 8.7 mg of Ir-catalyst.

| | | | | | | | | | | | |
|---|---|---|---|---|---|---|---|---|---|---|---|
| *Observed relative abundances* | | | | | | | | | | | |
| | | m/z | **Ph(H$_2$)DG** | **10 min** | **20 min** | **30 min** | **40 min** | **50 min** | **60 min** | **90 min** | **120 min** |
| (M+H) | $d_0$ | 157 | 100 | 100.0 | 100.0 | 100.0 | 100.0 | 100.0 | 100.0 | 98.8 | 63.7 |
| (M+H)+1 | $d_1$ | 158 | 11.9 | 18.7 | 25.5 | 34.1 | 42.4 | 50.2 | 69.9 | 100.0 | 100.0 |
| (M+H)+2 | $d_2$ | 159 | 0.7 | 8.2 | 11.7 | 15.8 | 25.0 | 35.3 | 50.0 | 97.2 | 96.3 |
| *Relative abundances adjusted for natural abundance of isotopes* | | | | | | | | | | | |
| | | m/z | **Ph(H$_2$)DG** | **10 min** | **20 min** | **30 min** | **40 min** | **50 min** | **60 min** | **90 min** | **120 min** |
| (M+H) | $d_0$ | 157 | 100.0 | 100.0 | 100.0 | 100.0 | 100.0 | 100.0 | 100.0 | 98.8 | 63.7 |
| (M+H)+1 | $d_1$ | 158 | 0.0 | 6.8 | 13.6 | 22.2 | 30.5 | 38.3 | 58.0 | 88.2 | 92.4 |
| (M+H)+2 | $d_2$ | 159 | 0.0 | 5.3 | 8.0 | 11.0 | 19.3 | 28.6 | 41.0 | 84.6 | 84.0 |
| Total abundance | | | 100.0 | 112.1 | 121.6 | 133.2 | 149.8 | 166.9 | 199.0 | 271.7 | 240.1 |
| *Normalised relative abundances* | | | | | | | | | | | |
| | | m/z | **Ph(H$_2$)DG** | **10 min** | **20 min** | **30 min** | **40 min** | **50 min** | **60 min** | **90 min** | **120 min** |
| (M+H) | $d_0$ | 157 | 100.0 | 89.2 | 82.3 | 75.1 | 66.8 | 59.9 | 50.3 | 36.4 | 26.5 |
| (M+H)+1 | $d_1$ | 158 | 0.0 | 6.1 | 11.2 | 16.7 | 20.4 | 22.9 | 29.1 | 32.5 | 38.5 |
| (M+H)+2 | $d_2$ | 159 | 0.0 | 4.7 | 6.6 | 8.3 | 12.9 | 17.1 | 20.6 | 31.1 | 35.0 |
| *Level of deuterium incorporation* | | | | | | | | | | | |
| | | | **Ph(H$_2$)DG** | **10 min** | **20 min** | **30 min** | **40 min** | **50 min** | **60 min** | **90 min** | **120 min** |
| %D by LC-MS | | | 0 | 8 | 12 | 17 | 23 | 29 | 35 | 47 | 54 |
| %D by NMR | | | 0 | 9 | 14 | 17 | 24 | 29 | 35 | 44 | 53 |

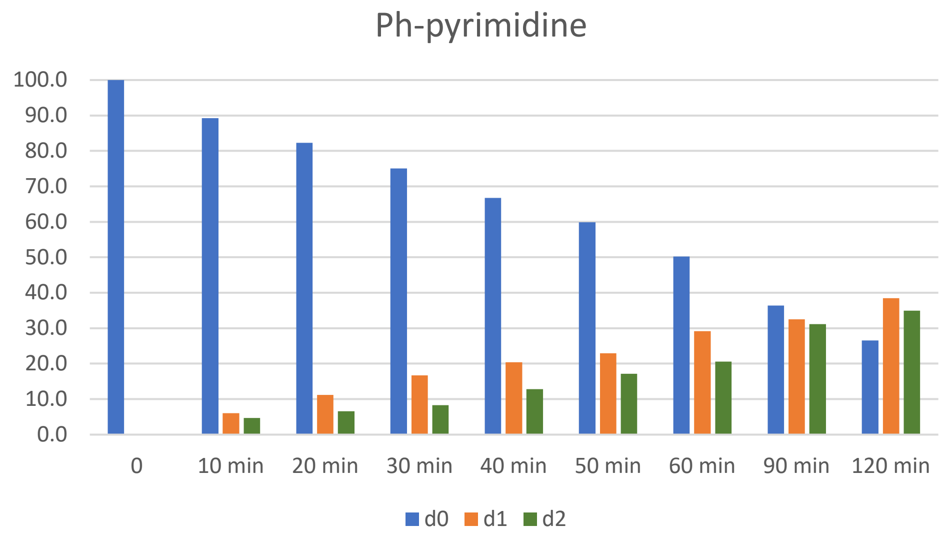


**Figure S6**. The distribution of $d_0$, $d_1$, and $d_2$ during 2-phenylpyrimidine labelling as determined by LC-MS analysis**.**

**Table S7**. The extent of deuterium labelling and the distribution of $d_0$, $d_1$, and $d_2$ in 2-phenylpyrazole as determined by LC-MS analysis.

Following the General Kinetic Protocol using 72.1 mg of 2-phenylpyrazole, 8.7 mg of Ir-catalyst.

| | | | | | | | | |
|---|---|---|---|---|---|---|---|---|
| *Observed relative abundances* | | | | | | | | |
| | | m/z | **Ph(H$_2$)DG** | **10 min** | **20 min** | **30 min** | **60 min** | **120 min** |
| (M+H) | $d_0$ | 145 | 100 | 100.0 | 63.1 | 49.2 | 32.8 | 22.9 |
| (M+H)+1 | $d_1$ | 146 | 11.2 | 74.4 | 100.0 | 100.0 | 100.0 | 95.2 |
| (M+H)+2 | $d_2$ | 147 | 0.5 | 13.3 | 45.6 | 60.9 | 94.6 | 100.0 |
| *Relative abundances adjusted for natural abundance of isotopes* | | | | | | | | |
| | | m/z | **Ph(H$_2$)DG** | **10 min** | **20 min** | **30 min** | **60 min** | **120 min** |
| (M+H) | $d_0$ | 145 | 100.0 | 100.0 | 63.1 | 49.2 | 32.8 | 22.9 |
| (M+H)+1 | $d_1$ | 146 | 0.0 | 63.2 | 92.9 | 94.5 | 96.3 | 92.6 |
| (M+H)+2 | $d_2$ | 147 | 0.0 | 4.5 | 34.1 | 49.5 | 83.2 | 89.2 |
| Total abundance | | | 100.0 | 167.7 | 190.1 | 193.1 | 212.4 | 204.8 |
| *Normalised relative abundances* | | | | | | | | |
| | | m/z | **Ph(H$_2$)DG** | **10 min** | **20 min** | **30 min** | **60 min** | **120 min** |
| (M+H) | $d_0$ | 145 | 100.0 | 59.6 | 33.2 | 25.5 | 15.4 | 11.2 |
| (M+H)+1 | $d_1$ | 146 | 0.0 | 37.7 | 48.9 | 48.9 | 45.4 | 45.2 |
| (M+H)+2 | $d_2$ | 147 | 0.0 | 2.7 | 17.9 | 25.6 | 39.2 | 43.6 |
| *Level of deuterium incorporation* | | | | | | | | |
| | | | **Ph(H$_2$)DG** | **10 min** | **20 min** | **30 min** | **60 min** | **120 min** |
| %D by LC-MS | | | 0 | 22 | 42 | 50 | 62 | 66 |
| %D by NMR | | | 0 | 20 | 36 | 46 | 58 | 62 |

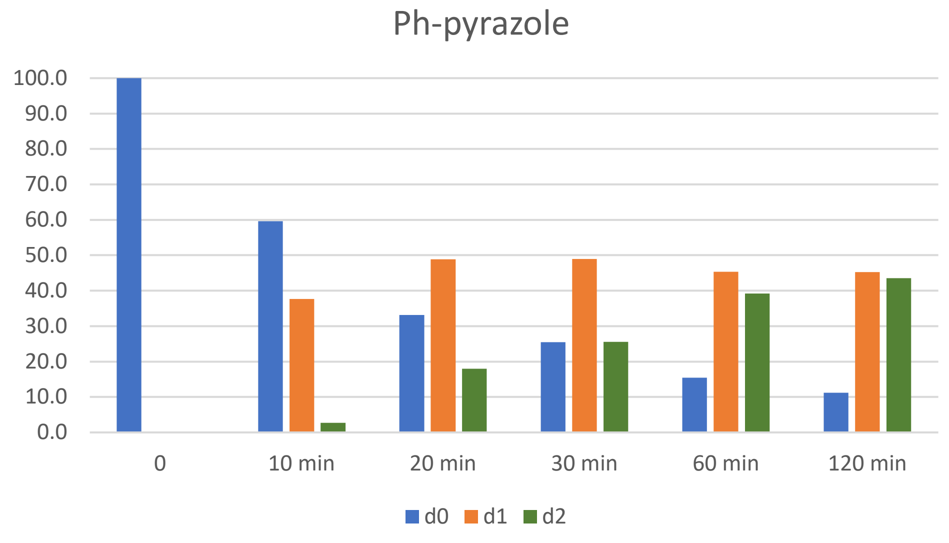


**Figure S7**. The distribution of $d_0$, $d_1$, and $d_2$ during 2-phenylpyrazole labelling as determined by LC-MS analysis**.**

**Table S8**. The extent of deuterium labelling and the distribution of $d_0$, $d_1$, and $d_2$ in 2-phenyloxazoline as determined by LC-MS analysis.

Following the General Kinetic Protocol using 73.6 mg of 2-phenyloxazoline, 8.7 mg of Ir-catalyst.

| *Observed relative abundances* | | | | | | | | | | |
|---|---|---|---|---|---|---|---|---|---|---|
| | | m/z | **Ph(H$_2$)DG** | **5 min** | **10 min** | **20 min** | **30 min** | **40 min** | **60 min** | **80 min** |
| (M+H) | $d_0$ | 147 | 100 | 100.0 | 100.0 | 59.5 | 40.9 | 30.6 | 18.3 | 14.2 |
| (M+H)+1 | $d_1$ | 148 | 10.5 | 46.9 | 99.3 | 100.0 | 100.0 | 100.0 | 84.2 | 73.6 |
| (M+H)+2 | $d_2$ | 149 | 0.8 | 7.8 | 28.6 | 49.4 | 70.7 | 84.5 | 100.0 | 100.0 |
| *Relative abundances adjusted for natural abundance of isotopes* | | | | | | | | | | |
| | | m/z | **Ph(H$_2$)DG** | **5 min** | **10 min** | **20 min** | **30 min** | **40 min** | **60 min** | **80 min** |
| (M+H) | $d_0$ | 147 | 100 | 100.0 | 100.0 | 59.5 | 40.9 | 30.6 | 18.3 | 14.2 |
| (M+H)+1 | $d_1$ | 148 | 10.5 | 46.9 | 99.3 | 100.0 | 100.0 | 100.0 | 84.2 | 73.6 |
| (M+H)+2 | $d_2$ | 149 | 0.8 | 7.8 | 28.6 | 49.4 | 70.7 | 84.5 | 100.0 | 100.0 |
| Total abundance | | | 100 | 100.0 | 100.0 | 59.5 | 40.9 | 30.6 | 18.3 | 14.2 |
| *Normalised relative abundances* | | | | | | | | | | |
| | | m/z | **Ph(H$_2$)DG** | **5 min** | **10 min** | **20 min** | **30 min** | **40 min** | **60 min** | **80 min** |
| (M+H) | $d_0$ | 147 | 100.0 | 72.2 | 48.5 | 31.0 | 20.8 | 15.2 | 9.6 | 8.0 |
| (M+H)+1 | $d_1$ | 148 | 0.0 | 26.3 | 43.1 | 48.9 | 48.7 | 48.1 | 42.9 | 40.5 |
| (M+H)+2 | $d_2$ | 149 | 0.0 | 1.5 | 8.4 | 20.0 | 30.5 | 36.7 | 47.5 | 51.5 |
| *Level of deuterium incorporation* | | | | | | | | | | |
| | | | **Ph(H$_2$)DG** | **5 min** | **10 min** | **20 min** | **30 min** | **40 min** | **60 min** | **80 min** |
| %D by LC-MS | | | 0 | 15 | 30 | 45 | 55 | 61 | 69 | 72 |
| %D by NMR | | | 0 | 16 | 29 | 44 | 54 | 61 | 67 | 69 |

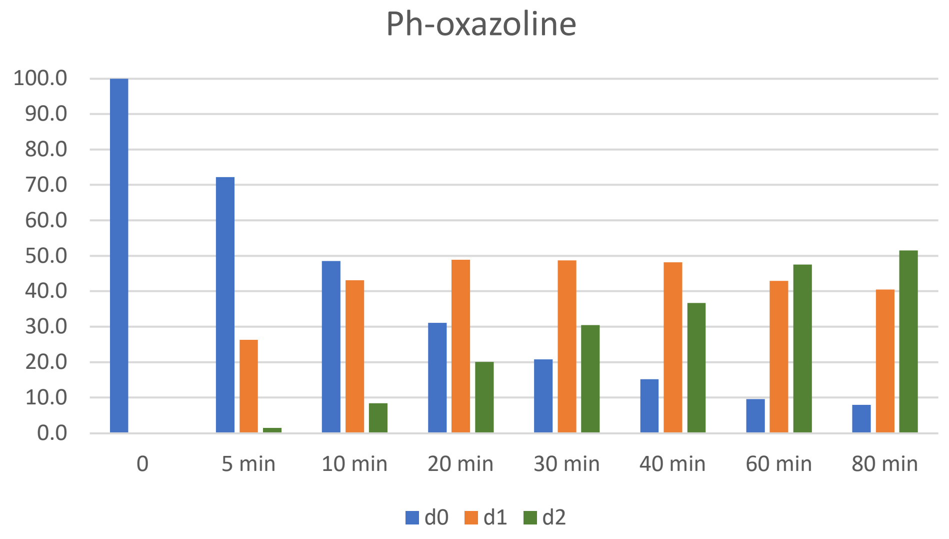


**Figure S8**. The distribution of $d_0$, $d_1$, and $d_2$ during 2-phenyloxazoline labelling as determined by LC-MS analysis**.**

## 3.3. Kinetic data and isotopologue distribution for substituted phenylpyridines

**Table S9.** Rate monitoring for the deuteration of 4-nitro-2-phenyl pyridine.

Following the General Kinetic Protocol using 100.1 mg of 4-nitro-2-phenyl pyridine, 8.7 mg of Ir-catalyst.

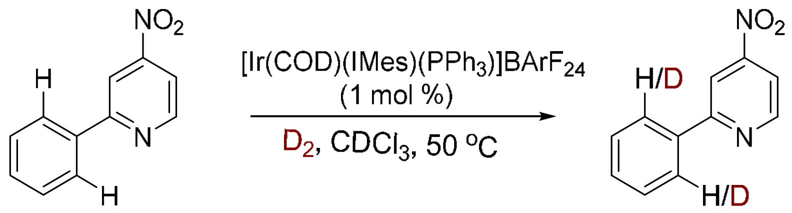


| Entry | Time, s | Integral (H/D) | [Substrate], M | ln [Substrate] |
|---|---|---|---|---|
| 0 | 0 | 2.00 | 0.200 | -1.61 |
| 1 | 600 | 1.76 | 0.176 | -1.74 |
| 2 | 1200 | 1.67 | 0.167 | -1.79 |
| 3 | 1800 | 1.57 | 0.157 | -1.85 |
| 4 | 3600 | 1.34 | 0.134 | -2.01 |
| 5 | 7200 | 1.04 | 0.104 | -2.26 |
| 6 | 10800 | 0.82 | 0.082 | -2.50 |
| 7 | 14400 | 0.69 | 0.069 | -2.67 |

**$k_{obs}$ = 1.44 × $10^{-4}$ ($s^{-1}$)**

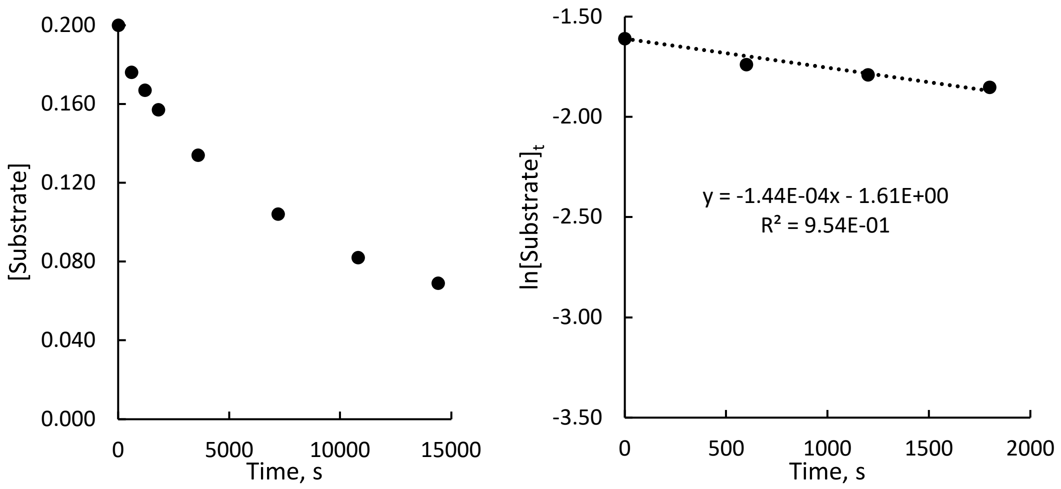


**Figure S9** Kinetic profile for the HIE reaction of 4-nitro-2-phenyl pyridine over time in $CDCl_3$ at 50 °C (left). Fitting a first-order kinetic model to the data (right).

**Table S10**. The extent of deuterium labelling and the distribution of $d_0$, $d_1$, and $d_2$ in 4-nitro-2-phenyl pyridine as determined by LC-MS analysis (Data from experiment in Table S9).

| | | | | | | | |
|---|---|---|---|---|---|---|---|
| *Observed relative abundances* | | | | | | | |
| | | m/z | **Ph(H$_2$)DG** | **60 min** | **120 min** | **180 min** | **240 min** |
| (M-H) | $d_0$ | 200 | 100.0 | 100.0 | 50.8 | 34.8 | 15.0 |
| (M-H)+1 | $d_1$ | 201 | 15.1 | 98.5 | 100.0 | 100.0 | 94.7 |
| (M-H)+2 | $d_2$ | 202 | 1.4 | 51.7 | 59.6 | 93.3 | 100.0 |
| *Relative abundances adjusted for natural abundance of isotopes* | | | | | | | |
| | | m/z | **Ph(H$_2$)DG** | **60 min** | **120 min** | **180 min** | **240 min** |
| (M-H) | $d_0$ | 200 | 100.0 | 100.0 | 50.8 | 34.8 | 15.0 |
| (M-H)+1 | $d_1$ | 201 | 0.0 | 83.5 | 92.4 | 94.8 | 92.5 |
| (M-H)+2 | $d_2$ | 202 | 0.0 | 35.5 | 43.9 | 77.8 | 85.6 |
| Total abundance | | | 100.0 | 219.0 | 187.1 | 207.4 | 193.0 |
| *Normalised relative abundances* | | | | | | | |
| | | m/z | **Ph(H$_2$)DG** | **60 min** | **120 min** | **180 min** | **240 min** |
| (M-H) | $d_0$ | 200 | 100.0 | 45.0 | 25.5 | 17.1 | 8.2 |
| (M-H)+1 | $d_1$ | 201 | 0.0 | 37.6 | 46.3 | 46.6 | 50.3 |
| (M-H)+2 | $d_2$ | 202 | 0.0 | 16.0 | 22.0 | 38.3 | 46.5 |
| *Level of deuterium incorporation* | | | | | | | |
| | | | **Ph(H$_2$)DG** | **60 min** | **120 min** | **180 min** | **240 min** |
| %D by LC-MS | | | 0 | 35 | 45 | 62 | 72 |
| %D by NMR | | | 0 | 33 | 48 | 59 | 66 |

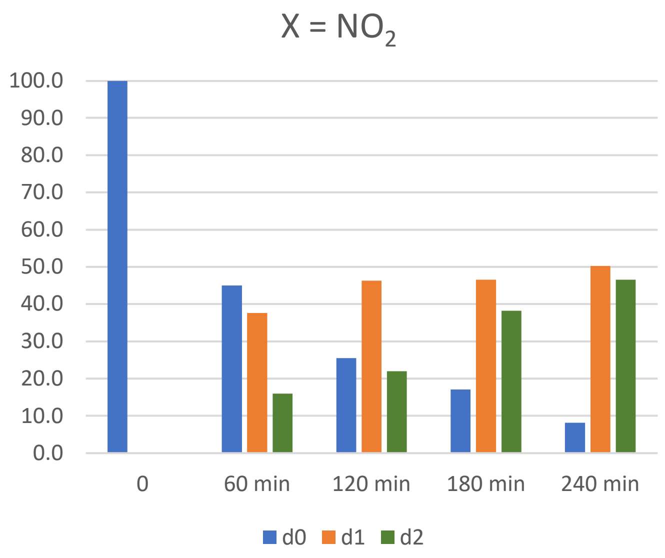


**Figure S10**. The distribution of $d_0$, $d_1$, and $d_2$ during 4-nitro-2-phenyl pyridine labelling as determined by LC-MS analysis**.**

**Table S11.** Rate monitoring for the deuteration of 4-methoxy-2-phenyl pyridine.

Following the General Kinetic Protocol using 92.6 mg of 4-methoxy-2-phenyl pyridine, 8.7 mg of Ir-catalyst.

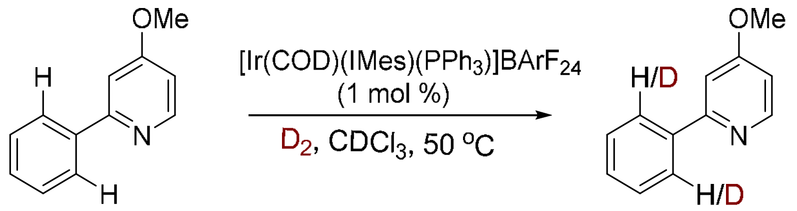


| Entry | Time, s | Integral (H/D) | [Substrate], M | ln [Substrate] |
|---|---|---|---|---|
| 0 | 0 | 2.00 | 0.200 | -1.61 |
| 1 | 600 | 1.88 | 0.188 | -1.67 |
| 2 | 1200 | 1.77 | 0.177 | -1.73 |
| 3 | 1800 | 1.70 | 0.170 | -1.77 |
| 4 | 3600 | 1.48 | 0.148 | -1.91 |
| 5 | 7200 | 1.12 | 0.112 | -2.19 |
| 6 | 10800 | 0.86 | 0.086 | -2.45 |
| 7 | 14400 | 0.67 | 0.067 | -2.70 |

**$k_{obs}$ = 7.96 × 10$^{-5}$ (s$^{-1}$)**

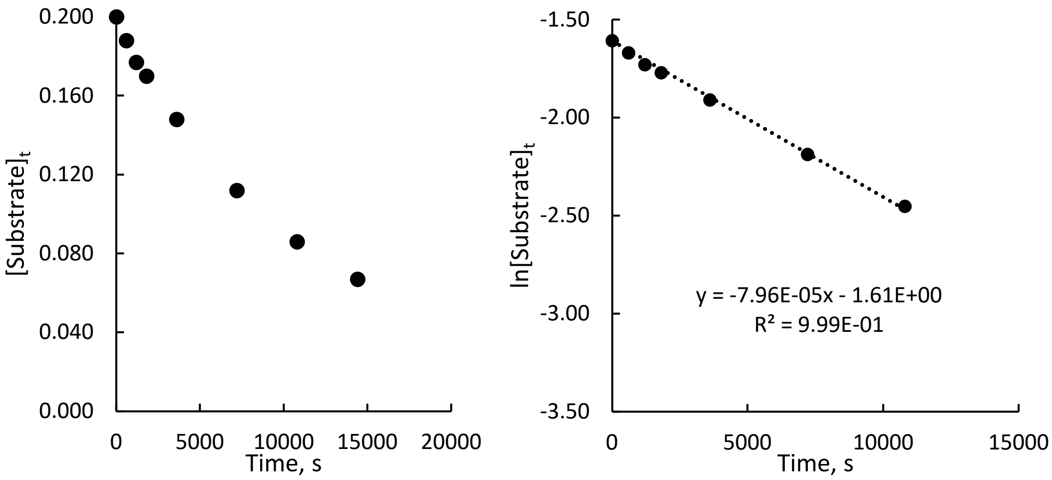


**Figure S11.** Kinetic profile for the HIE reaction of 4-methoxy-2-phenyl pyridine over time in $CDCl_3$ at 50 °C (left). Fitting a first-order kinetic model to the data (right).

**Table S12**. The extent of deuterium labelling and the distribution of $d_0$, $d_1$, and $d_2$ in 4-methoxy-2-phenyl pyridine as determined by LC-MS analysis (Data from experiment in Table S11).

| | | | | | | | | | |
|---|---|---|---|---|---|---|---|---|---|
| *Observed relative abundances* | | | | | | | | | |
| | | m/z | **Ph(H$_2$)DG** | **10 min** | **20 min** | **30 min** | **60 min** | **120 min** | **180 min** | **240 min** |
| (M+H) | $d_0$ | 186 | 100.0 | 100.0 | 100.0 | 100.0 | 100.0 | 100.0 | 55.0 | 27.2 |
| (M+H)+1 | $d_1$ | 187 | 14.1 | 17.0 | 18.2 | 20.2 | 27.5 | 63.8 | 75.9 | 68.3 |
| (M+H)+2 | $d_2$ | 188 | 1.2 | 5.9 | 10.3 | 16.3 | 31.7 | 90.7 | 100.0 | 100.0 |
| *Relative abundances adjusted for natural abundance of isotopes* | | | | | | | | | | |
| | | m/z | **Ph(H$_2$)DG** | **10 min** | **20 min** | **30 min** | **60 min** | **120 min** | **180 min** | **240 min** |
| (M+H) | $d_0$ | 186 | 100.0 | 100.0 | 100.0 | 100.0 | 100.0 | 100.0 | 55.0 | 27.2 |
| (M+H)+1 | $d_1$ | 187 | 0.0 | 2.9 | 4.1 | 6.1 | 13.4 | 49.7 | 68.1 | 64.5 |
| (M+H)+2 | $d_2$ | 188 | 0.0 | 2.3 | 6.6 | 12.3 | 26.7 | 80.6 | 88.7 | 90.1 |
| Total abundance | | | 100.0 | 105.2 | 110.7 | 118.4 | 140.1 | 230.3 | 211.9 | 181.8 |
| *Normalised relative abundances* | | | | | | | | | | |
| | | m/z | **Ph(H$_2$)DG** | **10 min** | **20 min** | **30 min** | **60 min** | **120 min** | **180 min** | **240 min** |
| (M+H) | $d_0$ | 186 | 100.0 | 95.0 | 90.4 | 84.5 | 71.4 | 43.4 | 26.0 | 15.0 |
| (M+H)+1 | $d_1$ | 187 | 0.0 | 2.8 | 3.7 | 5.2 | 9.6 | 21.6 | 32.2 | 35.5 |
| (M+H)+2 | $d_2$ | 188 | 0.0 | 2.2 | 5.9 | 10.4 | 19.0 | 35.0 | 41.9 | 49.6 |
| *Level of deuterium incorporation* | | | | | | | | | | |
| | | | **Ph(H$_2$)DG** | **10 min** | **20 min** | **30 min** | **60 min** | **120 min** | **180 min** | **240 min** |
| %D by LC-MS | | | 0 | 4 | 8 | 13 | 24 | 46 | 58 | 67 |
| %D by NMR | | | 0 | 6 | 12 | 15 | 26 | 44 | 57 | 67 |

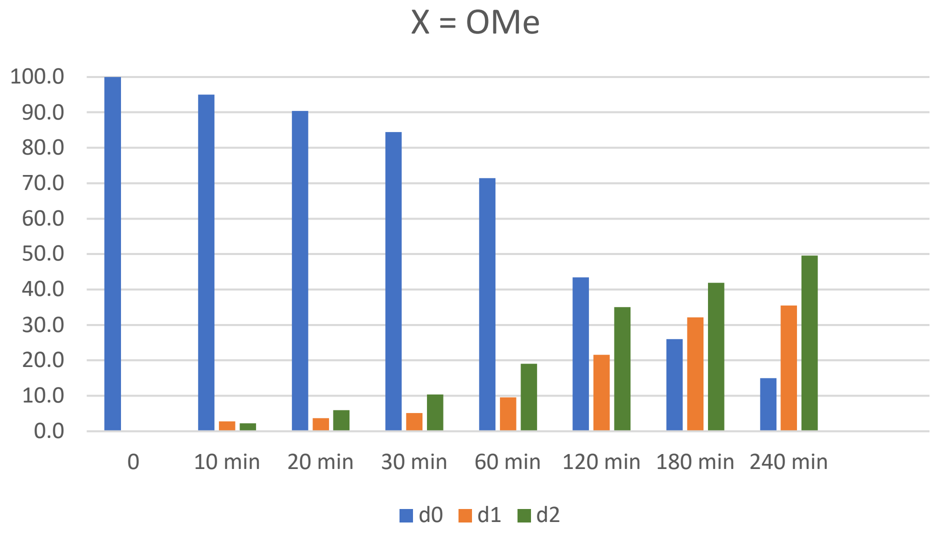


**Figure S12**. The distribution of $d_0$, $d_1$, and $d_2$ during 4-methoxy-2-phenyl pyridine labelling as determined by LC-MS analysis**.**

**Table S13.** Rate monitoring for the deuteration of 4-amino-2-phenyl pyridine.

Following the General Kinetic Protocol using 85.1 mg of 4-amino-2-phenyl pyridine, 8.7 mg of Ir-catalyst.

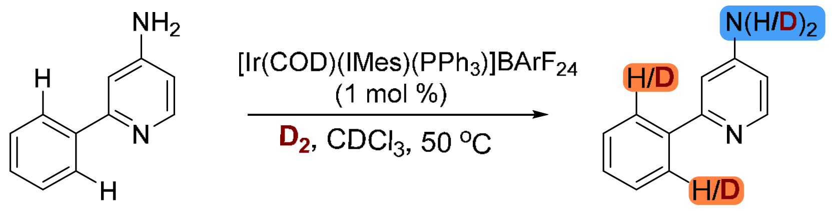


| Entry | Time, s | Integral (H/D, Ar) | [Substrate], M | ln [Substrate] |
|---|---|---|---|---|
| 0 | 0 | 2.00 | 0.200 | -1.61 |
| 1 | 600 | 1.94 | 0.194 | -1.64 |
| 2 | 1200 | 1.88 | 0.188 | -1.67 |
| 3 | 1800 | 1.81 | 0.181 | -1.71 |
| 4 | 3600 | 1.60 | 0.160 | -1.83 |
| 5 | 5400 | 1.35 | 0.135 | -2.00 |
| 6 | 7200 | 1.24 | 0.124 | -2.09 |
| 7 | 10800 | 1.09 | 0.109 | -2.22 |

$k_{obs}$ **= 6.77 × 10$^{-5}$ (s$^{-1}$)**

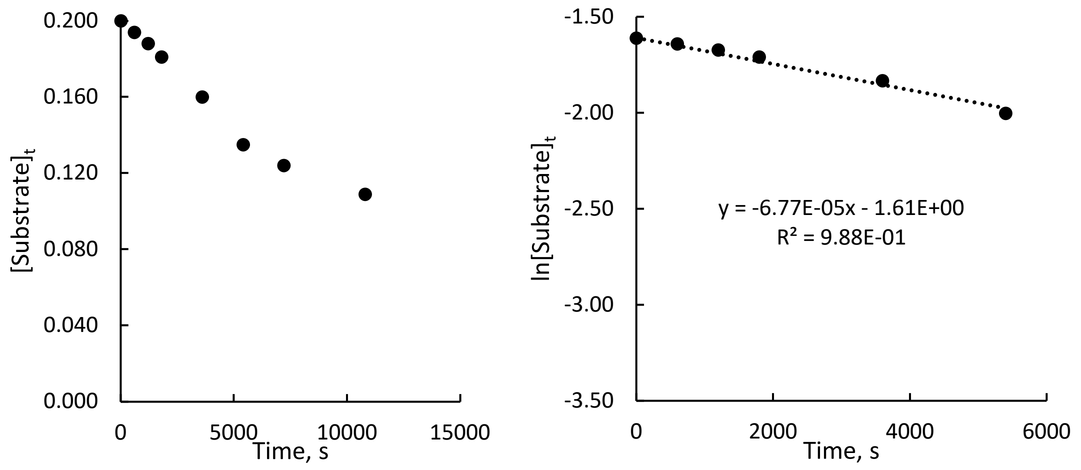


**Figure S13.** Kinetic profile for the HIE reaction of 4-amino-2-phenyl pyridine over time in $CDCl_3$ at 50 °C (left). Fitting a first-order kinetic model to the data (right).

**Table S14.** Deuterium incorporation in the HIE reaction of 4-amino-2-phenyl pyridine.

| Entry | Time, min | ortho-Aryl-H | | $NH_2$ group | |
|---|---|---|---|---|---|
| | | integral | %D | integral | %D |
| 0 | 0 | 2.00 | 0 | 2.00 | 0 |
| 1 | 10 | 1.94 | 3 | 1.54 | 23 |
| 2 | 20 | 1.88 | 6 | 1.41 | 30 |
| 3 | 30 | 1.81 | 10 | 1.30 | 35 |
| 4 | 60 | 1.60 | 20 | 1.16 | 42 |
| 5 | 90 | 1.35 | 33 | 1.06 | 47 |
| 6 | 120 | 1.24 | 38 | 0.96 | 52 |
| 7 | 180 | 1.09 | 46 | 0.87 | 57 |

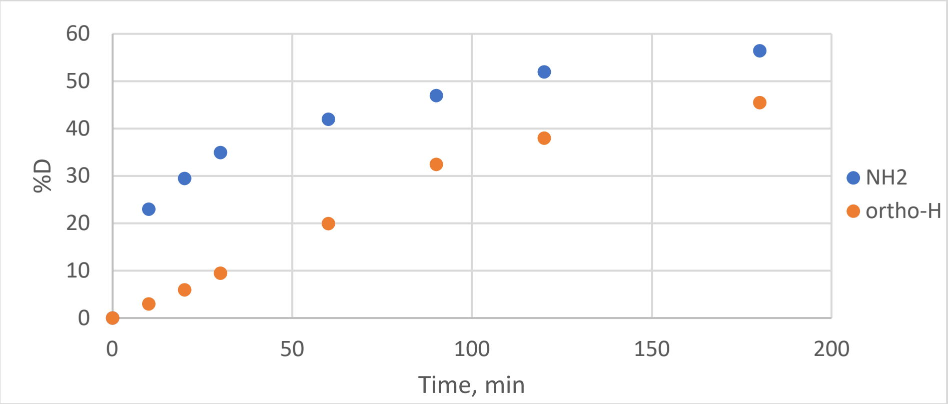


**Figure S14.** Rate study of the deuteration of 4-amino-2-phenyl-pyridine.

**Table S15.** Control experiments.

| | Conditions[a] | | Integral *ortho*-H | %D *ortho*-H | Integral $NH_2$ | %D $NH_2$ |
|---|---|---|---|---|---|---|
| **A** | Substrate + $D_2$ in $CDCl_3$ | NO catalyst | 2.00 | 0 | 2.00 | 0 |
| **B** | Substrate + Catalyst in $CDCl_3$ | NO $D_2$ | 2.00 | 0 | 2.00 | 0 |
| **C** | (Catalyst in $CDCl_3$) -> vac/$D_2$ -> freeze -> vac/Argon -> r.t. -> (Substrate in $CDCl_3$) | *Direct analysis* | 2.00 | 0 | 1.70 | 15 |
| | *$CDCl_3$ evaporated and fresh used for NMR* | | 2.00 | 0 | 1.91 | 5 |
| **D** | General HIE procedure | *Direct analysis* | 0.58 | 71 | 0.51 | 75 |
| | *$CDCl_3$ evaporated and fresh used for NMR* | | 0.57 | 72 | 0.97 | 52 |

[a] Following General HIE procedure using 10mg (0.06 mmol) of the substrate and 5.0 mg (0.003 mmol) of catalyst, unless noted otherwise

**Table S16.** Rate monitoring for the deuteration of 4-trifluoromethyl-2-phenyl pyridine.

Following the General Kinetic Protocol using 111.6 mg of 4-trifluoromethyl-2-phenyl pyridine, 8.7 mg of Ir-catalyst (run1).

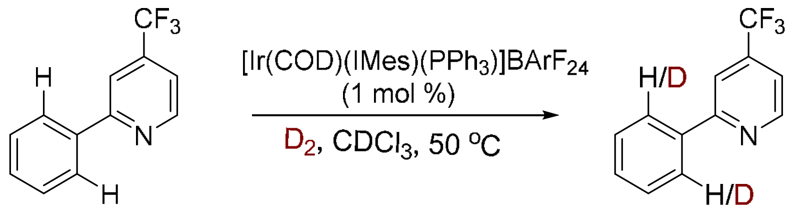


| Entry | Time, s | Integral (H/D) | [Substrate], M | ln [Substrate] |
|---|---|---|---|---|
| 0 | 0 | 2.00 | 0.200 | -1.61 |
| 1 | 600 | 1.97 | 0.197 | -1.62 |
| 2 | 1200 | 1.95 | 0.195 | -1.63 |
| 3 | 1800 | 1.93 | 0.193 | -1.65 |
| 4 | 3600 | 1.87 | 0.187 | -1.68 |
| 5 | 7200 | 1.78 | 0.178 | -1.73 |
| 6 | 10800 | 1.72 | 0.172 | -1.76 |

**$k_{obs}$ = 1.50 × $10^{-5}$ ($s^{-1}$)**

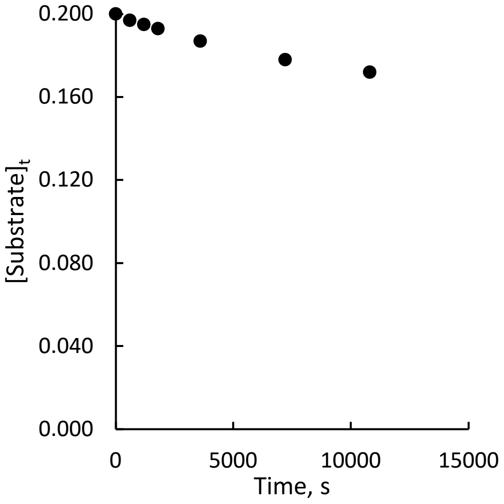


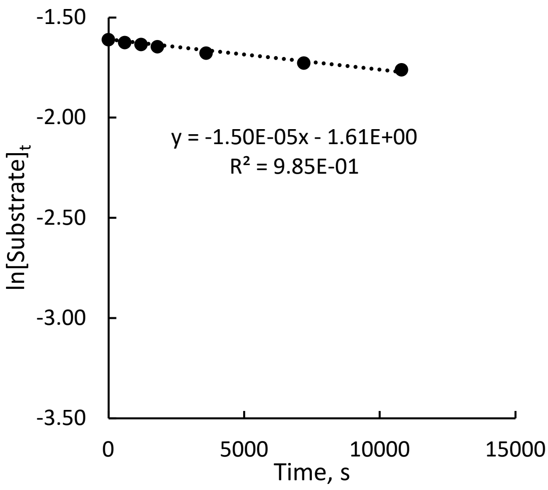


**Figure S15.** Kinetic profile for the HIE reaction of 4- trifluoromethyl-2-phenyl pyridine over time in $CDCl_3$ at 50 °C (left). Fitting a first-order kinetic model to the data (right).

**Table S17.** Rate monitoring for the deuteration of 4-trifluoromethyl-2-phenyl pyridine.

Following the General Kinetic Protocol using 111.6 mg of 4-trifluoromethyl-2-phenyl pyridine, 8.7 mg of Ir-catalyst (run 2).

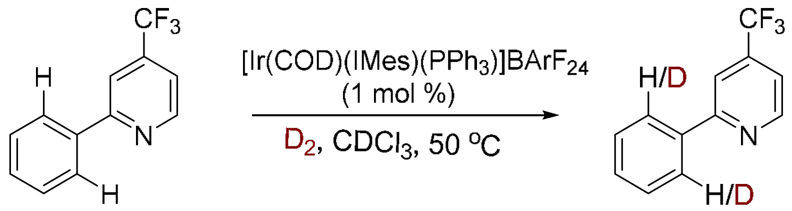


| Entry | Time, s | Integral (H/D) | [Substrate], M | ln [Substrate] |
|---|---|---|---|---|
| 0 | 0 | 2.00 | 0.200 | -1.61 |
| 1 | 600 | 1.97 | 0.197 | -1.62 |
| 2 | 1200 | 1.94 | 0.194 | -1.64 |
| 3 | 1800 | 1.91 | 0.191 | -1.66 |
| 4 | 3600 | 1.86 | 0.186 | -1.68 |
| 5 | 7200 | 1.76 | 0.176 | -1.74 |
| 6 | 10800 | 1.67 | 0.167 | -1.79 |
| 7 | 14400 | 1.58 | 0.158 | -1.85 |
| 8 | 18000 | 1.51 | 0.151 | -1.89 |

**$k_{obs}$ = 1.74 × 10$^{-5}$ (s$^{-1}$)**

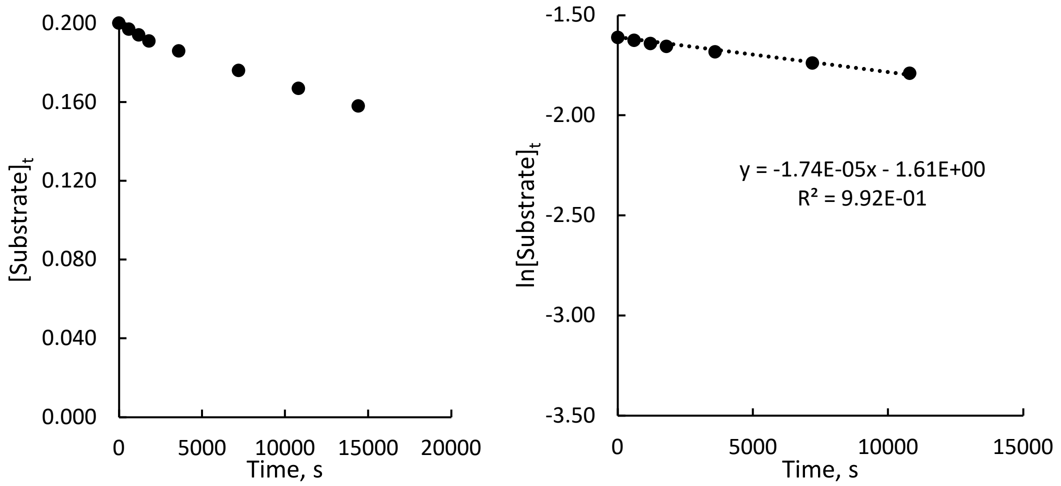


**Figure S16.** Kinetic profile for the HIE reaction of 4- trifluoromethyl-2-phenyl pyridine over time in $CDCl_3$ at 50 °C (left). Fitting a first-order kinetic model to the data (right).

**Table S18.** Rate monitoring for the deuteration of 4-trifluoromethyl-2-phenyl pyridine.

Following the General Kinetic Protocol using 111.6 mg of 4-trifluoromethyl-2-phenyl pyridine, 8.7 mg of Ir-catalyst (run 3).

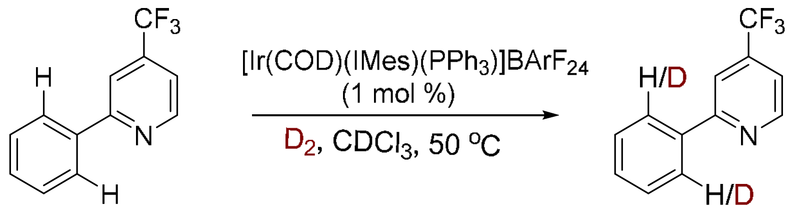


| Entry | Time, s | Integral (H/D) | [Substrate], M | ln [Substrate] |
|---|---|---|---|---|
| 0 | 0 | 2.00 | 0.200 | -1.61 |
| 1 | 600 | 1.99 | 0.199 | -1.61 |
| 2 | 1200 | 1.97 | 0.197 | -1.62 |
| 3 | 1800 | 1.94 | 0.194 | -1.64 |
| 4 | 3600 | 1.92 | 0.192 | -1.65 |
| 5 | 7200 | 1.81 | 0.181 | -1.71 |
| 6 | 10800 | 1.75 | 0.175 | -1.74 |
| 7 | 16200 | 1.63 | 0.163 | -1.81 |

**$k_{obs}$ = 1.26 × $10^{-5}$ ($s^{-1}$)**

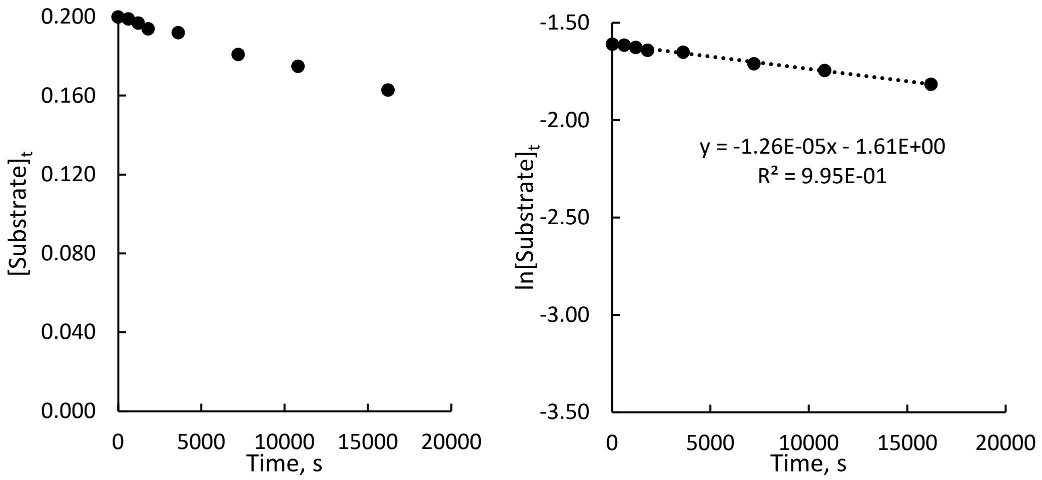


**Figure S17.** Kinetic profile for the HIE reaction of 4- trifluoromethyl-2-phenyl pyridine over time in $CDCl_3$ at 50 °C (left). Fitting a first-order kinetic model to the data (right).

**Table S19.** Rate monitoring for the deuteration of 4-trifluoromethyl-2-phenyl pyridine.

Following the General Kinetic Protocol using 111.6 mg of 4-trifluoromethyl-2-phenyl pyridine, 8.7 mg of Ir-catalyst (run 4).

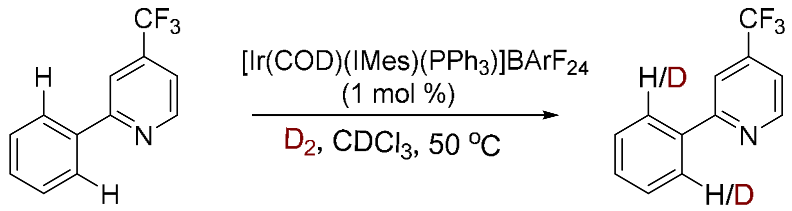


| Entry | Time, s | Integral (H/D) | [Substrate], M | ln [Substrate] |
|---|---|---|---|---|
| 0 | 0 | 2.00 | 0.200 | -1.61 |
| 1 | 600 | 1.95 | 0.195 | -1.63 |
| 2 | 1200 | 1.93 | 0.193 | -1.65 |
| 3 | 1800 | 1.90 | 0.190 | -1.66 |
| 4 | 3600 | 1.79 | 0.179 | -1.72 |
| 5 | 7200 | 1.54 | 0.154 | -1.87 |
| 6 | 10800 | 1.36 | 0.136 | -2.00 |
| 7 | 14400 | 1.22 | 0.122 | -2.10 |

**$k_{obs}$ = 3.48 × 10$^{-5}$ (s$^{-1}$)**

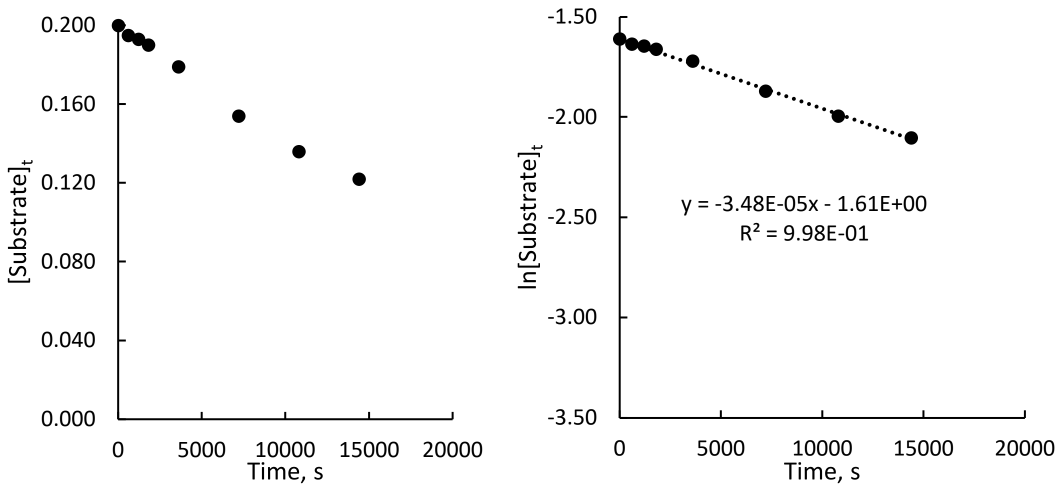


**Figure S18.** Kinetic profile for the HIE reaction of 4- trifluoromethyl-2-phenyl pyridine over time in $CDCl_3$ at 50 °C (left). Fitting a first-order kinetic model to the data (right).

**Table S20.** Rate monitoring for the deuteration of 4-trifluoromethyl-2-phenyl pyridine.

Following the General Kinetic Protocol using 85.0 mg (0.38 mmol) of 4-trifluoromethyl-2-phenyl pyridine, 6.9 mg (0.004 mmol) of Ir-catalyst and 2.0 mL of $CDCl_3$ (run 1).

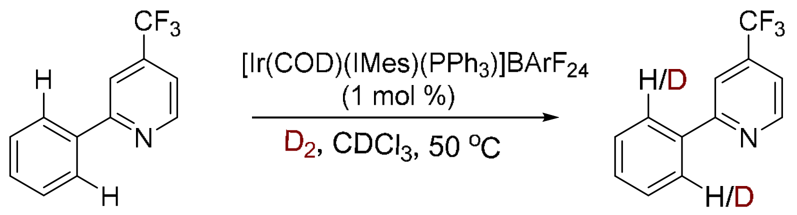


| Entry | Time, s | Integral (H/D) | [Substrate], M | ln [Substrate] |
|---|---|---|---|---|
| 0 | 0 | 2.00 | 0.200 | -1.61 |
| 1 | 600 | 1.87 | 0.187 | -1.68 |
| 2 | 1200 | 1.73 | 0.173 | -1.75 |
| 3 | 1800 | 1.61 | 0.161 | -1.83 |
| 4 | 3600 | 1.38 | 0.138 | -1.98 |
| 5 | 7200 | 1.15 | 0.115 | -2.16 |
| 6 | 10800 | 1.04 | 0.104 | -2.26 |

$\boldsymbol{k_{obs} = 1.08 \times 10^{-4}\ (s^{-1})}$

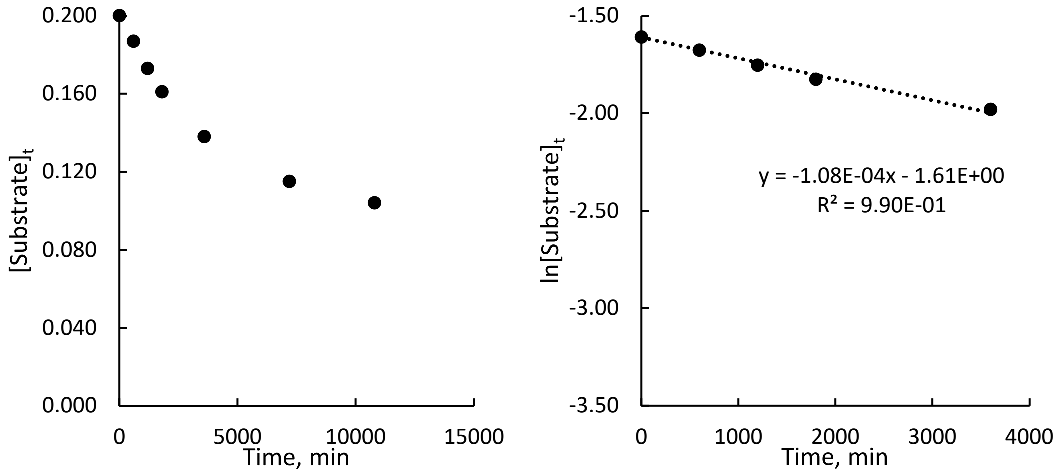


**Figure S19.** Kinetic profile for the HIE reaction of 4- trifluoromethyl-2-phenyl pyridine over time in $CDCl_3$ at 50 °C (left). Fitting a first-order kinetic model to the data (right).

**Table S21.** Rate monitoring for the deuteration of 4-trifluoromethyl-2-phenyl pyridine.

Following the General Kinetic Protocol using 85.0 mg (0.38 mmol) of 4-trifluoromethyl-2-phenyl pyridine, 6.9 mg (0.004 mmol) of Ir-catalyst and 2.0 mL of $CDCl_3$ (run 2).

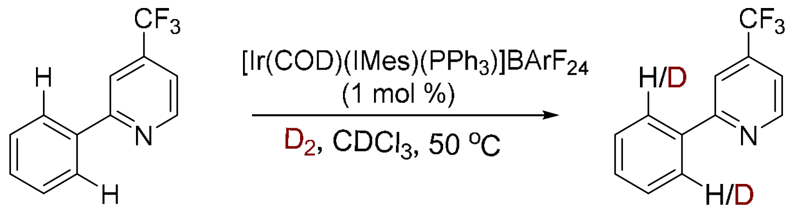


| Entry | Time, s | Integral (H/D) | [Substrate], M | ln [Substrate] |
|---|---|---|---|---|
| 0 | 0 | 2.00 | 0.200 | -1.61 |
| 1 | 600 | 1.89 | 0.189 | -1.67 |
| 2 | 1200 | 1.76 | 0.176 | -1.74 |
| 3 | 1800 | 1.65 | 0.165 | -1.80 |
| 4 | 3600 | 1.34 | 0.134 | -2.01 |
| 5 | 7800 | 0.99 | 0.099 | -2.31 |
| 6 | 10800 | 0.83 | 0.083 | -2.49 |
| 7 | 14400 | 0.75 | 0.075 | -2.59 |

**$k_{obs}$ = 1.10 × 10$^{-4}$ (s$^{-1}$)**

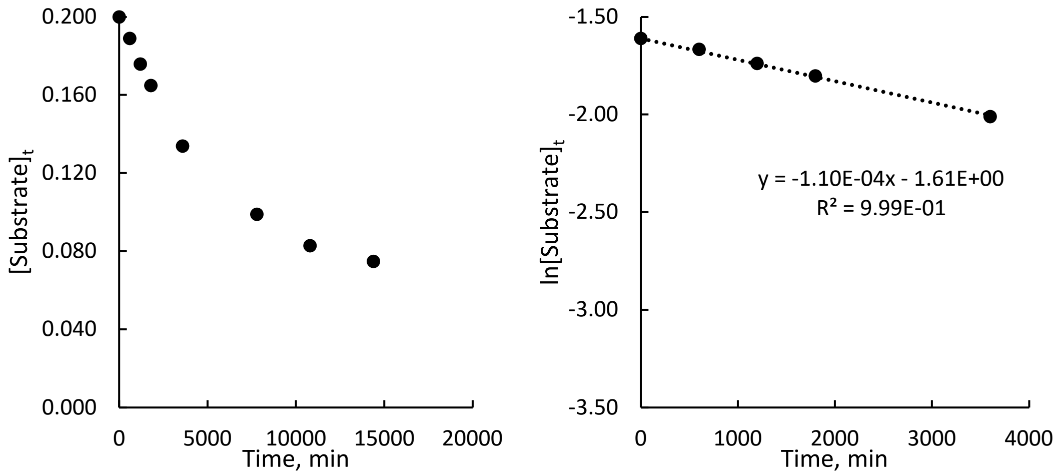


**Figure S20.** Kinetic profile for the HIE reaction of 4- trifluoromethyl-2-phenyl pyridine over time in $CDCl_3$ at 50 °C (left). Fitting a first-order kinetic model to the data (right).

**Table S22.** Rate monitoring for the deuteration of 4-trifluoromethyl-2-phenyl pyridine.

Following the General Kinetic Protocol using 98.2 mg (0.44 mmol) of 4-trifluoromethyl-2-phenyl pyridine, 7.6 mg (0.004 mmol) of Ir-catalyst and 2.2 mL of $CDCl_3$ (run 1).

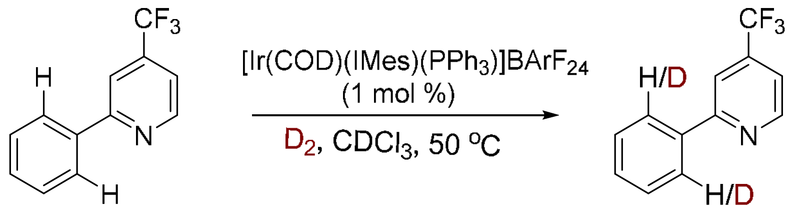


| Entry | Time, s | Integral (H/D) | [Substrate], M | ln [Substrate] |
|---|---|---|---|---|
| 0 | 0 | 2.00 | 0.200 | -1.61 |
| 1 | 600 | 2.00 | 0.200 | -1.61 |
| 2 | 1200 | 2.00 | 0.200 | -1.61 |
| 3 | 1800 | 1.98 | 0.198 | -1.62 |
| 4 | 3600 | 1.90 | 0.190 | -1.66 |
| 5 | 5400 | 1.84 | 0.184 | -1.69 |
| 6 | 9000 | 1.70 | 0.170 | -1.77 |
| 7 | 12600 | 1.55 | 0.155 | -1.86 |
| $k_{obs}$ = 1.85 × 10$^{-5}$ (s$^{-1}$) | | | | |

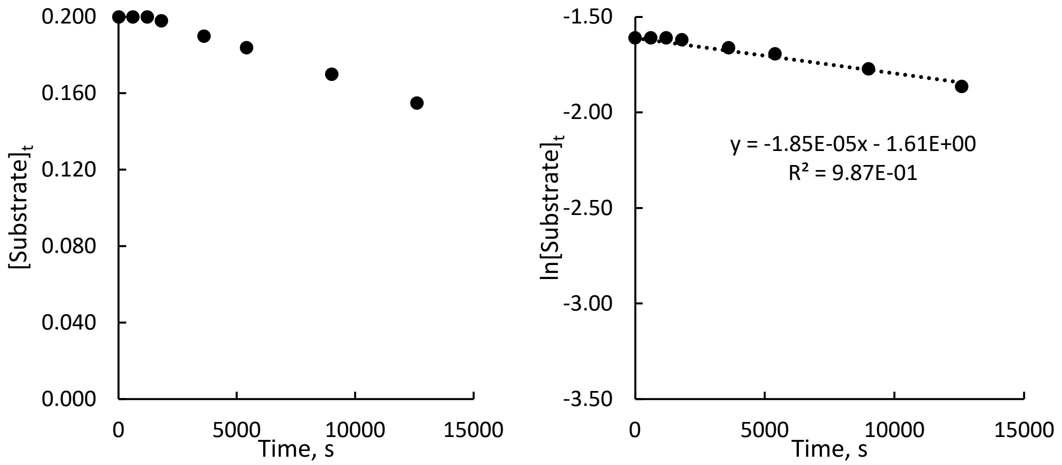


**Figure S21.** Kinetic profile for the HIE reaction of 4- trifluoromethyl-2-phenyl pyridine over time in $CDCl_3$ at 50 °C (left). Fitting a first-order kinetic model to the data (right).

**Table S23.** Rate monitoring for the deuteration of 4-trifluoromethyl-2-phenyl pyridine.

Following the General Kinetic Protocol using 111.6 mg of 4-trifluoromethyl-2-phenyl pyridine, 8.7 mg of Ir-catalyst (catalyst was preactivated with $D_2$ before substrate addition).

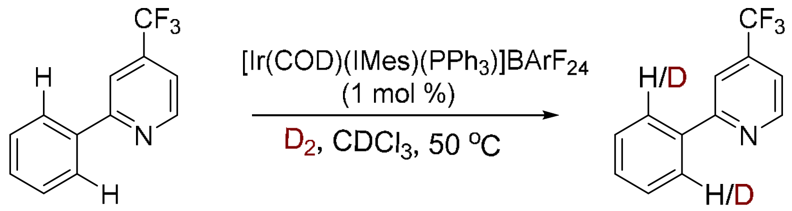


| Entry | Time, s | Integral (H/D) | [Substrate], M | ln [Substrate] |
|---|---|---|---|---|
| 0 | 0 | 2.00 | 0.200 | -1.61 |
| 1 | 600 | 1.89 | 0.189 | -1.67 |
| 2 | 1200 | 1.82 | 0.182 | -1.70 |
| 3 | 1800 | 1.75 | 0.175 | -1.74 |
| 4 | 2400 | 1.66 | 0.166 | -1.80 |
| 5 | 3000 | 1.60 | 0.160 | -1.83 |
| 6 | 3600 | 1.54 | 0.154 | -1.87 |
| 7 | 5400 | 1.42 | 0.142 | -1.95 |
| 8 | 9000 | 1.30 | 0.130 | -2.04 |
| 9 | 12600 | 1.27 | 0.127 | -2.06 |
| 10 | 15600 | 1.24 | 0.124 | -2.09 |

**$k_{obs}$ = 7.44 × $10^{-5}$ ($s^{-1}$)**

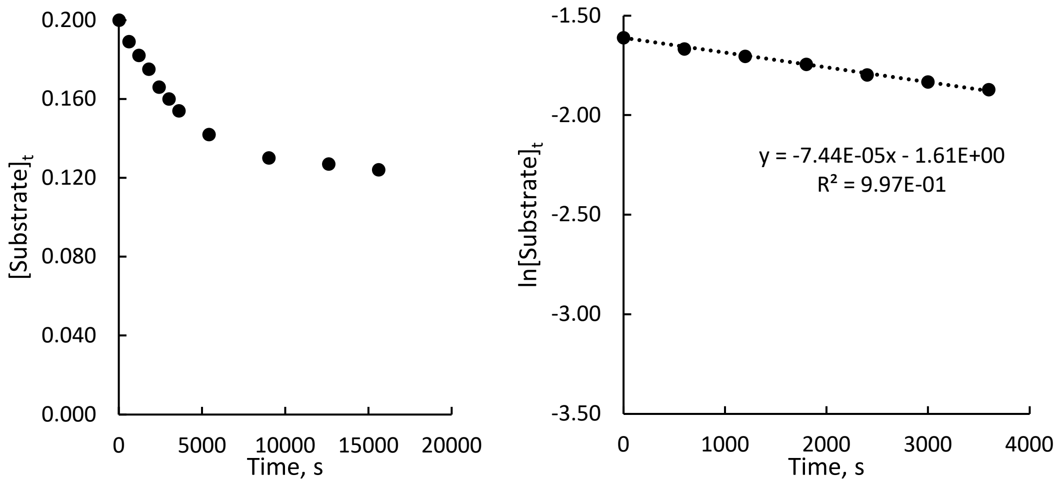


**Figure S22.** Kinetic profile for the HIE reaction of 4- trifluoromethyl-2-phenyl pyridine over time in $CDCl_3$ at 50 °C (left). Fitting a first-order kinetic model to the data (right).

**Table S24**. The extent of deuterium labelling and the distribution of $d_0$, $d_1$, and $d_2$ in 4-trifluoromethyl-2-phenyl pyridine as determined by LC-MS analysis (Data from experiment in Table S20).

| *Observed relative abundances* | | | | | | | |
|---|---|---|---|---|---|---|---|
| | | m/z | **Ph($H_2$)DG** | **60 min** | **120 min** | **180 min** | **240 min** |
| (M-H) | $d_0$ | 223 | 100.0 | 100.0 | 100.0 | 71.8 | 56.0 |
| (M-H)+1 | $d_1$ | 224 | 13.3 | 34.6 | 63.5 | 62.0 | 51.6 |
| (M-H)+2 | $d_2$ | 225 | 1.4 | 38.4 | 91.1 | 100.0 | 100.0 |
| *Relative abundances adjusted for natural abundance of isotopes* | | | | | | | |
| | | m/z | **Ph($H_2$)DG** | **60 min** | **120 min** | **180 min** | **240 min** |
| (M-H) | $d_0$ | 223 | 100.0 | 100.0 | 100.0 | 71.8 | 56.0 |
| (M-H)+1 | $d_1$ | 224 | 0.0 | 23.6 | 50.2 | 52.4 | 44.1 |
| (M-H)+2 | $d_2$ | 225 | 0.0 | 32.6 | 81.6 | 91.0 | 92.5 |
| Total abundance | | | 100.0 | 219.0 | 187.1 | 207.4 | 193.0 |
| *Normalised relative abundances* | | | | | | | |
| | | m/z | **Ph($H_2$)DG** | **60 min** | **120 min** | **180 min** | **240 min** |
| (M-H) | $d_0$ | 223 | 100.0 | 64.0 | 43.2 | 33.4 | 29.1 |
| (M-H)+1 | $d_1$ | 224 | 0.0 | 15.1 | 21.7 | 24.4 | 22.9 |
| (M-H)+2 | $d_2$ | 225 | 0.0 | 20.9 | 35.2 | 42.3 | 48.0 |
| *Level of deuterium incorporation* | | | | | | | |
| | | | **Ph($H_2$)DG** | **60 min** | **120 min** | **180 min** | **240 min** |
| %D by LC-MS | | | 0 | 28 | 46 | 54 | 59 |
| %D by NMR | | | 0 | 33 | 51 | 59 | 63 |

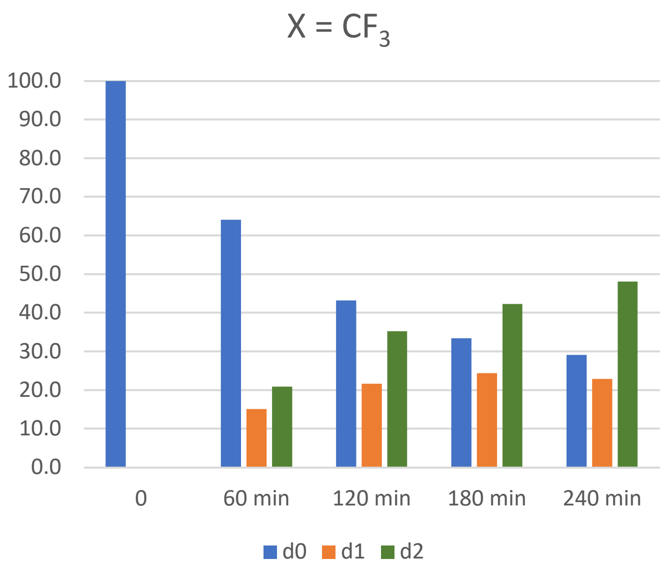


**Figure S23**. The distribution of $d_0$, $d_1$, and $d_2$ during 4-trifluoromethyl-2-phenyl pyridine labelling as determined by LC-MS analysis**.**

## 3.4. Hammett plot for substituted phenylpyridines

**Table S25.** Rate constants for deuteration of 4-substituted 2-Phenylpyridines and Hammett parameters[13]

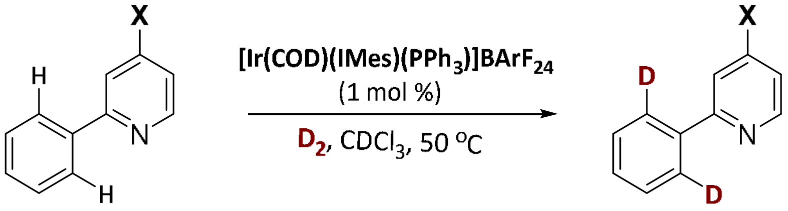


| X | $k_{obs}$ ($s^{-1}$) | log ($k_X/k_H$) | $s_p$ | $s_p^-$ | $s_p^+$ |
|---|---|---|---|---|---|
| **H**[a,b] | $9.90 \cdot 10^{-5}$ | 1.00 | 0.00 | 0.00 | 0.00 |
| **OMe** | $7.96 \cdot 10^{-4}$ | 0.80 | -0.09 | -0.27 | -0.26 |
| **$NO_2$** | $1.44 \cdot 10^{-4}$ | 1.45 | 0.16 | 0.78 | 1.27 |
| **$NH_2$** | $6.77 \cdot 10^{-4}$ | 0.68 | -0.17 | -0.66 | -0.15 |
| **$CF_3$ (0.38 mmol)**[c] | $1.09 \cdot 10^{-4}$ | 1.10 | 0.04 | 0.54 | 0.65 |
| **$CF_3$ (0.50 mmol)**[d] | $2.00 \cdot 10^{-4}$ | | | | |
| **$CF_3$ (0.44 mmol)** | $1.85 \cdot 10^{-4}$ | | | | |

[a] For X=H $k_{obs}$ from ref.[14]; [b] Using 0.50 mmol of substrate; [c] Average of 2 runs; [d] Average of 4 runs;

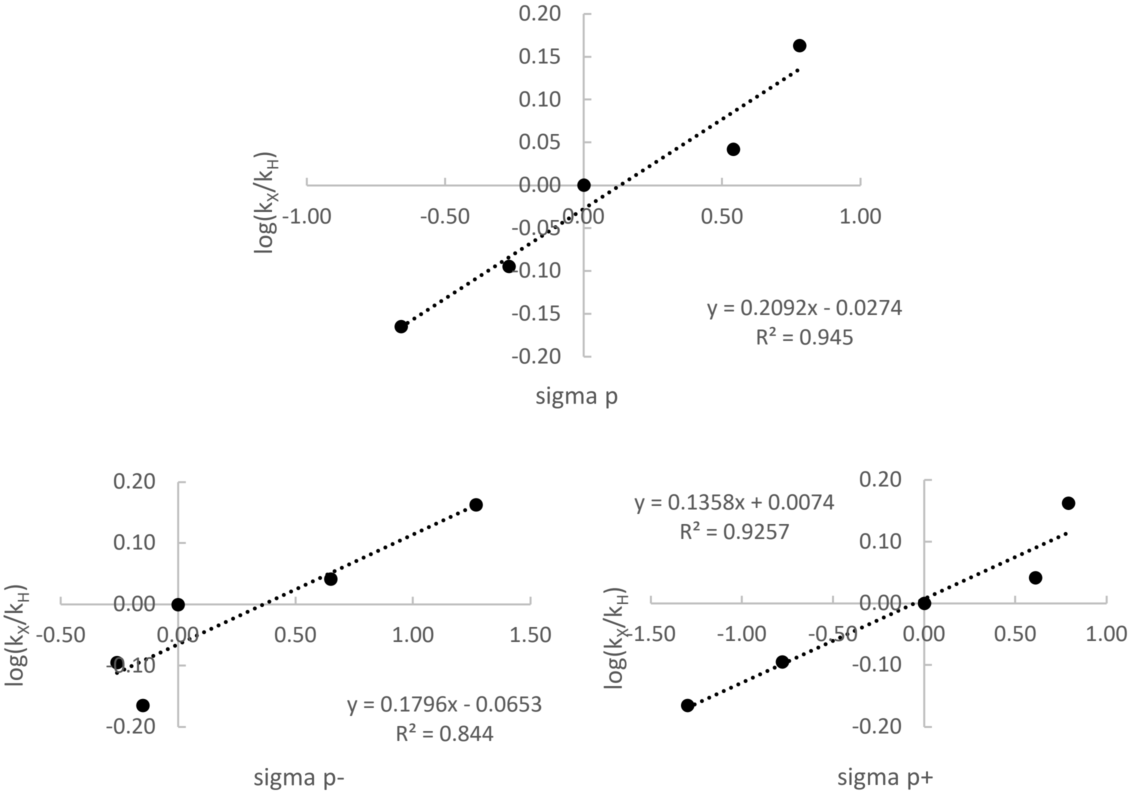


**Figure S24.** Hammett plots.

# 4. Computational chemistry

## 4.1. General computational details

Conformational sampling was conducted using CREST 2.12. DFT calculations were carried out using Gaussian16 Rev C.01.

Geometry optimisations were carried out at 298.15 K without symmetry constraints using the M06-L density functional,[15] the SDD basis set and pseudopotential on iridium,[16] and the 6-311G(d,p) basis set on all other atoms (H, C, N, O, P, Cl). Tight convergence criteria were used for all geometry optimisations. The nature of each stationary point was confirmed using a frequency calculation at 298.15 K. Corrections for temperature (323.15 K), concentration (1 M), and quasiharmonic entropy and enthalpy corrections were applied using GoodVibes.[17]

Energies for each species were further refined using single point calculations with the M06-L density functional, the def2-QZVP basis set and – where relevant – associated pseudopotential,[18,19] and solvation in dichloromethane with the SMD model.[20] Free energies quoted in the manuscript are the sum of electronic energy with the larger basis set and the (corrected) correction to free energy obtained with the smaller basis set.

## 4.2. Energies of optimised structures

**Table S26.** Energies of optimised structures from DFT calculations: data from optimisation/frequency calculations using a small basis set (6-311G(d,p) + SDD) and data from single point calculations using a large basis set (Def2-QZVP).

| Structure | Small Basis: no solvation model | | | Small Basis: E (Ha) | Large Basis: E (Ha) | |
|---|---|---|---|---|---|---|
| | E (Ha) | $G_{corr}$ (Ha)[a] | $G'_{corr}$ (Ha)[b] | SMD($CHCl_3$) | SMD(DCM) | SMD($CHCl_3$) |
| | | | | | | |
| *Active Catalyst generation* | | | | | | |
| **I-$CHCl_3$** | -4904.837931 | 0.638262 | 0.631475 | -4904.916622 | N/A | -4905.370873 |
| **I-DCM** | -3985.660675 | 0.660029 | 0.653693 | N/A | -3986.163813 | N/A |
| **$CHCl_3$** | -1419.306870 | -0.009336 | -0.008939 | -1419.305408 | N/A | -1419.375981 |
| **DCM** | -959.707643 | 0.002525 | 0.003224 | N/A | -959.704419 | N/A |
| **II-$CHCl_3$** | -3485.518623 | 0.622184 | 0.617418 | -3485.605779 | N/A | -3485.996414 |
| **II-DCM** | -3025.927873 | 0.633406 | 0.628435 | N/A | -3025.299438 | N/A |
| **$H_2$** | -1.171616 | -0.001598 | 0.000504 | -1.171312 | -1.171112 | -1.173323 |
| **III-$CHCl_3$** | -3486.710663 | 0.641220 | 0.634990 | -3486.789501 | N/A | -3487.182681 |
| **III-DCM** | -3027.118414 | 0.650688 | 0.645547 | N/A | -3026.472867 | N/A |
| | | | | | | |
| *Acetophenone* | | | | | | |
| **Acetophenone** | -384.939717 | 0.104947 | 0.104647 | -384.951760 | -385.025002 | -385.023803 |
| **IV-$CHCl_3$** | -3870.502685 | 0.754793 | 0.747089 | -3870.584320 | N/A | -3871.04355 |
| **IV-DCM** | -3410.911774 | 0.764932 | 0.757646 | N/A | -3411.439640 | N/A |
| **V** | -2836.161405 | 0.870853 | 0.863805 | -2836.244017 | -2836.714919 | -2836.709156 |
| **VI** | -2451.179789 | 0.740988 | 0.735025 | -2451.264950 | -2451.669379 | -2451.662431 |
| **TS1** | -2451.145441 | 0.737744 | 0.731734 | -2451.223700 | -2451.628984 | -2451.622734 |
| **VII** | -2451.173723 | 0.740832 | 0.734768 | -2451.252804 | -2451.656716 | -2451.650798 |
| **TS2** | -2451.159147 | 0.739570 | 0.733538 | -2451.250922 | -2451.657880 | -2451.648661 |
| | | | | | | |
| *Nitrobenzene* | | | | | | |
| **Nitrobenzene** | -436.813122 | 0.110568 | 0.070874 | -436.823160 | -436.907044 | -436.905656 |
| **IV-$CHCl_3$** | -3922.372227 | 0.717204 | 0.724324 | -3922.452587 | N/A | -3922.919509 |
| **IV-DCM** | -3462.781303 | 0.883248 | 0.724324 | N/A | -3463.317405 | N/A |
| **V** | -2939.897824 | 0.959539 | 0.792126 | -2939.979336 | -2940.464924 | -2940.45903 |
| **VI** | -2503.043798 | 0.846611 | 0.700180 | -2503.128477 | -2503.541018 | -2503.533724 |
| **TS1** | -2503.013753 | 0.842008 | 0.697512 | -2503.092894 | -2503.506493 | -2503.49985 |
| **VII** | -2503.044108 | 0.844631 | 0.699954 | -2503.124129 | -2503.536198 | -2503.529955 |
| **TS2** | -2503.025713 | 0.845584 | 0.709933 | -2503.118009 | -2503.533784 | -2503.524105 |
| **V'** | -2503.055534 | 0.847022 | 0.709798 | -2503.136545 | -2503.549155 | -2503.542727 |
| | | | | | | |
| *Methyl benzoate* | | | | | | |
| **Methyl benzoate** | -460.184852 | 0.152843 | 0.108205 | -460.194731 | -460.282036 | -460.280917 |
| **IV-$CHCl_3$** | -3945.746696 | 0.75783 | 0.762105 | -3945.826133 | N/A | -3946.299122 |
| **IV-DCM** | -3486.155314 | 0.925432 | 0.762105 | N/A | -3486.696089 | N/A |
| **V** | -2986.638582 | 1.045223 | 0.872549 | -2986.718065 | -2987.215751 | -2987.209570 |
| **VI** | -2526.421835 | 0.888825 | 0.736879 | -2526.507063 | -2526.926889 | -2526.918772 |
| **TS1** | -2526.384460 | 0.884443 | 0.735321 | -2526.461515 | -2526.880582 | -2526.874438 |
| **VII** | -2526.412046 | 0.887039 | 0.737749 | -2526.490988 | -2526.909634 | -2526.903533 |
| **TS2** | -2526.404374 | 0.888197 | 0.750658 | -2526.489095 | -2526.907817 | -2526.901682 |
| **V'** | -2526.409416 | 0.888804 | 0.736824 | -2526.497802 | -2526.918160 | -2526.909817 |
| | | | | | | |
| *Phenylpyridine* | | | | | | |
| **2-Phenylpyridine** | -479.391280 | 0.178900 | 0.133908 | -479.4061194 | -479.495615 | -479.494499 |
| **IV-$CHCl_3$** | -3964.955314 | 0.785897 | 0.789878 | -3965.038861 | N/A | -3965.514215 |
| **IV-DCM** | -3505.362610 | 0.952322 | 0.789878 | N/A | -3505.909597 | N/A |
| **V** | -3025.049060 | 1.097946 | 0.927240 | -3025.138943 | -3025.642608 | -3025.635146 |
| **VI** | -2545.643428 | 0.915604 | 0.764257 | -2545.728144 | -2546.149421 | -2546.141998 |

| | | | | | | |
|---|---|---|---|---|---|---|
| **TS1** | -2545.610620 | 0.910923 | 0.763194 | -2545.690872 | -2546.112866 | -2546.106367 |
| **VII** | -2545.635606 | 0.913481 | 0.765257 | -2545.717235 | -2546.138818 | -2546.132201 |
| **TS2** | -2545.620256 | 0.914771 | 0.776365 | -2545.715007 | -2546.139086 | -2546.129237 |
| | | | | | | |
| *2-Methyl-1-phenylimidazole* | | | | | | |
| **2-Methyl-1-phenylimidazole** | -496.6393613 | 0.190362 | 0.143768 | -496.6577431 | -496.752600 | -496.750617 |
| **IV-CHCl$_3$** | -3982.213578 | 0.794494 | 0.801045 | -3982.296983 | N/A | -3982.776283 |
| **IV-DCM** | -3522.627987 | 0.963817 | 0.801045 | N/A | -3523.177730 | N/A |
| **V** | -3059.566448 | 1.121094 | 0.948302 | -3059.653896 | -3060.166837 | -3060.158972 |
| **VI** | -2562.897739 | 0.927107 | 0.773557 | -2562.984488 | -2563.410878 | -2563.402672 |
| **TS1** | -2562.861354 | 0.922444 | 0.773032 | -2562.941815 | -2563.368482 | -2563.361587 |
| **VII** | -2562.885226 | 0.925084 | 0.773813 | -2562.966773 | -2563.392980 | -2563.386205 |
| **TS2** | -2562.884941 | 0.925735 | 0.787434 | -2562.974647 | -2563.401427 | -2563.392619 |
| | | | | | | |
| *2-Phenylpyrimidine* | | | | | | |
| **2-Phenylpyrimidine** | -495.437700 | 0.166935 | 0.122193 | -495.4529781 | -495.544470 | -495.5432632 |
| **IV-CHCl$_3$** | -3980.997762 | 0.772933 | 0.778618 | -3981.082321 | N/A | -3981.559451 |
| **IV-DCM** | -3521.408710 | 0.940058 | 0.778618 | N/A | -3521.956955 | N/A |
| **V** | -3057.135911 | 1.073641 | 0.903153 | -3057.227046 | -3057.735213 | -3057.727359 |
| **VI** | -2561.686994 | 0.903703 | 0.755092 | -2561.771724 | -2562.194807 | -2562.187191 |
| **TS1** | -2561.652048 | 0.898926 | 0.750075 | -2561.732636 | -2562.156491 | -2562.150112 |
| **VII** | -2561.677985 | 0.901402 | 0.753220 | -2561.760795 | -2562.184577 | -2562.177582 |
| **TS2** | -2561.666008 | 0.902537 | 0.764799 | -2561.758989 | -2562.184516 | -2562.175182 |
| | | | | | | |
| *1-Phenylpyrazole* | | | | | | |
| **1-Phenylpyrazole** | -457.302839 | 0.160376 | 0.117215 | -457.316402 | -457.403509 | -457.402148 |
| **IV-CHCl$_3$** | -3942.869647 | 0.770906 | 0.773915 | -3942.951713 | N/A | -3943.424039 |
| **IV-DCM** | -3483.279528 | 0.933507 | 0.773915 | N/A | -3483.821512 | N/A |
| **V** | -2980.879903 | 1.061035 | 0.894100 | -2980.965715 | -2981.463099 | -2981.455798 |
| **VI** | -2523.550831 | 0.896875 | 0.747240 | -2523.637025 | -2524.055874 | -2524.048000 |
| **TS1** | -2523.517657 | 0.892340 | 0.746302 | -2523.598168 | -2524.017495 | -2524.010835 |
| **VII** | -2523.544242 | 0.895024 | 0.749062 | -2523.625711 | -2524.044075 | -2524.037382 |
| | | | | | | |
| *2-Phenyloxazoline* | | | | | | |
| **2-Phenyloxazoline** | -478.3958261 | 0.172188 | 0.126848 | -478.408933 | -478.499669 | -478.498594 |
| **IV-CHCl$_3$** | -3963.968053 | 0.780856 | 0.784423 | -3964.050133 | N/A | -3964.526440 |
| **IV-DCM** | -3504.378855 | 0.945505 | 0.784423 | N/A | -3504.923140 | N/A |
| **V** | -3023.073915 | 1.085233 | 0.915155 | -3023.158804 | -3023.664094 | -3023.656852 |
| **VI** | -2544.650349 | 0.908962 | 0.759293 | -2544.735137 | -2545.157544 | -2545.149724 |
| **TS1** | -2544.610792 | 0.904482 | 0.757605 | -2544.689647 | -2545.111506 | -2545.105231 |
| **VII** | -2544.637886 | 0.907103 | 0.759467 | -2544.717542 | -2545.139605 | -2545.133019 |
| **TS2** | -2544.623980 | 0.907963 | 0.772689 | -2544.713283 | -2545.137226 | -2545.128486 |
| | | | | | | |
| *For EDA* | | | | | | |
| **VIII** | -2066.185739 | 0.607716 | 0.604321 | N/A | N/A | -2066.609377 |
| | | | | | | |

a) Uncorrected correction to free energy. b) Correction to free energy after adjusting for temperature (323.15 K), concentration (1 M), and quasiharmonic corrections to entropy and enthalpy.

## 4.3. Energy decomposition analysis

| Structure | M06-L/Def2-QZVP (no solvent model) | | |
|---|---|---|---|
| | E (Ha) | $E_{dist}$ (kcal/mol) | $E_{int}$ (kcal/mol) |
| **[Ir(H)$_2$(IMes)(PPh$_3$)] (VIII)** | -2066.518138 | | |
| **Acetophenone** | -385.011842 | | |
| **2-Phenylpyridine** | -479.480164 | | |
| | | | |
| *[Ir(H)$_2$(PhAc)(IMes)(PPh$_3$)* (**VI**) | | | |
| **[Ir(H)$_2$(IMes)(PPh$_3$)] fragment** | -2066.502547 | 9.8 | -- |
| **PhAc fragment** | -385.007393 | 2.8 | -- |
| **[Ir(H)$_2$(PhAc)(IMes)(PPh$_3$)]** | -2451.578846 | -- | -43.2 |
| ***Total*** | | 12.6 | -43.2 |
| | | | |
| *[Ir(H)$_2$(PhPy)(IMes)(PPh$_3$)* (**VI**) | | | |
| **[Ir(H)$_2$(IMes)(PPh$_3$)] fragment** | -2066.499926 | 11.4 | -- |
| **PhPy fragment** | -479.475577 | 2.9 | -- |
| **[Ir(H)$_2$(PhPy)(IMes)(PPh$_3$)]** | -2546.058870 | -- | -52.3 |
| ***Total*** | | 14.3 | -52.3 |
| | | | |
| *[Ir(H)$_2$(PhAc)$_2$(IMes)(PPh$_3$)* (**V**) | | | |
| **[Ir(H)$_2$(IMes)(PPh$_3$)] fragment** | -2066.500012 | 11.4 | -- |
| **(PhAc)$_2$ fragment** | -770.015744 | 5.0 | -- |
| **[Ir(H)$_2$(PhAc)$_2$(IMes)(PPh$_3$)]** | -2836.628285 | -- | -70.6 |
| ***Total*** | | 16.5 | -70.6 |
| | | | |
| *[Ir(H)$_2$(PhPy)$_2$(IMes)(PPh$_3$)* (**V**) | | | |
| **[Ir(H)$_2$(IMes)(PPh$_3$)] fragment** | -2066.487827 | 19.0 | -- |
| **(PhPy)$_2$ fragment** | -958.945172 | 9.5 | -- |
| **[Ir(H)$_2$(PhPy)$_2$(IMes)(PPh$_3$)]** | -3025.546954 | -- | -71.5 |
| ***Total*** | | 28.5 | -71.5 |
| | | | |
| *[Ir(H)$_2$(PhAc)(IMes)(PPh$_3$) C-C rotation* (**TS2**) | | | |
| **[Ir(H)$_2$(IMes)(PPh$_3$)] fragment** | -2066.503048 | 9.5 | |
| **PhAc fragment** | -385.001042 | 6.8 | |
| **TS2** | -2451.557894 | | -33.8 |
| ***Total*** | | 16.3 | -33.8 |
| | | | |
| *[Ir(H)$_2$(PhPy)(IMes)(PPh$_3$) C-C rotation* (**TS2**) | | | |
| **[Ir(H)$_2$(IMes)(PPh$_3$)] fragment** | -2066.491260 | 16.9 | |
| **PhPy fragment** | -479.470263 | 6.2 | |
| **TS2** | -2546.035321 | | -46.3 |
| ***Total*** | | 23.1 | -46.3 |

### 4.4. IRC Electronic Energy Profiles

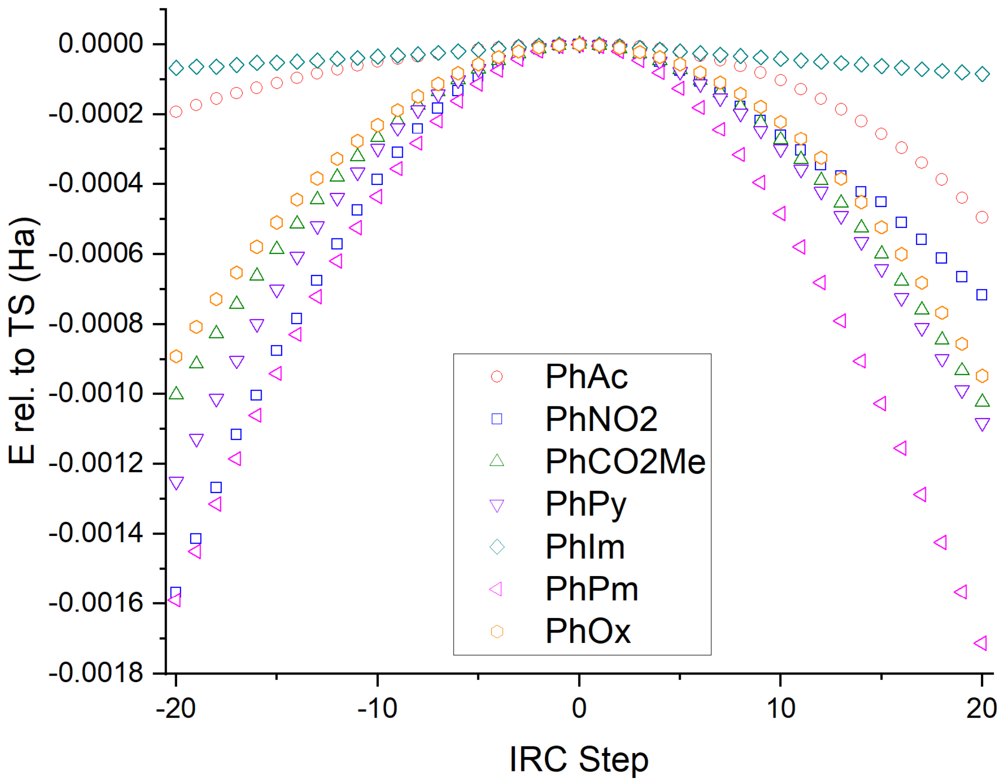


**Figure S25**. Electronic energy plots for **TS2** for each substrate (except 1-phenylpyrazole, see manuscript text).

### 4.5. Coordinates of optimised structures

*These are supplied as a separate file.*

## 5. Literature Data on Lewis Basicity

**Table S27.** Lewis basicity quantified using literature data.

| **Compound** | **Rel. Directing Ability** [21] (*vs* 2-phenylpyridine) | **$BF_3$ Scale** (kJ/mol) | ***p*-$FC_6H_4OH$ Scale** (ΔG°, kJ/mol) | **$I_2$ Scale** (ΔG°, kJ/mol, heptane) | **$K^+$ Affinity** (298 K, kJ/mol) |
|---|---|---|---|---|---|
| ***Parent N-Heterocycles*** | | | | | |
| 1-methyl-imidazole | n/a | | -15.53 [22] | | 117.7 ± 2.7 [23] |
| Pyridine | n/a | 128.08 ± 0.50 [24] | -10.62 [25] | -12.67 [26] | 90.6 ± 3.9 [27] |
| 1-methyl-pyrazole | n/a | | -10.50 [22] | -12.22 [28] | 94.8 ± 3.6 [23] |
| Pyrazole | n/a | | | -10.79 [29] | |
| Pyrimidine | n/a | 113.02 ± 0.21 [30] | -6.11 [25] | -5.59 [31] | 69.7 ± 4.3 [27] |
| ***N*-Heterocyclic Substrates** | | | | | |
| 2-phenyl-pyridine | 1.00 | | -8.16 [25] | | |
| 1-phenyl-pyrazole | 1.77 | | | -5.71 [28] | |
| **Other Substrates** | | | | | |
| Acetophenone | 0.06 | 74.52 ± 0.15 [32] | | | |
| Ethyl benzoate | 0.01 | 61.2 ± 0.8 [32] | | | |
| Nitrobenzene | 0.03 | 35.79 ± 1.40 [24] | | | |

## 6. References.


1 A. Beillard, X. Bantreil, T.-X. Métro, J. Martinez and F. Lamaty, *Dalton Trans.*, 2016, **45**, 17859–17866.

2 M. R. Kumar, K. Park and S. Lee, *Advanced Synthesis & Catalysis*, 2010, **352**, 3255–3266.

3 M. M. Debdab Florence; Bazureau, Jean Pierre, *Synthesis*, 2006, **2006**, 4046–4052.

4 J. Yang, S. Liu, J.-F. Zheng and J. (Steve) Zhou, *European Journal of Organic Chemistry*, 2012, **2012**, 6248–6259.

5 N. A. Yakelis and R. G. Bergman, *Organometallics*, 2005, **24**, 3579–3581.

6 A. J. Martínez-Martínez and A. S. Weller, *Dalton Trans.*, 2019, **48**, 3551–3554.

7 A. R. Cochrane, S. Irvine, W. J. Kerr, M. Reid, S. Andersson and G. N. Nilsson, *Journal of Labelled Compounds and Radiopharmaceuticals*, 2013, **56**, 451–454.

8 A. R. Kennedy, W. J. Kerr, R. Moir and M. Reid, *Org. Biomol. Chem.*, 2014, **12**, 7927–7931.

9 H. Andersson, T. Sainte-Luce Banchelin, S. Das, R. Olsson and F. Almqvist, *Chem. Commun.*, 2010, **46**, 3384–3386.

10 P. Guo, J. M. Joo, S. Rakshit and D. Sames, *J. Am. Chem. Soc.*, 2011, **133**, 16338–16341.

11 J. Wang, S. Wang, G. Wang, J. Zhang and X.-Q. Yu, *Chem. Commun.*, 2012, **48**, 11769–11771.

12 K. L. Billingsley, K. W. Anderson and S. L. Buchwald, *Angewandte Chemie International Edition*, 2006, **45**, 3484–3488.

13 C. Hansch, A. Leo and R. W. Taft, *Chem. Rev.*, 1991, **91**, 165–195.

14 D. S. Timofeeva, D. M. Lindsay, W. J. Kerr and D. J. Nelson, *Catal. Sci. Technol.*, 2021, **11**, 5498–5504.

15 Y. Zhao and D. G. Truhlar, *Theoretical Chemistry Accounts*, 2008, **120**, 215–241.

16 D. Andrae, U. Häußermann, M. Dolg, H. Stoll and H. Preuß, *Theoretica chimica acta*, 1990, **77**, 123–141.

17 G. Luchini, J. Alegre-Requena, I. Funes-Ardoiz and R. Paton, *F1000Research*, DOI:10.12688/f1000research.22758.1.

18 F. Weigend and R. Ahlrichs, *Phys. Chem. Chem. Phys.*, 2005, **7**, 3297–3305.

19 F. Weigend, *Phys. Chem. Chem. Phys.*, 2006, **8**, 1057–1065.

20 A. V. Marenich, C. J. Cramer and D. G. Truhlar, *J. Phys. Chem. B*, 2009, **113**, 6378–6396.

21 D. S. Timofeeva, D. M. Lindsay, W. J. Kerr and D. J. Nelson, *Catal. Sci. Technol.*, 2020, **10**, 7249–7255.

22 C. Laurence and J. F. Gal, in *Lewis Basicity and Affinity Scales*, John Wiley & Sons, Chichester, United Kingdom, 1st edn., 2009, pp. 111–227.

23 H. Huang and M. T. Rodgers, *J. Phys. Chem. A*, 2002, **106**, 4277–4289.

24 P.-C. Maria, J.-F. Gal, J. De Franceschi and E. Fargin, *J. Am. Chem. Soc.*, 1987, **109**, 483–492.

25 M. Berthelot, C. Laurence, M. Safar and F. Besseau, *J. Chem. Soc., Perkin Trans. 2*, 1998, 283–290.

26 C. Laurence and J. F. Gal, in *Lewis Basicity and Affinity Scales*, John Wiley & Sons, Chichester, United Kingdom, 1st edn., 2009, pp. 229–321.

27 R. Amunugama and M. T. Rodgers, *International Journal of Mass Spectrometry*, 2000, **195–196**, 439–457.

28 M. J. El Ghomari, R. Mokhlisse, C. Laurence, J.-Y. Le Questel and M. Berthelot, *Journal of Physical Organic Chemistry*, 1997, **10**, 669–674.

29 J. L. M. Abboud, R. Notario, M. Berthelot, R. M. Claramunt, P. Cabildo, J. Elguero, M. J. El Ghomari, W. Bouab, R. Mokhlisse and G. Guihéneuf, *J. Am. Chem. Soc.*, 1991, **113**, 7489–7493.

30 M. Berthelot, J. F. Gal, C. Laurence and P. C. Maria, *J. Chim. Phys. Phys.-Chim. Biol.*, 1984, **81**, 327–331.

31 P. A. Clark, T. J. Lerner, S. Hayes and S. G. Fischer, *J. Phys. Chem.*, 1976, **80**, 1809–1811.

32 P. C. Maria and J. F. Gal, *J. Phys. Chem.*, 1985, **89**, 1296–1304.
